# Genomic characteristics of cattle copy number variations

**DOI:** 10.1186/1471-2164-12-127

**Published:** 2011-02-23

**Authors:** Yali Hou, George E Liu, Derek M Bickhart, Maria Francesca Cardone, Kai Wang, Eui-soo Kim, Lakshmi K Matukumalli, Mario Ventura, Jiuzhou Song, Paul M VanRaden, Tad S Sonstegard, Curt P Van Tassell

**Affiliations:** 1 Bovine Functional Genomics Laboratory, ANRI, USDA-ARS, Beltsville, Maryland 20705, USA; 2 Department of Animal and Avian Sciences, University of Maryland, College Park, Maryland 20742, USA; 3 Department of Genetics and Microbiology, University of Bari, Bari 70126, Italy; 4 Center for Applied Genomics, Children's Hospital of Philadelphia, Philadelphia, PA 19104, USA; 5 Bioinformatics and Computational Biology, George Mason University, Manassas, VA 20110, USA; 6 Animal Improvement Programs Laboratory, ANRI, USDA-ARS, Beltsville, Maryland 20705, USA

## Abstract

**Background:**

Copy number variation (CNV) represents another important source of genetic variation complementary to single nucleotide polymorphism (SNP). High-density SNP array data have been routinely used to detect human CNVs, many of which have significant functional effects on gene expression and human diseases. In the dairy industry, a large quantity of SNP genotyping results are becoming available and can be used for CNV discovery to understand and accelerate genetic improvement for complex traits.

**Results:**

We performed a systematic analysis of CNV using the Bovine HapMap SNP genotyping data, including 539 animals of 21 modern cattle breeds and 6 outgroups. After correcting genomic waves and considering the pedigree information, we identified 682 candidate CNV regions, which represent 139.8 megabases (~4.60%) of the genome. Selected CNVs were further experimentally validated and we found that copy number "gain" CNVs were predominantly clustered in tandem rather than existing as interspersed duplications. Many CNV regions (~56%) overlap with cattle genes (1,263), which are significantly enriched for immunity, lactation, reproduction and rumination. The overlap of this new dataset and other published CNV studies was less than 40%; however, our discovery of large, high frequency (> 5% of animals surveyed) CNV regions showed 90% agreement with other studies. These results highlight the differences and commonalities between technical platforms.

**Conclusions:**

We present a comprehensive genomic analysis of cattle CNVs derived from SNP data which will be a valuable genomic variation resource. Combined with SNP detection assays, gene-containing CNV regions may help identify genes undergoing artificial selection in domesticated animals.

## Background

With two cattle genome assemblies available (Btau_4 and UMD3)[1,2], the cattle research community has been focusing on single nucleotide polymorphisms (SNPs) as the main source of genetic variation in cattle. This effort led to the development of the cattle SNP map [3] and the Illumina Bovine SNP50 (> 50,000 SNP probes) genotyping array [4,5]. Evaluations of genetic merit based on SNPs became a reality in early 2009 leading to an acceleration of improvements to dairy and beef breed stocks [6-8]. Widespread use of the BovineSNP50 array has resulted in the availability of tens of thousands of SNP genotyping results. Based on SNP genotyping assays, QTL distributions and artificial selection signatures in dairy cattle have been reported [9,10].

Copy Number Variation (CNV) represents another important source of genetic variation that provides genomic structural information complementary to SNP data. Genomic structural variations ranging from 1 kb to 5 Mb comprise mainly of CNVs in the form of large-scale insertions and deletions, as well as inversions and translocations [11]. In humans, ~29,000 CNVs that correspond to over 8,400 CNV regions have been identified, and over 9,000 genes have been mapped within or near regions of human structural variation [12,13]. Some of these CNVs have been shown to be important in both normal phenotypic variability and disease susceptibility. Several recent publications have reviewed the effects of CNVs on gene expression and human diseases [14-17]. Due to their low cost and high-density, SNP arrays have been routinely used for human CNV detection and analysis [13]. Compared to CGH arrays which only report relative signal intensities, SNP arrays collect normalized total intensities (Log R ratio - LRR) and allelic intensity ratios (B allele frequency - BAF) which represent overall copy numbers and allelic contrasts [18]. Multiple algorithms have been developed to exploit SNP data to identify CNVs, including QuantiSNP [19], PennCNV [20], Birdseye [21] and Cokgen [22]. Comparisons of the strengths and weaknesses of these algorithms have been published [23,24]. As one of the leading methods, PennCNV incorporates multiple sources of information, including total signal intensity and allelic intensity ratio at each SNP marker, the distance between neighboring SNPs, and the allele frequency of SNPs. PennCNV also integrates a computational approach by fitting regression models with GC content to overcome "genomic waves" [25,26]. Furthermore, PennCNV is capable of considering pedigree information (a parents-offspring trio) to improve call rates and accuracy of breakpoint prediction as well as to infer chromosome-specific SNP genotypes in CNVs [27].

Previous cattle studies have produced a number of CNV datasets. For example, our earlier array CGH survey using 3 Holstein bulls identified 25 germline CNVs [28]. Recently, we reported a broader, systematic CNV survey in 90 cattle using array CGH [29]. We identified over 200 candidate CNV regions (CNVRs); some of which are likely to underlie cattle domestication and breed formation. Fadista et al. recently reported 304 CNV regions in 20 animals of 4 cattle breeds using high-density array CGH [30]. Besides array CGH experiments, other evidences for cattle CNV came from SNP genotyping results, where a screen of Bovine HapMap Consortium samples (over 500 animals from multiple cattle breeds) identified 79 candidate deletions using an earlier version of cnvPartition [5]. However, these results only included homozygous deletions which were validated by multiple observations. A recent paper reported 368 unique CNV regions from 265 Korean Hanwoo cattle based on BovineSNP50 genotyping data; however, during the PennCNV calling, the "genomic waves" pattern was not discussed and pedigree information was not considered [31]. In this study, we reprocessed the published Bovine HapMap Consortium SNP genotyping results using optimal settings for PennCNV by adjusting for "genomic waves" and utilizing trio/pedigree information whenever possible. We identified 682 candidate CNV regions in a diverse panel of 521 animals from 21 different breeds. We also included 18 animals from 6 outgroups to derive the ancestral states of CNVs. We then compared this CNV call set with the existing cattle CNV call sets, validated several novel CNVR calls and discussed the evolutionary impact of cattle CNVs.

## Results and Discussion

### Optimization of cattle CNV detection

A total of 58,336 markers were selected for the BovineSNP50 assay [4,5]. Except for 1,389 markers which failed to pass manufacturer assay production pipeline, we intentionally kept all remaining 56,947 markers without any other filtering. These included 1,465 markers (2.57%) which had a call rate of 0. The markers with a call rate of 0 are resistant to the default biallelic SNP clustering and often fall in CNV regions. Compared to the standard BovineSNP50 Genotyping Beadchip v1 featuring 54,001 SNP probes, 2,946 more SNPs were included in our analysis, of which, ~17% located in cattle segmental duplication (SD) regions [32], ~9% overlapped with the CNVRs detected by array CGH method [29], and ~27% contributed to the CNVRs reported here.

We tested the cattle CNV calls made with or without the -gcmodel option on Batu_4.0 to identify the impact of genomic waves on CNV calling. Agreeing with previous results [26], we found the total CNVR counts were higher without -gcmodel (719) than those with -gcmodel enabled (682). However, only 86.80% (592/682) of the gcmodel calls directly overlapped with 79.28% (570/719) calls made without gcmodel, revealing a ~20% CNV discordancy rate. These discordant calls were likely due to false positives called from the differentiating signal intensities caused by "genomic waves" rather than by real CNV events. This further demonstrated that genomic waves have a significant effect on this type of analysis.

We also compared results of PennCNV using -test, -trio and -joint options sequentially. In other words, we compared data resultant from not considering trio information (-test), considering trio information only after calling (-trio) and finally by considering trio information in a simultaneous fashion during CNV calling (-joint) (Additional file 1: Table S5). Consistent with the earlier comparisons using simulated and real SNP data [27,33], trio information significantly increased our CNV call rates. The result of the -joint option (1276 calls) was significantly higher than those of the other options: -test (684 calls) and -trio (1019 calls). After merging overlapping CNVs, ~87% of the 682 CNVRs deduced from the -joint option overlapped with those deduced from the -test and -trio options (both with a total of 621 CNVRs). Due to its improved call sensitivity and breakpoint inference, the -joint option reported about 13% more CNVRs which were not detected by the -test or -trio options.

### Cattle CNV discovery and distribution

Due to issues regarding CNVR calls, we excluded chrX and chrUn from our analysis. In our initial analysis of chrX, it was found that almost half of the potential CNVRs were unreasonably large (> 1 Mb) and several events were present in high frequencies (> 25%). This is likely due to the fact that PennCNV assumes two copies of each SNP as the normal copy number state, which was likely not the case within the pseudoautosomal region [34] and segmental duplications [32] on chrX. Additionally, since chrX sequence and annotation also differ dramatically between Btau_4.0 and UMD3 builds, we considered the CNV calls on chrX as unreliable and excluded them from further analysis. Since chrUn only contains unassigned sequence contigs, it was not included due to the lack of sequence and SNPs as well as the SNP mapping uncertainty.

**Figure 1:**
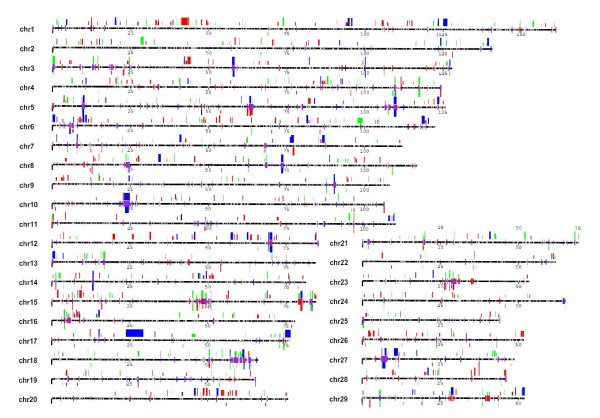
**Genomic landscape of cattle copy number variations and segmental duplications**. CNV regions (682 events, 139 Mb, ~4.60% of the bovine genome) reported by 521 SNP genotyped individuals are shown above the chromosomes in green (gain), red (loss) and dark blue (both), while below are the CNV regions (177 events, 28 Mb, ~1% of the bovine genome) reported by 90 array CGH experiments by Liu et al. The bar height represents their frequencies: short (appeared in 1 sample), median (≥2 samples) and tall (≥5 samples). Segmental duplications (94.4 Mb, 3.1% of the bovine genome) predicted by two independent computational approaches are illustrated on the chromosomes in red (WSSD), blue (WGAC) or purple (both). The patterns are depicted for all duplications for ≥5 kb in length and ≥90% sequence identity. The gaps in the assembly are represented on the chromosomes as white ticks.

Within the placed autosomes, a total of 3,666 CNVs in 521 samples were detected and an average of 7.09 gain or loss events were evident in each sample (Table 1). CNVRs were determined by aggregating overlapping CNVs identified across all samples, following previously published protocols [13]. A total of 682 high-confidence autosomal CNVRs were identified, covering 139.8 Mb of polymorphic sequence and corresponding to 5.49% of the autosomal genome sequence (139.8/2,545.9 Mb) and 4.60% of the whole cattle genome (139.8/3,036.6 Mb, Figure 1 and Additional file 1: Table S2).

**Table 1:** CNV events by species and breeds.

*Btau_4.0*	*Sample*	*Count*	*Unique*	*Gain*	*Loss*	*Gene*	*Total Length*
Taurine^a^	366	2,256(6.23)	239(0.66)	1,454(4.02)	802(2.22)	4,744(13.10)	373,001,599(165,337)
Composite	46	330(7.17)	23(0.50)	224(4.87)	106(2.30)	651(14.15)	113,483,966(142,032)
Indicine	70	799(11.41)	62(0.89)	401(5.73)	398(5.69)	1,464(20.91)	57,402,891(173,948)
African Breeds	39	281(7.21)	38(0.97)	213(5.46)	68(1.74)	775(19.87)	54,728,022(194,761)
CNV^a^	521	3,666(7.09)	362(0.70)	2,292(4.43)	1,374(2.66)	7,634(14.77)	598,616,478(163,288)
CNVR^b^	521	682	278^c^	216^d^	370^d^	1,263	139,786,166(204,965)
Outgroup^e^							
CNV	18	1,003(55.72)	284(15.78)	48(2.67)	955(53.06)	2,603(144.61)	442,235,607(440,912)
CNVR	18	483	187	21	458	1,593	276,846,573(573,181)

The numbers in parentheses are normalized by sample counts except that the lengths in parentheses are average lengths normalized by CNV counts. ^a^At sample level, each sample has 7.09 (3666/517) CNVRs averagely and 6.23 (2256/362) for Taurine, since there are 4 taurine individuals without identified CNVs; ^b ^These numbers are nonredundent CNVR counts. ^c ^278 CNVRs are unique to one sample while 404 CNVRs are shared by at least 2 individuals or breeds and 18 of 404 multiple events have frequency >5%; ^d ^Besides 370 loss and 216 gain CNVRs, there are 96 CNVRs containing both loss and gain events; ^e ^Outgroup animals are not included in the total counts for CNV and CNVR.

To test the stability of CNV calls with respect to different genome builds and SNP mapping, we also migrated 56,408 out of 56,947 SNP markers from Btau_4.0 to UMD3 using the UCSC liftOver tool [35], and repeated the entire calling analyses to ensure consistency in calls (Additional file 1: Tables S3 and S4). Only 61 more CNVRs were identified on the UMD3 assembly (making a total of 743 CNVRs). A simple comparison indicated that the total coverage of variable regions were 13.05% larger on UMD3 (158.0 Mb, Additional file 1: Table S3) than on Btau_4.0. For all three CNVR types (gain, loss and both), counts increased slightly. This was expected as both assemblies were based on the same raw WGS reads. The most obvious difference between the two assemblies is that Btau_4.0 unplaced contigs are placed on UMD3. This resulted in more markers that were on Btau_4.0 ChrUn contigs to be placed on UMD3 autosomes, which could partially explain the increase in the CNVR counts. Since the majority of cattle genome annotations were performed on the Btau_4.0 assembly, we focused on further characterization of the 682 high-confidence CNV regions from Btau_4.0 autosomes.

These 682 CNVRs include 370 loss, 216 gain and 96 both (loss and gain within the same region) events, ranging from 32,566 to 5,569,091 bp with a mean or median of 204,965 or 131,179 bp, respectively (Additional file 1: Table S2). Loss events are approximately 1.7-fold more common than gain events, but have slightly smaller sizes than gain regions on average. Furthermore, 278 CNVRs were found in only one sample (Unique), 404 CNVRs were present in two or more animals or breeds and 18 of 404 multiple events had a frequency >5% (Table 1 and Additional file 1: Table S2). These datasets confirm that segregating CNVs exist among these 21 cattle breeds and/or groups, which is consistent with our earlier results based on array CGH [29]. In general, the number of CNVs identified in each sample is consistent with SNP estimates of breed-specific founding and effective population sizes and levels of polymorphism based on ≥50,000 SNPs [5]. As shown in Table 1, more CNV events were detected in indicine (11.41 per sample) than in African groups (7.21 per sample) and composite (7.17 per sample). The taurine breeds (6.23 per sample) had the fewest detected CNVs. While some of these differences could be related to the fact that the SNP markers were designed based on the Btau_4.0 reference genome (which was derived from the sequence of a Hereford cow of European origin; Dominette 01449), this observation is consistent with the concept of subspecies divergence and supports the hypothesis of multiple independent domestications of cattle in the Fertile Crescent, Southwest Asia and likely Africa [36,37].

Cattle CNVs are distributed in a nonrandom fashion at two different levels. First, CNV content varies significantly among different chromosomes. The proportion of any given known chromosome susceptible to CNV regions varies from 1.32-8.80% (Additional file 1: Table S2). Chromosomes 1 and 6 show the greatest enrichment for CNV (Figure 1 and Additional file 1: Table S2) with almost two-fold of the variable content of the autosomal average. It is interesting to note that these chromosomes do not have the highest SD content [29,32]. Furthermore, similar to the human, mouse, rat and dog genomes, there are a greater proportion of CNVs near pericentromeric and subtelomeric regions. Excluding chrX and chrUn, pericentromeric and subtelomeric regions each represent 3.42% of genomic sequence but show an enrichment of 1.5-2.4-fold more CNVRs (both P values <0.001) and contain 7.78-12.54% of all polymorphic sequence.

### Quality assessment of selected CNV Regions

**Figure 2:**
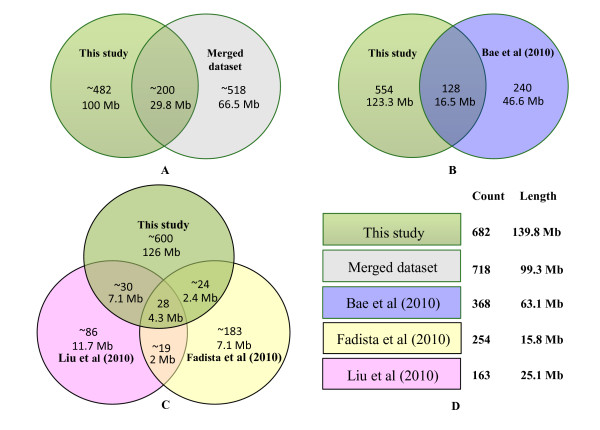
**Comparisons between identified 682 CNVRs in this study and the other existing cattle CNVR datasets in terms of count and length**. A, compared to the total nonredundant CNVR merged from existing published datasets; B, compared to CNVR derived from SNP array (Bae et al, 2010); C, compared to two CNVR datasets derived from array CGH studies (Liu et al,2010; Fadista et al, 2010); D, the summaries and legends of existing cattle CNVR datasets.

The quality of our 682 CNV calls was assessed in multiple ways, though our first assessment was a comparison against existing cattle CNV datasets (Table 2 and Figure 2). One of the earlier datasets included 79 filtered deletion variants (representing 42 unique genomic loci and 9 single SNPs) reported earlier using the Illumina genotyping software module cnvPartition v1.0.2 [5]. Nineteen of our CNVRs overlapped with 11 of the deletion variants (21.57%) in that dataset (Table 2). We also identified 129 CNVRs (18.91%) in our dataset that overlapped with 128 CNVRs from a SNP-based CNV study on 265 Korean Hanwoo cattle [31] (Figure 2B). The Hanwoo CNV study identified 368 CNVRs in total, so our dataset overlapped with 34.83% of their calls [31]. We then compared our calls against an array CGH-based study of 20 cattle from four breeds [30]. Since our dataset excluded CNV calls in the chrX, chrUn and mitochondrial sequences, we compared our autosomal CNVR calls (682 CNVRs) to the autosomal CNV calls of that study (254 CNVRs) [30]. Only 51 of our CNVRs (7.48%) directly overlapped with 55 of their calls (21.65%, Figure 2C). Our final comparison was against our previous array CGH-based study of 90 animals from 14 breeds which resulted in 163 autosomal CNVR calls [29]. In this comparison, 57 of our SNP-based CNVR calls (8.36%) overlapped with 59 CNVRs derived from array CGH (36.20%, Figure 2C). If we only focused on the 16 HapMap samples which were assessed by both platforms (60 CNVRs derived from array CGH and 106 CNVRs reported by SNP array), there were 21 overlapping CNVRs: 19 for array CGH (31.67%, 19/60), and 20 for SNP array (18.87%, 20/106). When we merged existing CNV datasets, a total of ~ 200 out of 682 (about 30%) newly identified CNVRs overlapped with them (Figure 2A).

**Table 2:** Summary of genome-wide studies of cattle copy number variations

Study	Assay	Count			CNVR		Size			
				
		Marker	Sample	Breed	Type	Count	Range (kb)	Median (kb)	Mean (kb)	Total (Mb)
Matukumalli et al. 2009	BovineSNP50	54,001	556	21	Deletion only	51^a^	22.92-11,050.69	394.87	960.67	49.0
Liu et al. 2010	Array CGH	~385,000	90	17	Deletion, insertion	163^b^	18.00-1,261.90	86.19	153.75	25.1
Bae et al. 2010	BovineSNP50	54,001	265	1	Deletion, insertion	368	25.35-967.18	128.33	171.49	63.1
Fadista et al. 2010	Array CGH	~6,300,000	20	4	Deletion, insertion	254^c^	1.72-2,031.34	15.51	62.26	15.8
This study	BovineSNP50	56,947	521	21	Deletion, insertion	682	32.57-5,569.09	131.18	204.97	139.8

^a ^This includes 9 independent SNPs and 42 CNVRs. The statistics are calculated for 42 CNVR excluding the 9 SNPs; ^b ^This is the number excluding chrX and chrUn; ^c ^This is the number excluding chrX, chrUn and mitochondrial sequence.

It is expected that the variants identified in these studies do not overlap, suggesting a vast amount of CNVs exist in cattle population and saturation for this type of variation has not yet been approached. It is likely that many thousands of more common structural variants may still remain undiscovered in the cattle genome. A similar situation was encountered in human CNV studies using the early version of SNP, CGH arrays and detection methods [38,39]. For example, although cnvPartition detects CNVs by processing the similar raw data as PennCNV (i.e. LRR and BAF), it is based on a different proprietary sliding window algorithm. Only those homozygous deletion events segregating in different animals were reported due to concerns with the quality of calls [5]. In the future, high-density SNP arrays combined with improved CNV calling algorithms could remedy these differences.

Besides the technology and detection method differences, the following could also contribute to the observed differences: (1) sampling differences: 521 individuals from 21 diverse breeds and/or groups were included in our study; (2) genome coverage biases: 56,947 markers were included in our study rather than a subset of "well-behaved" SNPs (54,001 markers) which exclude those SNPs in CNV-rich regions; (3) correction of genomic waves in order to minimize false positive calls; and (4) trio/pedigree information was fully explored in our study to improve the accuracy and call rate of CNVs. When filtering criteria varying the CNVR length and frequency were applied, we observed significant overlap within our 2 datasets derived from SNP arrays and array CGH (Additional file 1: Table S8). For example, when the large CNVRs (SNP count = 10, a median ~574kb) were considered, the overlap reached 21.74%. When the CNVR frequency was increased to 1, 2, or 5% (animal count = 5, 10, or 25, respectively), the overlap increased to 89.47%. When we filtered the CNVR frequency to greater than 10% of our population (50 animals), the overlap included 100% of our filtered dataset. This further demonstrated that large, common CNVRs can be reliably detected through using different detection technologies even when the majority of samples were different. For example, our current SNP array study identified most of the large, common CNVRs which were confirmed in our published results [29].

After comparison with other existing datasets, we found that ~70% of our CNVR calls were not reported in the literature. In order to confirm these novel CNVRs, we performed 24 quantitative PCR (qPCR) assays for 15 low frequency, novel CNVR calls spread among seven individuals (Additional file 1: Table S9). Nine of the CNV regions had two target amplicons placed near two different SNP loci. Out of 24 total locations, 11 loci (~48%) were in agreement with CNV estimates by PennCNV (Additional file 1: Table S9 and Figure S1). When counting the CNV regions, 9 out of 15 (60%) CNV regions had positive qPCR confirmations in at least one location. If CNVRs previously validated in the literature [29] were also included, approximately two third (30% + 70% × 48%) of our detected CNVRs had positive confirmations.

As expected, the Bovine SNP50 platform has a large resolution limit under the current PennCNV calling criteria. The size of the CNVRs detected ranged from 32.6 kb to 5.6 Mb, with a median size of 131.2 kb (Additional file 1: Table S2). This is partially due to the fact that the Bovine SNP50 assay was originally developed for high-throughput SNP genotyping in association studies. Although CNV detection is feasible with SNP arrays, it is impaired by low density and non-uniform distribution of SNPs, especially in CNV and SD regions. Compared to a CGH array, a SNP array lacks non-polymorphic probes designed specifically for CNV identification. Thus, only the large CNVRs are expected to be identified with the Bovine SNP50 assay. This explains the difference in CNV length between our study and the earlier results.

The discrepancies between the qPCR and PennCNV results may represent small CNV events that were missed in the PennCNV calls, or instances where SNPs caused the qPCR reaction to fail or be suboptimal but did not affect the SNP assay. Despite the fact that a two-copy state was assumed for test PCR loci in Dominette, smaller CNV events in Dominette may have evaded detection by PennCNV. If our test primers amplified a small CNV event in Dominette, that would skew the relative copy number estimates of our qPCR reactions. Although qPCR primers were designed within 250 bp around the target SNP positions, additional SNPs and small indels may have influenced the hybridization of the qPCR primers in some animals, thereby reducing primer efficiency. Other causes may also contribute to the discrepancy in CNVR validation by qPCR. The draft status of the cattle genome assembly and the low SNP density of the Bovine SNP assay make it difficult to determine the real breakpoints of CNVRs. For example, multiple, neighboring, discrete CNV events could result in a larger call by PennCNV; therefore, giving an over estimation of the CNV size. Therefore, it cannot be ruled out that the qPCR primers used to confirm the CNVRs may have been designed outside the breakpoints.

### CNVs overlap with segmental duplications and other genomic features

Following previous studies of other genomes, we detected the association between CNVRs and SDs. Agreeing with previous predictions regarding cattle SDs [32], a local tandem distribution pattern is predominant in our cattle CNVR dataset (Figure 1). It should be noted that about 25.66% (175/682) of CNV regions directly overlap with cattle SDs with an overlapping span of 16,283,071 bp (11.65% of the total 139,786,166 bp). Approximately 12.06% (356/2952) of the SDs (excluding chrX and chrUn) identified by WGAC and WSSD [32] exhibit CNVs. In comparison, 58.90% of the CNVRs (96/163) detected by using array CGH [29] excluding X chromosome overlap with cattle SDs, corresponding to 15,176,612 bp (60.56% of the total 25,061,646 bp). The proportion of our new CNVR calls (identified in this study) that overlap with SDs reaches approximately 40% compared to 61% in our previous study. This lower overlap fraction probably reflects the fact that the BovineSNP50 array used in this study is biased against cattle SD regions. SNP density on the array drops by one-third (from 21 probes/Mb in unique regions down to 14 probes/Mb) in SD regions. We also failed to detect any correlation between 682 CNV regions and evolutionary breakpoint regions (EBRs). Compared to the genomic averages, cattle-specific EBRs and artiodactyl-specific EBRs do not show enrichments of CNV sequences [40]. This negative result is consent with the fact that EBRs have fewer overlaps with SD regions.

### Gene Content of Cattle CNV regions

Within autosomes, the 682 CNV regions overlap with 1,679 Ensembl peptides, corresponding to 1,263 unique Ensembl genes (Table 1 and Additional file 2: S6). About 55.57% (379/682) of high-confidence CNVRs completely or partially span cattle Ensembl genes. We assigned PANTHER accessions to a total of 1,263 overlapping genes. Statistically significant over or under representations were observed for multiple categories (Additional file 1: Table S10). This set of copy number variable genes possess a wide spectrum of molecular functions, and provides a rich resource for testing hypotheses on the genetic basis of phenotypic variation within and among breeds.

Consistent with similar CNV analyses in other mammals (human, mouse and dog), several of these CNVs, which are important in drug detoxification, defense/innate and adaptive immunity and receptor and signal recognition, are also present in cattle. These gene families include olfactory receptors, ATP-binding cassette (ABC) transporters, Cytochrome P450, β-defensins, interleukins, the bovine MHC (BoLA) and multiple solute carrier family proteins which support the shared GO terms among mammals as shown in Additional file 1: Table S10. For gene families that went through cattle-specific gene duplication [32], such as interferon tau, pregnancy-associated glycoproteins, *SCP2 *and *ULBP *and *WC1.1 *subfamilies, we also detected marked variation in copy number between individuals and across diverse cattle breeds and/or groups (Additional file 2: Table S6). It is intriguing to note that we also detected variations of *TLR3 *(toll-like receptor 3) and *PPARA *(peroxisome proliferator-activated receptor alpha). This current CNV survey further supports a hypothesis that the generation of new CNV insertions and deletions may be a constant phenomenon in multiple cattle breeds/individuals [41].

We also overlapped our CNVRs with two sets of genomic regions under positive or balance selection detected by iHS and F_ST _using SNP data [3,10] (Additional file 3: Table S11). By doing so, we have identified CNV regions that may span potential cattle QTLs and human orthologous OMIM genes influencing disease susceptibility (Additional file 3: Table S11). For instance, multiple CNV regions directly overlap with QTLs for significant and typical economic traits and 87 out of 682 CNVRs correspond to loci known to cause disease in humans. However, since the cattle genome and cattle QTLs are less well defined, future study is warranted.

### Cattle CNV frequency differences among breeds

To highlight the potential evolutionary contributions of these CNVs to cattle breed formation and adaptation, we queried 91 CNVRs that have breed-specific CNV frequency differences (Additional file 1: Table S12). We only considered breeds that had at least 12 samples and any detectable variations must be identified in at least 3 individuals or 10% of samples (for Holstein, Angus and Limousin where n > 30). Fifty-eight of these CNVRs correspond to annotated genes or gene families; many of which were identified in other mammals as influencing adaptation to the environment. Some of the annotated genes are known to be important in cattle adaptation including CNVR266(*IFNT) *in Brown Swiss, CNVR122 (*SCP2) *in Hereford [32] and CNVR178 (Olfactory Receptors) in most of breeds [See also [42]]. The differences of CNV frequency among cattle breeds supported our earlier hypothesis that some cattle CNVs are likely to arise independently in breeds, are likely to contribute to breed differences and are therefore related to the breed formation and adaptation. However, the observed differences between breed variations could be caused by both selection and genetic drift due to genetic bottlenecks for some breeds. Our findings, therefore, must be confirmed with an even larger sample size.

### CNVs in outgroup animals

For the 18 individuals in outgroups, which were analyzed similarly together with 521 modern breeds individuals, 1003 CNVs and 483 CNVRs were detected, covering 276.8 Mb base pairs, with 21 gain and significantly more (458) loss events (Table 1 and Additional file 1: S7). About 34.60% of our current CNVRs (236/682) directly overlapped with 37.47% of these ancient CNVRs (181/483) derived from ancient outgroups, which indicates over one third of CNVRs were likely ancestral. We suspected this observation of more loss events than gain events was at least partially related to the high genetic divergences between these outgroup animals and the cattle reference genome. With additional cattle, sheep, goat and pig CNV papers published recently [43-46], it will be interesting to look into the evolutionary mechanism of CNVs within livestock animals.

## Conclusions

We have performed a comprehensive genomic analysis of cattle CNVs based on whole genome SNP genotyping data, therefore providing a valuable genomic variation resource. A total of 682 CNVRs were identified, covering 139.8 megabases (~4.60%) of the genome. A subset of these CNVRs showed Mendelian inheritance and were also confirmed in other cattle CNV studies and other mammalian species. As high density cattle SNP genotyping data are becoming available, CNVs combined with SNPs, may help identify genes undergoing artificial selection in domesticated animals.

## Methods

### Selection of cattle breeds and animals

It has been demonstrated that the BovineSNP50 genotyping array provides a robust resource for genome-wide, high-density SNP genotyping of cattle and for population genetic analyses of closely related artiodactyl species [4,47]. In which, less than 3% of markers had call rates below 99.94%, the average call rate for individual samples was greater than 97.5% and 85% of samples had call rates above 98.8% [5]. Cattle CNVs in this study were detected by using the same SNP genotyping results, including those collected for the Bovine HapMap project [3] (Additional file 1: Table S1). PennCNV quality filters were applied after the CNV detection, resulting in 521 distinct high quality genotyping results from the original 556 animals. This panel included 366 animals from 14 taurine dairy and beef breeds, 70 animals from three breeds of predominantly indicine background, 46 animals from two breeds that are Taurine × Indicine composites, and 39 animals from two African groups, one of which (Sheko) is an ancient hybrid. It is worth to note that for many of the breeds, individuals were sampled from more than one continent to represent the global cattle population. This panel contained 39 trios where both parents and an offspring were genotyped. Additionally, we included 18 animals from 6 outgroups (*Bos gaurus *- Gaur, *Bos bison *- North American Bison,* Bubalus depressicornis*- Lowland Anoa,* Bos javanicus *- Banteng,* Bos grunniens *- Yak, and *Syncerus caffer *-Cape Buffalo) with 1 trio information to derive the ancestral states of CNVs. Among these outgroups, the average sample call rate was 89.91%, reflecting their divergent relationship from *Bos Taurus*.

### Identification of cattle CNVs

PennCNV algorithm [20] was only applied to autosomes (-lastchr 29) to detect cattle CNV in this study. In our initial analysis, chrX (-chrx) was also considered separately from automosomes. PennCNV incorporates multiple sources of information together, including LRR and BAF at each SNP marker, more realistic models for state transition between different copy number states based on the distance between neighboring SNPs, population frequency of B allele (PFB), the allele frequency of SNPs, and the pedigree information where available, into a hidden Markov model (HMM). Both LRR and BAF were exported from Illumina GenomeStudio Genotyping Module v1.0 software given the default clustering file for each SNP. The PFB file was calculated based on the BAF of each marker in this population. Because there were 153 out of 556 animals (~27.5%) with absolute values of waviness factor larger than 0.04 in our original analysis, the genomic waves were adjusted using the -gcmodel option. The cattle gcmodel file was generated by calculating the GC content of the 1Mb genomic region surrounding each marker (500kb each side). For comparison, the analysis without considering gcmodel was also conducted. Three different PennCNV options were performed wherever possible: 1) -test: the individual-calling algorithm that treats family members as if they were unrelated; 2) -trio: the posterior-calling algorithm which accommodates family information to improve the accuracy of individual-based CNV calling and boundary prediction; 3) -joint: the joint-calling algorithm that identifies CNVs using family data simultaneously. After CNV detection, filtering of low-quality samples was carried out with the default cutoffs: standard deviation (STD) of LRR as 0.30, BAF drift as 0.01 and waviness factor as 0.05. The filtered results from the three algorithms were compared in terms of CNV numbers, lengths and number of SNP in CNVs (Additional file 1: Table S5). The final CNVs set was the nonredundant combination of CNVs from the -joint results for family trio members and the -test results for unrelated individuals. For the outgroup animals, quality filtering was not performed due to their divergent relationship from *Bos Taurus*. CNVRs are determined by aggregating overlapping CNVs identified across all samples [13].

### CNV validation

array CGH experiments were performed as previously described [11]. Primers were designed for qPCR validation using the Primer3 webtool http://frodo.wi.mit.edu/primer3/ by limiting amplicon length to 150bp to 250bp and by incorporating a GC clamp of 2. All other settings were left at the default. Primer information is shown in Additional file 1: Table S9. Quantitative PCR experiments were conducted using SYBR green chemistry in triplicate reactions, each with a reaction volume of 25 μl. All reactions were amplified on a BioRad MyIQ thermocycler. An intron-exon junction of the BTF3 gene was chosen as a reference location for all qPCR experiments. Analysis of resultant crossing thresholds (Ct) was performed using the ΔΔCt method [48,49]. Calibration ΔCt values were derived from amplification of reference and test primers on a genomic DNA template derived from the European Hereford, Dominette 01449. Since all reference and test primers did not overlap with any of Dominette's CNV regions, two-copy states were assumed for both amplicons in Dominette.

### Cattle CNV distribution and association with Segmental Duplications and other features

We investigated the genomic distribution of 682 CNVRs by testing the hypothesis that pericentromeric and subtelomeric regions were enriched for CNVs as described previously [32]. Briefly, all predicted variable bases that overlapped with these regions were totaled and chi-square tests were used to test the null hypothesis of no enrichment. CNVRs were also overlapped with SD and the other genomic features such as evolutionary breakpoint regions (ERBs), which were obtained from literature and public databases listed in web site references.

### Gene content

Gene content of cattle CNV regions was assessed using Ensembl genes ftp://ftp.ensembl.org/pub/current_fasta/bos_taurus/pep/, the Glean consensus gene set, cattle RefSeq and *in silico *mapped human RefSeq (the UCSC Genome Browser website at http://genome.ucsc.edu/). We obtained a catalog of all bovine peptides from Ensembl. This yielded 26,271 peptides, 1,679 of which overlap with predicted 682 high-confidence CNV regions, and correspond to 1,263 unique Ensembl genes. Using the PANTHER classification system, we tested the hypothesis that the PANTHER molecular function, biological process and pathway terms were under- or overrepresented in CNV regions after Bonferroni corrections [32]. It is worth noting that a portion of the genes in the bovine genome has not been annotated or has been annotated with unknown function, which may influence the outcome of this analysis.

## Web Site References

The Database of Genomic Variants: http://projects.tcag.ca/variation/

Ensembl genes ftp://ftp.ensembl.org/pub/current_fasta/bos_taurus/pep/

PANTHER http://www.pantherdb.org/

OMIM http://www.ncbi.nlm.nih.gov/omim/

OMIA http://omia.angis.org.au/

QTL http://www.animalgenome.org/

## Authors' contributions

YH, KW, EK, LKM, JS carried out computational analysis. DMB, MFC, MV carried out the experimental validations. PMV, TSS and CPVT participated in data collection. GEL conceived of the study and led in its design and coordination. All authors contributed to writing the manuscript, read and approved the final manuscript.

## Supplementary Material

Additional file 1 **Supplemental Material file**. Table S1. Numbers of species, breeds, animals and trios used to call CNVs genotyped by BovineSNP50 assay. Table S2. Btau_4.0 cattle CNV regions and their frequencies. Table S3. Comparison of CNV regions identified on two cattle genome assemblies. Table S4. UMD3 cattle CNV regions and their frequencies. Table S5. The comparison of CNVs from 39 trios using three CNV calling algorithms: individual-calling, posterior-calling and joint-calling. Table S7. Outgroup CNV regions and their frequencies. Table S8. The effects of CNV length and frequency on calling consistances between CNV callings based on SNP array and aCGH. Table S9. qPCR Summary. Table S10. Over/Underrepresentation of PANTHER molecular function, biological process and pathway terms. Table S12. CNVR frequency differences among breeds. Figure S1. Illustration of a typical CNV call with qPCR validation. Click here for file

Additional file 2 **Supplemental Material file**. Table S6. Gene contents of cattle CNV regions. Click here for file

Additional file 3 **Supplemental Material file**. Table S11. Cattle CNV regions overlap with genomic regions under positive selection, human orthologous OMIM genes and cattle QTLs. Click here for file
