# Supplementary material for: Genomic characteristics of cattle copy number variations
Source: BMC Genomics. 2011 Feb 23;12:127. doi: 10.1186/1471-2164-12-127 (PMC3053260; doi:10.1186/1471-2164-12-127)
Supplement: Additional file 1 — Supplemental Material file. Table S1. Numbers of species, breeds, animals and trios used to call CNVs genotyped by BovineSNP50 assay. Table S2. Btau_4.0 cattle CNV regions and their frequencies. Table S3. Comparison of CNV regions identified on two cattle genome assemblies. Table S4. UMD3 cattle CNV regions and their frequencies. Table S5. The comparison of CNVs from 39 trios using three CNV calling algorithms: individual-calling, posterior-calling and joint-calling. Table S7. Outgroup CNV regions and their frequencies. Table S8. The effects of CNV length and frequency on calling consistances between CNV callings based on SNP array and aCGH. Table S9. qPCR Summary. Table S10. Over/Underrepresentation of PANTHER molecular function, biological process and pathway terms. Table S12. CNVR frequency differences among breeds. Figure S1. Illustration of a typical CNV call with qPCR validation. [file 1471-2164-12-127-S1.DOC]

**Table S1. Numbers of species, breeds, animals and trios used to call CNVs genotyped by BovineSNP50 assay.**

**Table S2. Btau_4.0 cattle CNV regions and their frequencies.**

**Table S3. Comparison of CNV regions identified on two cattle genome assemblies.**

**Table S4. UMD3 cattle CNV regions and their frequencies.**

**Table S5. The comparison of CNVs from 39 trios using three CNV calling algorithms: individual-calling, posterior-calling and joint-calling.**

**Table S6. Gene contents of cattle CNV regions. See Additional file 2: Table S6.xls.**

**Table S7. Outgroup CNV regions and their frequencies.**

**Table S8. The effects of CNV length and frequency on calling consistances between CNV callings based on SNP array and aCGH.**

**Table S9. qPCR Summary.**

**Table S10. Over/Underrepresentation of PANTHER molecular function, biological process and pathway terms.**

**Table S11. Cattle CNV regions overlap with genomic regions under positive selection, human orthologous OMIM genes and cattle QTLs. See Additional file 3: Table S11.xls.**

**Table S12. CNVR frequency differences among breeds.**

**Figure S1. Illustration of a typical CNV call with qPCR validation.**

**Table S1. Numbers of species, breeds, animals and trios used to call CNVs genotyped by BovineSNP50 assay.**

| ***Breed or species*** | ***Acronym*** | ***# Animals*** | ***# Males*** | ***# Females*** | ***# Trios*** |
| --- | --- | --- | --- | --- | --- |
| ***Taurine*** |  |  |  |  |  |
| Holstein | HOL | 58 | 49 | 9 | 3 |
| Angus | ANG | 59 | 55 | 4 | 3 |
| Limousin | LMS | 41 | 38 | 3 | 2 |
| Hereford | HFD | 24 | 20 | 4 | 2 |
| Jersey | JER | 28 | 25 | 3 | 3 |
| Charolais | CHL | 25 | 19 | 6 | 3 |
| Brown Swiss | BSW | 24 | 21 | 3 | 3 |
| Piedmontese | PMT | 22 | 14 | 8 | 3 |
| Ramagnola | RMG | 23 | 21 | 2 | 2 |
| Guernsey | GNS | 21 | 1 | 20 | 1 |
| Norwegian Red | NRC | 21 | 16 | 5 | 1 |
| Red Angus | RGU | 14 | 13 | 1 | 0 |
| Gelbvieh | GBV | 3 | 3 | 0 | 0 |
| Simmental | SIM | 3 | 3 | 0 | 0 |
|  |  |  |  |  |  |
| ***Indicine*** |  |  |  |  |  |
| Brahman | BRM | 25 | 12 | 13 | 3 |
| Gir | GIR | 24 | 20 | 4 | 3 |
| Nelore | NEL | 21 | 9 | 12 | 1 |
|  |  |  |  |  |  |
| ***Taurine×Indicine*** |  |  |  |  |  |
| Beefmaster | BMA | 23 | 22 | 1 | 1 |
| Santa Gertrudis | SGT | 23 | 20 | 3 | 3 |
|  |  |  |  |  |  |
| ***African Breeds*** |  |  |  |  |  |
| N'Dama (Taurine) | NDA | 22 | 5 | 17 | 1 |
| Sheko  (Ancient Taurine×Indicine hybrid) | SHK | 17 | 5 | 12 | 1 |
|  |  |  |  |  |  |
| ***Outgroup Species*** |  |  |  |  |  |
| Gaur (*Bos gaurus*) | OGR | 4 | 0 | 4 | 0 |
| North American Bison (*Bos bison*) | OBB | 4 | 2 | 2 | 1 |
| Lowland Anoa  (*Bubalus depressicornis*) | OWB | 4 | 3 | 1 | 0 |
| Banteng (*Bos javanicus*) | OBJ | 2 | 2 | 0 | 0 |
| Yak (*Bos grunniens*) | OYK | 2 | 2 | 0 | 0 |
| Cape Buffalo (*Syncerus caffer*) | OCB | 2 | NA | NA | 0 |
|  |  |  |  |  |  |
| **Total** |  | 539 | 400 | 137 | 40 |

**Table S2. Btau_4.0 cattle CNV regions and their frequencies.**

| ***No*** | ***Chr*** | ***Start*** | ***End*** | ***Length*** | ***Type*** | ***StartSNP*** | ***EndSNP*** | ***No. SNP*** | ***Count*** | ***Frequency*** |
| --- | --- | --- | --- | --- | --- | --- | --- | --- | --- | --- |
| 1 | chr1 | 1,247,321 | 1,458,242 | 210,922 | gain | ARS-BFGL-NGS-62826 | ARS-BFGL-BAC-4980 | 7 | 3 | 0.58% |
| 2 | chr1 | 1,705,125 | 1,791,641 | 86,517 | gain | ARS-BFGL-NGS-25183 | ARS-BFGL-BAC-7317 | 4 | 1 | Unique |
| 3 | chr1 | 4,324,291 | 4,588,701 | 264,411 | both | Hapmap33075-BTA-162916 | BTB-01973385 | 5 | 3 | 0.58% |
| 4 | chr1 | 11,324,871 | 11,498,897 | 174,027 | loss | Hapmap43814-BTA-22629 | BTA-29334-no-rs | 5 | 1 | Unique |
| 5 | chr1 | 12,265,746 | 12,544,191 | 278,446 | loss | BTB-00005637 | Hapmap60346-rs29019231 | 4 | 8 | 1.54% |
| 6 | chr1 | 14,238,026 | 14,360,343 | 122,318 | loss | BTA-103292-no-rs | ARS-BFGL-BAC-14883 | 4 | 1 | Unique |
| 7 | chr1 | 17,178,059 | 17,278,945 | 100,887 | loss | BTA-118731-no-rs | BTB-01083468 | 4 | 1 | Unique |
| 8 | chr1 | 19,851,471 | 20,265,533 | 414,063 | both | BTA-39399-no-rs | BTA-39450-no-rs | 9 | 5 | 0.96% |
| 9 | chr1 | 20,373,074 | 20,421,151 | 48,078 | loss | Hapmap49594-BTA-39447 | ARS-BFGL-NGS-10334 | 3 | 3 | 0.58% |
| 10 | chr1 | 21,595,039 | 21,658,255 | 63,217 | loss | BTB-01210202 | BTB-01210076 | 3 | 1 | Unique |
| 11 | chr1 | 23,551,250 | 23,694,843 | 143,594 | loss | BTB-01153392 | BTB-01153241 | 3 | 2 | Multiple |
| 12 | chr1 | 27,204,282 | 27,415,115 | 210,834 | loss | Hapmap51561-BTA-120459 | Hapmap33501-BTA-152681 | 5 | 1 | Unique |
| 13 | chr1 | 30,006,608 | 30,066,112 | 59,505 | gain | BTB-00012627 | BFGL-NGS-119297 | 3 | 2 | Multiple |
| 14 | chr1 | 32,878,144 | 33,296,052 | 417,909 | loss | Hapmap52004-BTA-69178 | Hapmap40138-BTA-69192 | 11 | 3 | 0.58% |
| 15 | chr1 | 33,637,325 | 33,711,422 | 74,098 | loss | BTA-69212-no-rs | BTB-00015939 | 3 | 1 | Unique |
| 16 | chr1 | 33,897,882 | 33,983,402 | 85,521 | loss | BTA-69229-no-rs | BTB-00901868 | 3 | 2 | Multiple |
| 17 | chr1 | 34,987,550 | 35,047,499 | 59,950 | loss | Hapmap43629-BTA-60810 | BTB-01211075 | 3 | 1 | Unique |
| 18 | chr1 | 37,718,260 | 37,962,259 | 244,000 | loss | Hapmap52575-rs29013932 | Hapmap46343-BTA-111768 | 8 | 1 | Unique |
| 19 | chr1 | 38,372,695 | 38,620,893 | 248,199 | loss | Hapmap29197-BTA-97143 | ARS-BFGL-NGS-53463 | 5 | 3 | 0.58% |
| 20 | chr1 | 39,645,815 | 39,716,032 | 70,218 | gain | ARS-BFGL-BAC-15722 | BTA-07775-no-rs | 3 | 9 | 1.73% |
| 21 | chr1 | 39,820,991 | 39,933,731 | 112,741 | gain | Hapmap33732-BTA-160835 | Hapmap39970-BTA-117984 | 3 | 1 | Unique |
| 22 | chr1 | 40,691,263 | 40,758,983 | 67,721 | gain | ARS-BFGL-NGS-55025 | Hapmap38790-BTA-87413 | 3 | 1 | Unique |
| 23 | chr1 | 41,168,633 | 43,216,667 | 2,048,035 | loss | BTB-01249999 | ARS-BFGL-BAC-15157 | 51 | 7 | 1.34% |
| 24 | chr1 | 43,461,071 | 43,522,084 | 61,014 | loss | BTB-01708786 | BTB-01955018 | 3 | 7 | 1.34% |
| 25 | chr1 | 45,647,138 | 45,704,661 | 57,524 | loss | BTB-00021257 | ARS-BFGL-NGS-81540 | 3 | 2 | Multiple |
| 26 | chr1 | 45,898,622 | 46,114,237 | 215,616 | gain | BTB-01788892 | BTB-02044553 | 5 | 2 | Multiple |
| 27 | chr1 | 47,565,937 | 47,729,242 | 163,306 | loss | ARS-BFGL-NGS-69858 | BTB-01625181 | 3 | 2 | Multiple |
| 28 | chr1 | 49,221,200 | 49,403,484 | 182,285 | loss | ARS-BFGL-NGS-50906 | ARS-BFGL-NGS-41264 | 7 | 4 | 0.77% |
| 29 | chr1 | 49,614,529 | 49,754,204 | 139,676 | loss | BTB-01146938 | Hapmap50572-BTA-113037 | 5 | 1 | Unique |
| 30 | chr1 | 50,555,211 | 50,631,826 | 76,616 | loss | BTB-00022142 | BTB-01165109 | 3 | 1 | Unique |
| 31 | chr1 | 51,972,286 | 52,088,249 | 115,964 | gain | BTB-00023604 | BTB-00023392 | 4 | 1 | Unique |
| 32 | chr1 | 59,799,327 | 59,853,274 | 53,948 | loss | ARS-USMARC-Parent-DQ404150-rs29012530 | ARS-BFGL-NGS-16908 | 4 | 1 | Unique |
| 33 | chr1 | 60,686,366 | 60,751,595 | 65,230 | loss | BTA-31643-no-rs | BTB-00028937 | 3 | 2 | Multiple |
| 34 | chr1 | 61,749,988 | 61,816,725 | 66,738 | loss | Hapmap41191-BTA-16674 | Hapmap59735-rs29016420 | 3 | 1 | Unique |
| 35 | chr1 | 72,076,618 | 72,224,768 | 148,151 | loss | Hapmap43841-BTA-34601 | Hapmap40324-BTA-34602 | 4 | 1 | Unique |
| 36 | chr1 | 73,659,512 | 73,981,192 | 321,681 | gain | ARS-BFGL-NGS-59448 | ARS-BFGL-NGS-4162 | 4 | 6 | 1.15% |
| 37 | chr1 | 77,668,174 | 77,789,354 | 121,181 | loss | ARS-BFGL-NGS-54199 | ARS-BFGL-BAC-31482 | 4 | 1 | Unique |
| 38 | chr1 | 83,964,513 | 84,071,804 | 107,292 | loss | ARS-BFGL-NGS-19217 | BTB-01059241 | 4 | 1 | Unique |
| 39 | chr1 | 90,954,551 | 91,027,727 | 73,177 | loss | BTB-01196514 | Hapmap27729-BTA-108726 | 3 | 2 | Multiple |
| 40 | chr1 | 93,782,655 | 94,369,685 | 587,031 | loss | BFGL-NGS-115400 | Hapmap52705-rs29019397 | 16 | 1 | Unique |
| 41 | chr1 | 94,552,473 | 94,958,244 | 405,772 | both | BTA-17925-no-rs | BTA-07846-rs29027508 | 8 | 17 | 3.26% |
| 42 | chr1 | 95,616,570 | 95,930,224 | 313,655 | both | ARS-BFGL-BAC-36882 | BTB-00768631 | 9 | 13 | 2.50% |
| 43 | chr1 | 103,356,064 | 103,501,423 | 145,360 | loss | BTB-01136854 | BTB-01136812 | 5 | 2 | Multiple |
| 44 | chr1 | 105,349,689 | 105,829,264 | 479,576 | loss | BTB-00006833 | Hapmap38948-BTA-28757 | 12 | 4 | 0.77% |
| 45 | chr1 | 107,216,857 | 107,399,663 | 182,807 | loss | BTB-00048807 | Hapmap54763-rs29012506 | 5 | 1 | Unique |
| 46 | chr1 | 113,500,011 | 113,546,579 | 46,569 | loss | ARS-BFGL-BAC-6549 | Hapmap43087-BTA-87783 | 3 | 2 | Multiple |
| 47 | chr1 | 114,148,558 | 114,216,362 | 67,805 | loss | BTB-00738261 | DIAS-1 | 3 | 3 | 0.58% |
| 48 | chr1 | 114,871,330 | 115,085,095 | 213,766 | loss | Hapmap61008-rs29023119 | Hapmap42001-BTA-89523 | 3 | 2 | Multiple |
| 49 | chr1 | 121,845,903 | 121,931,242 | 85,340 | gain | BTA-22577-no-rs | ARS-BFGL-NGS-64650 | 4 | 2 | Multiple |
| 50 | chr1 | 123,290,078 | 123,610,350 | 320,273 | both | BTB-01793232 | Hapmap43795-BTA-16918 | 7 | 7 | 1.34% |
| 51 | chr1 | 124,989,231 | 126,365,382 | 1,376,152 | both | ARS-BFGL-BAC-14851 | Hapmap59026-rs29022289 | 30 | 10 | 1.92% |
| 52 | chr1 | 127,725,447 | 127,803,933 | 78,487 | loss | BFGL-NGS-113021 | ARS-BFGL-NGS-100109 | 3 | 1 | Unique |
| 53 | chr1 | 130,965,630 | 131,023,932 | 58,303 | gain | Hapmap39600-BTA-89434 | BFGL-NGS-116159 | 3 | 3 | 0.58% |
| 54 | chr1 | 141,468,989 | 141,618,694 | 149,706 | gain | ARS-BFGL-NGS-14502 | Hapmap51743-BTA-54330 | 4 | 1 | Unique |
| 55 | chr1 | 149,363,846 | 149,438,776 | 74,931 | gain | ARS-BFGL-NGS-21137 | ARS-BFGL-NGS-46522 | 3 | 1 | Unique |
| 56 | chr1 | 149,767,231 | 149,835,950 | 68,720 | gain | ARS-BFGL-NGS-20306 | ARS-BFGL-NGS-18738 | 3 | 3 | 0.58% |
| 57 | chr1 | 153,670,496 | 153,966,569 | 296,074 | gain | BTA-16239-no-rs | BTA-57354-no-rs | 7 | 3 | 0.58% |
| 58 | chr1 | 156,320,504 | 156,390,945 | 70,442 | loss | Hapmap28036-BTA-125012 | BTA-58309-no-rs | 3 | 1 | Unique |
| 59 | chr1 | 157,661,921 | 157,758,037 | 96,117 | loss | BTB-00023885 | BTB-00023937 | 3 | 1 | Unique |
| 60 | chr1 | 160,532,854 | 160,590,619 | 57,766 | gain | BTB-00075865 | BTA-15338-no-rs | 3 | 1 | Unique |
| 61 | chr2 | 1,352,094 | 1,402,725 | 50,632 | loss | ARS-BFGL-NGS-39059 | Hapmap58923-rs29010138 | 3 | 2 | Multiple |
| 62 | chr2 | 4,395,448 | 4,478,568 | 83,121 | gain | BTB-00078262 | ARS-BFGL-NGS-105116 | 3 | 4 | 0.77% |
| 63 | chr2 | 8,289,738 | 8,501,993 | 212,256 | gain | UA-IFASA-9562 | BTA-85695-no-rs | 5 | 1 | Unique |
| 64 | chr2 | 8,925,959 | 8,971,940 | 45,982 | gain | ARS-BFGL-NGS-65047 | Hapmap40313-BTA-27938 | 3 | 2 | Multiple |
| 65 | chr2 | 9,337,707 | 9,664,438 | 326,732 | loss | Hapmap50551-BTA-104382 | BTB-00079257 | 11 | 3 | 0.58% |
| 66 | chr2 | 12,426,923 | 12,587,408 | 160,486 | loss | Hapmap44546-BTA-48673 | Hapmap46046-BTA-114001 | 3 | 1 | Unique |
| 67 | chr2 | 14,067,215 | 14,156,827 | 89,613 | loss | Hapmap39412-BTA-48779 | ARS-BFGL-NGS-38281 | 4 | 2 | Multiple |
| 68 | chr2 | 14,916,341 | 14,985,891 | 69,551 | both | Hapmap52534-rs29010223 | Hapmap38137-BTA-112893 | 3 | 3 | 0.58% |
| 69 | chr2 | 15,844,221 | 15,941,973 | 97,753 | gain | BTB-01084313 | ARS-BFGL-BAC-29210 | 3 | 2 | Multiple |
| 70 | chr2 | 18,634,999 | 18,875,569 | 240,571 | both | BTB-00081145 | Hapmap30496-BTA-123052 | 9 | 3 | 0.58% |
| 71 | chr2 | 21,649,588 | 21,706,967 | 57,380 | loss | BTB-00083524 | Hapmap48774-BTA-46697 | 3 | 1 | Unique |
| 72 | chr2 | 28,290,230 | 29,014,103 | 723,874 | gain | BTB-00091195 | ARS-BFGL-NGS-40971 | 21 | 6 | 1.15% |
| 73 | chr2 | 29,787,820 | 29,871,688 | 83,869 | loss | ARS-BFGL-BAC-30043 | BTB-01494156 | 4 | 1 | Unique |
| 74 | chr2 | 32,515,385 | 32,592,374 | 76,990 | gain | Hapmap52594-rs29026993 | BTB-00092571 | 3 | 2 | Multiple |
| 75 | chr2 | 43,141,034 | 43,294,132 | 153,099 | loss | Hapmap48777-BTA-47434 | Hapmap46653-BTA-47447 | 7 | 1 | Unique |
| 76 | chr2 | 54,417,203 | 54,544,859 | 127,657 | gain | ARS-BFGL-NGS-59320 | BTA-47656-no-rs | 5 | 1 | Unique |
| 77 | chr2 | 56,689,251 | 56,721,816 | 32,566 | loss | BTA-122373-no-rs | BTB-01775964 | 3 | 1 | Unique |
| 78 | chr2 | 58,250,628 | 58,580,086 | 329,459 | loss | Hapmap27618-BTA-152634 | BTB-01588936 | 10 | 5 | 0.96% |
| 79 | chr2 | 59,718,306 | 59,832,175 | 113,870 | loss | BTA-19224-no-rs | BTB-00099884 | 4 | 19 | 3.65% |
| 80 | chr2 | 66,654,373 | 66,884,779 | 230,407 | gain | ARS-BFGL-NGS-42334 | ARS-BFGL-NGS-94 | 5 | 8 | 1.54% |
| 81 | chr2 | 70,268,963 | 70,508,564 | 239,602 | loss | BTA-24669-no-rs | ARS-BFGL-NGS-38034 | 6 | 2 | Multiple |
| 82 | chr2 | 70,838,276 | 70,962,445 | 124,170 | loss | BTB-01654063 | BTB-01297078 | 4 | 2 | Multiple |
| 83 | chr2 | 76,547,298 | 76,628,976 | 81,679 | gain | Hapmap52909-rs29011193 | ARS-BFGL-NGS-100817 | 3 | 1 | Unique |
| 84 | chr2 | 81,219,099 | 81,288,597 | 69,499 | loss | BTA-98940-no-rs | BTA-98942-no-rs | 3 | 1 | Unique |
| 85 | chr2 | 81,861,373 | 82,032,203 | 170,831 | loss | BTB-01767855 | BTB-00103489 | 7 | 2 | Multiple |
| 86 | chr2 | 85,079,752 | 85,391,095 | 311,344 | loss | BTA-89488-no-rs | BTA-105824-no-rs | 4 | 2 | Multiple |
| 87 | chr2 | 91,745,584 | 92,032,671 | 287,088 | both | BTA-116567-no-rs | BFGL-NGS-119235 | 4 | 3 | 0.58% |
| 88 | chr2 | 97,569,469 | 97,639,061 | 69,593 | loss | DIAS-311 | Hapmap33390-BTA-157206 | 3 | 1 | Unique |
| 89 | chr2 | 116,296,776 | 116,360,343 | 63,568 | gain | BTA-94318-no-rs | BTB-00112049 | 3 | 1 | Unique |
| 90 | chr2 | 116,577,788 | 116,655,227 | 77,440 | gain | ARS-BFGL-NGS-27703 | BFGL-NGS-111877 | 3 | 2 | Multiple |
| 91 | chr2 | 118,921,732 | 119,028,634 | 106,903 | loss | Hapmap27370-BTA-123236 | Hapmap49461-BTA-115001 | 4 | 2 | Multiple |
| 92 | chr2 | 124,176,319 | 124,379,630 | 203,312 | both | INRA-102 | Hapmap38678-BTA-49219 | 7 | 8 | 1.54% |
| 93 | chr2 | 126,042,823 | 126,114,742 | 71,920 | gain | BFGL-NGS-119638 | BTA-49594-no-rs | 3 | 2 | Multiple |
| 94 | chr2 | 129,269,182 | 129,364,891 | 95,710 | loss | BFGL-NGS-117131 | BFGL-NGS-116217 | 3 | 2 | Multiple |
| 95 | chr2 | 130,570,719 | 130,628,150 | 57,432 | loss | ARS-BFGL-BAC-32408 | BFGL-NGS-118505 | 3 | 7 | 1.34% |
| 96 | chr2 | 137,634,388 | 138,018,047 | 383,660 | gain | BFGL-NGS-110390 | ARS-BFGL-NGS-66860 | 14 | 1 | Unique |
| 97 | chr2 | 138,859,369 | 139,004,087 | 144,719 | loss | ARS-BFGL-NGS-14190 | ARS-BFGL-NGS-17681 | 5 | 1 | Unique |
| 98 | chr2 | 139,133,717 | 139,665,915 | 532,199 | both | BFGL-NGS-115989 | ARS-BFGL-NGS-27654 | 18 | 19 | 3.65% |
| 99 | chr3 | 743 | 783,652 | 782,910 | gain | BTB-01996597 | Hapmap42163-BTA-27347 | 12 | 11 | 2.11% |
| 100 | chr3 | 2,381,696 | 2,475,960 | 94,265 | loss | ARS-BFGL-NGS-15921 | ARS-BFGL-NGS-654 | 3 | 1 | Unique |
| 101 | chr3 | 5,442,010 | 5,539,104 | 97,095 | loss | Hapmap39882-BTA-106655 | ARS-BFGL-NGS-7652 | 3 | 1 | Unique |
| 102 | chr3 | 5,733,836 | 6,024,177 | 290,342 | loss | ARS-BFGL-NGS-50158 | BTB-01234863 | 5 | 1 | Unique |
| 103 | chr3 | 12,903,718 | 13,253,595 | 349,878 | gain | ARS-BFGL-NGS-25865 | INRA-237 | 4 | 5 | 0.96% |
| 104 | chr3 | 14,393,518 | 14,589,463 | 195,946 | gain | BFGL-NGS-111504 | ARS-BFGL-NGS-3037 | 6 | 5 | 0.96% |
| 105 | chr3 | 23,900,198 | 23,987,162 | 86,965 | loss | BFGL-NGS-117867 | BTB-00123279 | 4 | 1 | Unique |
| 106 | chr3 | 24,038,997 | 24,238,044 | 199,048 | loss | ARS-BFGL-NGS-16066 | INRA-257 | 3 | 1 | Unique |
| 107 | chr3 | 24,484,313 | 24,739,952 | 255,640 | gain | BTA-93841-no-rs | Hapmap26676-BTA-141146 | 9 | 27 | 5.18% |
| 108 | chr3 | 41,821,890 | 41,868,771 | 46,882 | loss | Hapmap58660-rs29011628 | BTA-93899-no-rs | 3 | 3 | 0.58% |
| 109 | chr3 | 42,084,450 | 42,180,969 | 96,520 | gain | BTB-01737920 | BTB-01155279 | 3 | 2 | Multiple |
| 110 | chr3 | 42,218,097 | 42,265,299 | 47,203 | both | BTB-01155190 | BTB-01155098 | 3 | 5 | 0.96% |
| 111 | chr3 | 42,717,282 | 42,985,746 | 268,465 | loss | Hapmap52326-rs29020672 | BTB-01436290 | 9 | 1 | Unique |
| 112 | chr3 | 43,200,031 | 44,190,402 | 990,372 | loss | BTB-01405443 | Hapmap33718-BTA-152247 | 19 | 5 | 0.96% |
| 113 | chr3 | 49,778,568 | 49,933,149 | 154,582 | loss | BTB-01463390 | Hapmap27022-BTA-159739 | 6 | 4 | 0.77% |
| 114 | chr3 | 51,305,265 | 51,443,092 | 137,828 | loss | Hapmap32815-BTA-141391 | BTB-00127606 | 5 | 3 | 0.58% |
| 115 | chr3 | 57,559,243 | 58,233,307 | 674,065 | both | INRA-240 | INRA-241 | 14 | 86 | 16.51% |
| 116 | chr3 | 64,219,875 | 64,297,079 | 77,205 | loss | BTB-01323076 | BTB-01322824 | 4 | 1 | Unique |
| 117 | chr3 | 66,074,357 | 66,194,505 | 120,149 | loss | BTB-02017777 | BTB-01210458 | 4 | 1 | Unique |
| 118 | chr3 | 68,276,498 | 68,369,473 | 92,976 | loss | BTA-68158-no-rs | BTB-00131667 | 4 | 1 | Unique |
| 119 | chr3 | 68,897,722 | 69,118,846 | 221,125 | loss | Hapmap52640-rs29027369 | BTA-118190-no-rs | 5 | 1 | Unique |
| 120 | chr3 | 82,102,949 | 82,232,441 | 129,493 | both | BTB-01509140 | BTB-01789933 | 5 | 5 | 0.96% |
| 121 | chr3 | 99,323,038 | 99,442,972 | 119,935 | loss | ARS-BFGL-NGS-55541 | BTB-00144361 | 4 | 2 | Multiple |
| 122 | chr3 | 100,075,814 | 100,191,284 | 115,471 | both | BTB-01604189 | ARS-BFGL-NGS-14376 | 5 | 10 | 1.92% |
| 123 | chr3 | 122,623,225 | 122,811,999 | 188,775 | loss | Hapmap39506-BTA-69666 | ARS-BFGL-NGS-42740 | 3 | 1 | Unique |
| 124 | chr3 | 123,708,545 | 123,748,949 | 40,405 | loss | BTB-00719198 | ARS-BFGL-NGS-15642 | 3 | 1 | Unique |
| 125 | chr3 | 126,097,657 | 126,380,127 | 282,471 | gain | Hapmap52849-rs29021928 | INRA-192 | 9 | 1 | Unique |
| 126 | chr3 | 127,123,475 | 127,304,446 | 180,972 | both | BTB-00297406 | ARS-BFGL-NGS-105794 | 7 | 4 | 0.77% |
| 127 | chr4 | 6,067,893 | 6,441,010 | 373,118 | gain | ARS-BFGL-NGS-17038 | ARS-BFGL-NGS-66869 | 6 | 2 | Multiple |
| 128 | chr4 | 9,544,381 | 9,640,761 | 96,381 | loss | BTA-96426-no-rs | BTB-00165658 | 3 | 1 | Unique |
| 129 | chr4 | 10,640,831 | 10,801,271 | 160,441 | both | ARS-BFGL-NGS-64636 | ARS-BFGL-NGS-36252 | 5 | 2 | Multiple |
| 130 | chr4 | 18,365,091 | 18,466,019 | 100,929 | loss | BTB-01176772 | BTA-68467-no-rs | 3 | 2 | Multiple |
| 131 | chr4 | 20,227,219 | 20,432,461 | 205,243 | loss | BTB-01108314 | ARS-BFGL-NGS-108298 | 3 | 2 | Multiple |
| 132 | chr4 | 20,892,617 | 21,022,961 | 130,345 | gain | BTA-38554-no-rs | BTA-93539-no-rs | 4 | 7 | 1.34% |
| 133 | chr4 | 22,015,576 | 22,095,458 | 79,883 | gain | BFGL-NGS-117041 | BTA-99534-no-rs | 3 | 1 | Unique |
| 134 | chr4 | 23,703,769 | 23,975,480 | 271,712 | loss | BTB-01581687 | Hapmap50909-BTA-17901 | 7 | 1 | Unique |
| 135 | chr4 | 25,321,539 | 25,381,361 | 59,823 | loss | BTA-72695-no-rs | BTB-00169573 | 3 | 2 | Multiple |
| 136 | chr4 | 25,546,708 | 25,743,842 | 197,135 | loss | BTB-01768855 | BTB-01912042 | 6 | 1 | Unique |
| 137 | chr4 | 31,261,759 | 31,394,017 | 132,259 | loss | Hapmap44276-BTA-69937 | Hapmap54229-rs29017613 | 3 | 1 | Unique |
| 138 | chr4 | 38,486,603 | 38,553,348 | 66,746 | loss | Hapmap33483-BTA-142021 | BTB-00175546 | 3 | 1 | Unique |
| 139 | chr4 | 40,597,911 | 40,869,775 | 271,865 | gain | BTA-21017-no-rs | Hapmap50762-BTA-70080 | 8 | 2 | Multiple |
| 140 | chr4 | 43,321,794 | 43,458,502 | 136,709 | both | BTB-00178816 | BTB-00178966 | 6 | 3 | 0.58% |
| 141 | chr4 | 44,193,866 | 44,388,090 | 194,225 | gain | ARS-BFGL-NGS-97154 | BFGL-NGS-116726 | 3 | 1 | Unique |
| 142 | chr4 | 50,394,779 | 50,564,772 | 169,994 | both | Hapmap46158-BTA-70371 | BTB-00181018 | 5 | 2 | Multiple |
| 143 | chr4 | 56,969,263 | 57,121,588 | 152,326 | loss | BTB-01298953 | BTB-00186521 | 5 | 1 | Unique |
| 144 | chr4 | 68,833,565 | 69,130,299 | 296,735 | gain | BTA-13086-rs29018051 | Hapmap57289-ss46526668 | 9 | 2 | Multiple |
| 145 | chr4 | 71,499,521 | 71,617,486 | 117,966 | gain | Hapmap38430-BTA-71131 | ARS-BFGL-NGS-97660 | 5 | 2 | Multiple |
| 146 | chr4 | 75,693,605 | 75,751,872 | 58,268 | loss | BTB-00196932 | Hapmap44399-BTA-71366 | 3 | 6 | 1.15% |
| 147 | chr4 | 83,445,170 | 83,797,597 | 352,428 | loss | BTA-71558-no-rs | BTA-71571-no-rs | 9 | 1 | Unique |
| 148 | chr4 | 85,750,870 | 85,923,634 | 172,765 | both | BTB-02092948 | ARS-BFGL-NGS-5222 | 6 | 14 | 2.69% |
| 149 | chr4 | 86,213,244 | 86,712,105 | 498,862 | loss | BTB-01857387 | BTB-01143040 | 13 | 1 | Unique |
| 150 | chr4 | 87,082,107 | 87,293,611 | 211,505 | loss | BTA-16834-no-rs | BTB-01278734 | 6 | 1 | Unique |
| 151 | chr4 | 87,907,646 | 88,191,209 | 283,564 | gain | UA-IFASA-181 | Hapmap39905-BTA-27381 | 8 | 8 | 1.54% |
| 152 | chr4 | 89,087,907 | 89,201,904 | 113,998 | gain | BTB-01032530 | ARS-BFGL-NGS-33395 | 4 | 1 | Unique |
| 153 | chr4 | 92,193,078 | 92,263,952 | 70,875 | loss | BTB-01759027 | BTA-122943-no-rs | 3 | 2 | Multiple |
| 154 | chr4 | 92,534,110 | 92,781,084 | 246,975 | both | Hapmap25897-BTA-158225 | BTB-01901306 | 8 | 3 | 0.58% |
| 155 | chr4 | 94,870,406 | 94,975,831 | 105,426 | loss | BTB-00202180 | BTB-01295144 | 3 | 1 | Unique |
| 156 | chr4 | 97,492,385 | 97,532,889 | 40,505 | gain | UA-IFASA-7564 | ARS-BFGL-NGS-53072 | 3 | 2 | Multiple |
| 157 | chr4 | 98,375,185 | 98,463,138 | 87,954 | gain | BTB-01298816 | ARS-BFGL-NGS-11916 | 3 | 2 | Multiple |
| 158 | chr4 | 109,607,343 | 109,690,847 | 83,505 | gain | ARS-BFGL-NGS-54107 | BTB-02023900 | 3 | 9 | 1.73% |
| 159 | chr4 | 111,515,378 | 111,659,295 | 143,918 | loss | BTA-72108-no-rs | BTA-72199-no-rs | 4 | 6 | 1.15% |
| 160 | chr4 | 112,133,333 | 112,214,063 | 80,731 | loss | ARS-BFGL-NGS-73118 | BFGL-NGS-119262 | 3 | 1 | Unique |
| 161 | chr4 | 115,605,286 | 115,650,551 | 45,266 | loss | BTB-01163185 | ARS-BFGL-NGS-33889 | 3 | 1 | Unique |
| 162 | chr4 | 117,147,050 | 117,631,720 | 484,671 | gain | ARS-BFGL-NGS-74214 | ARS-BFGL-NGS-1225 | 14 | 86 | 16.51% |
| 163 | chr4 | 122,050,101 | 122,097,244 | 47,144 | gain | Hapmap35657-SCAFFOLD15929_9264 | ARS-BFGL-NGS-105018 | 3 | 1 | Unique |
| 164 | chr5 | 3,563,151 | 3,858,443 | 295,293 | loss | Hapmap44365-BTA-28169 | BTB-00909797 | 10 | 7 | 1.34% |
| 165 | chr5 | 4,046,316 | 4,129,013 | 82,698 | gain | ARS-BFGL-NGS-27281 | Hapmap48402-BTA-60008 | 3 | 1 | Unique |
| 166 | chr5 | 4,627,084 | 4,987,905 | 360,822 | gain | Hapmap39974-BTA-119920 | BTB-01486702 | 12 | 2 | Multiple |
| 167 | chr5 | 6,713,153 | 6,803,754 | 90,602 | gain | Hapmap59616-ss46526976 | ARS-BFGL-NGS-8671 | 3 | 2 | Multiple |
| 168 | chr5 | 9,411,764 | 9,467,780 | 56,017 | loss | BTB-00218987 | BTB-00219084 | 3 | 2 | Multiple |
| 169 | chr5 | 9,713,680 | 10,277,314 | 563,635 | both | BTB-00235875 | ARS-BFGL-NGS-30033 | 11 | 15 | 2.88% |
| 170 | chr5 | 14,666,552 | 14,725,677 | 59,126 | loss | BTA-75576-no-rs | DIAS-102 | 3 | 1 | Unique |
| 171 | chr5 | 16,957,758 | 17,141,183 | 183,426 | loss | BTA-87039-no-rs | Hapmap44154-BTA-87046 | 6 | 2 | Multiple |
| 172 | chr5 | 23,812,682 | 23,942,378 | 129,697 | gain | ARS-BFGL-NGS-7668 | DIAS-17 | 3 | 1 | Unique |
| 173 | chr5 | 33,437,589 | 33,493,535 | 55,947 | loss | Hapmap38536-BTA-85959 | BTA-73280-no-rs | 3 | 2 | Multiple |
| 174 | chr5 | 44,075,126 | 44,232,398 | 157,273 | gain | Hapmap44326-BTA-97746 | ARS-BFGL-NGS-19252 | 6 | 4 | 0.77% |
| 175 | chr5 | 45,904,554 | 45,966,700 | 62,147 | loss | BTA-119080-no-rs | BTB-01255957 | 3 | 4 | 0.77% |
| 176 | chr5 | 46,996,517 | 47,059,557 | 63,041 | loss | BTB-00227037 | Hapmap51299-BTA-73473 | 3 | 2 | Multiple |
| 177 | chr5 | 60,409,033 | 60,570,822 | 161,790 | both | ARS-BFGL-NGS-100975 | Hapmap44616-BTA-73618 | 3 | 5 | 0.96% |
| 178 | chr5 | 63,031,140 | 63,605,941 | 574,802 | both | BTA-87618-no-rs | BTB-02067717 | 10 | 114 | 21.88% |
| 179 | chr5 | 64,141,257 | 64,253,582 | 112,326 | gain | ARS-BFGL-NGS-44413 | BTB-01636697 | 4 | 3 | 0.58% |
| 180 | chr5 | 68,912,199 | 69,020,840 | 108,642 | loss | Hapmap51479-BTA-66720 | BTA-66714-no-rs | 3 | 2 | Multiple |
| 181 | chr5 | 81,874,749 | 82,665,617 | 790,869 | loss | Hapmap24162-BTA-160429 | BTB-00231979 | 21 | 4 | 0.77% |
| 182 | chr5 | 83,776,222 | 84,068,558 | 292,337 | both | Hapmap52101-rs29018073 | ARS-BFGL-NGS-99969 | 5 | 2 | Multiple |
| 183 | chr5 | 93,046,854 | 93,153,750 | 106,897 | loss | BTA-98442-no-rs | ARS-BFGL-NGS-73390 | 4 | 1 | Unique |
| 184 | chr5 | 96,448,995 | 96,517,829 | 68,835 | gain | Hapmap42271-BTA-61859 | ARS-BFGL-NGS-58101 | 3 | 2 | Multiple |
| 185 | chr5 | 106,451,095 | 106,646,138 | 195,044 | loss | ARS-BFGL-NGS-108996 | BTB-02009804 | 3 | 1 | Unique |
| 186 | chr5 | 109,235,853 | 110,191,418 | 955,566 | both | Hapmap3063-BTA-15439 | Hapmap40294-BTA-89295 | 12 | 116 | 22.26% |
| 187 | chr5 | 115,808,996 | 115,919,741 | 110,746 | loss | ARS-BFGL-NGS-85264 | ARS-BFGL-NGS-85311 | 4 | 1 | Unique |
| 188 | chr5 | 119,128,022 | 119,223,666 | 95,645 | loss | ARS-BFGL-NGS-10616 | ARS-BFGL-NGS-102035 | 4 | 1 | Unique |
| 189 | chr5 | 120,238,291 | 120,362,864 | 124,574 | loss | BFGL-NGS-119376 | ARS-BFGL-NGS-31332 | 3 | 2 | Multiple |
| 190 | chr5 | 121,502,580 | 121,557,022 | 54,443 | gain | ARS-BFGL-NGS-38686 | ARS-BFGL-NGS-100195 | 3 | 1 | Unique |
| 191 | chr5 | 122,148,990 | 122,208,842 | 59,853 | loss | ARS-BFGL-NGS-11640 | ARS-BFGL-NGS-64268 | 3 | 2 | Multiple |
| 192 | chr5 | 123,156,376 | 123,840,785 | 684,410 | both | BFGL-NGS-116103 | ARS-BFGL-NGS-1089 | 22 | 5 | 0.96% |
| 193 | chr5 | 125,036,256 | 125,147,296 | 111,041 | loss | Hapmap42757-BTA-99810 | ARS-BFGL-NGS-13216 | 5 | 1 | Unique |
| 194 | chr6 | 43,581 | 883,890 | 840,310 | both | BTB-01518631 | BTB-01796690 | 24 | 23 | 4.41% |
| 195 | chr6 | 1,374,013 | 1,452,846 | 78,834 | loss | BTB-01461517 | BTB-01744782 | 3 | 2 | Multiple |
| 196 | chr6 | 3,215,714 | 3,372,536 | 156,823 | loss | Hapmap53408-rs29014449 | BTB-00241116 | 3 | 1 | Unique |
| 197 | chr6 | 6,757,367 | 6,845,580 | 88,214 | both | BTB-01596025 | Hapmap29564-BTA-143742 | 4 | 2 | Multiple |
| 198 | chr6 | 8,235,476 | 8,351,663 | 116,188 | loss | Hapmap50460-BTA-75791 | BTA-75766-no-rs | 3 | 1 | Unique |
| 199 | chr6 | 8,658,350 | 9,046,434 | 388,085 | loss | BTB-02087354 | BTB-01704614 | 10 | 4 | 0.77% |
| 200 | chr6 | 9,230,391 | 9,337,873 | 107,483 | loss | BTB-01068781 | BTA-104961-no-rs | 4 | 1 | Unique |
| 201 | chr6 | 9,612,713 | 10,206,655 | 593,943 | both | Hapmap33379-BTA-153882 | BTB-02016906 | 10 | 10 | 1.92% |
| 202 | chr6 | 10,495,988 | 11,000,273 | 504,286 | both | BTA-22613-no-rs | Hapmap58659-rs29011609 | 15 | 5 | 0.96% |
| 203 | chr6 | 11,405,908 | 11,648,469 | 242,562 | loss | ARS-BFGL-NGS-38131 | Hapmap40034-BTA-93974 | 7 | 1 | Unique |
| 204 | chr6 | 12,191,817 | 12,295,124 | 103,308 | loss | BTB-01685239 | Hapmap23501-BTA-155913 | 4 | 1 | Unique |
| 205 | chr6 | 12,358,731 | 12,472,476 | 113,746 | gain | BTA-112689-no-rs | BTA-88018-no-rs | 4 | 3 | 0.58% |
| 206 | chr6 | 17,769,611 | 17,853,627 | 84,017 | loss | BTB-00247622 | BTA-77526-no-rs | 5 | 1 | Unique |
| 207 | chr6 | 31,694,807 | 31,807,491 | 112,685 | loss | ARS-BFGL-NGS-11498 | Hapmap49740-BTA-75691 | 4 | 1 | Unique |
| 208 | chr6 | 35,462,920 | 35,690,337 | 227,418 | loss | Hapmap60855-rs29024248 | Hapmap43447-BTA-105138 | 3 | 2 | Multiple |
| 209 | chr6 | 38,233,089 | 38,326,148 | 93,060 | loss | Hapmap23507-BTC-041133 | Hapmap33628-BTC-041023 | 4 | 2 | Multiple |
| 210 | chr6 | 41,242,588 | 41,323,841 | 81,254 | loss | BTB-00252870 | Hapmap24317-BTC-037593 | 4 | 1 | Unique |
| 211 | chr6 | 42,786,668 | 42,923,659 | 136,992 | loss | BTA-95818-no-rs | BTB-01468543 | 5 | 10 | 1.92% |
| 212 | chr6 | 47,058,970 | 47,210,710 | 151,741 | gain | BTA-76119-no-rs | Hapmap33922-BES7_Contig380_533 | 3 | 2 | Multiple |
| 213 | chr6 | 47,771,417 | 48,044,634 | 273,218 | gain | BTA-04576-rs29014935 | ARS-BFGL-NGS-101567 | 7 | 1 | Unique |
| 214 | chr6 | 48,367,512 | 48,639,805 | 272,294 | loss | Hapmap44495-BTA-91063 | ARS-BFGL-NGS-41037 | 7 | 4 | 0.77% |
| 215 | chr6 | 48,928,335 | 49,042,264 | 113,930 | loss | BTB-01688071 | BTB-01527594 | 4 | 1 | Unique |
| 216 | chr6 | 52,438,316 | 52,640,931 | 202,616 | loss | Hapmap23184-BTC-039909 | ARS-BFGL-NGS-35824 | 8 | 3 | 0.58% |
| 217 | chr6 | 53,329,684 | 53,747,773 | 418,090 | loss | Hapmap31996-BTC-066011 | BTB-00257742 | 10 | 9 | 1.73% |
| 218 | chr6 | 56,960,794 | 57,233,856 | 273,063 | loss | BTB-00258367 | BTB-01453811 | 9 | 2 | Multiple |
| 219 | chr6 | 57,548,761 | 57,612,503 | 63,743 | loss | Hapmap25928-BTA-18390 | BFGL-NGS-114099 | 3 | 2 | Multiple |
| 220 | chr6 | 63,706,460 | 63,850,642 | 144,183 | loss | ARS-BFGL-NGS-35835 | ARS-BFGL-NGS-109055 | 3 | 3 | 0.58% |
| 221 | chr6 | 71,356,946 | 71,526,956 | 170,011 | loss | Hapmap44512-BTA-107928 | BTA-76705-no-rs | 5 | 2 | Multiple |
| 222 | chr6 | 72,784,128 | 73,035,436 | 251,309 | gain | Hapmap31616-BTC-042811 | Hapmap29128-BTC-042274 | 7 | 1 | Unique |
| 223 | chr6 | 78,554,840 | 78,780,301 | 225,462 | loss | BTA-28969-no-rs | BTA-114800-no-rs | 6 | 4 | 0.77% |
| 224 | chr6 | 81,354,371 | 81,808,192 | 453,822 | both | ARS-BFGL-NGS-108577 | BTB-01578666 | 10 | 9 | 1.73% |
| 225 | chr6 | 82,017,155 | 82,278,023 | 260,869 | loss | BTB-01801188 | Hapmap22939-BTA-149533 | 5 | 1 | Unique |
| 226 | chr6 | 82,579,209 | 82,641,003 | 61,795 | loss | BTB-00264815 | Hapmap51938-BTA-21491 | 3 | 1 | Unique |
| 227 | chr6 | 83,023,197 | 83,106,835 | 83,639 | loss | BTA-20903-no-rs | Hapmap27224-BTA-161106 | 3 | 2 | Multiple |
| 228 | chr6 | 84,894,568 | 85,083,241 | 188,674 | loss | BTA-76960-no-rs | Hapmap43417-BTA-96760 | 7 | 2 | Multiple |
| 229 | chr6 | 88,831,001 | 88,983,536 | 152,536 | gain | ARS-BFGL-NGS-83385 | Hapmap33210-BTA-144234 | 3 | 1 | Unique |
| 230 | chr6 | 90,737,718 | 90,803,037 | 65,320 | loss | BTB-01946648 | BTA-77205-no-rs | 3 | 2 | Multiple |
| 231 | chr6 | 93,417,007 | 93,463,203 | 46,197 | gain | BTB-01496144 | BTB-01496160 | 3 | 3 | 0.58% |
| 232 | chr6 | 97,693,438 | 99,298,384 | 1,604,947 | gain | BTB-00272142 | BTB-00272812 | 29 | 2 | Multiple |
| 233 | chr6 | 103,381,990 | 103,467,953 | 85,964 | gain | Hapmap39434-BTA-77640 | BTA-26167-no-rs | 3 | 1 | Unique |
| 234 | chr6 | 104,117,721 | 104,161,908 | 44,188 | loss | Hapmap23305-BTC-071388 | Hapmap47766-BTA-87827 | 3 | 1 | Unique |
| 235 | chr6 | 104,503,905 | 104,567,927 | 64,023 | loss | Hapmap26858-BTC-044514 | BFGL-NGS-117296 | 3 | 2 | Multiple |
| 236 | chr6 | 118,232,279 | 119,068,909 | 836,631 | both | ARS-BFGL-NGS-43597 | BFGL-NGS-110156 | 20 | 6 | 1.15% |
| 237 | chr6 | 119,163,113 | 119,241,601 | 78,489 | gain | ARS-BFGL-NGS-41054 | ARS-BFGL-NGS-39838 | 4 | 1 | Unique |
| 238 | chr6 | 119,339,223 | 119,771,391 | 432,169 | loss | ARS-BFGL-NGS-56487 | BTA-09585-no-rs | 14 | 1 | Unique |
| 239 | chr6 | 120,303,386 | 120,450,993 | 147,608 | both | ARS-BFGL-NGS-10182 | ARS-BFGL-NGS-17086 | 5 | 3 | 0.58% |
| 240 | chr7 | 1,343,221 | 1,493,916 | 150,696 | both | ARS-BFGL-NGS-44014 | ARS-BFGL-NGS-62046 | 6 | 10 | 1.92% |
| 241 | chr7 | 7,784,351 | 8,110,307 | 325,957 | both | Hapmap33677-BTA-159272 | ARS-BFGL-NGS-20506 | 9 | 23 | 4.41% |
| 242 | chr7 | 14,635,618 | 14,721,613 | 85,996 | gain | ARS-BFGL-NGS-4774 | ARS-BFGL-NGS-35463 | 3 | 1 | Unique |
| 243 | chr7 | 22,339,658 | 22,400,852 | 61,195 | loss | BTA-78589-no-rs | Hapmap51318-BTA-78584 | 3 | 4 | 0.77% |
| 244 | chr7 | 32,121,704 | 32,195,122 | 73,419 | loss | BTB-01115116 | BTB-01947935 | 4 | 1 | Unique |
| 245 | chr7 | 40,664,184 | 41,353,074 | 688,891 | both | BTB-00307908 | BTB-02092796 | 19 | 35 | 6.72% |
| 246 | chr7 | 42,506,811 | 42,816,140 | 309,330 | loss | ARS-BFGL-NGS-11022 | BFGL-NGS-109750 | 9 | 1 | Unique |
| 247 | chr7 | 47,858,364 | 47,930,714 | 72,351 | gain | ARS-BFGL-NGS-57250 | BFGL-NGS-116486 | 3 | 1 | Unique |
| 248 | chr7 | 53,858,216 | 53,907,028 | 48,813 | loss | Hapmap52400-rs29025316 | BFGL-NGS-119198 | 3 | 1 | Unique |
| 249 | chr7 | 62,197,017 | 62,245,328 | 48,312 | loss | DIAS-106 | DIAS-107 | 3 | 1 | Unique |
| 250 | chr7 | 62,273,994 | 62,486,891 | 212,898 | gain | ARS-BFGL-NGS-81508 | Hapmap48570-BTA-104179 | 3 | 2 | Multiple |
| 251 | chr7 | 70,517,489 | 70,636,975 | 119,487 | loss | Hapmap26235-BTA-79759 | BTB-00319090 | 5 | 1 | Unique |
| 252 | chr7 | 76,167,617 | 76,437,168 | 269,552 | both | Hapmap53801-rs29012591 | Hapmap33614-BTA-79859 | 6 | 4 | 0.77% |
| 253 | chr7 | 76,490,016 | 77,340,597 | 850,582 | both | BTA-98898-no-rs | BTA-98858-no-rs | 17 | 44 | 8.45% |
| 254 | chr7 | 85,242,291 | 85,311,447 | 69,157 | loss | ARS-BFGL-NGS-92532 | ARS-BFGL-NGS-17425 | 3 | 2 | Multiple |
| 255 | chr7 | 85,904,613 | 86,060,867 | 156,255 | loss | ARS-BFGL-NGS-70648 | BTB-01388280 | 5 | 1 | Unique |
| 256 | chr7 | 86,376,789 | 87,203,186 | 826,398 | both | Hapmap44668-BTA-119022 | BTB-00325285 | 23 | 8 | 1.54% |
| 257 | chr7 | 96,959,792 | 97,148,954 | 189,163 | loss | Hapmap36470-SCAFFOLD25306_18005 | BFGL-NGS-119112 | 5 | 2 | Multiple |
| 258 | chr7 | 103,071,120 | 103,135,628 | 64,509 | gain | BTA-80440-no-rs | ARS-BFGL-NGS-94392 | 3 | 1 | Unique |
| 259 | chr7 | 104,003,205 | 104,139,089 | 135,885 | gain | BTB-00329283 | ARS-BFGL-NGS-5197 | 5 | 1 | Unique |
| 260 | chr7 | 111,069,469 | 111,352,040 | 282,572 | loss | ARS-BFGL-NGS-84234 | Hapmap58496-rs29017214 | 11 | 1 | Unique |
| 261 | chr8 | 4,139,375 | 4,555,303 | 415,929 | loss | BTA-86030-no-rs | BTA-92628-no-rs | 9 | 1 | Unique |
| 262 | chr8 | 13,181,581 | 13,303,341 | 121,761 | loss | ARS-BFGL-NGS-69683 | Hapmap42694-BTA-82927 | 4 | 2 | Multiple |
| 263 | chr8 | 13,562,781 | 13,842,047 | 279,267 | loss | Hapmap36058-SCAFFOLD313402_3070 | BTA-82931-no-rs | 5 | 1 | Unique |
| 264 | chr8 | 14,891,603 | 15,041,047 | 149,445 | loss | BTA-16324-no-rs | BTB-00336089 | 4 | 5 | 0.96% |
| 265 | chr8 | 19,459,909 | 19,519,579 | 59,671 | gain | ARS-BFGL-NGS-36682 | BTB-01099280 | 3 | 1 | Unique |
| 266 | chr8 | 23,551,032 | 23,657,920 | 106,889 | gain | Hapmap50207-BTA-108741 | BTB-01944005 | 4 | 9 | 1.73% |
| 267 | chr8 | 24,330,763 | 24,397,208 | 66,446 | gain | BTB-01833512 | ARS-BFGL-NGS-106633 | 3 | 3 | 0.58% |
| 268 | chr8 | 27,001,090 | 27,103,092 | 102,003 | loss | BFGL-NGS-110838 | Hapmap22995-BTA-120691 | 3 | 1 | Unique |
| 269 | chr8 | 27,391,620 | 27,816,984 | 425,365 | loss | Hapmap49881-BTA-120681 | BTB-01108991 | 11 | 2 | Multiple |
| 270 | chr8 | 34,020,944 | 34,694,187 | 673,244 | both | Hapmap55761-rs29014147 | ARS-BFGL-NGS-5314 | 14 | 8 | 1.54% |
| 271 | chr8 | 36,650,818 | 36,779,293 | 128,476 | loss | Hapmap33684-BTA-27870 | Hapmap53908-rs29021644 | 6 | 1 | Unique |
| 272 | chr8 | 39,075,904 | 39,184,274 | 108,371 | gain | BTB-00340836 | BTB-00675470 | 4 | 8 | 1.54% |
| 273 | chr8 | 45,414,798 | 45,693,223 | 278,426 | loss | BTA-118168-no-rs | Hapmap40627-BTA-100509 | 4 | 3 | 0.58% |
| 274 | chr8 | 58,683,837 | 58,779,630 | 95,794 | loss | BTA-120434-no-rs | Hapmap42859-BTA-17125 | 3 | 2 | Multiple |
| 275 | chr8 | 61,139,487 | 61,207,443 | 67,957 | loss | BTB-00350639 | Hapmap42118-BTA-16761 | 3 | 2 | Multiple |
| 276 | chr8 | 62,727,602 | 63,021,880 | 294,279 | gain | Hapmap39976-BTA-121843 | BTA-94058-no-rs | 4 | 1 | Unique |
| 277 | chr8 | 68,112,627 | 68,174,408 | 61,782 | gain | BTA-62442-no-rs | BTB-01657439 | 3 | 1 | Unique |
| 278 | chr8 | 68,525,502 | 68,680,839 | 155,338 | gain | BTB-01952185 | BFGL-NGS-110360 | 6 | 13 | 2.50% |
| 279 | chr8 | 73,267,223 | 73,976,064 | 708,842 | both | BTB-01468721 | BFGL-NGS-119743 | 10 | 10 | 1.92% |
| 280 | chr8 | 88,677,969 | 88,842,974 | 165,006 | both | ARS-BFGL-NGS-63666 | ARS-BFGL-NGS-104204 | 5 | 9 | 1.73% |
| 281 | chr8 | 91,326,289 | 91,594,769 | 268,481 | gain | Hapmap49329-BTA-82142 | BTB-00365092 | 5 | 1 | Unique |
| 282 | chr8 | 93,841,920 | 93,945,194 | 103,275 | gain | ARS-BFGL-NGS-102412 | Hapmap33490-BTA-146089 | 4 | 1 | Unique |
| 283 | chr8 | 94,670,030 | 94,712,098 | 42,069 | gain | ARS-BFGL-NGS-41178 | BTA-23643-no-rs | 3 | 1 | Unique |
| 284 | chr8 | 96,931,174 | 97,028,748 | 97,575 | loss | BTB-01363589 | BTB-01537249 | 3 | 2 | Multiple |
| 285 | chr8 | 97,267,682 | 97,475,630 | 207,949 | loss | BTB-00367725 | BTB-00368101 | 7 | 1 | Unique |
| 286 | chr8 | 99,823,699 | 99,961,134 | 137,436 | loss | Hapmap47520-BTA-117879 | Hapmap51091-BTA-92938 | 4 | 2 | Multiple |
| 287 | chr8 | 104,804,277 | 104,885,680 | 81,404 | both | BTB-00370321 | Hapmap58644-rs29010529 | 3 | 3 | 0.58% |
| 288 | chr8 | 104,929,794 | 104,993,059 | 63,266 | loss | BTB-00371041 | ARS-BFGL-NGS-74165 | 3 | 2 | Multiple |
| 289 | chr9 | 1,203,864 | 1,463,199 | 259,336 | loss | BTB-01752812 | BTB-01837292 | 6 | 1 | Unique |
| 290 | chr9 | 2,798,929 | 3,339,189 | 540,261 | loss | ARS-BFGL-NGS-12335 | BTA-83392-no-rs | 7 | 1 | Unique |
| 291 | chr9 | 3,546,077 | 3,628,463 | 82,387 | loss | ARS-BFGL-NGS-20003 | Hapmap48549-BTA-99373 | 3 | 2 | Multiple |
| 292 | chr9 | 3,681,332 | 3,758,052 | 76,721 | loss | ARS-BFGL-NGS-42521 | Hapmap42378-BTA-97334 | 3 | 1 | Unique |
| 293 | chr9 | 4,949,585 | 5,214,444 | 264,860 | loss | Hapmap31176-BTA-158553 | BTB-01428058 | 6 | 3 | 0.58% |
| 294 | chr9 | 12,286,794 | 12,430,446 | 143,653 | loss | ARS-BFGL-NGS-26493 | ARS-BFGL-NGS-42000 | 3 | 1 | Unique |
| 295 | chr9 | 14,893,068 | 15,046,354 | 153,287 | gain | Hapmap27634-BTA-158717 | Hapmap54560-rs29009707 | 7 | 9 | 1.73% |
| 296 | chr9 | 15,096,593 | 15,232,993 | 136,401 | loss | BTA-84936-no-rs | Hapmap44930-BTA-109958 | 5 | 3 | 0.58% |
| 297 | chr9 | 16,215,898 | 16,512,390 | 296,493 | gain | ARS-BFGL-NGS-70421 | BTB-01556111 | 8 | 1 | Unique |
| 298 | chr9 | 18,477,636 | 18,550,265 | 72,630 | loss | Hapmap46542-BTA-94412 | BTA-94407-no-rs | 3 | 1 | Unique |
| 299 | chr9 | 24,569,246 | 24,700,522 | 131,277 | both | BTB-01362150 | Hapmap42168-BTA-28710 | 5 | 3 | 0.58% |
| 300 | chr9 | 25,805,685 | 25,863,098 | 57,414 | loss | BTA-83093-no-rs | BTB-02020793 | 3 | 2 | Multiple |
| 301 | chr9 | 54,176,473 | 54,247,544 | 71,072 | loss | Hapmap23928-BTA-102869 | ARS-BFGL-NGS-104966 | 3 | 2 | Multiple |
| 302 | chr9 | 55,877,624 | 56,055,016 | 177,393 | gain | Hapmap41423-BTA-109716 | Hapmap44208-BTA-119972 | 5 | 3 | 0.58% |
| 303 | chr9 | 57,618,813 | 57,796,887 | 178,075 | loss | BTB-02010143 | BTB-01716755 | 6 | 2 | Multiple |
| 304 | chr9 | 59,637,570 | 59,823,512 | 185,943 | gain | BTA-92472-no-rs | BTB-01573139 | 7 | 1 | Unique |
| 305 | chr9 | 61,426,424 | 61,529,985 | 103,562 | loss | BTB-00395419 | Hapmap51401-BTA-117495 | 4 | 1 | Unique |
| 306 | chr9 | 80,970,124 | 81,277,653 | 307,530 | loss | Hapmap40091-BTA-91850 | Hapmap43371-BTA-84377 | 5 | 1 | Unique |
| 307 | chr9 | 87,633,214 | 87,790,636 | 157,423 | gain | BTA-84561-no-rs | BTB-01637411 | 5 | 1 | Unique |
| 308 | chr9 | 89,952,780 | 90,070,187 | 117,408 | gain | BTA-08180-no-rs | BTA-01364-no-rs | 5 | 6 | 1.15% |
| 309 | chr9 | 90,226,974 | 90,459,834 | 232,861 | both | ARS-BFGL-NGS-16116 | BFGL-NGS-110042 | 6 | 10 | 1.92% |
| 310 | chr9 | 94,364,803 | 94,446,464 | 81,662 | loss | Hapmap59634-rs29013969 | BTB-01586455 | 4 | 3 | 0.58% |
| 311 | chr9 | 94,703,203 | 94,766,825 | 63,623 | loss | Hapmap43080-BTA-84772 | ARS-BFGL-NGS-5159 | 3 | 1 | Unique |
| 312 | chr9 | 95,783,554 | 95,863,973 | 80,420 | loss | ARS-BFGL-NGS-52931 | BFGL-NGS-112933 | 3 | 2 | Multiple |
| 313 | chr9 | 101,300,976 | 101,364,396 | 63,421 | loss | ARS-BFGL-NGS-83787 | Hapmap43375-BTA-85005 | 3 | 2 | Multiple |
| 314 | chr9 | 104,333,966 | 104,462,688 | 128,723 | gain | Hapmap48173-BTA-107833 | Hapmap38266-BTA-97562 | 4 | 1 | Unique |
| 315 | chr9 | 105,067,571 | 105,172,904 | 105,334 | loss | ARS-BFGL-NGS-26554 | BFGL-NGS-117605 | 3 | 1 | Unique |
| 316 | chr9 | 106,108,336 | 106,157,975 | 49,640 | gain | Hapmap33058-BTA-158159 | ARS-BFGL-NGS-41284 | 3 | 1 | Unique |
| 317 | chr10 | 14,028,462 | 14,186,894 | 158,433 | loss | BTB-00410198 | Hapmap51496-BTA-79294 | 6 | 1 | Unique |
| 318 | chr10 | 22,672,607 | 22,831,437 | 158,831 | loss | ARS-BFGL-NGS-100895 | BFGL-NGS-112477 | 4 | 11 | 2.11% |
| 319 | chr10 | 22,991,034 | 24,808,294 | 1,817,261 | both | BTB-02001051 | BTB-01806171 | 28 | 115 | 22.07% |
| 320 | chr10 | 26,890,931 | 26,981,431 | 90,501 | loss | ARS-BFGL-NGS-94999 | BTB-02040954 | 3 | 3 | 0.58% |
| 321 | chr10 | 32,453,851 | 32,544,761 | 90,911 | gain | Hapmap50425-BTA-63280 | Hapmap44110-BTA-63281 | 3 | 2 | Multiple |
| 322 | chr10 | 34,965,552 | 35,069,086 | 103,535 | loss | ARS-BFGL-NGS-43296 | Hapmap54460-rs29016462 | 4 | 1 | Unique |
| 323 | chr10 | 38,157,709 | 38,292,369 | 134,661 | gain | BTA-66227-no-rs | ARS-BFGL-NGS-102016 | 4 | 1 | Unique |
| 324 | chr10 | 39,780,856 | 39,953,528 | 172,673 | loss | BTA-66276-no-rs | BTB-00418936 | 5 | 1 | Unique |
| 325 | chr10 | 41,373,532 | 41,478,087 | 104,556 | both | BTB-01400850 | BTB-01482386 | 3 | 5 | 0.96% |
| 326 | chr10 | 44,022,138 | 44,093,124 | 70,987 | loss | Hapmap28049-BTA-148967 | Hapmap55224-rs29012840 | 5 | 1 | Unique |
| 327 | chr10 | 51,875,050 | 51,963,015 | 87,966 | loss | BTB-00992193 | BTB-00426535 | 4 | 2 | Multiple |
| 328 | chr10 | 54,714,669 | 54,760,455 | 45,787 | loss | BTA-87361-no-rs | BTB-01137887 | 3 | 2 | Multiple |
| 329 | chr10 | 60,949,189 | 61,165,187 | 215,999 | loss | ARS-BFGL-NGS-87984 | BTA-66849-no-rs | 7 | 3 | 0.58% |
| 330 | chr10 | 71,340,536 | 71,442,939 | 102,404 | loss | ARS-BFGL-NGS-105887 | BTB-00433829 | 4 | 1 | Unique |
| 331 | chr10 | 72,560,546 | 72,618,675 | 58,130 | loss | Hapmap44482-BTA-74309 | BTB-00434592 | 3 | 1 | Unique |
| 332 | chr10 | 73,839,536 | 73,935,992 | 96,457 | loss | BTB-01693217 | Hapmap27480-BTA-75244 | 3 | 3 | 0.58% |
| 333 | chr10 | 78,817,442 | 78,918,396 | 100,955 | loss | Hapmap44484-BTA-75324 | ARS-BFGL-NGS-38839 | 3 | 1 | Unique |
| 334 | chr10 | 80,696,497 | 80,795,997 | 99,501 | gain | ARS-BFGL-NGS-55923 | BTA-76281-no-rs | 3 | 2 | Multiple |
| 335 | chr10 | 83,642,486 | 83,707,371 | 64,886 | gain | BTB-00438561 | ARS-BFGL-NGS-18421 | 3 | 1 | Unique |
| 336 | chr10 | 87,481,635 | 87,724,129 | 242,495 | loss | BTB-02091381 | ARS-BFGL-BAC-11028 | 6 | 1 | Unique |
| 337 | chr10 | 91,503,333 | 91,639,299 | 135,967 | gain | BTB-00442692 | ARS-BFGL-NGS-14139 | 6 | 7 | 1.34% |
| 338 | chr10 | 96,556,741 | 96,615,878 | 59,138 | loss | ARS-BFGL-NGS-21459 | ARS-BFGL-NGS-5768 | 3 | 2 | Multiple |
| 339 | chr10 | 96,659,357 | 96,741,162 | 81,806 | loss | BTB-01311442 | ARS-BFGL-NGS-102179 | 3 | 2 | Multiple |
| 340 | chr11 | 20,576 | 162,330 | 141,755 | gain | BTA-17117-no-rs | BTB-01593958 | 3 | 2 | Multiple |
| 341 | chr11 | 444,147 | 588,638 | 144,492 | loss | Hapmap26105-BTA-126070 | ARS-BFGL-BAC-14260 | 4 | 2 | Multiple |
| 342 | chr11 | 4,201,594 | 4,318,706 | 117,113 | loss | ARS-BFGL-NGS-18450 | ARS-BFGL-NGS-105764 | 4 | 5 | 0.96% |
| 343 | chr11 | 20,611,027 | 20,701,937 | 90,911 | loss | ARS-BFGL-NGS-102490 | BTB-00467628 | 3 | 2 | Multiple |
| 344 | chr11 | 40,028,208 | 40,126,730 | 98,523 | loss | Hapmap51861-BTA-86131 | Hapmap24189-BTA-123161 | 3 | 1 | Unique |
| 345 | chr11 | 41,312,831 | 41,486,028 | 173,198 | both | Hapmap32339-BTA-149195 | Hapmap27268-BTA-91439 | 6 | 3 | 0.58% |
| 346 | chr11 | 48,958,155 | 49,094,687 | 136,533 | loss | BTA-100645-no-rs | Hapmap59563-rs29017556 | 4 | 1 | Unique |
| 347 | chr11 | 49,263,223 | 49,317,563 | 54,341 | loss | BTA-97080-no-rs | ARS-BFGL-NGS-7012 | 3 | 1 | Unique |
| 348 | chr11 | 59,350,663 | 59,498,037 | 147,375 | loss | Hapmap60711-rs29014674 | BTB-01328920 | 3 | 1 | Unique |
| 349 | chr11 | 61,298,720 | 61,706,880 | 408,161 | loss | BTB-01756713 | BTB-01475120 | 6 | 2 | Multiple |
| 350 | chr11 | 71,998,049 | 72,419,793 | 421,745 | loss | BTA-28852-no-rs | ARS-BFGL-NGS-28978 | 12 | 1 | Unique |
| 351 | chr11 | 86,540,548 | 86,608,059 | 67,512 | gain | Hapmap52546-rs29026760 | Hapmap34777-BES11_Contig379_951 | 3 | 1 | Unique |
| 352 | chr11 | 90,588,405 | 90,730,972 | 142,568 | gain | BFGL-NGS-116951 | BFGL-NGS-114352 | 5 | 1 | Unique |
| 353 | chr11 | 94,925,578 | 95,382,284 | 456,707 | gain | BTA-109943-no-rs | Hapmap35103-BES3_Contig455_1055 | 11 | 8 | 1.54% |
| 354 | chr11 | 96,616,167 | 96,707,760 | 91,594 | gain | BTB-02006984 | ARS-BFGL-NGS-1846 | 4 | 5 | 0.96% |
| 355 | chr11 | 107,594,125 | 108,119,253 | 525,129 | both | ARS-BFGL-NGS-40995 | BFGL-NGS-116393 | 15 | 4 | 0.77% |
| 356 | chr11 | 108,506,371 | 108,579,842 | 73,472 | loss | BFGL-NGS-111682 | ARS-BFGL-NGS-14919 | 4 | 2 | Multiple |
| 357 | chr11 | 109,101,259 | 109,346,319 | 245,061 | both | Hapmap42853-BTA-15475 | ARS-BFGL-NGS-285 | 4 | 3 | 0.58% |
| 358 | chr11 | 109,373,092 | 109,607,717 | 234,626 | both | BFGL-NGS-114359 | ARS-BFGL-NGS-25833 | 7 | 11 | 2.11% |
| 359 | chr12 | 2,629,644 | 2,734,960 | 105,317 | loss | BTB-01713684 | BFGL-NGS-111874 | 5 | 2 | Multiple |
| 360 | chr12 | 3,581,207 | 3,779,257 | 198,051 | loss | ARS-BFGL-NGS-65811 | ARS-BFGL-NGS-10688 | 4 | 6 | 1.15% |
| 361 | chr12 | 5,665,748 | 5,856,028 | 190,281 | loss | ARS-BFGL-BAC-775 | BTA-96244-no-rs | 5 | 1 | Unique |
| 362 | chr12 | 7,596,510 | 7,739,106 | 142,597 | gain | Hapmap38662-BTA-22753 | BTB-00486292 | 5 | 1 | Unique |
| 363 | chr12 | 8,140,873 | 8,199,278 | 58,406 | gain | ARS-BFGL-BAC-7358 | ARS-BFGL-NGS-51579 | 3 | 1 | Unique |
| 364 | chr12 | 19,469,000 | 20,145,604 | 676,605 | loss | ARS-BFGL-NGS-21160 | ARS-BFGL-NGS-107895 | 9 | 4 | 0.77% |
| 365 | chr12 | 25,629,699 | 26,132,031 | 502,333 | loss | Hapmap26763-BTA-161285 | Hapmap24457-BTA-153392 | 9 | 2 | Multiple |
| 366 | chr12 | 31,265,332 | 31,582,307 | 316,976 | loss | ARS-BFGL-BAC-15023 | Hapmap33402-BTA-18602 | 7 | 19 | 3.65% |
| 367 | chr12 | 38,564,180 | 38,822,417 | 258,238 | loss | BTA-90709-no-rs | Hapmap49412-BTA-102739 | 6 | 1 | Unique |
| 368 | chr12 | 39,952,770 | 40,118,357 | 165,588 | both | ARS-BFGL-BAC-16276 | ARS-BFGL-BAC-16277 | 4 | 2 | Multiple |
| 369 | chr12 | 44,796,637 | 44,928,813 | 132,177 | loss | BTA-21671-no-rs | Hapmap43513-BTA-21673 | 5 | 2 | Multiple |
| 370 | chr12 | 45,149,025 | 45,202,694 | 53,670 | loss | BTB-01438340 | BTB-01438513 | 3 | 1 | Unique |
| 371 | chr12 | 45,562,272 | 45,730,829 | 168,558 | both | Hapmap44038-BTA-22707 | BTA-22699-no-rs | 6 | 9 | 1.73% |
| 372 | chr12 | 52,666,275 | 52,857,435 | 191,161 | loss | BTB-00493094 | Hapmap50625-BTA-23801 | 7 | 1 | Unique |
| 373 | chr12 | 57,219,963 | 57,946,286 | 726,324 | both | BTB-02035517 | Hapmap33957-BES4_Contig323_1373 | 21 | 10 | 1.92% |
| 374 | chr12 | 58,312,168 | 58,417,583 | 105,416 | loss | BFGL-NGS-113128 | BTB-00266404 | 4 | 3 | 0.58% |
| 375 | chr12 | 59,169,680 | 59,355,654 | 185,975 | loss | BFGL-NGS-109722 | Hapmap23461-BTA-147998 | 6 | 2 | Multiple |
| 376 | chr12 | 62,425,925 | 62,790,151 | 364,227 | both | Hapmap32843-BTA-151916 | BTB-01713461 | 11 | 15 | 2.88% |
| 377 | chr12 | 63,440,478 | 63,891,205 | 450,728 | loss | Hapmap51577-BTA-27003 | BTB-01733812 | 8 | 2 | Multiple |
| 378 | chr12 | 65,796,602 | 66,025,095 | 228,494 | loss | ARS-BFGL-NGS-90411 | BTA-85556-no-rs | 7 | 2 | Multiple |
| 379 | chr12 | 66,854,999 | 66,907,342 | 52,344 | gain | Hapmap48110-BTA-88422 | BTB-00501374 | 3 | 1 | Unique |
| 380 | chr12 | 69,335,922 | 69,599,639 | 263,718 | both | BTB-01992495 | BTA-95991-no-rs | 7 | 38 | 7.29% |
| 381 | chr12 | 69,736,018 | 70,511,189 | 775,172 | both | BFGL-NGS-111261 | ARS-BFGL-NGS-77009 | 21 | 240 | 46.07% |
| 382 | chr12 | 71,063,712 | 71,135,619 | 71,908 | loss | ARS-BFGL-NGS-57541 | Hapmap49113-BTA-30008 | 4 | 1 | Unique |
| 383 | chr12 | 71,749,438 | 71,878,089 | 128,652 | loss | ARS-BFGL-NGS-14529 | ARS-BFGL-NGS-53179 | 3 | 2 | Multiple |
| 384 | chr12 | 75,939,440 | 76,039,896 | 100,457 | loss | BTA-100491-no-rs | Hapmap43198-BTA-19292 | 3 | 1 | Unique |
| 385 | chr12 | 84,165,838 | 84,253,097 | 87,260 | loss | ARS-BFGL-NGS-106663 | ARS-BFGL-NGS-3885 | 4 | 4 | 0.77% |
| 386 | chr12 | 84,428,478 | 84,561,131 | 132,654 | both | ARS-BFGL-NGS-36097 | ARS-BFGL-NGS-23827 | 4 | 8 | 1.54% |
| 387 | chr13 | 131,119 | 1,024,646 | 893,528 | both | ARS-BFGL-NGS-22160 | Hapmap48963-BTA-96734 | 16 | 6 | 1.15% |
| 388 | chr13 | 1,498,184 | 1,549,765 | 51,582 | loss | Hapmap45253-BTA-15908 | ARS-BFGL-NGS-59455 | 3 | 2 | Multiple |
| 389 | chr13 | 3,529,873 | 3,611,970 | 82,098 | both | BTB-01935769 | BTA-117397-no-rs | 3 | 4 | 0.77% |
| 390 | chr13 | 8,036,012 | 8,087,649 | 51,638 | loss | Hapmap38890-BTA-32883 | Hapmap24075-BTA-128035 | 3 | 1 | Unique |
| 391 | chr13 | 10,450,578 | 10,591,791 | 141,214 | gain | ARS-BFGL-NGS-27780 | ARS-BFGL-NGS-6320 | 5 | 2 | Multiple |
| 392 | chr13 | 11,026,549 | 11,334,506 | 307,958 | gain | ARS-BFGL-NGS-2317 | ARS-BFGL-BAC-785 | 6 | 2 | Multiple |
| 393 | chr13 | 12,959,042 | 13,046,436 | 87,395 | loss | BTA-08210-rs29021773 | Hapmap43530-BTA-25745 | 3 | 2 | Multiple |
| 394 | chr13 | 15,639,529 | 15,721,921 | 82,393 | loss | UA-IFASA-8290 | Hapmap41504-BTA-31952 | 3 | 8 | 1.54% |
| 395 | chr13 | 16,458,536 | 17,028,764 | 570,229 | gain | ARS-BFGL-BAC-7407 | Hapmap55207-ss46526284 | 13 | 20 | 3.84% |
| 396 | chr13 | 36,307,098 | 36,417,444 | 110,347 | loss | BTA-17784-no-rs | Hapmap25918-BTA-163236 | 3 | 2 | Multiple |
| 397 | chr13 | 44,681,122 | 44,755,540 | 74,419 | loss | ARS-BFGL-NGS-101595 | ARS-BFGL-NGS-41527 | 3 | 2 | Multiple |
| 398 | chr13 | 50,392,542 | 50,465,143 | 72,602 | gain | ARS-BFGL-NGS-61030 | Hapmap51967-BTA-118944 | 3 | 1 | Unique |
| 399 | chr13 | 70,402,183 | 70,503,529 | 101,347 | gain | ARS-BFGL-NGS-63200 | ARS-BFGL-NGS-78378 | 3 | 4 | 0.77% |
| 400 | chr13 | 71,912,790 | 71,963,853 | 51,064 | loss | Hapmap53091-rs29015689 | ARS-BFGL-NGS-3159 | 3 | 1 | Unique |
| 401 | chr13 | 75,039,650 | 75,099,696 | 60,047 | gain | ARS-BFGL-NGS-105280 | ARS-BFGL-NGS-40101 | 3 | 2 | Multiple |
| 402 | chr14 | 260,342 | 483,499 | 223,158 | loss | ARS-BFGL-NGS-34135 | ARS-BFGL-BAC-26048 | 6 | 1 | Unique |
| 403 | chr14 | 1,215,180 | 1,307,997 | 92,818 | gain | ARS-BFGL-NGS-107222 | ARS-BFGL-NGS-31471 | 4 | 2 | Multiple |
| 404 | chr14 | 2,239,115 | 2,681,399 | 442,285 | gain | Hapmap33328-BTC-064942 | ARS-BFGL-NGS-42263 | 17 | 4 | 0.77% |
| 405 | chr14 | 7,620,649 | 7,731,016 | 110,368 | loss | Hapmap36763-SCAFFOLD240007_5847 | BFGL-NGS-112271 | 4 | 2 | Multiple |
| 406 | chr14 | 8,830,000 | 8,875,203 | 45,204 | loss | Hapmap31972-BTC-057258 | Hapmap26890-BTC-057492 | 3 | 2 | Multiple |
| 407 | chr14 | 12,958,060 | 13,377,785 | 419,726 | both | BFGL-NGS-119174 | ARS-BFGL-NGS-54437 | 14 | 190 | 36.47% |
| 408 | chr14 | 15,281,270 | 15,358,482 | 77,213 | loss | BFGL-NGS-114730 | UA-IFASA-9282 | 3 | 4 | 0.77% |
| 409 | chr14 | 18,004,867 | 18,190,755 | 185,889 | gain | BTB-01720493 | BTB-01283269 | 5 | 1 | Unique |
| 410 | chr14 | 25,514,563 | 25,604,507 | 89,945 | loss | Hapmap26621-BTC-072953 | ARS-BFGL-BAC-1290 | 4 | 2 | Multiple |
| 411 | chr14 | 28,938,889 | 29,056,831 | 117,943 | loss | BTB-01936090 | BTB-00563836 | 4 | 1 | Unique |
| 412 | chr14 | 33,608,812 | 33,680,199 | 71,388 | loss | Hapmap61004-rs29017348 | BFGL-NGS-109998 | 3 | 1 | Unique |
| 413 | chr14 | 36,720,873 | 36,800,835 | 79,963 | gain | BTA-63813-no-rs | BTA-63812-no-rs | 3 | 1 | Unique |
| 414 | chr14 | 45,109,181 | 45,221,196 | 112,016 | loss | ARS-BFGL-NGS-33585 | Hapmap51215-BTA-34925 | 3 | 2 | Multiple |
| 415 | chr14 | 46,902,158 | 47,271,787 | 369,630 | both | Hapmap58389-rs29019587 | UA-IFASA-8223 | 8 | 3 | 0.58% |
| 416 | chr14 | 47,471,892 | 47,614,690 | 142,799 | gain | BTA-111311-no-rs | BTB-01212623 | 4 | 2 | Multiple |
| 417 | chr14 | 48,395,915 | 48,702,852 | 306,938 | loss | BTA-34930-no-rs | ARS-BFGL-BAC-1625 | 9 | 1 | Unique |
| 418 | chr14 | 49,361,995 | 49,577,713 | 215,719 | both | Hapmap46234-BTA-60176 | Hapmap44105-BTA-60185 | 6 | 5 | 0.96% |
| 419 | chr14 | 50,317,495 | 50,390,187 | 72,693 | loss | BTB-00332985 | BTA-104340-no-rs | 3 | 1 | Unique |
| 420 | chr14 | 50,713,046 | 50,866,308 | 153,263 | loss | BTB-00333025 | BTB-00572165 | 3 | 1 | Unique |
| 421 | chr14 | 51,221,633 | 51,268,390 | 46,758 | loss | ARS-BFGL-BAC-23616 | ARS-BFGL-BAC-23606 | 3 | 3 | 0.58% |
| 422 | chr14 | 51,398,555 | 51,548,386 | 149,832 | loss | Hapmap60925-rs29018458 | Hapmap31256-BTC-012280 | 7 | 1 | Unique |
| 423 | chr14 | 51,668,755 | 51,795,751 | 126,997 | loss | Hapmap33279-BTC-012214 | Hapmap23234-BTC-001732 | 5 | 1 | Unique |
| 424 | chr14 | 68,905,113 | 69,014,198 | 109,086 | gain | Hapmap50328-BTA-35439 | BFGL-NGS-110770 | 4 | 5 | 0.96% |
| 425 | chr14 | 72,674,131 | 72,718,667 | 44,537 | gain | ARS-BFGL-NGS-13904 | ARS-BFGL-NGS-843 | 3 | 1 | Unique |
| 426 | chr14 | 75,704,070 | 75,848,910 | 144,841 | loss | ARS-BFGL-NGS-61550 | ARS-BFGL-NGS-63487 | 3 | 3 | 0.58% |
| 427 | chr14 | 76,011,150 | 76,101,692 | 90,543 | gain | BTB-01388619 | BTB-01974109 | 3 | 1 | Unique |
| 428 | chr14 | 80,240,548 | 80,458,252 | 217,705 | loss | Hapmap26378-BTA-129537 | Hapmap34730-BES8_Contig464_1373 | 4 | 1 | Unique |
| 429 | chr15 | 1,707,228 | 2,039,483 | 332,256 | loss | BTA-26081-no-rs | BTB-02021101 | 7 | 5 | 0.96% |
| 430 | chr15 | 3,628,357 | 3,684,759 | 56,403 | loss | BTA-102514-no-rs | BTB-01545112 | 3 | 1 | Unique |
| 431 | chr15 | 5,324,917 | 5,576,671 | 251,755 | loss | BTB-00580383 | Hapmap23284-BTA-162040 | 4 | 1 | Unique |
| 432 | chr15 | 6,452,018 | 6,585,863 | 133,846 | gain | ARS-BFGL-NGS-72772 | BTB-02089263 | 5 | 4 | 0.77% |
| 433 | chr15 | 7,072,319 | 7,262,823 | 190,505 | gain | Hapmap49562-BTA-29896 | BTB-01417458 | 3 | 1 | Unique |
| 434 | chr15 | 8,167,866 | 8,385,314 | 217,449 | both | ARS-BFGL-NGS-26612 | BTB-00580638 | 5 | 9 | 1.73% |
| 435 | chr15 | 8,816,057 | 9,581,859 | 765,803 | loss | ARS-BFGL-BAC-18229 | BTA-94770-no-rs | 15 | 12 | 2.30% |
| 436 | chr15 | 10,136,876 | 10,262,929 | 126,054 | both | BTB-01384592 | Hapmap44444-BTA-115434 | 4 | 4 | 0.77% |
| 437 | chr15 | 13,270,175 | 13,453,250 | 183,076 | loss | ARS-BFGL-BAC-31586 | Hapmap26711-BTA-149759 | 4 | 1 | Unique |
| 438 | chr15 | 14,632,044 | 14,729,248 | 97,205 | gain | BTA-121003-no-rs | Hapmap44541-BTA-37632 | 3 | 2 | Multiple |
| 439 | chr15 | 15,734,447 | 15,796,587 | 62,141 | gain | ARS-BFGL-BAC-2180 | BTB-01262385 | 3 | 1 | Unique |
| 440 | chr15 | 18,583,219 | 18,646,489 | 63,271 | gain | ARS-BFGL-NGS-104408 | BTA-05407-rs29019567 | 3 | 2 | Multiple |
| 441 | chr15 | 19,267,911 | 19,316,067 | 48,157 | gain | Hapmap38222-BTA-38012 | BTB-00582817 | 3 | 1 | Unique |
| 442 | chr15 | 22,541,607 | 22,627,194 | 85,588 | loss | ARS-BFGL-NGS-19320 | ARS-BFGL-BAC-20623 | 4 | 2 | Multiple |
| 443 | chr15 | 45,003,171 | 45,268,074 | 264,904 | gain | BTA-109760-no-rs | BFGL-NGS-115713 | 7 | 38 | 7.29% |
| 444 | chr15 | 46,321,011 | 46,424,312 | 103,302 | gain | ARS-BFGL-NGS-32444 | BTB-01881519 | 4 | 1 | Unique |
| 445 | chr15 | 47,889,321 | 48,459,943 | 570,623 | gain | ARS-BFGL-BAC-20572 | BTB-01798272 | 7 | 5 | 0.96% |
| 446 | chr15 | 49,158,251 | 49,529,709 | 371,459 | gain | BTB-01371877 | BTB-01873927 | 3 | 2 | Multiple |
| 447 | chr15 | 50,905,152 | 50,984,522 | 79,371 | both | UA-IFASA-8473 | BTB-02036460 | 4 | 12 | 2.30% |
| 448 | chr15 | 55,887,421 | 55,971,213 | 83,793 | loss | BTB-00519537 | BTB-00606070 | 3 | 1 | Unique |
| 449 | chr15 | 56,384,719 | 56,557,487 | 172,769 | loss | ARS-BFGL-BAC-19996 | ARS-BFGL-BAC-19991 | 7 | 6 | 1.15% |
| 450 | chr15 | 68,481,032 | 68,591,967 | 110,936 | gain | ARS-BFGL-NGS-26597 | ARS-BFGL-NGS-74913 | 4 | 3 | 0.58% |
| 451 | chr15 | 68,858,924 | 68,978,182 | 119,259 | both | Hapmap50885-BTA-121034 | BTA-27784-no-rs | 4 | 5 | 0.96% |
| 452 | chr15 | 69,779,831 | 69,924,552 | 144,722 | loss | BTA-89505-no-rs | BTA-89498-no-rs | 5 | 1 | Unique |
| 453 | chr15 | 78,970,733 | 79,340,261 | 369,529 | gain | ARS-BFGL-NGS-94527 | BTB-02012820 | 8 | 4 | 0.77% |
| 454 | chr15 | 79,583,278 | 80,226,852 | 643,575 | both | BTB-01690781 | BTB-01673658 | 11 | 172 | 33.01% |
| 455 | chr15 | 82,791,875 | 82,889,460 | 97,586 | gain | BTB-00622199 | UA-IFASA-7603 | 5 | 45 | 8.64% |
| 456 | chr15 | 83,328,724 | 83,519,235 | 190,512 | gain | BTB-01821444 | BTB-00622270 | 5 | 35 | 6.72% |
| 457 | chr15 | 84,155,279 | 84,271,828 | 116,550 | loss | Hapmap24864-BTA-155172 | ARS-BFGL-NGS-42520 | 5 | 4 | 0.77% |
| 458 | chr15 | 84,403,743 | 84,598,267 | 194,525 | loss | UA-IFASA-5953 | ARS-BFGL-NGS-24582 | 5 | 1 | Unique |
| 459 | chr16 | 3,970,643 | 4,155,953 | 185,311 | gain | ARS-BFGL-NGS-54578 | BTB-01958134 | 4 | 28 | 5.37% |
| 460 | chr16 | 4,623,920 | 4,718,732 | 94,813 | both | BTB-02010595 | Hapmap33722-BTA-155362 | 3 | 4 | 0.77% |
| 461 | chr16 | 5,143,952 | 5,522,350 | 378,399 | gain | BTB-01973077 | BTB-02077556 | 3 | 1 | Unique |
| 462 | chr16 | 6,703,995 | 6,849,563 | 145,569 | loss | BTB-01541076 | BTA-107144-no-rs | 5 | 2 | Multiple |
| 463 | chr16 | 7,213,773 | 7,480,522 | 266,750 | loss | BTB-01293013 | BTB-01221760 | 10 | 1 | Unique |
| 464 | chr16 | 7,832,336 | 8,085,819 | 253,484 | loss | ARS-BFGL-BAC-6371 | ARS-BFGL-NGS-101869 | 7 | 11 | 2.11% |
| 465 | chr16 | 8,113,183 | 8,334,137 | 220,955 | loss | BTA-39609-no-rs | Hapmap43811-BTA-22208 | 9 | 4 | 0.77% |
| 466 | chr16 | 8,667,471 | 8,899,608 | 232,138 | both | Hapmap26204-BTA-160802 | BTB-01402663 | 6 | 2 | Multiple |
| 467 | chr16 | 8,972,958 | 9,042,654 | 69,697 | loss | Hapmap33576-BTA-130425 | BTB-01533537 | 3 | 1 | Unique |
| 468 | chr16 | 14,267,595 | 14,416,109 | 148,515 | loss | BTA-91807-no-rs | BTA-91792-no-rs | 5 | 1 | Unique |
| 469 | chr16 | 14,511,568 | 14,624,913 | 113,346 | gain | BTA-27064-no-rs | BTA-122806-no-rs | 3 | 1 | Unique |
| 470 | chr16 | 14,882,504 | 15,353,483 | 470,980 | both | BTA-06888-no-rs | BTB-01113828 | 7 | 10 | 1.92% |
| 471 | chr16 | 40,726,229 | 41,077,367 | 351,139 | gain | ARS-BFGL-NGS-16286 | ARS-BFGL-NGS-12988 | 4 | 4 | 0.77% |
| 472 | chr16 | 46,815,495 | 47,006,427 | 190,933 | loss | BFGL-NGS-116784 | ARS-BFGL-NGS-44177 | 7 | 1 | Unique |
| 473 | chr16 | 54,755,641 | 54,946,702 | 191,062 | gain | ARS-BFGL-BAC-34694 | BFGL-NGS-116297 | 5 | 4 | 0.77% |
| 474 | chr16 | 57,780,413 | 57,887,734 | 107,322 | loss | BTB-00650039 | BTB-00650136 | 4 | 1 | Unique |
| 475 | chr16 | 58,132,374 | 58,364,305 | 231,932 | gain | BTB-01732320 | BTB-00653808 | 6 | 2 | Multiple |
| 476 | chr16 | 65,790,480 | 66,177,878 | 387,399 | loss | BTA-90680-no-rs | BFGL-NGS-113069 | 9 | 1 | Unique |
| 477 | chr16 | 69,624,481 | 69,702,424 | 77,944 | gain | BFGL-NGS-119389 | BTB-00660988 | 3 | 1 | Unique |
| 478 | chr16 | 70,515,327 | 70,566,371 | 51,045 | gain | ARS-BFGL-NGS-31253 | ARS-BFGL-BAC-27042 | 3 | 2 | Multiple |
| 479 | chr16 | 71,332,975 | 71,422,020 | 89,046 | loss | Hapmap39023-BTA-39937 | Hapmap41860-BTA-39932 | 4 | 1 | Unique |
| 480 | chr17 | 6,950,827 | 7,074,359 | 123,533 | loss | Hapmap32996-BTA-131276 | Hapmap36186-SCAFFOLD206949_1268 | 4 | 1 | Unique |
| 481 | chr17 | 12,274,583 | 12,392,661 | 118,079 | loss | ARS-BFGL-NGS-15402 | BTA-21866-no-rs | 3 | 5 | 0.96% |
| 482 | chr17 | 15,002,419 | 15,334,518 | 332,100 | both | Hapmap33086-BTA-19277 | ARS-BFGL-NGS-82854 | 7 | 16 | 3.07% |
| 483 | chr17 | 15,769,734 | 15,825,282 | 55,549 | loss | Hapmap60968-rs29016935 | BTB-00673952 | 3 | 1 | Unique |
| 484 | chr17 | 19,895,917 | 19,983,638 | 87,722 | loss | Hapmap51224-BTA-40595 | ARS-USMARC-Parent-AY858890-rs29002256 | 4 | 1 | Unique |
| 485 | chr17 | 23,724,251 | 29,293,341 | 5,569,091 | both | Hapmap55890-rs29027310 | ARS-BFGL-NGS-101527 | 85 | 21 | 4.03% |
| 486 | chr17 | 32,967,064 | 33,121,544 | 154,481 | loss | Hapmap28180-BTA-152938 | Hapmap51554-BTA-114371 | 5 | 4 | 0.77% |
| 487 | chr17 | 38,427,635 | 38,583,109 | 155,475 | gain | Hapmap43386-BTA-87090 | UA-IFASA-6417 | 3 | 1 | Unique |
| 488 | chr17 | 44,850,189 | 45,980,011 | 1,129,823 | gain | BTB-01220886 | Hapmap54457-rs29016184 | 22 | 1 | Unique |
| 489 | chr17 | 62,021,629 | 62,097,079 | 75,451 | gain | BTA-115762-no-rs | ARS-BFGL-NGS-54448 | 3 | 2 | Multiple |
| 490 | chr17 | 70,215,139 | 70,269,135 | 53,997 | loss | ARS-BFGL-NGS-11355 | BTB-01121732 | 3 | 1 | Unique |
| 491 | chr17 | 71,389,254 | 71,531,747 | 142,494 | gain | Hapmap27966-BTA-149885 | ARS-BFGL-NGS-103734 | 3 | 1 | Unique |
| 492 | chr17 | 72,890,151 | 73,116,926 | 226,776 | gain | ARS-BFGL-NGS-98109 | BTB-00689266 | 8 | 1 | Unique |
| 493 | chr17 | 74,325,960 | 74,420,465 | 94,506 | loss | ARS-BFGL-NGS-14354 | ARS-BFGL-NGS-41599 | 4 | 3 | 0.58% |
| 494 | chr17 | 74,809,501 | 76,487,768 | 1,678,268 | both | ARS-BFGL-NGS-74649 | ARS-BFGL-NGS-35060 | 46 | 19 | 3.65% |
| 495 | chr18 | 799,464 | 862,947 | 63,484 | loss | ARS-BFGL-NGS-88592 | BFGL-NGS-114633 | 3 | 1 | Unique |
| 496 | chr18 | 2,169,881 | 2,240,907 | 71,027 | loss | ARS-BFGL-NGS-16731 | Hapmap49988-BTA-42565 | 3 | 2 | Multiple |
| 497 | chr18 | 3,532,889 | 3,635,169 | 102,281 | loss | ARS-BFGL-NGS-76950 | BTA-121112-no-rs | 3 | 1 | Unique |
| 498 | chr18 | 11,604,818 | 11,696,665 | 91,848 | gain | ARS-BFGL-BAC-33119 | BFGL-NGS-116678 | 3 | 7 | 1.34% |
| 499 | chr18 | 13,657,912 | 13,829,583 | 171,672 | gain | ARS-BFGL-NGS-72034 | ARS-BFGL-NGS-28493 | 4 | 2 | Multiple |
| 500 | chr18 | 20,977,959 | 21,098,149 | 120,191 | loss | Hapmap51594-BTA-42721 | BTA-42733-no-rs | 3 | 1 | Unique |
| 501 | chr18 | 27,658,230 | 28,018,549 | 360,320 | gain | ARS-BFGL-NGS-36647 | ARS-BFGL-NGS-108426 | 6 | 4 | 0.77% |
| 502 | chr18 | 34,866,810 | 34,934,949 | 68,140 | loss | BFGL-NGS-113568 | ARS-BFGL-NGS-52078 | 3 | 2 | Multiple |
| 503 | chr18 | 37,321,698 | 37,401,683 | 79,986 | gain | Hapmap48151-BTA-101548 | ARS-BFGL-NGS-12047 | 3 | 1 | Unique |
| 504 | chr18 | 37,927,444 | 38,051,481 | 124,038 | gain | Hapmap56187-rs29014149 | Hapmap24078-BTA-132268 | 3 | 5 | 0.96% |
| 505 | chr18 | 44,010,754 | 44,081,244 | 70,491 | gain | Hapmap40732-BTA-43433 | BTA-11776-rs29017530 | 3 | 2 | Multiple |
| 506 | chr18 | 50,348,554 | 50,583,693 | 235,140 | gain | BFGL-NGS-118266 | Hapmap45491-BTA-43726 | 4 | 19 | 3.65% |
| 507 | chr18 | 52,748,165 | 52,808,308 | 60,144 | gain | ARS-BFGL-BAC-34398 | ARS-BFGL-NGS-71842 | 3 | 4 | 0.77% |
| 508 | chr18 | 54,444,810 | 54,632,111 | 187,302 | gain | ARS-BFGL-NGS-10623 | ARS-BFGL-NGS-70161 | 5 | 12 | 2.30% |
| 509 | chr18 | 57,046,784 | 57,282,827 | 236,044 | both | BTB-00730053 | BTA-43921-no-rs | 8 | 80 | 15.36% |
| 510 | chr18 | 58,544,308 | 59,220,972 | 676,665 | gain | ARS-BFGL-NGS-15484 | BTB-01425816 | 10 | 3 | 0.58% |
| 511 | chr18 | 59,720,625 | 59,878,689 | 158,065 | gain | BTB-01915827 | BFGL-NGS-114157 | 3 | 1 | Unique |
| 512 | chr18 | 60,286,569 | 60,667,576 | 381,008 | gain | BFGL-NGS-113553 | BTB-01457811 | 7 | 3 | 0.58% |
| 513 | chr18 | 60,795,899 | 60,958,687 | 162,789 | gain | ARS-BFGL-NGS-99840 | BTB-01960462 | 4 | 1 | Unique |
| 514 | chr18 | 61,142,079 | 61,607,122 | 465,044 | both | ARS-BFGL-NGS-11218 | BFGL-NGS-110828 | 12 | 37 | 7.10% |
| 515 | chr18 | 62,981,152 | 63,217,107 | 235,956 | loss | ARS-BFGL-NGS-78563 | BFGL-NGS-119439 | 4 | 2 | Multiple |
| 516 | chr19 | 4,423,766 | 4,478,846 | 55,081 | gain | Hapmap41273-BTA-46442 | ARS-BFGL-NGS-34992 | 3 | 1 | Unique |
| 517 | chr19 | 4,704,817 | 4,884,064 | 179,248 | loss | ARS-BFGL-NGS-102180 | BFGL-NGS-114778 | 4 | 2 | Multiple |
| 518 | chr19 | 5,247,360 | 5,341,359 | 94,000 | gain | BTB-02044220 | ARS-BFGL-NGS-107736 | 3 | 1 | Unique |
| 519 | chr19 | 10,154,276 | 10,255,994 | 101,719 | gain | ARS-BFGL-NGS-37614 | Hapmap30007-BTA-147050 | 3 | 1 | Unique |
| 520 | chr19 | 10,863,763 | 10,997,446 | 133,684 | loss | Hapmap52907-rs29010949 | ARS-BFGL-NGS-40746 | 5 | 2 | Multiple |
| 521 | chr19 | 11,810,936 | 11,885,688 | 74,753 | gain | UA-IFASA-6460 | ARS-BFGL-BAC-36180 | 5 | 4 | 0.77% |
| 522 | chr19 | 15,887,584 | 15,973,022 | 85,439 | gain | Hapmap53903-rs29027471 | ARS-BFGL-NGS-26149 | 4 | 2 | Multiple |
| 523 | chr19 | 21,944,913 | 22,027,436 | 82,524 | gain | ARS-BFGL-NGS-100026 | BFGL-NGS-112338 | 3 | 2 | Multiple |
| 524 | chr19 | 32,317,789 | 32,384,883 | 67,095 | loss | Hapmap59534-rs29022537 | BTA-05960-no-rs | 3 | 2 | Multiple |
| 525 | chr19 | 35,627,984 | 35,690,368 | 62,385 | loss | ARS-BFGL-NGS-101953 | ARS-BFGL-NGS-4759 | 3 | 1 | Unique |
| 526 | chr19 | 43,230,005 | 43,301,159 | 71,155 | loss | BFGL-NGS-119419 | ARS-BFGL-NGS-19337 | 3 | 1 | Unique |
| 527 | chr19 | 51,181,806 | 51,241,411 | 59,606 | both | ARS-BFGL-NGS-104238 | ARS-BFGL-NGS-22742 | 3 | 2 | Multiple |
| 528 | chr19 | 55,900,714 | 56,044,375 | 143,662 | gain | UA-IFASA-6029 | BFGL-NGS-116713 | 5 | 1 | Unique |
| 529 | chr19 | 58,447,838 | 58,596,190 | 148,353 | gain | ARS-BFGL-NGS-28678 | ARS-BFGL-NGS-34845 | 4 | 12 | 2.30% |
| 530 | chr19 | 63,651,365 | 63,762,752 | 111,388 | loss | UA-IFASA-9580 | ARS-BFGL-NGS-39527 | 3 | 2 | Multiple |
| 531 | chr20 | 20,322,167 | 20,383,438 | 61,272 | loss | BTA-110416-no-rs | BTA-110421-no-rs | 3 | 1 | Unique |
| 532 | chr20 | 30,206,385 | 30,271,213 | 64,829 | loss | BTA-50178-no-rs | ARS-BFGL-BAC-27918 | 3 | 2 | Multiple |
| 533 | chr20 | 31,750,025 | 31,832,019 | 81,995 | loss | BTB-01336444 | ARS-BFGL-BAC-27933 | 3 | 1 | Unique |
| 534 | chr20 | 37,103,943 | 37,184,011 | 80,069 | loss | BTB-00778405 | ARS-BFGL-BAC-31754 | 4 | 2 | Multiple |
| 535 | chr20 | 38,261,471 | 38,325,407 | 63,937 | gain | Hapmap53443-rs29021255 | Hapmap40359-BTA-50418 | 3 | 2 | Multiple |
| 536 | chr20 | 41,766,742 | 42,123,336 | 356,595 | loss | Hapmap31493-BTA-155702 | ARS-BFGL-NGS-14869 | 9 | 1 | Unique |
| 537 | chr20 | 45,581,511 | 46,191,558 | 610,048 | both | BTB-01263230 | ARS-BFGL-BAC-34877 | 15 | 7 | 1.34% |
| 538 | chr20 | 46,603,190 | 46,767,627 | 164,438 | both | BTB-01908187 | BTB-01441573 | 5 | 19 | 3.65% |
| 539 | chr20 | 47,747,151 | 47,808,154 | 61,004 | loss | ARS-BFGL-BAC-27247 | Hapmap41127-BTA-101667 | 3 | 1 | Unique |
| 540 | chr20 | 48,423,319 | 48,948,264 | 524,946 | loss | Hapmap32803-BTA-135212 | ARS-BFGL-BAC-32350 | 15 | 6 | 1.15% |
| 541 | chr20 | 49,510,403 | 49,948,734 | 438,332 | loss | Hapmap39563-BTA-41519 | BTB-01905047 | 13 | 5 | 0.96% |
| 542 | chr20 | 50,487,253 | 50,564,369 | 77,117 | loss | BTA-92533-no-rs | Hapmap54884-rs29017180 | 3 | 2 | Multiple |
| 543 | chr20 | 52,831,351 | 53,386,605 | 555,255 | both | ARS-BFGL-NGS-31163 | Hapmap54729-rs29023630 | 9 | 8 | 1.54% |
| 544 | chr20 | 54,410,151 | 55,030,958 | 620,808 | loss | BTB-00787134 | BTB-01748945 | 15 | 3 | 0.58% |
| 545 | chr20 | 55,385,094 | 55,518,976 | 133,883 | gain | BTB-01132101 | ARS-BFGL-NGS-40803 | 3 | 2 | Multiple |
| 546 | chr20 | 56,494,412 | 56,713,120 | 218,709 | loss | BTB-00787949 | UA-IFASA-4131 | 4 | 1 | Unique |
| 547 | chr20 | 57,091,179 | 57,345,282 | 254,104 | loss | BTB-00788635 | BTB-01435027 | 6 | 2 | Multiple |
| 548 | chr20 | 57,825,955 | 57,985,975 | 160,021 | loss | BTB-01127950 | BTB-01128234 | 5 | 2 | Multiple |
| 549 | chr20 | 58,927,207 | 59,260,303 | 333,097 | loss | Hapmap35564-SCAFFOLD115952_737 | BTB-01304704 | 4 | 3 | 0.58% |
| 550 | chr20 | 63,190,892 | 63,363,024 | 172,133 | loss | BTB-00790682 | Hapmap42134-BTA-21475 | 3 | 2 | Multiple |
| 551 | chr20 | 68,015,859 | 68,081,331 | 65,473 | gain | BTA-121292-no-rs | ARS-BFGL-NGS-21633 | 3 | 2 | Multiple |
| 552 | chr20 | 74,919,445 | 75,346,434 | 426,990 | loss | BTB-00572445 | BFGL-NGS-114380 | 12 | 1 | Unique |
| 553 | chr21 | 11,877,645 | 11,935,054 | 57,410 | gain | ARS-BFGL-NGS-11443 | BTA-53438-no-rs | 3 | 1 | Unique |
| 554 | chr21 | 14,654,138 | 14,725,723 | 71,586 | gain | ARS-BFGL-NGS-9271 | ARS-BFGL-NGS-100582 | 3 | 1 | Unique |
| 555 | chr21 | 16,638,720 | 16,734,668 | 95,949 | gain | DIAS-242 | Hapmap26740-BTA-155993 | 4 | 1 | Unique |
| 556 | chr21 | 19,426,586 | 19,682,085 | 255,500 | gain | ARS-BFGL-NGS-92246 | BTB-01787714 | 4 | 1 | Unique |
| 557 | chr21 | 19,839,634 | 19,988,738 | 149,105 | gain | ARS-BFGL-NGS-106149 | BTA-121314-no-rs | 5 | 6 | 1.15% |
| 558 | chr21 | 29,363,187 | 29,437,216 | 74,030 | loss | BTB-01107683 | Hapmap49946-BTA-29235 | 3 | 6 | 1.15% |
| 559 | chr21 | 33,431,258 | 33,498,729 | 67,472 | loss | Hapmap24204-BTA-153183 | Hapmap32329-BTA-147054 | 3 | 2 | Multiple |
| 560 | chr21 | 36,837,821 | 36,994,970 | 157,150 | loss | BTB-01303836 | BTB-01303818 | 4 | 2 | Multiple |
| 561 | chr21 | 38,217,249 | 38,297,681 | 80,433 | gain | Hapmap53995-rs29024931 | BTB-02072074 | 3 | 1 | Unique |
| 562 | chr21 | 40,541,634 | 40,965,381 | 423,748 | both | BTA-52239-no-rs | Hapmap32050-BTA-135951 | 12 | 10 | 1.92% |
| 563 | chr21 | 42,187,203 | 42,546,643 | 359,441 | gain | BFGL-NGS-111692 | ARS-BFGL-NGS-43635 | 8 | 4 | 0.77% |
| 564 | chr21 | 42,735,585 | 42,848,351 | 112,767 | gain | Hapmap39342-BTA-52219 | ARS-BFGL-BAC-29181 | 3 | 1 | Unique |
| 565 | chr21 | 51,616,048 | 51,719,711 | 103,664 | gain | Hapmap24313-BTA-29957 | BTB-01375511 | 4 | 2 | Multiple |
| 566 | chr21 | 51,877,182 | 52,064,649 | 187,468 | gain | BTB-01172066 | BTA-12087-no-rs | 4 | 8 | 1.54% |
| 567 | chr21 | 53,880,695 | 53,932,826 | 52,132 | loss | BTB-00649148 | ARS-BFGL-NGS-33035 | 3 | 1 | Unique |
| 568 | chr21 | 67,893,589 | 67,982,081 | 88,493 | gain | BFGL-NGS-115062 | ARS-BFGL-NGS-71969 | 3 | 5 | 0.96% |
| 569 | chr21 | 68,992,562 | 69,171,298 | 178,737 | gain | ARS-BFGL-NGS-104354 | ARS-BFGL-NGS-2644 | 7 | 4 | 0.77% |
| 570 | chr22 | 6,167,860 | 6,421,625 | 253,766 | loss | ARS-BFGL-NGS-66672 | ARS-BFGL-NGS-98305 | 7 | 2 | Multiple |
| 571 | chr22 | 12,075,818 | 12,126,595 | 50,778 | gain | ARS-BFGL-NGS-109636 | BFGL-NGS-118910 | 3 | 1 | Unique |
| 572 | chr22 | 18,605,560 | 18,668,106 | 62,547 | loss | ARS-BFGL-NGS-30495 | ARS-BFGL-NGS-26299 | 3 | 1 | Unique |
| 573 | chr22 | 20,031,541 | 20,093,810 | 62,270 | gain | Hapmap46118-BTA-108252 | BFGL-NGS-116099 | 3 | 2 | Multiple |
| 574 | chr22 | 35,672,288 | 35,838,375 | 166,088 | loss | ARS-BFGL-NGS-870 | BTA-54170-no-rs | 6 | 11 | 2.11% |
| 575 | chr22 | 54,765,322 | 54,919,565 | 154,244 | loss | ARS-BFGL-NGS-51980 | Hapmap2744-BTA-121362 | 5 | 1 | Unique |
| 576 | chr22 | 60,480,755 | 60,619,340 | 138,586 | both | ARS-BFGL-NGS-30728 | ARS-BFGL-NGS-13887 | 6 | 3 | 0.58% |
| 577 | chr22 | 61,117,993 | 61,279,256 | 161,264 | loss | ARS-BFGL-NGS-16841 | ARS-BFGL-NGS-101683 | 5 | 2 | Multiple |
| 578 | chr22 | 61,418,796 | 61,559,595 | 140,800 | gain | Hapmap39470-BTA-121373 | Hapmap30583-BTA-157589 | 5 | 1 | Unique |
| 579 | chr23 | 291,944 | 376,906 | 84,963 | loss | Hapmap31772-BTA-144780 | BTA-57034-no-rs | 3 | 1 | Unique |
| 580 | chr23 | 1,829,364 | 1,919,098 | 89,735 | loss | ARS-BFGL-BAC-31235 | Hapmap44551-BTA-55507 | 4 | 1 | Unique |
| 581 | chr23 | 8,866,725 | 8,952,822 | 86,098 | gain | ARS-BFGL-NGS-107320 | Hapmap24244-BTA-114119 | 3 | 1 | Unique |
| 582 | chr23 | 13,438,907 | 13,582,339 | 143,433 | gain | ARS-BFGL-NGS-2043 | ARS-BFGL-NGS-42525 | 6 | 1 | Unique |
| 583 | chr23 | 16,935,143 | 17,149,313 | 214,171 | both | ARS-BFGL-NGS-101390 | ARS-BFGL-BAC-28908 | 4 | 4 | 0.77% |
| 584 | chr23 | 22,484,237 | 22,727,191 | 242,955 | both | Hapmap40178-BTA-55802 | Hapmap44684-BTA-55791 | 7 | 7 | 1.34% |
| 585 | chr23 | 26,567,859 | 26,641,305 | 73,447 | gain | BTB-02046534 | BTB-02053415 | 3 | 4 | 0.77% |
| 586 | chr23 | 27,745,731 | 27,977,782 | 232,052 | gain | ARS-BFGL-NGS-99242 | ARS-BFGL-NGS-32979 | 8 | 24 | 4.61% |
| 587 | chr23 | 28,969,967 | 29,058,543 | 88,577 | gain | ARS-BFGL-NGS-105563 | ARS-BFGL-NGS-105966 | 3 | 2 | Multiple |
| 588 | chr23 | 30,661,699 | 30,778,606 | 116,908 | gain | Hapmap39414-BTA-56194 | ARS-BFGL-NGS-31776 | 5 | 1 | Unique |
| 589 | chr23 | 45,260,157 | 45,514,921 | 254,765 | loss | BTA-56690-no-rs | BFGL-NGS-115605 | 5 | 3 | 0.58% |
| 590 | chr23 | 52,611,474 | 52,869,716 | 258,243 | loss | ARS-BFGL-NGS-17155 | BFGL-NGS-117465 | 9 | 1 | Unique |
| 591 | chr24 | 466,797 | 634,720 | 167,924 | loss | ARS-BFGL-NGS-10928 | ARS-BFGL-NGS-39061 | 5 | 1 | Unique |
| 592 | chr24 | 3,462,469 | 3,510,715 | 48,247 | loss | ARS-BFGL-NGS-107177 | BTB-00877502 | 3 | 1 | Unique |
| 593 | chr24 | 5,775,857 | 5,966,663 | 190,807 | loss | BTA-58814-no-rs | Hapmap36731-SCAFFOLD236442_391 | 7 | 4 | 0.77% |
| 594 | chr24 | 9,338,076 | 9,443,776 | 105,701 | gain | ARS-BFGL-BAC-44134 | UA-IFASA-4296 | 4 | 2 | Multiple |
| 595 | chr24 | 12,657,549 | 12,722,061 | 64,513 | loss | BTA-57412-no-rs | Hapmap48000-BTA-57409 | 3 | 4 | 0.77% |
| 596 | chr24 | 13,615,287 | 13,763,058 | 147,772 | both | BTA-91085-no-rs | ARS-BFGL-NGS-86120 | 5 | 2 | Multiple |
| 597 | chr24 | 22,028,143 | 22,121,651 | 93,509 | gain | ARS-BFGL-NGS-106450 | ARS-BFGL-NGS-78497 | 4 | 1 | Unique |
| 598 | chr24 | 22,407,965 | 22,479,244 | 71,280 | loss | ARS-BFGL-NGS-61055 | ARS-BFGL-NGS-2686 | 3 | 2 | Multiple |
| 599 | chr24 | 28,778,307 | 28,938,521 | 160,215 | loss | BTA-109230-no-rs | Hapmap53299-rs29024075 | 7 | 3 | 0.58% |
| 600 | chr24 | 29,444,695 | 29,570,879 | 126,185 | loss | BTB-01485872 | Hapmap54981-rs29019846 | 5 | 1 | Unique |
| 601 | chr24 | 30,492,140 | 30,601,669 | 109,530 | gain | BTB-00885512 | BTB-00885423 | 3 | 1 | Unique |
| 602 | chr24 | 45,601,085 | 45,705,538 | 104,454 | loss | Hapmap51792-BTA-103575 | ARS-BFGL-NGS-67897 | 4 | 1 | Unique |
| 603 | chr24 | 52,778,973 | 52,914,946 | 135,974 | gain | ARS-BFGL-NGS-93376 | Hapmap42986-BTA-58601 | 3 | 1 | Unique |
| 604 | chr24 | 53,572,851 | 53,687,242 | 114,392 | loss | BTA-29467-no-rs | ARS-BFGL-NGS-117 | 3 | 1 | Unique |
| 605 | chr25 | 924,554 | 1,087,629 | 163,076 | loss | ARS-BFGL-NGS-12001 | ARS-BFGL-NGS-14220 | 5 | 1 | Unique |
| 606 | chr25 | 1,187,949 | 1,265,318 | 77,370 | both | BFGL-NGS-119486 | ARS-BFGL-NGS-62237 | 4 | 5 | 0.96% |
| 607 | chr25 | 1,375,336 | 1,436,322 | 60,987 | loss | BFGL-NGS-115164 | ARS-BFGL-NGS-4009 | 3 | 1 | Unique |
| 608 | chr25 | 1,888,811 | 1,938,257 | 49,447 | loss | ARS-BFGL-BAC-44214 | Hapmap31901-BTC-016378 | 3 | 1 | Unique |
| 609 | chr25 | 8,230,074 | 8,334,560 | 104,487 | gain | ARS-BFGL-NGS-3547 | Hapmap42268-BTA-60619 | 4 | 2 | Multiple |
| 610 | chr25 | 10,603,003 | 10,677,203 | 74,201 | gain | ARS-BFGL-NGS-43303 | BFGL-NGS-118929 | 3 | 1 | Unique |
| 611 | chr25 | 12,129,670 | 12,181,295 | 51,626 | loss | ARS-BFGL-NGS-102275 | ARS-BFGL-NGS-35331 | 3 | 2 | Multiple |
| 612 | chr25 | 38,022,991 | 38,089,451 | 66,461 | loss | ARS-BFGL-NGS-13197 | ARS-BFGL-NGS-5763 | 4 | 1 | Unique |
| 613 | chr25 | 40,835,213 | 41,074,508 | 239,296 | loss | ARS-BFGL-NGS-19270 | ARS-BFGL-NGS-43022 | 7 | 2 | Multiple |
| 614 | chr25 | 42,541,204 | 42,691,922 | 150,719 | gain | ARS-BFGL-BAC-47313 | Hapmap27068-BTC-031603 | 6 | 2 | Multiple |
| 615 | chr25 | 43,623,344 | 43,697,097 | 73,754 | loss | ARS-BFGL-NGS-21354 | ARS-BFGL-NGS-26308 | 3 | 1 | Unique |
| 616 | chr26 | 1,966,809 | 2,430,290 | 463,482 | loss | BTA-26713-no-rs | Hapmap51817-BTA-22015 | 6 | 2 | Multiple |
| 617 | chr26 | 2,527,237 | 2,658,317 | 131,081 | loss | Hapmap60383-rs29019554 | Hapmap44471-BTA-61074 | 4 | 1 | Unique |
| 618 | chr26 | 3,500,486 | 3,760,091 | 259,606 | loss | BTA-61571-no-rs | BTB-00920733 | 7 | 4 | 0.77% |
| 619 | chr26 | 5,177,986 | 5,318,046 | 140,061 | loss | ARS-BFGL-NGS-80925 | ARS-BFGL-NGS-13248 | 5 | 5 | 0.96% |
| 620 | chr26 | 14,031,690 | 14,127,641 | 95,952 | loss | ARS-BFGL-NGS-16205 | Hapmap38926-BTA-94783 | 4 | 2 | Multiple |
| 621 | chr26 | 17,376,061 | 17,486,445 | 110,385 | loss | BFGL-NGS-118205 | ARS-BFGL-NGS-107286 | 5 | 1 | Unique |
| 622 | chr26 | 19,180,038 | 19,331,787 | 151,750 | loss | BTB-01985838 | Hapmap31450-BTA-147701 | 5 | 1 | Unique |
| 623 | chr26 | 20,747,919 | 20,829,586 | 81,668 | gain | Hapmap39788-BTA-87624 | Hapmap48023-BTA-62139 | 3 | 2 | Multiple |
| 624 | chr26 | 24,656,664 | 24,837,303 | 180,640 | loss | ARS-BFGL-NGS-102845 | ARS-BFGL-NGS-1092 | 5 | 1 | Unique |
| 625 | chr26 | 28,475,780 | 28,536,015 | 60,236 | loss | Hapmap38478-BTA-20824 | ARS-BFGL-NGS-55727 | 3 | 5 | 0.96% |
| 626 | chr26 | 29,087,354 | 29,144,134 | 56,781 | loss | BTB-01547063 | BTB-01883871 | 3 | 2 | Multiple |
| 627 | chr26 | 29,183,334 | 29,247,528 | 64,195 | gain | BTB-01851763 | BTB-01583927 | 3 | 1 | Unique |
| 628 | chr26 | 29,393,749 | 29,483,852 | 90,104 | gain | BTB-01584166 | BTB-01619529 | 4 | 1 | Unique |
| 629 | chr26 | 33,369,701 | 33,442,530 | 72,830 | loss | Hapmap56942-ss46526051 | BTA-61189-no-rs | 3 | 1 | Unique |
| 630 | chr26 | 40,780,926 | 41,035,521 | 254,596 | loss | BTA-100861-no-rs | BFGL-NGS-112635 | 6 | 1 | Unique |
| 631 | chr26 | 49,092,128 | 49,170,960 | 78,833 | loss | BFGL-NGS-118574 | ARS-BFGL-NGS-76550 | 4 | 4 | 0.77% |
| 632 | chr26 | 50,804,300 | 51,276,688 | 472,389 | loss | ARS-BFGL-NGS-101725 | ARS-BFGL-NGS-28608 | 15 | 2 | Multiple |
| 633 | chr26 | 51,487,473 | 51,695,334 | 207,862 | loss | ARS-BFGL-NGS-948 | BFGL-NGS-119784 | 9 | 2 | Multiple |
| 634 | chr27 | 6,239,288 | 6,334,196 | 94,909 | both | ARS-BFGL-NGS-55443 | BTA-91332-no-rs | 3 | 5 | 0.96% |
| 635 | chr27 | 7,231,322 | 7,737,542 | 506,221 | both | BTB-02030774 | BFGL-NGS-116723 | 4 | 10 | 1.92% |
| 636 | chr27 | 8,130,825 | 8,211,737 | 80,913 | loss | BTA-105784-no-rs | BTA-112884-no-rs | 3 | 2 | Multiple |
| 637 | chr27 | 10,114,226 | 11,405,002 | 1,290,777 | both | BTB-01410000 | BTB-00950935 | 30 | 24 | 4.61% |
| 638 | chr27 | 11,666,482 | 11,745,676 | 79,195 | loss | BTA-63154-no-rs | Hapmap27590-BTA-139234 | 3 | 1 | Unique |
| 639 | chr27 | 13,250,583 | 13,464,247 | 213,665 | loss | BTB-00951788 | BTB-00951536 | 5 | 1 | Unique |
| 640 | chr27 | 17,397,248 | 17,547,965 | 150,718 | both | Hapmap61080-rs29013547 | BTB-00953338 | 6 | 2 | Multiple |
| 641 | chr27 | 30,234,591 | 30,292,434 | 57,844 | loss | BTB-00965793 | BTA-00436-rs29013593 | 3 | 1 | Unique |
| 642 | chr27 | 32,048,619 | 32,184,909 | 136,291 | loss | BFGL-NGS-116607 | ARS-BFGL-NGS-21155 | 3 | 7 | 1.34% |
| 643 | chr27 | 39,848,172 | 39,987,486 | 139,315 | gain | ARS-BFGL-NGS-92338 | DIAS-332 | 5 | 1 | Unique |
| 644 | chr27 | 41,653,993 | 41,820,989 | 166,997 | gain | DIAS-335 | DIAS-336 | 3 | 2 | Multiple |
| 645 | chr27 | 42,285,165 | 42,567,894 | 282,730 | gain | BTB-01682077 | BTB-00971533 | 4 | 2 | Multiple |
| 646 | chr27 | 42,827,795 | 42,987,259 | 159,465 | gain | ARS-BFGL-NGS-239 | Hapmap38844-BTA-63080 | 4 | 1 | Unique |
| 647 | chr28 | 1,327,903 | 1,401,069 | 73,167 | gain | ARS-BFGL-NGS-59257 | ARS-BFGL-NGS-107787 | 3 | 4 | 0.77% |
| 648 | chr28 | 9,580,455 | 10,073,352 | 492,898 | loss | Hapmap39009-BTA-121529 | BTB-00976622 | 7 | 4 | 0.77% |
| 649 | chr28 | 10,430,536 | 10,563,623 | 133,088 | loss | Hapmap55640-rs29014036 | Hapmap50192-BTA-104693 | 5 | 1 | Unique |
| 650 | chr28 | 19,938,671 | 20,141,448 | 202,778 | loss | BTB-02080610 | BTB-01171367 | 4 | 1 | Unique |
| 651 | chr28 | 20,375,994 | 20,520,824 | 144,831 | loss | BTB-01171634 | BTB-01522449 | 3 | 1 | Unique |
| 652 | chr28 | 20,898,742 | 21,258,397 | 359,656 | both | ARS-BFGL-NGS-36180 | BTB-01650236 | 10 | 5 | 0.96% |
| 653 | chr28 | 22,744,897 | 22,811,303 | 66,407 | loss | Hapmap27166-BTA-139945 | ARS-BFGL-NGS-351 | 4 | 3 | 0.58% |
| 654 | chr28 | 23,347,127 | 23,442,097 | 94,971 | gain | ARS-BFGL-NGS-87034 | ARS-BFGL-NGS-15201 | 3 | 2 | Multiple |
| 655 | chr28 | 24,148,146 | 24,327,772 | 179,627 | loss | BTA-63757-no-rs | Hapmap44654-BTA-63754 | 4 | 3 | 0.58% |
| 656 | chr28 | 24,436,312 | 24,628,489 | 192,178 | gain | Hapmap44554-BTA-63753 | ARS-BFGL-NGS-87782 | 5 | 9 | 1.73% |
| 657 | chr28 | 34,863,921 | 34,952,112 | 88,192 | loss | BTB-01693480 | BTB-01851415 | 3 | 3 | 0.58% |
| 658 | chr28 | 35,942,913 | 36,126,791 | 183,879 | loss | BTA-24338-no-rs | BTB-01469727 | 4 | 5 | 0.96% |
| 659 | chr28 | 37,383,796 | 37,494,090 | 110,295 | loss | BFGL-NGS-115768 | BTB-01713276 | 4 | 6 | 1.15% |
| 660 | chr28 | 45,481,307 | 46,020,951 | 539,645 | loss | ARS-BFGL-NGS-14462 | Hapmap43005-BTA-64606 | 13 | 2 | Multiple |
| 661 | chr29 | 238,770 | 332,901 | 94,132 | loss | Hapmap53898-rs29026969 | Hapmap58534-rs29018685 | 3 | 1 | Unique |
| 662 | chr29 | 5,405,323 | 5,540,557 | 135,235 | gain | BTB-00999163 | BTA-94746-no-rs | 4 | 1 | Unique |
| 663 | chr29 | 5,596,797 | 5,808,288 | 211,492 | gain | BTA-116348-no-rs | ARS-BFGL-NGS-32198 | 5 | 3 | 0.58% |
| 664 | chr29 | 16,025,428 | 16,334,339 | 308,912 | both | BTA-117784-no-rs | Hapmap42430-BTA-112191 | 5 | 3 | 0.58% |
| 665 | chr29 | 16,769,413 | 16,979,197 | 209,785 | loss | BFGL-NGS-115782 | BFGL-NGS-118102 | 4 | 4 | 0.77% |
| 666 | chr29 | 22,869,086 | 22,927,227 | 58,142 | loss | ARS-BFGL-NGS-18922 | Hapmap36817-SCAFFOLD245829_8774 | 3 | 1 | Unique |
| 667 | chr29 | 25,611,084 | 25,716,302 | 105,219 | loss | BFGL-NGS-119138 | Hapmap43319-BTA-65094 | 3 | 1 | Unique |
| 668 | chr29 | 27,814,492 | 27,909,466 | 94,975 | gain | ARS-BFGL-NGS-32195 | Hapmap39080-BTA-112330 | 3 | 2 | Multiple |
| 669 | chr29 | 27,969,811 | 28,119,274 | 149,464 | gain | BTB-01015252 | BTB-01016570 | 4 | 1 | Unique |
| 670 | chr29 | 28,417,195 | 29,211,966 | 794,772 | both | BTA-111991-no-rs | BFGL-NGS-114791 | 14 | 26 | 4.99% |
| 671 | chr29 | 30,016,239 | 30,132,989 | 116,751 | loss | BTB-01017180 | ARS-BFGL-NGS-79220 | 5 | 2 | Multiple |
| 672 | chr29 | 37,630,151 | 37,709,693 | 79,543 | gain | ARS-BFGL-NGS-45128 | ARS-BFGL-NGS-39172 | 3 | 1 | Unique |
| 673 | chr29 | 38,406,381 | 38,500,354 | 93,974 | gain | BTA-65647-no-rs | Hapmap54433-rs29022463 | 3 | 8 | 1.54% |
| 674 | chr29 | 40,070,110 | 40,186,222 | 116,113 | gain | ARS-BFGL-NGS-97760 | ARS-BFGL-NGS-22832 | 3 | 1 | Unique |
| 675 | chr29 | 40,672,416 | 40,748,813 | 76,398 | gain | ARS-BFGL-NGS-22514 | ARS-BFGL-NGS-23135 | 3 | 1 | Unique |
| 676 | chr29 | 44,132,149 | 44,277,693 | 145,545 | loss | ARS-BFGL-NGS-29002 | ARS-BFGL-NGS-7054 | 5 | 1 | Unique |
| 677 | chr29 | 45,391,413 | 45,488,027 | 96,615 | gain | ARS-BFGL-NGS-82048 | Hapmap36151-SCAFFOLD96691_12353 | 3 | 4 | 0.77% |
| 678 | chr29 | 47,861,402 | 47,955,033 | 93,632 | gain | BTB-01036181 | Hapmap47369-BTA-66337 | 4 | 1 | Unique |
| 679 | chr29 | 48,040,664 | 48,213,917 | 173,254 | loss | ARS-BFGL-NGS-65789 | ARS-BFGL-NGS-108161 | 6 | 1 | Unique |
| 680 | chr29 | 49,109,423 | 49,228,426 | 119,004 | both | ARS-BFGL-NGS-86466 | Hapmap24672-BTA-140771 | 5 | 6 | 1.15% |
| 681 | chr29 | 51,000,624 | 51,132,407 | 131,784 | gain | ARS-BFGL-NGS-102385 | ARS-BFGL-NGS-92419 | 4 | 2 | Multiple |
| 682 | chr29 | 51,301,849 | 51,979,343 | 677,495 | both | ARS-BFGL-NGS-29984 | ARS-BFGL-NGS-16118 | 18 | 9 | 1.73% |

**Table S3. Comparison of CNV regions identified on two cattle genome assemblies.**

| ***Statistics*** | ***Btau_4.0*** | ***UMD3*** |
| --- | --- | --- |
| **CNVR Count** | 682 | 743 |
| **Loss** | 370 | 404 |
| **Gain** | 216 | 230 |
| **Both** | 96 | 109 |
| **Total Length** | 139,786,166 | 158,022,355 |
| **Minimum Length** | 32,566 | 19,448 |
| **Maximum Length** | 5,569,091 | 5,433,882 |
| **Average Length** | 204,965 | 212,682 |
| **Standard Deviation** | 296,485 | 352,574 |
| **Median Length** | 131,179 | 128,906 |
| **Total SNP Number** | 3826 | 4376 |

**Table S4. UMD3 cattle CNV regions and their frequencies.**

| ***No*** | ***Chr*** | ***Start*** | ***End*** | ***Length*** | ***Type*** | ***StartSNP*** | ***EndSNP*** | ***No. SNP*** | ***Count*** | ***Frequency*** |
| --- | --- | --- | --- | --- | --- | --- | --- | --- | --- | --- |
| 1 | chr1 | 1,436,503 | 1,646,700 | 210,198 | gain | ARS-BFGL-NGS-62826 | ARS-BFGL-BAC-4980 | 7 | 3 | 0.58% |
| 2 | chr1 | 1,896,112 | 1,983,902 | 87,791 | gain | ARS-BFGL-NGS-25183 | ARS-BFGL-BAC-7317 | 4 | 1 | Unique |
| 3 | chr1 | 11,221,889 | 11,400,265 | 178,377 | loss | Hapmap43814-BTA-22629 | BTA-29334-no-rs | 5 | 1 | Unique |
| 4 | chr1 | 12,342,374 | 12,468,631 | 126,258 | loss | ARS-BFGL-BAC-14244 | Hapmap28058-BTA-161831 | 3 | 7 | 1.34% |
| 5 | chr1 | 14,025,660 | 14,102,864 | 77,205 | loss | ARS-BFGL-NGS-26402 | ARS-BFGL-BAC-14883 | 3 | 1 | Unique |
| 6 | chr1 | 14,551,165 | 14,961,941 | 410,777 | loss | BTA-28028-no-rs | BTB-01224765 | 11 | 2 | Multiple |
| 7 | chr1 | 16,997,662 | 17,073,972 | 76,311 | loss | BTA-118731-no-rs | BTB-01083468 | 4 | 1 | Unique |
| 8 | chr1 | 19,641,727 | 20,056,335 | 414,609 | both | BTA-39399-no-rs | BTA-39450-no-rs | 9 | 5 | 0.96% |
| 9 | chr1 | 20,165,566 | 20,213,558 | 47,993 | loss | Hapmap49594-BTA-39447 | ARS-BFGL-NGS-10334 | 3 | 3 | 0.58% |
| 10 | chr1 | 21,330,326 | 21,393,283 | 62,958 | loss | BTB-01210202 | BTB-01210076 | 3 | 1 | Unique |
| 11 | chr1 | 23,217,832 | 23,307,655 | 89,824 | loss | BTB-01153392 | BTB-01153241 | 3 | 2 | Multiple |
| 12 | chr1 | 26,782,245 | 26,966,818 | 184,574 | loss | Hapmap51561-BTA-120459 | Hapmap33501-BTA-152681 | 5 | 4 | 0.77% |
| 13 | chr1 | 29,273,705 | 29,336,218 | 62,514 | gain | BTB-00012627 | BFGL-NGS-119297 | 3 | 2 | Multiple |
| 14 | chr1 | 30,724,028 | 30,813,844 | 89,817 | loss | BTA-87372-no-rs | Hapmap32039-BTA-131798 | 3 | 1 | Unique |
| 15 | chr1 | 32,340,451 | 32,738,897 | 398,447 | loss | Hapmap52004-BTA-69178 | Hapmap58994-rs29021251 | 10 | 3 | 0.58% |
| 16 | chr1 | 32,961,952 | 33,036,107 | 74,156 | loss | BTA-69212-no-rs | BTB-00015939 | 3 | 1 | Unique |
| 17 | chr1 | 33,224,566 | 33,305,018 | 80,453 | loss | BTA-69229-no-rs | BTB-00901868 | 3 | 2 | Multiple |
| 18 | chr1 | 34,240,957 | 34,304,788 | 63,832 | loss | Hapmap43629-BTA-60810 | BTB-01211075 | 3 | 1 | Unique |
| 19 | chr1 | 35,604,177 | 35,867,269 | 263,093 | loss | DPI-2 | ARS-BFGL-BAC-7452 | 7 | 1 | Unique |
| 20 | chr1 | 36,874,545 | 37,117,597 | 243,053 | loss | Hapmap52575-rs29013932 | Hapmap46343-BTA-111768 | 8 | 1 | Unique |
| 21 | chr1 | 37,355,979 | 37,677,212 | 321,234 | loss | ARS-BFGL-BAC-14429 | ARS-BFGL-NGS-53463 | 7 | 3 | 0.58% |
| 22 | chr1 | 38,352,766 | 38,649,369 | 296,604 | gain | BTA-16749-no-rs | BTA-07775-no-rs | 6 | 7 | 1.34% |
| 23 | chr1 | 40,046,179 | 41,913,980 | 1,867,802 | loss | BTB-01249999 | ARS-BFGL-BAC-15157 | 51 | 6 | 1.15% |
| 24 | chr1 | 42,142,033 | 42,265,516 | 123,484 | loss | BTB-01708786 | BTB-01955018 | 4 | 2 | Multiple |
| 25 | chr1 | 44,215,211 | 44,272,642 | 57,432 | loss | BTB-00021257 | ARS-BFGL-NGS-81540 | 3 | 2 | Multiple |
| 26 | chr1 | 44,536,105 | 44,593,858 | 57,754 | gain | BTB-00075865 | BTA-15338-no-rs | 3 | 1 | Unique |
| 27 | chr1 | 46,201,490 | 46,251,987 | 50,498 | gain | BTB-01786029 | BTA-14361-rs29023125 | 3 | 1 | Unique |
| 28 | chr1 | 46,808,068 | 46,962,353 | 154,286 | loss | ARS-BFGL-NGS-69858 | BTB-01625181 | 3 | 2 | Multiple |
| 29 | chr1 | 48,347,264 | 48,523,202 | 175,939 | loss | ARS-BFGL-NGS-50906 | ARS-BFGL-NGS-41264 | 7 | 4 | 0.77% |
| 30 | chr1 | 48,726,549 | 48,971,016 | 244,468 | loss | BTB-01146938 | ARS-BFGL-NGS-49634 | 6 | 2 | Multiple |
| 31 | chr1 | 50,434,457 | 50,511,043 | 76,587 | loss | BTB-00022142 | BTB-01165109 | 3 | 1 | Unique |
| 32 | chr1 | 52,251,694 | 52,316,632 | 64,939 | loss | Hapmap23148-BTA-123974 | ARS-BFGL-NGS-69861 | 3 | 1 | Unique |
| 33 | chr1 | 59,409,838 | 59,463,782 | 53,945 | loss | ARS-USMARC-Parent-DQ404150-rs29012530 | ARS-BFGL-NGS-16908 | 4 | 1 | Unique |
| 34 | chr1 | 60,254,737 | 60,321,156 | 66,420 | loss | BTA-31643-no-rs | BTB-00028937 | 3 | 2 | Multiple |
| 35 | chr1 | 61,329,180 | 61,381,912 | 52,733 | loss | Hapmap41191-BTA-16674 | Hapmap59735-rs29016420 | 3 | 1 | Unique |
| 36 | chr1 | 71,504,767 | 71,623,173 | 118,407 | loss | Hapmap43841-BTA-34601 | BTA-34631-no-rs | 4 | 1 | Unique |
| 37 | chr1 | 71,932,575 | 71,990,165 | 57,591 | both | BTA-120754-no-rs | Hapmap51826-BTA-34625 | 3 | 3 | 0.58% |
| 38 | chr1 | 73,140,918 | 73,286,310 | 145,393 | gain | ARS-BFGL-NGS-59448 | ARS-BFGL-NGS-4162 | 4 | 7 | 1.34% |
| 39 | chr1 | 76,670,244 | 76,792,695 | 122,452 | loss | ARS-BFGL-NGS-54199 | ARS-BFGL-BAC-31482 | 4 | 1 | Unique |
| 40 | chr1 | 82,680,131 | 82,787,221 | 107,091 | loss | ARS-BFGL-NGS-19217 | BTB-01059241 | 4 | 1 | Unique |
| 41 | chr1 | 89,358,747 | 89,431,170 | 72,424 | loss | BTB-01196514 | Hapmap27729-BTA-108726 | 3 | 2 | Multiple |
| 42 | chr1 | 92,090,517 | 92,648,727 | 558,211 | loss | Hapmap34097-BES3_Contig167_336 | Hapmap52705-rs29019397 | 15 | 1 | Unique |
| 43 | chr1 | 92,856,829 | 93,279,488 | 422,660 | both | BTA-17925-no-rs | BTA-07846-rs29027508 | 8 | 18 | 3.45% |
| 44 | chr1 | 94,053,202 | 94,367,295 | 314,094 | both | ARS-BFGL-BAC-36882 | BTB-00768631 | 9 | 14 | 2.69% |
| 45 | chr1 | 94,512,100 | 94,562,544 | 50,445 | loss | BTB-01087150 | BTA-85695-no-rs | 3 | 2 | Multiple |
| 46 | chr1 | 94,699,308 | 94,833,263 | 133,956 | loss | ARS-BFGL-NGS-104582 | BTB-01793064 | 4 | 1 | Unique |
| 47 | chr1 | 101,664,632 | 101,816,107 | 151,476 | loss | BTB-01136854 | BTB-01136812 | 5 | 2 | Multiple |
| 48 | chr1 | 102,056,710 | 102,249,245 | 192,536 | loss | BTB-01136347 | Hapmap28099-BTA-150614 | 3 | 1 | Unique |
| 49 | chr1 | 103,618,079 | 103,973,956 | 355,878 | loss | BTA-39419-no-rs | Hapmap38948-BTA-28757 | 12 | 5 | 0.96% |
| 50 | chr1 | 104,842,361 | 104,926,538 | 84,178 | loss | BTB-01331013 | BTB-01613887 | 3 | 1 | Unique |
| 51 | chr1 | 105,053,285 | 105,264,358 | 211,074 | loss | BTB-02031540 | BTB-02073725 | 9 | 11 | 2.11% |
| 52 | chr1 | 106,228,836 | 106,365,182 | 136,347 | loss | BTB-01808842 | Hapmap54763-rs29012506 | 6 | 1 | Unique |
| 53 | chr1 | 112,225,759 | 112,273,665 | 47,907 | loss | ARS-BFGL-BAC-6549 | Hapmap43087-BTA-87783 | 3 | 2 | Multiple |
| 54 | chr1 | 112,729,281 | 112,797,583 | 68,303 | loss | BTB-00738261 | DIAS-1 | 3 | 3 | 0.58% |
| 55 | chr1 | 113,417,379 | 113,631,332 | 213,954 | loss | Hapmap61008-rs29023119 | Hapmap42001-BTA-89523 | 3 | 2 | Multiple |
| 56 | chr1 | 115,291,574 | 115,420,903 | 129,330 | gain | BTB-00050559 | BTA-12381-rs29024334 | 3 | 2 | Multiple |
| 57 | chr1 | 120,787,195 | 120,874,289 | 87,095 | gain | BTA-22577-no-rs | ARS-BFGL-NGS-64650 | 4 | 2 | Multiple |
| 58 | chr1 | 122,057,632 | 122,360,054 | 302,423 | both | BTB-01793232 | Hapmap43795-BTA-16918 | 7 | 7 | 1.34% |
| 59 | chr1 | 123,646,279 | 125,188,443 | 1,542,165 | both | ARS-BFGL-BAC-14851 | BTB-01404702 | 30 | 10 | 1.92% |
| 60 | chr1 | 126,606,801 | 126,680,933 | 74,133 | loss | BFGL-NGS-113021 | ARS-BFGL-NGS-100109 | 3 | 1 | Unique |
| 61 | chr1 | 127,458,995 | 127,841,330 | 382,336 | loss | BTA-96197-no-rs | BTB-01663737 | 9 | 3 | 0.58% |
| 62 | chr1 | 129,743,120 | 129,801,503 | 58,384 | gain | Hapmap39600-BTA-89434 | BFGL-NGS-116159 | 3 | 3 | 0.58% |
| 63 | chr1 | 133,753,152 | 133,850,518 | 97,367 | loss | BTB-01215047 | BTB-01215148 | 4 | 2 | Multiple |
| 64 | chr1 | 138,816,095 | 138,861,667 | 45,573 | loss | ARS-BFGL-NGS-27857 | BTA-26323-no-rs | 3 | 1 | Unique |
| 65 | chr1 | 140,685,557 | 140,835,240 | 149,684 | gain | ARS-BFGL-NGS-14502 | Hapmap51743-BTA-54330 | 4 | 1 | Unique |
| 66 | chr1 | 148,123,790 | 148,194,534 | 70,745 | gain | ARS-BFGL-NGS-21137 | ARS-BFGL-NGS-46522 | 3 | 1 | Unique |
| 67 | chr1 | 151,991,418 | 152,245,009 | 253,592 | gain | BTA-16239-no-rs | BTA-57354-no-rs | 7 | 3 | 0.58% |
| 68 | chr1 | 155,866,844 | 155,955,828 | 88,985 | loss | BTB-00023885 | BTB-00023937 | 3 | 1 | Unique |
| 69 | chr2 | 1,316,010 | 1,366,643 | 50,634 | loss | ARS-BFGL-NGS-39059 | Hapmap58923-rs29010138 | 3 | 2 | Multiple |
| 70 | chr2 | 4,314,286 | 4,399,808 | 85,523 | gain | BTB-00078262 | ARS-BFGL-NGS-105116 | 3 | 4 | 0.77% |
| 71 | chr2 | 8,384,570 | 8,430,514 | 45,945 | gain | ARS-BFGL-NGS-65047 | Hapmap40313-BTA-27938 | 3 | 2 | Multiple |
| 72 | chr2 | 8,788,219 | 9,113,368 | 325,150 | loss | Hapmap50551-BTA-104382 | BTB-00079257 | 11 | 3 | 0.58% |
| 73 | chr2 | 12,138,830 | 12,277,461 | 138,632 | loss | Hapmap44546-BTA-48673 | Hapmap46046-BTA-114001 | 3 | 1 | Unique |
| 74 | chr2 | 13,709,935 | 13,800,470 | 90,536 | loss | Hapmap39412-BTA-48779 | ARS-BFGL-NGS-38281 | 4 | 2 | Multiple |
| 75 | chr2 | 14,428,308 | 14,662,015 | 233,708 | both | BTB-01144233 | Hapmap33059-BTA-158581 | 6 | 4 | 0.77% |
| 76 | chr2 | 15,385,210 | 15,482,715 | 97,506 | gain | BTB-01084313 | ARS-BFGL-BAC-29210 | 3 | 2 | Multiple |
| 77 | chr2 | 18,078,290 | 18,318,018 | 239,729 | both | BTB-00081145 | Hapmap30496-BTA-123052 | 9 | 3 | 0.58% |
| 78 | chr2 | 27,412,491 | 28,073,362 | 660,872 | gain | BTB-00091195 | ARS-BFGL-NGS-40971 | 22 | 6 | 1.15% |
| 79 | chr2 | 28,855,504 | 28,923,176 | 67,673 | loss | ARS-BFGL-BAC-30043 | BTB-01494156 | 4 | 1 | Unique |
| 80 | chr2 | 31,569,746 | 31,671,085 | 101,340 | gain | Hapmap52594-rs29026993 | BTB-01571963 | 4 | 2 | Multiple |
| 81 | chr2 | 41,786,376 | 41,938,704 | 152,329 | loss | Hapmap48777-BTA-47434 | Hapmap46653-BTA-47447 | 7 | 1 | Unique |
| 82 | chr2 | 52,289,749 | 52,417,495 | 127,747 | gain | ARS-BFGL-NGS-59320 | BTA-47656-no-rs | 5 | 1 | Unique |
| 83 | chr2 | 54,487,230 | 54,519,795 | 32,566 | loss | BTA-122373-no-rs | BTB-01775964 | 3 | 1 | Unique |
| 84 | chr2 | 56,063,430 | 56,234,794 | 171,365 | loss | Hapmap27618-BTA-152634 | BTA-101352-no-rs | 5 | 3 | 0.58% |
| 85 | chr2 | 56,583,793 | 56,636,575 | 52,783 | loss | BTB-01588936 | ARS-BFGL-NGS-43395 | 3 | 1 | Unique |
| 86 | chr2 | 57,260,012 | 57,373,897 | 113,886 | loss | BTA-19224-no-rs | BTB-00099884 | 4 | 20 | 3.84% |
| 87 | chr2 | 63,900,374 | 64,065,171 | 164,798 | gain | ARS-BFGL-NGS-42334 | ARS-BFGL-NGS-94 | 5 | 8 | 1.54% |
| 88 | chr2 | 67,484,628 | 67,575,238 | 90,611 | loss | BTB-01689724 | ARS-BFGL-NGS-38034 | 3 | 1 | Unique |
| 89 | chr2 | 67,813,507 | 67,971,113 | 157,607 | loss | BTB-01654053 | BTB-01297078 | 5 | 2 | Multiple |
| 90 | chr2 | 74,548,518 | 74,805,654 | 257,137 | loss | ARS-BFGL-NGS-104166 | Hapmap43713-BTA-86767 | 3 | 2 | Multiple |
| 91 | chr2 | 77,832,115 | 77,900,295 | 68,181 | loss | BTA-98940-no-rs | BTA-98942-no-rs | 3 | 1 | Unique |
| 92 | chr2 | 78,433,731 | 78,604,521 | 170,791 | loss | BTB-01767855 | BTB-00103489 | 7 | 2 | Multiple |
| 93 | chr2 | 81,721,478 | 81,878,865 | 157,388 | loss | Hapmap51700-BTA-21117 | BTA-105824-no-rs | 3 | 2 | Multiple |
| 94 | chr2 | 87,843,486 | 88,162,563 | 319,078 | gain | ARS-BFGL-NGS-91673 | BFGL-NGS-119235 | 4 | 2 | Multiple |
| 95 | chr2 | 93,094,119 | 93,172,942 | 78,824 | loss | DIAS-311 | Hapmap33390-BTA-157206 | 3 | 1 | Unique |
| 96 | chr2 | 103,421,027 | 103,491,995 | 70,969 | loss | ARS-BFGL-NGS-96747 | Hapmap49191-BTA-48538 | 3 | 1 | Unique |
| 97 | chr2 | 104,406,422 | 104,800,752 | 394,331 | loss | ARS-BFGL-NGS-103574 | BFGL-NGS-117120 | 9 | 1 | Unique |
| 98 | chr2 | 115,157,873 | 115,264,249 | 106,377 | loss | Hapmap27370-BTA-123236 | Hapmap49461-BTA-115001 | 4 | 2 | Multiple |
| 99 | chr2 | 120,820,417 | 121,009,297 | 188,881 | both | INRA-102 | Hapmap38678-BTA-49219 | 7 | 8 | 1.54% |
| 100 | chr2 | 122,608,751 | 122,680,438 | 71,688 | gain | BFGL-NGS-119638 | BTA-49594-no-rs | 3 | 2 | Multiple |
| 101 | chr2 | 125,677,712 | 125,772,675 | 94,964 | loss | BFGL-NGS-117131 | BFGL-NGS-116217 | 3 | 2 | Multiple |
| 102 | chr2 | 126,926,945 | 126,957,475 | 30,531 | loss | ARS-BFGL-BAC-32408 | BFGL-NGS-118505 | 3 | 10 | 1.92% |
| 103 | chr2 | 127,922,394 | 128,081,087 | 158,694 | loss | BFGL-NGS-114532 | ARS-BFGL-NGS-80059 | 3 | 1 | Unique |
| 104 | chr2 | 133,599,634 | 133,979,803 | 380,170 | gain | BFGL-NGS-110390 | ARS-BFGL-NGS-66860 | 14 | 1 | Unique |
| 105 | chr2 | 134,824,616 | 134,964,271 | 139,656 | loss | ARS-BFGL-NGS-14190 | ARS-BFGL-NGS-17681 | 5 | 3 | 0.58% |
| 106 | chr2 | 135,011,539 | 135,580,681 | 569,143 | both | BTA-52274-no-rs | ARS-BFGL-NGS-27654 | 21 | 19 | 3.65% |
| 107 | chr2 | 136,697,236 | 137,037,714 | 340,479 | both | BFGL-NGS-117203 | ARS-BFGL-NGS-67782 | 5 | 14 | 2.69% |
| 108 | chr3 | 16,131 | 259,213 | 243,083 | gain | BTB-01688323 | Hapmap42163-BTA-27347 | 5 | 5 | 0.96% |
| 109 | chr3 | 1,843,353 | 1,937,626 | 94,274 | loss | ARS-BFGL-NGS-15921 | ARS-BFGL-NGS-654 | 3 | 1 | Unique |
| 110 | chr3 | 4,741,110 | 4,828,827 | 87,718 | loss | Hapmap39882-BTA-106655 | ARS-BFGL-NGS-7652 | 3 | 1 | Unique |
| 111 | chr3 | 5,022,182 | 5,306,081 | 283,900 | loss | ARS-BFGL-NGS-50158 | BTB-01234863 | 5 | 2 | Multiple |
| 112 | chr3 | 11,937,022 | 12,288,724 | 351,703 | gain | ARS-BFGL-NGS-25865 | Hapmap56299-rs29010540 | 7 | 8 | 1.54% |
| 113 | chr3 | 13,261,355 | 13,418,182 | 156,828 | gain | ARS-BFGL-NGS-106179 | ARS-BFGL-NGS-3037 | 4 | 1 | Unique |
| 114 | chr3 | 17,086,048 | 17,123,706 | 37,659 | gain | BTA-66676-no-rs | BFGL-NGS-111960 | 3 | 2 | Multiple |
| 115 | chr3 | 17,681,439 | 17,796,934 | 115,496 | gain | BTA-121572-no-rs | INRA-468 | 3 | 1 | Unique |
| 116 | chr3 | 18,247,150 | 18,320,912 | 73,763 | gain | ARS-BFGL-NGS-106077 | ARS-BFGL-NGS-92168 | 3 | 2 | Multiple |
| 117 | chr3 | 22,115,328 | 22,203,610 | 88,283 | loss | BFGL-NGS-117867 | BTB-00123279 | 4 | 1 | Unique |
| 118 | chr3 | 22,326,368 | 22,487,111 | 160,744 | loss | Hapmap60947-rs29019824 | INRA-257 | 3 | 2 | Multiple |
| 119 | chr3 | 22,740,940 | 22,975,725 | 234,786 | both | BTA-93841-no-rs | Hapmap26676-BTA-141146 | 8 | 30 | 5.76% |
| 120 | chr3 | 39,097,420 | 39,143,931 | 46,512 | loss | Hapmap58660-rs29011628 | BTA-93899-no-rs | 3 | 3 | 0.58% |
| 121 | chr3 | 39,360,570 | 39,456,939 | 96,370 | gain | BTB-01737920 | BTB-01155279 | 3 | 2 | Multiple |
| 122 | chr3 | 39,494,091 | 39,543,882 | 49,792 | both | BTB-01155190 | BTB-01155098 | 3 | 5 | 0.96% |
| 123 | chr3 | 40,443,176 | 41,432,313 | 989,138 | loss | BTB-01405443 | Hapmap33718-BTA-152247 | 19 | 5 | 0.96% |
| 124 | chr3 | 46,842,537 | 47,005,201 | 162,665 | loss | BTB-01463390 | Hapmap27022-BTA-159739 | 6 | 4 | 0.77% |
| 125 | chr3 | 48,254,392 | 48,392,443 | 138,052 | loss | Hapmap32815-BTA-141391 | BTB-00127606 | 5 | 3 | 0.58% |
| 126 | chr3 | 54,314,529 | 55,074,485 | 759,957 | gain | INRA-240 | ARS-BFGL-NGS-83033 | 16 | 120 | 23.03% |
| 127 | chr3 | 60,388,539 | 60,465,697 | 77,159 | loss | BTB-01323076 | BTB-01322824 | 4 | 1 | Unique |
| 128 | chr3 | 62,035,181 | 62,145,904 | 110,724 | loss | BTB-02017777 | BTB-01210458 | 4 | 1 | Unique |
| 129 | chr3 | 64,132,985 | 64,186,622 | 53,638 | loss | BTA-68158-no-rs | BTB-00131667 | 4 | 1 | Unique |
| 130 | chr3 | 64,724,037 | 64,944,718 | 220,682 | loss | Hapmap52640-rs29027369 | BTA-118190-no-rs | 5 | 1 | Unique |
| 131 | chr3 | 71,238,926 | 71,383,017 | 144,092 | loss | BTB-01293614 | BTA-87963-no-rs | 5 | 1 | Unique |
| 132 | chr3 | 72,183,926 | 72,296,340 | 112,415 | gain | BTA-114873-no-rs | Hapmap54050-rs29013285 | 3 | 1 | Unique |
| 133 | chr3 | 76,775,255 | 76,870,934 | 95,680 | loss | BTA-13007-rs29017974 | BTB-01789933 | 6 | 1 | Unique |
| 134 | chr3 | 93,936,476 | 94,058,556 | 122,081 | both | BTB-01604189 | ARS-BFGL-NGS-14376 | 5 | 10 | 1.92% |
| 135 | chr3 | 98,329,782 | 98,632,548 | 302,767 | loss | BTB-00151846 | BTB-01546439 | 9 | 1 | Unique |
| 136 | chr3 | 112,073,191 | 112,160,436 | 87,246 | loss | BTA-69557-no-rs | Hapmap30841-BTA-147966 | 4 | 2 | Multiple |
| 137 | chr3 | 115,790,125 | 115,888,900 | 98,776 | loss | Hapmap39506-BTA-69666 | ARS-BFGL-NGS-42740 | 3 | 1 | Unique |
| 138 | chr3 | 116,781,408 | 116,821,782 | 40,375 | loss | BTB-00719198 | ARS-BFGL-NGS-15642 | 3 | 1 | Unique |
| 139 | chr3 | 119,704,501 | 119,747,335 | 42,835 | gain | ARS-BFGL-NGS-67852 | ARS-BFGL-NGS-17137 | 3 | 2 | Multiple |
| 140 | chr3 | 120,220,712 | 120,547,501 | 326,790 | loss | ARS-BFGL-NGS-35805 | ARS-BFGL-NGS-103152 | 6 | 2 | Multiple |
| 141 | chr3 | 120,737,342 | 121,157,967 | 420,626 | loss | ARS-BFGL-NGS-92456 | ARS-BFGL-NGS-107367 | 13 | 1 | Unique |
| 142 | chr4 | 6,209,229 | 6,291,498 | 82,270 | gain | Hapmap28904-BTA-120019 | ARS-BFGL-NGS-69941 | 3 | 1 | Unique |
| 143 | chr4 | 9,152,279 | 9,249,035 | 96,757 | loss | BTA-96426-no-rs | BTB-00165658 | 3 | 1 | Unique |
| 144 | chr4 | 10,235,907 | 10,499,057 | 263,151 | both | ARS-BFGL-NGS-64636 | Hapmap40298-BTA-109122 | 6 | 3 | 0.58% |
| 145 | chr4 | 16,452,962 | 16,554,797 | 101,836 | loss | BTB-01176772 | BTA-68467-no-rs | 3 | 2 | Multiple |
| 146 | chr4 | 19,510,899 | 19,715,038 | 204,140 | loss | BTB-01108314 | ARS-BFGL-NGS-108298 | 3 | 2 | Multiple |
| 147 | chr4 | 20,663,663 | 20,693,421 | 29,759 | gain | BFGL-NGS-117041 | BTA-99534-no-rs | 3 | 1 | Unique |
| 148 | chr4 | 21,248,954 | 21,374,651 | 125,698 | gain | BTA-38554-no-rs | BTA-93539-no-rs | 4 | 7 | 1.34% |
| 149 | chr4 | 22,667,688 | 22,939,067 | 271,380 | loss | BTB-01581687 | Hapmap50909-BTA-17901 | 7 | 1 | Unique |
| 150 | chr4 | 24,066,704 | 24,209,956 | 143,253 | loss | ARS-BFGL-NGS-40507 | BTB-00169573 | 5 | 3 | 0.58% |
| 151 | chr4 | 24,436,431 | 24,589,403 | 152,973 | loss | BTB-01768855 | BTB-01912042 | 6 | 1 | Unique |
| 152 | chr4 | 30,068,687 | 30,200,987 | 132,301 | loss | Hapmap44276-BTA-69937 | Hapmap54229-rs29017613 | 3 | 1 | Unique |
| 153 | chr4 | 37,341,832 | 37,409,461 | 67,630 | loss | Hapmap33483-BTA-142021 | BTB-00175546 | 3 | 1 | Unique |
| 154 | chr4 | 39,351,027 | 39,634,823 | 283,797 | gain | BTA-21017-no-rs | Hapmap50762-BTA-70080 | 8 | 2 | Multiple |
| 155 | chr4 | 41,444,423 | 41,583,591 | 139,169 | both | BTB-01922107 | BTB-00178966 | 5 | 2 | Multiple |
| 156 | chr4 | 42,925,876 | 43,121,671 | 195,796 | gain | ARS-BFGL-NGS-97154 | BFGL-NGS-116726 | 3 | 1 | Unique |
| 157 | chr4 | 48,649,499 | 48,819,329 | 169,831 | both | Hapmap46158-BTA-70371 | BTB-00181018 | 5 | 2 | Multiple |
| 158 | chr4 | 54,930,726 | 55,081,785 | 151,060 | loss | BTB-01298953 | BTB-00186521 | 5 | 1 | Unique |
| 159 | chr4 | 66,559,450 | 66,854,427 | 294,978 | gain | BTA-13086-rs29018051 | Hapmap57289-ss46526668 | 9 | 2 | Multiple |
| 160 | chr4 | 69,220,543 | 69,339,515 | 118,973 | gain | Hapmap38430-BTA-71131 | ARS-BFGL-NGS-97660 | 5 | 2 | Multiple |
| 161 | chr4 | 73,699,663 | 73,758,088 | 58,426 | loss | BTB-00196932 | Hapmap44399-BTA-71366 | 3 | 6 | 1.15% |
| 162 | chr4 | 74,975,523 | 75,115,247 | 139,725 | gain | BTB-00198166 | ARS-BFGL-NGS-59926 | 7 | 21 | 4.03% |
| 163 | chr4 | 83,294,785 | 83,480,309 | 185,525 | both | BTB-02092948 | ARS-BFGL-NGS-5222 | 6 | 14 | 2.69% |
| 164 | chr4 | 83,779,701 | 84,274,238 | 494,538 | loss | BTB-01857387 | BTB-01143040 | 14 | 1 | Unique |
| 165 | chr4 | 84,872,989 | 85,092,977 | 219,989 | loss | BTA-16834-no-rs | BTB-01278734 | 6 | 1 | Unique |
| 166 | chr4 | 85,635,270 | 85,904,999 | 269,730 | gain | UA-IFASA-181 | Hapmap39905-BTA-27381 | 8 | 8 | 1.54% |
| 167 | chr4 | 86,811,243 | 86,925,976 | 114,734 | gain | BTB-01032530 | ARS-BFGL-NGS-33395 | 4 | 1 | Unique |
| 168 | chr4 | 89,850,655 | 89,921,990 | 71,336 | loss | BTB-01759027 | BTA-122943-no-rs | 3 | 2 | Multiple |
| 169 | chr4 | 90,192,806 | 90,426,809 | 234,004 | both | Hapmap25897-BTA-158225 | BTB-01901306 | 9 | 4 | 0.77% |
| 170 | chr4 | 92,436,001 | 92,518,922 | 82,922 | loss | BTB-00202180 | BTB-00202224 | 3 | 5 | 0.96% |
| 171 | chr4 | 95,069,722 | 95,110,823 | 41,102 | gain | UA-IFASA-7564 | ARS-BFGL-NGS-9433 | 3 | 2 | Multiple |
| 172 | chr4 | 95,969,034 | 96,056,187 | 87,154 | gain | BTB-01298816 | ARS-BFGL-NGS-11916 | 3 | 2 | Multiple |
| 173 | chr4 | 106,484,083 | 106,758,862 | 274,780 | gain | ARS-BFGL-NGS-78121 | BTB-02023900 | 5 | 13 | 2.50% |
| 174 | chr4 | 108,168,742 | 108,313,356 | 144,615 | loss | BTA-72108-no-rs | BTA-72199-no-rs | 4 | 4 | 0.77% |
| 175 | chr4 | 108,786,955 | 108,867,683 | 80,729 | loss | ARS-BFGL-NGS-73118 | BFGL-NGS-119262 | 3 | 1 | Unique |
| 176 | chr4 | 112,139,960 | 112,184,024 | 44,065 | loss | BTB-01163185 | ARS-BFGL-NGS-33889 | 3 | 1 | Unique |
| 177 | chr4 | 113,662,920 | 114,140,242 | 477,323 | gain | ARS-BFGL-NGS-74214 | ARS-BFGL-NGS-1225 | 14 | 94 | 18.04% |
| 178 | chr4 | 114,688,732 | 114,744,308 | 55,577 | loss | BTB-00213370 | ARS-BFGL-NGS-100194 | 3 | 2 | Multiple |
| 179 | chr4 | 116,013,931 | 116,083,500 | 69,570 | loss | ARS-BFGL-NGS-5125 | Hapmap57046-rs29017141 | 3 | 6 | 1.15% |
| 180 | chr4 | 118,608,842 | 118,655,986 | 47,145 | gain | Hapmap35657-SCAFFOLD15929_9264 | ARS-BFGL-NGS-105018 | 3 | 1 | Unique |
| 181 | chr5 | 3,260,057 | 3,645,270 | 385,214 | loss | Hapmap44365-BTA-28169 | Hapmap55203-rs29023737 | 11 | 8 | 1.54% |
| 182 | chr5 | 3,740,223 | 3,823,446 | 83,224 | gain | ARS-BFGL-NGS-27281 | Hapmap48402-BTA-60008 | 3 | 1 | Unique |
| 183 | chr5 | 4,309,575 | 4,677,157 | 367,583 | gain | Hapmap39974-BTA-119920 | BTB-01486702 | 12 | 2 | Multiple |
| 184 | chr5 | 6,266,261 | 6,357,367 | 91,107 | gain | Hapmap59616-ss46526976 | ARS-BFGL-NGS-8671 | 3 | 2 | Multiple |
| 185 | chr5 | 8,893,080 | 8,949,076 | 55,997 | loss | BTB-00218987 | BTB-00219084 | 3 | 1 | Unique |
| 186 | chr5 | 12,601,297 | 12,660,434 | 59,138 | loss | BTA-75576-no-rs | DIAS-102 | 3 | 1 | Unique |
| 187 | chr5 | 14,770,370 | 14,953,446 | 183,077 | loss | BTA-87039-no-rs | Hapmap44154-BTA-87046 | 6 | 2 | Multiple |
| 188 | chr5 | 20,118,511 | 20,218,000 | 99,490 | gain | BTA-20092-no-rs | BFGL-NGS-114197 | 3 | 2 | Multiple |
| 189 | chr5 | 21,181,621 | 21,258,465 | 76,845 | gain | BTA-121676-no-rs | ARS-BFGL-NGS-7668 | 3 | 1 | Unique |
| 190 | chr5 | 30,562,466 | 30,618,036 | 55,571 | loss | Hapmap38536-BTA-85959 | BTA-73280-no-rs | 3 | 2 | Multiple |
| 191 | chr5 | 40,868,010 | 41,025,310 | 157,301 | gain | Hapmap44326-BTA-97746 | ARS-BFGL-NGS-19252 | 6 | 4 | 0.77% |
| 192 | chr5 | 42,656,382 | 42,720,718 | 64,337 | loss | BTA-119080-no-rs | BTB-01255957 | 3 | 4 | 0.77% |
| 193 | chr5 | 43,736,571 | 43,799,282 | 62,712 | loss | BTB-00227037 | Hapmap51299-BTA-73473 | 3 | 2 | Multiple |
| 194 | chr5 | 51,252,309 | 51,392,175 | 139,867 | loss | BTA-73549-no-rs | Hapmap34504-BES10_Contig662_1293 | 4 | 2 | Multiple |
| 195 | chr5 | 56,279,114 | 56,410,718 | 131,605 | both | ARS-BFGL-NGS-100975 | Hapmap44616-BTA-73618 | 3 | 5 | 0.96% |
| 196 | chr5 | 58,812,576 | 59,598,727 | 786,152 | both | BTA-87618-no-rs | Hapmap26488-BTA-164056 | 11 | 92 | 17.66% |
| 197 | chr5 | 59,840,330 | 60,120,730 | 280,401 | gain | ARS-BFGL-NGS-44413 | BTB-01920291 | 5 | 7 | 1.34% |
| 198 | chr5 | 76,501,658 | 77,211,408 | 709,751 | loss | Hapmap24162-BTA-160429 | ARS-BFGL-NGS-27335 | 23 | 4 | 0.77% |
| 199 | chr5 | 78,267,464 | 78,385,143 | 117,680 | gain | Hapmap52101-rs29018073 | BTA-104619-no-rs | 3 | 2 | Multiple |
| 200 | chr5 | 86,997,963 | 87,104,035 | 106,073 | loss | BTA-98442-no-rs | ARS-BFGL-NGS-73390 | 4 | 1 | Unique |
| 201 | chr5 | 90,376,600 | 90,445,432 | 68,833 | gain | Hapmap42271-BTA-61859 | ARS-BFGL-NGS-58101 | 3 | 2 | Multiple |
| 202 | chr5 | 98,915,804 | 99,044,605 | 128,802 | gain | BTA-114179-no-rs | Hapmap47185-BTA-114173 | 4 | 1 | Unique |
| 203 | chr5 | 99,527,745 | 99,702,501 | 174,757 | both | ARS-BFGL-NGS-108996 | BTB-02009804 | 4 | 5 | 0.96% |
| 204 | chr5 | 102,308,562 | 102,631,651 | 323,090 | both | Hapmap3063-BTA-15439 | BTB-01912130 | 9 | 124 | 23.80% |
| 205 | chr5 | 103,035,242 | 103,659,884 | 624,643 | both | ARS-BFGL-NGS-39840 | ARS-BFGL-NGS-30033 | 12 | 16 | 3.07% |
| 206 | chr5 | 112,494,968 | 112,591,306 | 96,339 | loss | ARS-BFGL-NGS-10616 | ARS-BFGL-NGS-102035 | 4 | 1 | Unique |
| 207 | chr5 | 113,611,955 | 113,737,833 | 125,879 | loss | BFGL-NGS-119376 | ARS-BFGL-NGS-31332 | 3 | 2 | Multiple |
| 208 | chr5 | 115,507,553 | 115,561,004 | 53,452 | gain | ARS-BFGL-NGS-38686 | ARS-BFGL-NGS-100195 | 3 | 1 | Unique |
| 209 | chr5 | 116,073,984 | 116,134,789 | 60,806 | loss | ARS-BFGL-NGS-11640 | ARS-BFGL-NGS-64268 | 3 | 2 | Multiple |
| 210 | chr5 | 116,895,329 | 117,217,354 | 322,026 | loss | ARS-BFGL-NGS-109232 | ARS-BFGL-NGS-41090 | 10 | 1 | Unique |
| 211 | chr5 | 117,769,309 | 118,014,757 | 245,449 | both | ARS-BFGL-NGS-36365 | ARS-BFGL-NGS-1089 | 10 | 2 | Multiple |
| 212 | chr5 | 118,960,375 | 119,071,205 | 110,831 | loss | Hapmap42757-BTA-99810 | ARS-BFGL-NGS-13216 | 5 | 1 | Unique |
| 213 | chr5 | 119,256,124 | 119,325,698 | 69,575 | loss | BFGL-NGS-109805 | Hapmap47809-BTA-102708 | 3 | 2 | Multiple |
| 214 | chr5 | 119,795,140 | 119,853,322 | 58,183 | both | ARS-BFGL-NGS-1995 | ARS-BFGL-NGS-56909 | 3 | 4 | 0.77% |
| 215 | chr6 | 138,563 | 939,016 | 800,454 | both | BTB-01518631 | BTB-01796690 | 21 | 17 | 3.26% |
| 216 | chr6 | 3,316,210 | 3,404,563 | 88,354 | loss | Hapmap53408-rs29014449 | BTB-00241116 | 3 | 1 | Unique |
| 217 | chr6 | 6,923,857 | 7,101,152 | 177,296 | both | ARS-BFGL-NGS-26083 | BFGL-NGS-117412 | 6 | 6 | 1.15% |
| 218 | chr6 | 8,478,503 | 8,595,245 | 116,743 | loss | Hapmap50460-BTA-75791 | BTA-75766-no-rs | 3 | 1 | Unique |
| 219 | chr6 | 8,953,238 | 9,340,204 | 386,967 | loss | BTA-104539-no-rs | BTB-01704614 | 9 | 3 | 0.58% |
| 220 | chr6 | 9,524,226 | 9,633,428 | 109,203 | loss | BTB-01068781 | BTA-104961-no-rs | 4 | 1 | Unique |
| 221 | chr6 | 9,914,189 | 10,295,237 | 381,049 | both | Hapmap33379-BTA-153882 | BTB-01069210 | 10 | 6 | 1.15% |
| 222 | chr6 | 10,609,153 | 11,053,007 | 443,855 | loss | BTB-01322092 | BTA-27165-no-rs | 14 | 2 | Multiple |
| 223 | chr6 | 11,447,723 | 11,746,541 | 298,819 | loss | ARS-BFGL-NGS-38131 | Hapmap40034-BTA-93974 | 8 | 1 | Unique |
| 224 | chr6 | 12,282,881 | 12,383,455 | 100,575 | loss | BTB-01685239 | Hapmap23501-BTA-155913 | 4 | 1 | Unique |
| 225 | chr6 | 12,447,020 | 12,558,447 | 111,428 | gain | BTA-112689-no-rs | BTA-88018-no-rs | 4 | 3 | 0.58% |
| 226 | chr6 | 23,134,761 | 23,217,855 | 83,095 | gain | BTB-00235875 | BTB-00246639 | 4 | 1 | Unique |
| 227 | chr6 | 24,398,925 | 24,495,145 | 96,221 | loss | BTA-94484-no-rs | BTB-01673078 | 4 | 1 | Unique |
| 228 | chr6 | 31,241,066 | 31,353,827 | 112,762 | loss | ARS-BFGL-NGS-11498 | Hapmap49740-BTA-75691 | 4 | 1 | Unique |
| 229 | chr6 | 35,360,248 | 35,524,123 | 163,876 | loss | Hapmap60855-rs29024248 | Hapmap43447-BTA-105138 | 3 | 2 | Multiple |
| 230 | chr6 | 38,845,992 | 38,939,012 | 93,021 | loss | Hapmap23507-BTC-041133 | Hapmap33628-BTC-041023 | 4 | 2 | Multiple |
| 231 | chr6 | 41,530,548 | 41,611,777 | 81,230 | loss | BTB-00252870 | Hapmap24317-BTC-037593 | 4 | 1 | Unique |
| 232 | chr6 | 43,037,439 | 43,153,620 | 116,182 | loss | BTA-95818-no-rs | BTB-01468543 | 5 | 9 | 1.73% |
| 233 | chr6 | 48,087,895 | 48,216,479 | 128,585 | gain | ARS-BFGL-NGS-73994 | ARS-BFGL-NGS-101567 | 3 | 1 | Unique |
| 234 | chr6 | 48,541,257 | 48,776,662 | 235,406 | loss | Hapmap44495-BTA-91063 | ARS-BFGL-NGS-41037 | 7 | 4 | 0.77% |
| 235 | chr6 | 49,048,876 | 49,166,985 | 118,110 | loss | BTB-01688071 | BTB-01527594 | 4 | 1 | Unique |
| 236 | chr6 | 50,046,827 | 50,255,777 | 208,951 | loss | Hapmap51152-BTA-115003 | Hapmap55410-rs29023010 | 4 | 1 | Unique |
| 237 | chr6 | 50,717,954 | 50,981,312 | 263,359 | loss | BTB-01382552 | Hapmap29605-BTA-27618 | 7 | 1 | Unique |
| 238 | chr6 | 52,521,870 | 52,725,432 | 203,563 | loss | Hapmap23184-BTC-039909 | ARS-BFGL-NGS-35824 | 8 | 3 | 0.58% |
| 239 | chr6 | 53,312,840 | 53,719,693 | 406,854 | loss | Hapmap31996-BTC-066011 | BTB-00257742 | 10 | 9 | 1.73% |
| 240 | chr6 | 56,464,060 | 56,770,486 | 306,427 | loss | BTB-00258418 | BTB-01453811 | 10 | 4 | 0.77% |
| 241 | chr6 | 57,075,347 | 57,139,728 | 64,382 | loss | Hapmap25928-BTA-18390 | BFGL-NGS-114099 | 3 | 2 | Multiple |
| 242 | chr6 | 62,788,712 | 62,933,010 | 144,299 | loss | ARS-BFGL-NGS-35835 | ARS-BFGL-NGS-109055 | 3 | 3 | 0.58% |
| 243 | chr6 | 70,376,204 | 70,546,004 | 169,801 | loss | Hapmap44512-BTA-107928 | BTA-76705-no-rs | 5 | 2 | Multiple |
| 244 | chr6 | 71,873,004 | 72,118,486 | 245,483 | gain | Hapmap31616-BTC-042811 | Hapmap29128-BTC-042274 | 7 | 1 | Unique |
| 245 | chr6 | 74,637,717 | 74,735,141 | 97,425 | loss | BTA-121759-no-rs | BTB-00264080 | 3 | 1 | Unique |
| 246 | chr6 | 77,289,713 | 77,520,815 | 231,103 | loss | BTA-28969-no-rs | BTA-114800-no-rs | 6 | 4 | 0.77% |
| 247 | chr6 | 79,901,183 | 80,291,113 | 389,931 | both | ARS-BFGL-NGS-108577 | BTB-01578666 | 10 | 13 | 2.50% |
| 248 | chr6 | 80,528,138 | 80,719,224 | 191,087 | loss | BTB-01801188 | Hapmap22939-BTA-149533 | 6 | 1 | Unique |
| 249 | chr6 | 81,019,581 | 81,057,816 | 38,236 | loss | BTB-00264815 | Hapmap51938-BTA-21491 | 3 | 1 | Unique |
| 250 | chr6 | 81,467,492 | 81,551,479 | 83,988 | loss | BTA-20903-no-rs | Hapmap27224-BTA-161106 | 3 | 2 | Multiple |
| 251 | chr6 | 83,229,855 | 83,416,784 | 186,930 | loss | BTA-76960-no-rs | Hapmap43417-BTA-96760 | 7 | 2 | Multiple |
| 252 | chr6 | 89,572,513 | 89,637,828 | 65,316 | loss | BTB-01946648 | BTA-77205-no-rs | 3 | 2 | Multiple |
| 253 | chr6 | 91,951,608 | 91,997,279 | 45,672 | gain | BTB-01496144 | BTB-01496160 | 3 | 3 | 0.58% |
| 254 | chr6 | 96,037,952 | 97,666,752 | 1,628,801 | gain | BTB-00272142 | BTB-00272812 | 33 | 1 | Unique |
| 255 | chr6 | 101,176,530 | 101,247,792 | 71,263 | gain | Hapmap39434-BTA-77640 | BTA-26167-no-rs | 3 | 1 | Unique |
| 256 | chr6 | 102,257,627 | 102,321,627 | 64,001 | loss | Hapmap26858-BTC-044514 | BFGL-NGS-117296 | 3 | 2 | Multiple |
| 257 | chr6 | 107,138,051 | 107,285,912 | 147,862 | both | ARS-BFGL-NGS-17086 | ARS-BFGL-NGS-10182 | 5 | 3 | 0.58% |
| 258 | chr6 | 108,135,281 | 108,504,034 | 368,754 | both | BTA-09585-no-rs | ARS-BFGL-NGS-56487 | 14 | 2 | Multiple |
| 259 | chr6 | 108,567,814 | 108,677,334 | 109,521 | gain | Hapmap55617-rs29025460 | ARS-BFGL-NGS-41054 | 5 | 2 | Multiple |
| 260 | chr6 | 108,971,589 | 109,868,839 | 897,251 | both | ARS-BFGL-NGS-56883 | BFGL-NGS-110156 | 27 | 6 | 1.15% |
| 261 | chr6 | 114,468,993 | 114,595,955 | 126,963 | gain | Hapmap51864-BTA-90826 | BTA-109928-no-rs | 5 | 1 | Unique |
| 262 | chr7 | 1,263,597 | 1,416,276 | 152,680 | both | ARS-BFGL-NGS-44014 | ARS-BFGL-NGS-62046 | 6 | 10 | 1.92% |
| 263 | chr7 | 9,602,196 | 12,004,746 | 2,402,551 | both | ARS-BFGL-NGS-104333 | BTB-02071318 | 28 | 108 | 20.73% |
| 264 | chr7 | 17,403,976 | 17,490,481 | 86,506 | gain | ARS-BFGL-NGS-4774 | ARS-BFGL-NGS-35463 | 3 | 1 | Unique |
| 265 | chr7 | 24,920,904 | 24,982,024 | 61,121 | loss | BTA-78589-no-rs | Hapmap51318-BTA-78584 | 3 | 4 | 0.77% |
| 266 | chr7 | 31,189,486 | 31,278,372 | 88,887 | loss | Hapmap47242-BTA-31230 | Hapmap41223-BTA-31228 | 3 | 1 | Unique |
| 267 | chr7 | 34,559,205 | 34,633,456 | 74,252 | loss | BTB-01115116 | BTB-01947935 | 4 | 1 | Unique |
| 268 | chr7 | 42,647,638 | 42,788,788 | 141,151 | both | BTB-00307598 | ARS-BFGL-NGS-23938 | 4 | 7 | 1.34% |
| 269 | chr7 | 42,811,272 | 43,311,132 | 499,861 | both | BTB-01207097 | BTB-02092796 | 16 | 35 | 6.72% |
| 270 | chr7 | 45,097,887 | 45,412,030 | 314,144 | loss | ARS-BFGL-NGS-11022 | BFGL-NGS-109750 | 8 | 1 | Unique |
| 271 | chr7 | 50,227,210 | 50,299,494 | 72,285 | gain | ARS-BFGL-NGS-57250 | BFGL-NGS-116486 | 3 | 1 | Unique |
| 272 | chr7 | 64,533,406 | 64,728,155 | 194,750 | both | DIAS-106 | Hapmap48570-BTA-104179 | 6 | 5 | 0.96% |
| 273 | chr7 | 70,118,464 | 70,276,122 | 157,659 | gain | ARS-BFGL-NGS-18754 | BTB-02018073 | 3 | 2 | Multiple |
| 274 | chr7 | 70,555,698 | 70,886,022 | 330,325 | gain | BFGL-NGS-119592 | ARS-BFGL-NGS-39099 | 8 | 4 | 0.77% |
| 275 | chr7 | 72,824,476 | 72,931,661 | 107,186 | loss | Hapmap26235-BTA-79759 | BTB-00319090 | 5 | 1 | Unique |
| 276 | chr7 | 78,081,511 | 78,348,620 | 267,110 | both | Hapmap53801-rs29012591 | Hapmap33614-BTA-79859 | 6 | 4 | 0.77% |
| 277 | chr7 | 78,401,256 | 78,599,961 | 198,706 | both | BTA-98898-no-rs | BTA-23130-no-rs | 6 | 7 | 1.34% |
| 278 | chr7 | 86,485,159 | 86,554,323 | 69,165 | loss | ARS-BFGL-NGS-92532 | ARS-BFGL-NGS-17425 | 3 | 2 | Multiple |
| 279 | chr7 | 87,087,169 | 87,241,197 | 154,029 | loss | ARS-BFGL-NGS-70648 | BTB-01388280 | 5 | 1 | Unique |
| 280 | chr7 | 87,496,026 | 88,381,298 | 885,273 | both | ARS-BFGL-NGS-3604 | BTB-00325285 | 23 | 7 | 1.34% |
| 281 | chr7 | 97,961,659 | 98,154,511 | 192,853 | loss | Hapmap36470-SCAFFOLD25306_18005 | BFGL-NGS-119112 | 5 | 2 | Multiple |
| 282 | chr7 | 103,758,284 | 103,822,789 | 64,506 | gain | BTA-80440-no-rs | ARS-BFGL-NGS-94392 | 3 | 1 | Unique |
| 283 | chr7 | 107,115,712 | 107,247,666 | 131,955 | gain | ARS-BFGL-NGS-5197 | BTB-00329283 | 5 | 1 | Unique |
| 284 | chr7 | 111,655,507 | 111,931,788 | 276,282 | loss | ARS-BFGL-NGS-84234 | Hapmap58496-rs29017214 | 11 | 1 | Unique |
| 285 | chr8 | 4,270,697 | 4,546,756 | 276,060 | loss | Hapmap54974-rs29015318 | BTA-92628-no-rs | 9 | 1 | Unique |
| 286 | chr8 | 12,900,036 | 13,022,351 | 122,316 | loss | ARS-BFGL-NGS-69683 | Hapmap42694-BTA-82927 | 4 | 2 | Multiple |
| 287 | chr8 | 13,269,109 | 13,535,724 | 266,616 | loss | Hapmap36058-SCAFFOLD313402_3070 | BTA-82931-no-rs | 5 | 1 | Unique |
| 288 | chr8 | 14,571,777 | 14,729,192 | 157,416 | loss | BTA-16324-no-rs | BTB-00336089 | 4 | 5 | 0.96% |
| 289 | chr8 | 18,987,672 | 19,047,194 | 59,523 | gain | ARS-BFGL-NGS-36682 | BTB-01099280 | 3 | 1 | Unique |
| 290 | chr8 | 22,548,179 | 22,663,016 | 114,838 | gain | Hapmap50207-BTA-108741 | BTB-01790530 | 5 | 10 | 1.92% |
| 291 | chr8 | 22,935,291 | 23,084,011 | 148,721 | gain | BTB-00338736 | ARS-BFGL-NGS-106633 | 4 | 5 | 0.96% |
| 292 | chr8 | 25,499,941 | 25,599,725 | 99,785 | loss | BFGL-NGS-110838 | Hapmap22995-BTA-120691 | 3 | 1 | Unique |
| 293 | chr8 | 25,850,783 | 26,241,724 | 390,942 | loss | Hapmap49881-BTA-120681 | ARS-BFGL-NGS-28432 | 10 | 2 | Multiple |
| 294 | chr8 | 26,971,008 | 27,198,294 | 227,287 | loss | Hapmap24639-BTA-113261 | BTA-93638-no-rs | 6 | 1 | Unique |
| 295 | chr8 | 29,977,696 | 30,129,534 | 151,839 | loss | ARS-BFGL-NGS-85886 | ARS-BFGL-NGS-16216 | 4 | 1 | Unique |
| 296 | chr8 | 31,871,995 | 31,908,986 | 36,992 | gain | BTA-123382-no-rs | Hapmap43060-BTA-81019 | 3 | 1 | Unique |
| 297 | chr8 | 32,254,996 | 32,931,536 | 676,541 | both | Hapmap55761-rs29014147 | ARS-BFGL-NGS-5314 | 12 | 3 | 0.58% |
| 298 | chr8 | 34,795,275 | 34,920,926 | 125,652 | loss | Hapmap33684-BTA-27870 | Hapmap53908-rs29021644 | 6 | 1 | Unique |
| 299 | chr8 | 37,230,536 | 37,338,560 | 108,025 | gain | BTB-00340836 | BTB-00675470 | 4 | 8 | 1.54% |
| 300 | chr8 | 43,628,838 | 43,816,192 | 187,355 | loss | ARS-BFGL-NGS-83850 | Hapmap40627-BTA-100509 | 4 | 16 | 3.07% |
| 301 | chr8 | 58,560,232 | 58,678,555 | 118,324 | loss | Hapmap27409-BTA-144593 | Hapmap46519-BTA-28770 | 5 | 1 | Unique |
| 302 | chr8 | 58,986,469 | 59,054,244 | 67,776 | loss | BTB-00350639 | Hapmap42118-BTA-16761 | 3 | 2 | Multiple |
| 303 | chr8 | 65,694,250 | 65,816,449 | 122,200 | gain | BTA-62442-no-rs | BTB-01657524 | 5 | 2 | Multiple |
| 304 | chr8 | 65,833,320 | 66,090,160 | 256,841 | gain | BFGL-NGS-110360 | BTB-01779316 | 10 | 6 | 1.15% |
| 305 | chr8 | 66,596,921 | 66,653,491 | 56,571 | gain | ARS-BFGL-NGS-78877 | BTB-01916334 | 3 | 1 | Unique |
| 306 | chr8 | 70,831,385 | 71,053,386 | 222,002 | both | BTA-95860-no-rs | BFGL-NGS-111734 | 6 | 10 | 1.92% |
| 307 | chr8 | 85,937,098 | 86,101,796 | 164,699 | both | ARS-BFGL-NGS-63666 | ARS-BFGL-NGS-104204 | 5 | 9 | 1.73% |
| 308 | chr8 | 88,464,533 | 88,717,437 | 252,905 | gain | Hapmap49329-BTA-82142 | BTB-00365092 | 5 | 1 | Unique |
| 309 | chr8 | 90,579,016 | 90,693,363 | 114,348 | gain | ARS-BFGL-NGS-102412 | Hapmap33490-BTA-146089 | 4 | 1 | Unique |
| 310 | chr8 | 91,401,417 | 91,443,463 | 42,047 | gain | ARS-BFGL-NGS-41178 | BTA-23643-no-rs | 3 | 1 | Unique |
| 311 | chr8 | 93,733,918 | 93,825,702 | 91,785 | loss | BTB-01363589 | BTB-01537249 | 3 | 1 | Unique |
| 312 | chr8 | 94,054,045 | 94,258,722 | 204,678 | loss | BTB-00367725 | BTB-00368101 | 7 | 1 | Unique |
| 313 | chr8 | 95,844,517 | 96,199,051 | 354,535 | both | BTB-01460446 | BFGL-NGS-109757 | 9 | 11 | 2.11% |
| 314 | chr8 | 96,712,245 | 96,839,903 | 127,659 | loss | Hapmap47520-BTA-117879 | Hapmap51091-BTA-92938 | 4 | 2 | Multiple |
| 315 | chr8 | 101,574,067 | 101,637,375 | 63,309 | loss | BTB-00371041 | ARS-BFGL-NGS-74165 | 3 | 3 | 0.58% |
| 316 | chr9 | 1,926,314 | 2,146,085 | 219,772 | loss | BTB-01752812 | BTB-01837292 | 7 | 1 | Unique |
| 317 | chr9 | 3,209,712 | 3,337,249 | 127,538 | loss | Hapmap36664-SCAFFOLD50340_7682 | BTB-01917058 | 4 | 1 | Unique |
| 318 | chr9 | 3,651,455 | 4,130,601 | 479,147 | loss | ARS-BFGL-NGS-26056 | BTA-83392-no-rs | 9 | 2 | Multiple |
| 319 | chr9 | 4,305,338 | 4,386,831 | 81,494 | loss | ARS-BFGL-NGS-20003 | Hapmap48549-BTA-99373 | 3 | 2 | Multiple |
| 320 | chr9 | 4,439,872 | 4,516,636 | 76,765 | loss | ARS-BFGL-NGS-42521 | Hapmap42378-BTA-97334 | 3 | 1 | Unique |
| 321 | chr9 | 5,108,189 | 5,288,398 | 180,210 | loss | Hapmap31550-BTA-83571 | BTB-01504055 | 6 | 1 | Unique |
| 322 | chr9 | 5,923,370 | 6,018,947 | 95,578 | loss | BTB-01517292 | BTB-01428058 | 4 | 2 | Multiple |
| 323 | chr9 | 12,905,791 | 13,050,004 | 144,214 | loss | ARS-BFGL-NGS-26493 | ARS-BFGL-NGS-42000 | 3 | 1 | Unique |
| 324 | chr9 | 15,312,685 | 15,640,590 | 327,906 | both | Hapmap27634-BTA-158717 | Hapmap44930-BTA-109958 | 12 | 13 | 2.50% |
| 325 | chr9 | 16,613,842 | 16,861,043 | 247,202 | gain | BTB-01632577 | BTB-01556111 | 7 | 1 | Unique |
| 326 | chr9 | 18,790,503 | 18,862,613 | 72,111 | loss | Hapmap46542-BTA-94412 | BTA-94407-no-rs | 3 | 1 | Unique |
| 327 | chr9 | 24,024,654 | 24,155,896 | 131,243 | both | BTB-01362150 | Hapmap42168-BTA-28710 | 5 | 3 | 0.58% |
| 328 | chr9 | 25,126,494 | 25,185,694 | 59,201 | loss | BTA-83093-no-rs | BTB-02020793 | 3 | 3 | 0.58% |
| 329 | chr9 | 49,521,353 | 49,597,855 | 76,503 | loss | BTA-01860-no-rs | BTB-01352876 | 3 | 2 | Multiple |
| 330 | chr9 | 54,190,285 | 54,369,375 | 179,091 | gain | Hapmap41423-BTA-109716 | Hapmap44208-BTA-119972 | 5 | 3 | 0.58% |
| 331 | chr9 | 54,715,379 | 54,778,504 | 63,126 | gain | BTA-83746-no-rs | BTB-00393918 | 3 | 1 | Unique |
| 332 | chr9 | 56,003,900 | 56,193,601 | 189,702 | loss | BTB-02010143 | BTB-01716755 | 6 | 2 | Multiple |
| 333 | chr9 | 57,891,069 | 58,075,303 | 184,235 | gain | BTA-92472-no-rs | BTB-01573139 | 7 | 1 | Unique |
| 334 | chr9 | 59,645,951 | 59,756,194 | 110,244 | loss | BTB-00395419 | Hapmap51401-BTA-117495 | 4 | 1 | Unique |
| 335 | chr9 | 71,450,748 | 71,598,281 | 147,534 | gain | ARS-BFGL-NGS-65921 | BTB-01420701 | 4 | 5 | 0.96% |
| 336 | chr9 | 79,530,093 | 79,988,795 | 458,703 | loss | BTA-113455-no-rs | Hapmap43371-BTA-84377 | 6 | 1 | Unique |
| 337 | chr9 | 85,704,836 | 85,944,461 | 239,626 | gain | BTA-84561-no-rs | ARS-BFGL-NGS-109193 | 6 | 2 | Multiple |
| 338 | chr9 | 87,926,421 | 88,043,912 | 117,492 | both | BTA-08180-no-rs | BTA-01364-no-rs | 5 | 7 | 1.34% |
| 339 | chr9 | 88,196,384 | 88,322,599 | 126,216 | gain | ARS-BFGL-NGS-16116 | BFGL-NGS-110042 | 3 | 3 | 0.58% |
| 340 | chr9 | 91,459,477 | 91,672,738 | 213,262 | loss | BTB-00403463 | Hapmap52598-rs29022272 | 9 | 1 | Unique |
| 341 | chr9 | 92,061,663 | 92,141,851 | 80,189 | loss | Hapmap59634-rs29013969 | BTB-01586455 | 4 | 3 | 0.58% |
| 342 | chr9 | 92,400,217 | 92,462,210 | 61,994 | loss | Hapmap43080-BTA-84772 | ARS-BFGL-NGS-5159 | 3 | 1 | Unique |
| 343 | chr9 | 93,472,326 | 93,552,330 | 80,005 | loss | ARS-BFGL-NGS-52931 | BFGL-NGS-112933 | 3 | 2 | Multiple |
| 344 | chr9 | 98,784,166 | 98,847,521 | 63,356 | loss | ARS-BFGL-NGS-83787 | Hapmap43375-BTA-85005 | 3 | 2 | Multiple |
| 345 | chr9 | 101,785,435 | 101,909,183 | 123,749 | gain | Hapmap48173-BTA-107833 | Hapmap38266-BTA-97562 | 4 | 1 | Unique |
| 346 | chr9 | 102,826,828 | 102,930,778 | 103,951 | loss | ARS-BFGL-NGS-26554 | BFGL-NGS-117605 | 3 | 1 | Unique |
| 347 | chr9 | 103,727,630 | 103,777,260 | 49,631 | gain | Hapmap33058-BTA-158159 | ARS-BFGL-NGS-41284 | 3 | 1 | Unique |
| 348 | chr10 | 5,392,944 | 5,437,359 | 44,416 | loss | BTA-75317-no-rs | ARS-BFGL-NGS-60051 | 3 | 1 | Unique |
| 349 | chr10 | 14,129,663 | 14,288,475 | 158,813 | loss | BTB-00410198 | Hapmap51496-BTA-79294 | 6 | 1 | Unique |
| 350 | chr10 | 14,638,720 | 14,680,950 | 42,231 | both | ARS-BFGL-NGS-14122 | ARS-BFGL-NGS-18282 | 4 | 3 | 0.58% |
| 351 | chr10 | 22,190,416 | 25,493,882 | 3,303,467 | both | ARS-BFGL-NGS-100895 | BTB-01806171 | 44 | 141 | 27.06% |
| 352 | chr10 | 27,366,173 | 27,547,387 | 181,215 | loss | ARS-BFGL-NGS-30228 | BTB-00418037 | 5 | 5 | 0.96% |
| 353 | chr10 | 32,837,737 | 32,930,297 | 92,561 | gain | Hapmap50425-BTA-63280 | Hapmap44110-BTA-63281 | 3 | 2 | Multiple |
| 354 | chr10 | 35,164,064 | 35,269,105 | 105,042 | loss | ARS-BFGL-NGS-43296 | Hapmap54460-rs29016462 | 4 | 1 | Unique |
| 355 | chr10 | 39,846,476 | 40,003,945 | 157,470 | loss | BTA-66276-no-rs | BTB-00418936 | 5 | 2 | Multiple |
| 356 | chr10 | 41,514,241 | 41,596,729 | 82,489 | both | BTB-01400850 | BTB-01482386 | 3 | 5 | 0.96% |
| 357 | chr10 | 42,189,473 | 42,238,158 | 48,686 | gain | BTA-117973-no-rs | BTA-117968-no-rs | 3 | 1 | Unique |
| 358 | chr10 | 43,194,408 | 43,326,658 | 132,251 | gain | BTA-67185-no-rs | Hapmap59191-rs29010446 | 4 | 2 | Multiple |
| 359 | chr10 | 51,416,085 | 51,502,722 | 86,638 | both | BTB-00992193 | BTB-00426535 | 4 | 3 | 0.58% |
| 360 | chr10 | 53,987,232 | 54,032,992 | 45,761 | loss | BTA-87361-no-rs | BTB-01137887 | 3 | 2 | Multiple |
| 361 | chr10 | 55,591,993 | 55,611,885 | 19,893 | gain | BTB-00428180 | Hapmap59786-rs29012019 | 3 | 1 | Unique |
| 362 | chr10 | 59,731,691 | 59,948,769 | 217,079 | loss | ARS-BFGL-NGS-87984 | BTA-66849-no-rs | 7 | 4 | 0.77% |
| 363 | chr10 | 63,947,314 | 64,059,354 | 112,041 | loss | BTA-73360-no-rs | BTA-23917-no-rs | 3 | 2 | Multiple |
| 364 | chr10 | 69,557,467 | 69,658,256 | 100,790 | loss | ARS-BFGL-NGS-105887 | BTB-00433829 | 4 | 1 | Unique |
| 365 | chr10 | 70,813,663 | 70,871,943 | 58,281 | loss | Hapmap44482-BTA-74309 | BTB-00434592 | 3 | 1 | Unique |
| 366 | chr10 | 72,023,342 | 72,119,807 | 96,466 | loss | BTB-01693217 | Hapmap27480-BTA-75244 | 3 | 3 | 0.58% |
| 367 | chr10 | 76,220,343 | 76,397,552 | 177,210 | gain | Hapmap40252-BTA-75263 | ARS-BFGL-NGS-55396 | 3 | 1 | Unique |
| 368 | chr10 | 76,931,362 | 77,032,581 | 101,220 | loss | Hapmap44484-BTA-75324 | ARS-BFGL-NGS-38839 | 3 | 1 | Unique |
| 369 | chr10 | 78,805,285 | 78,905,418 | 100,134 | gain | ARS-BFGL-NGS-55923 | BTA-76281-no-rs | 3 | 2 | Multiple |
| 370 | chr10 | 81,695,290 | 81,760,421 | 65,132 | gain | BTB-00438561 | ARS-BFGL-NGS-18421 | 3 | 1 | Unique |
| 371 | chr10 | 85,407,544 | 85,649,939 | 242,396 | loss | BTB-02091381 | ARS-BFGL-BAC-11028 | 6 | 1 | Unique |
| 372 | chr10 | 87,707,756 | 87,848,389 | 140,634 | loss | BFGL-NGS-110987 | Hapmap57672-rs29015733 | 5 | 2 | Multiple |
| 373 | chr10 | 90,060,173 | 90,119,776 | 59,604 | gain | BFGL-NGS-119578 | ARS-BFGL-NGS-14139 | 3 | 1 | Unique |
| 374 | chr10 | 94,765,926 | 94,807,697 | 41,772 | loss | ARS-BFGL-NGS-24191 | BTB-01526503 | 3 | 1 | Unique |
| 375 | chr10 | 95,172,902 | 95,232,032 | 59,131 | loss | ARS-BFGL-NGS-21459 | ARS-BFGL-NGS-5768 | 3 | 2 | Multiple |
| 376 | chr10 | 95,274,618 | 95,353,865 | 79,248 | loss | BTB-01311442 | ARS-BFGL-NGS-102179 | 3 | 2 | Multiple |
| 377 | chr11 | 394,772 | 541,577 | 146,806 | loss | Hapmap26105-BTA-126070 | ARS-BFGL-BAC-14260 | 4 | 2 | Multiple |
| 378 | chr11 | 3,971,531 | 4,097,932 | 126,402 | loss | ARS-BFGL-NGS-18450 | ARS-BFGL-NGS-105764 | 4 | 5 | 0.96% |
| 379 | chr11 | 17,359,680 | 17,484,533 | 124,854 | loss | ARS-BFGL-BAC-15477 | BTB-01766447 | 4 | 1 | Unique |
| 380 | chr11 | 19,410,756 | 19,530,404 | 119,649 | loss | ARS-BFGL-NGS-17016 | BTA-88614-no-rs | 4 | 4 | 0.77% |
| 381 | chr11 | 19,971,032 | 20,061,567 | 90,536 | loss | ARS-BFGL-NGS-102490 | BTB-00467628 | 3 | 2 | Multiple |
| 382 | chr11 | 38,375,934 | 38,474,579 | 98,646 | loss | Hapmap51861-BTA-86131 | Hapmap24189-BTA-123161 | 3 | 1 | Unique |
| 383 | chr11 | 47,116,171 | 47,183,635 | 67,465 | loss | Hapmap42396-BTA-100630 | Hapmap59563-rs29017556 | 3 | 1 | Unique |
| 384 | chr11 | 47,338,489 | 47,393,436 | 54,948 | loss | BTA-97080-no-rs | ARS-BFGL-NGS-7012 | 3 | 1 | Unique |
| 385 | chr11 | 59,421,902 | 59,724,651 | 302,750 | loss | BTB-01756713 | BTB-01475120 | 6 | 3 | 0.58% |
| 386 | chr11 | 70,016,398 | 70,438,902 | 422,505 | loss | BTA-28852-no-rs | ARS-BFGL-NGS-28978 | 12 | 1 | Unique |
| 387 | chr11 | 83,938,054 | 84,005,567 | 67,514 | gain | Hapmap52546-rs29026760 | Hapmap34777-BES11_Contig379_951 | 3 | 1 | Unique |
| 388 | chr11 | 87,793,629 | 87,935,788 | 142,160 | gain | BFGL-NGS-116951 | BFGL-NGS-114352 | 5 | 1 | Unique |
| 389 | chr11 | 91,875,823 | 92,297,411 | 421,589 | gain | BTA-109943-no-rs | Hapmap35103-BES3_Contig455_1055 | 11 | 8 | 1.54% |
| 390 | chr11 | 93,445,185 | 93,612,322 | 167,138 | gain | BTB-02006984 | ARS-BFGL-NGS-1846 | 4 | 5 | 0.96% |
| 391 | chr11 | 103,555,170 | 104,217,085 | 661,916 | both | ARS-BFGL-NGS-12348 | ARS-BFGL-NGS-25912 | 19 | 4 | 0.77% |
| 392 | chr11 | 104,633,267 | 104,706,709 | 73,443 | loss | BFGL-NGS-111682 | ARS-BFGL-NGS-14919 | 4 | 2 | Multiple |
| 393 | chr11 | 105,677,940 | 106,019,172 | 341,233 | both | ARS-BFGL-NGS-97847 | Hapmap42853-BTA-15475 | 9 | 6 | 1.15% |
| 394 | chr11 | 106,308,481 | 106,543,262 | 234,782 | both | BFGL-NGS-114359 | ARS-BFGL-NGS-25833 | 7 | 11 | 2.11% |
| 395 | chr11 | 106,974,478 | 107,096,304 | 121,827 | gain | BTA-09046-no-rs | ARS-BFGL-NGS-32260 | 4 | 1 | Unique |
| 396 | chr12 | 622,388 | 742,930 | 120,543 | both | BTB-01248923 | ARS-BFGL-NGS-64891 | 6 | 11 | 2.11% |
| 397 | chr12 | 1,546,821 | 1,725,598 | 178,778 | both | BTB-01981334 | ARS-BFGL-NGS-6218 | 6 | 2 | Multiple |
| 398 | chr12 | 3,705,325 | 3,937,685 | 232,361 | gain | Hapmap49354-BTA-87231 | Hapmap23316-BTA-147643 | 9 | 3 | 0.58% |
| 399 | chr12 | 4,459,869 | 4,516,381 | 56,513 | loss | BTB-01713684 | Hapmap43549-BTA-28582 | 3 | 3 | 0.58% |
| 400 | chr12 | 5,101,246 | 5,299,076 | 197,831 | loss | ARS-BFGL-NGS-65811 | ARS-BFGL-NGS-10688 | 4 | 6 | 1.15% |
| 401 | chr12 | 6,987,206 | 7,179,384 | 192,179 | loss | ARS-BFGL-BAC-775 | BTA-96244-no-rs | 5 | 1 | Unique |
| 402 | chr12 | 7,660,934 | 7,766,553 | 105,620 | loss | BTA-112946-no-rs | BTA-28903-no-rs | 4 | 1 | Unique |
| 403 | chr12 | 8,987,624 | 9,127,500 | 139,877 | gain | Hapmap38662-BTA-22753 | BTB-00486292 | 5 | 1 | Unique |
| 404 | chr12 | 9,515,754 | 9,574,292 | 58,539 | gain | Hapmap48117-BTA-90454 | ARS-BFGL-NGS-51579 | 3 | 1 | Unique |
| 405 | chr12 | 20,129,895 | 20,720,170 | 590,276 | loss | ARS-BFGL-NGS-21160 | ARS-BFGL-NGS-107895 | 8 | 4 | 0.77% |
| 406 | chr12 | 25,934,953 | 26,434,681 | 499,729 | loss | Hapmap26763-BTA-161285 | Hapmap24457-BTA-153392 | 9 | 2 | Multiple |
| 407 | chr12 | 31,368,562 | 31,679,957 | 311,396 | loss | ARS-BFGL-BAC-15023 | Hapmap33402-BTA-18602 | 7 | 16 | 3.07% |
| 408 | chr12 | 38,409,372 | 38,660,049 | 250,678 | loss | BTA-90709-no-rs | Hapmap49412-BTA-102739 | 6 | 1 | Unique |
| 409 | chr12 | 39,752,637 | 39,925,909 | 173,273 | loss | Hapmap43991-BTA-102559 | BTA-102528-no-rs | 4 | 2 | Multiple |
| 410 | chr12 | 45,002,070 | 45,135,480 | 133,411 | loss | BTA-21671-no-rs | Hapmap43513-BTA-21673 | 5 | 3 | 0.58% |
| 411 | chr12 | 45,358,430 | 45,409,287 | 50,858 | loss | BTB-01438340 | BTB-01438513 | 3 | 1 | Unique |
| 412 | chr12 | 45,870,499 | 45,919,459 | 48,961 | loss | BTB-00491073 | BTA-22699-no-rs | 3 | 1 | Unique |
| 413 | chr12 | 50,539,661 | 50,628,129 | 88,469 | loss | Hapmap27383-BTA-127654 | BTB-00494145 | 4 | 4 | 0.77% |
| 414 | chr12 | 52,573,538 | 52,767,067 | 193,530 | loss | BTB-00493094 | Hapmap50625-BTA-23801 | 7 | 1 | Unique |
| 415 | chr12 | 57,077,252 | 57,803,744 | 726,493 | both | BTB-02035517 | Hapmap33957-BES4_Contig323_1373 | 21 | 12 | 2.30% |
| 416 | chr12 | 58,089,757 | 58,375,654 | 285,898 | loss | BTB-01280469 | BTB-01238185 | 8 | 3 | 0.58% |
| 417 | chr12 | 59,609,816 | 59,786,584 | 176,769 | loss | BFGL-NGS-109722 | Hapmap23461-BTA-147998 | 6 | 2 | Multiple |
| 418 | chr12 | 62,069,128 | 62,194,350 | 125,223 | loss | BTB-01989473 | BTB-01922844 | 4 | 1 | Unique |
| 419 | chr12 | 62,648,430 | 62,813,820 | 165,391 | loss | Hapmap55458-rs29025293 | BTB-01507160 | 5 | 1 | Unique |
| 420 | chr12 | 63,969,950 | 64,461,309 | 491,360 | both | Hapmap33377-BTA-152903 | BTB-01499710 | 11 | 13 | 2.50% |
| 421 | chr12 | 64,866,107 | 65,481,742 | 615,636 | loss | Hapmap51577-BTA-27003 | BTB-01733812 | 9 | 3 | 0.58% |
| 422 | chr12 | 68,453,584 | 68,506,471 | 52,888 | gain | Hapmap48110-BTA-88422 | BTB-00501374 | 3 | 1 | Unique |
| 423 | chr12 | 70,331,880 | 72,227,978 | 1,896,099 | both | BTB-01971757 | BFGL-NGS-117613 | 39 | 391 | 75.05% |
| 424 | chr12 | 72,341,102 | 76,739,759 | 4,398,658 | both | BTA-95991-no-rs | ARS-BFGL-NGS-77009 | 71 | 420 | 80.61% |
| 425 | chr12 | 77,315,938 | 77,388,557 | 72,620 | loss | ARS-BFGL-NGS-57541 | Hapmap49113-BTA-30008 | 4 | 1 | Unique |
| 426 | chr12 | 78,014,490 | 78,146,737 | 132,248 | loss | ARS-BFGL-NGS-14529 | ARS-BFGL-NGS-53179 | 3 | 2 | Multiple |
| 427 | chr12 | 82,159,124 | 82,260,121 | 100,998 | loss | BTA-100491-no-rs | Hapmap43198-BTA-19292 | 3 | 1 | Unique |
| 428 | chr12 | 90,015,695 | 90,101,327 | 85,633 | loss | ARS-BFGL-NGS-106663 | ARS-BFGL-NGS-3885 | 4 | 4 | 0.77% |
| 429 | chr12 | 90,179,467 | 90,412,384 | 232,918 | both | ARS-BFGL-NGS-89118 | ARS-BFGL-NGS-23827 | 7 | 8 | 1.54% |
| 430 | chr12 | 91,057,879 | 91,116,965 | 59,087 | gain | Hapmap46770-BTA-120932 | BTA-92634-no-rs | 3 | 1 | Unique |
| 431 | chr13 | 104,140 | 999,229 | 895,090 | both | ARS-BFGL-NGS-22160 | Hapmap48963-BTA-96734 | 16 | 7 | 1.34% |
| 432 | chr13 | 1,477,972 | 1,531,838 | 53,867 | loss | Hapmap45253-BTA-15908 | ARS-BFGL-NGS-59455 | 3 | 2 | Multiple |
| 433 | chr13 | 3,527,716 | 3,609,795 | 82,080 | both | BTB-01935769 | BTA-117397-no-rs | 3 | 4 | 0.77% |
| 434 | chr13 | 7,970,420 | 8,006,379 | 35,960 | loss | Hapmap38890-BTA-32883 | Hapmap24075-BTA-128035 | 3 | 1 | Unique |
| 435 | chr13 | 10,508,131 | 10,806,321 | 298,191 | loss | ARS-BFGL-NGS-32505 | ARS-BFGL-NGS-85588 | 9 | 1 | Unique |
| 436 | chr13 | 11,158,082 | 11,504,730 | 346,649 | gain | BTA-123464-no-rs | BTA-61986-no-rs | 9 | 17 | 3.26% |
| 437 | chr13 | 12,009,567 | 12,152,700 | 143,134 | gain | ARS-BFGL-NGS-27780 | ARS-BFGL-NGS-6320 | 5 | 2 | Multiple |
| 438 | chr13 | 12,587,622 | 12,808,180 | 220,559 | gain | ARS-BFGL-NGS-2317 | ARS-BFGL-BAC-785 | 6 | 3 | 0.58% |
| 439 | chr13 | 14,356,317 | 14,443,021 | 86,705 | loss | BTA-08210-rs29021773 | Hapmap43530-BTA-25745 | 3 | 2 | Multiple |
| 440 | chr13 | 16,901,756 | 16,988,665 | 86,910 | loss | UA-IFASA-8290 | Hapmap41504-BTA-31952 | 3 | 8 | 1.54% |
| 441 | chr13 | 17,696,147 | 17,941,765 | 245,619 | gain | ARS-BFGL-BAC-7407 | Hapmap39401-BTA-34229 | 8 | 5 | 0.96% |
| 442 | chr13 | 36,672,545 | 36,782,813 | 110,269 | loss | BTA-17784-no-rs | Hapmap25918-BTA-163236 | 3 | 2 | Multiple |
| 443 | chr13 | 43,152,577 | 43,191,991 | 39,415 | loss | ARS-BFGL-NGS-100409 | ARS-BFGL-NGS-53405 | 3 | 1 | Unique |
| 444 | chr13 | 44,876,436 | 44,947,976 | 71,541 | loss | ARS-BFGL-NGS-101595 | ARS-BFGL-NGS-41527 | 3 | 2 | Multiple |
| 445 | chr13 | 53,864,519 | 53,993,014 | 128,496 | gain | ARS-BFGL-NGS-59418 | Hapmap39639-BTA-25037 | 5 | 2 | Multiple |
| 446 | chr13 | 55,365,134 | 55,409,520 | 44,387 | gain | ARS-BFGL-NGS-68008 | Hapmap46742-BTA-01253 | 3 | 2 | Multiple |
| 447 | chr13 | 70,392,504 | 70,496,054 | 103,551 | gain | ARS-BFGL-NGS-63200 | ARS-BFGL-NGS-78378 | 3 | 4 | 0.77% |
| 448 | chr13 | 71,860,088 | 71,919,584 | 59,497 | loss | Hapmap53091-rs29015689 | ARS-BFGL-NGS-3159 | 3 | 1 | Unique |
| 449 | chr13 | 83,242,122 | 83,371,147 | 129,026 | gain | ARS-BFGL-NGS-14974 | BTB-01317883 | 4 | 1 | Unique |
| 450 | chr14 | 1,164,764 | 1,393,146 | 228,383 | gain | BTB-02078886 | BTB-02008469 | 5 | 1 | Unique |
| 451 | chr14 | 1,616,618 | 1,841,484 | 224,867 | loss | ARS-BFGL-NGS-20321 | ARS-BFGL-BAC-26048 | 9 | 1 | Unique |
| 452 | chr14 | 3,994,681 | 4,438,267 | 443,587 | gain | Hapmap33328-BTC-064942 | ARS-BFGL-NGS-42263 | 17 | 4 | 0.77% |
| 453 | chr14 | 9,215,622 | 9,345,140 | 129,519 | loss | Hapmap36763-SCAFFOLD240007_5847 | BFGL-NGS-112271 | 4 | 2 | Multiple |
| 454 | chr14 | 10,446,281 | 10,491,491 | 45,211 | loss | Hapmap31972-BTC-057258 | Hapmap26890-BTC-057492 | 3 | 2 | Multiple |
| 455 | chr14 | 14,474,033 | 15,460,695 | 986,663 | both | ARS-BFGL-NGS-104239 | ARS-BFGL-NGS-54437 | 14 | 111 | 21.31% |
| 456 | chr14 | 17,378,950 | 17,457,836 | 78,887 | loss | BFGL-NGS-114730 | UA-IFASA-9282 | 3 | 4 | 0.77% |
| 457 | chr14 | 20,047,788 | 20,196,617 | 148,830 | gain | BTB-01720493 | BTB-01283269 | 5 | 1 | Unique |
| 458 | chr14 | 27,380,992 | 27,465,907 | 84,916 | loss | Hapmap26621-BTC-072953 | ARS-BFGL-BAC-1290 | 4 | 2 | Multiple |
| 459 | chr14 | 30,702,073 | 30,818,549 | 116,477 | loss | BTB-01936090 | BTB-00563836 | 4 | 1 | Unique |
| 460 | chr14 | 35,518,739 | 35,590,232 | 71,494 | loss | Hapmap61004-rs29017348 | BFGL-NGS-109998 | 3 | 1 | Unique |
| 461 | chr14 | 49,883,707 | 49,955,911 | 72,205 | loss | ARS-BFGL-NGS-33585 | Hapmap51215-BTA-34925 | 3 | 2 | Multiple |
| 462 | chr14 | 51,540,635 | 51,890,266 | 349,632 | both | Hapmap58389-rs29019587 | UA-IFASA-8223 | 8 | 3 | 0.58% |
| 463 | chr14 | 52,049,002 | 52,244,037 | 195,036 | both | BTA-111311-no-rs | BTB-01212678 | 6 | 3 | 0.58% |
| 464 | chr14 | 53,023,931 | 53,324,858 | 300,928 | loss | BTA-34930-no-rs | ARS-BFGL-BAC-1625 | 9 | 1 | Unique |
| 465 | chr14 | 53,979,439 | 54,501,131 | 521,693 | both | Hapmap46234-BTA-60176 | BTA-90428-no-rs | 12 | 6 | 1.15% |
| 466 | chr14 | 55,141,942 | 55,219,280 | 77,339 | loss | BTB-00333025 | BTB-00572165 | 3 | 1 | Unique |
| 467 | chr14 | 55,592,245 | 55,638,745 | 46,501 | loss | ARS-BFGL-BAC-23616 | ARS-BFGL-BAC-23606 | 3 | 3 | 0.58% |
| 468 | chr14 | 55,765,964 | 55,910,671 | 144,708 | loss | Hapmap60925-rs29018458 | Hapmap31256-BTC-012280 | 7 | 1 | Unique |
| 469 | chr14 | 56,031,029 | 56,156,340 | 125,312 | loss | Hapmap33279-BTC-012214 | Hapmap23234-BTC-001732 | 5 | 1 | Unique |
| 470 | chr14 | 62,794,502 | 63,004,255 | 209,754 | loss | BTB-00575283 | BTB-01048106 | 5 | 1 | Unique |
| 471 | chr14 | 72,827,899 | 72,937,271 | 109,373 | gain | Hapmap50328-BTA-35439 | BFGL-NGS-110770 | 4 | 5 | 0.96% |
| 472 | chr14 | 76,348,124 | 76,394,153 | 46,030 | gain | ARS-BFGL-NGS-13904 | ARS-BFGL-NGS-843 | 3 | 1 | Unique |
| 473 | chr14 | 79,178,022 | 79,322,701 | 144,680 | loss | ARS-BFGL-NGS-61550 | ARS-BFGL-NGS-63487 | 3 | 3 | 0.58% |
| 474 | chr14 | 79,486,476 | 79,523,486 | 37,011 | gain | BTB-01388619 | UA-IFASA-9226 | 3 | 2 | Multiple |
| 475 | chr14 | 83,582,981 | 83,749,297 | 166,317 | loss | Hapmap26378-BTA-129537 | Hapmap34730-BES8_Contig464_1373 | 3 | 1 | Unique |
| 476 | chr15 | 3,084,118 | 3,416,019 | 331,902 | loss | BTA-26081-no-rs | BTB-02021101 | 7 | 5 | 0.96% |
| 477 | chr15 | 4,944,846 | 5,001,246 | 56,401 | loss | BTA-102514-no-rs | BTB-01545112 | 3 | 1 | Unique |
| 478 | chr15 | 6,548,250 | 6,808,535 | 260,286 | loss | BTB-00580383 | Hapmap23284-BTA-162040 | 4 | 1 | Unique |
| 479 | chr15 | 9,315,623 | 9,474,687 | 159,065 | gain | Hapmap49562-BTA-29896 | BTB-01417458 | 3 | 1 | Unique |
| 480 | chr15 | 10,320,166 | 10,544,820 | 224,655 | both | ARS-BFGL-NGS-26612 | BTB-00580638 | 5 | 9 | 1.73% |
| 481 | chr15 | 10,792,635 | 10,906,509 | 113,875 | gain | ARS-BFGL-NGS-3060 | BTB-01538812 | 3 | 2 | Multiple |
| 482 | chr15 | 11,001,598 | 11,804,058 | 802,461 | loss | BTB-01538650 | ARS-BFGL-NGS-14904 | 16 | 4 | 0.77% |
| 483 | chr15 | 12,329,817 | 12,412,932 | 83,116 | loss | BTB-02020473 | Hapmap44444-BTA-115434 | 3 | 2 | Multiple |
| 484 | chr15 | 15,305,071 | 15,480,683 | 175,613 | loss | ARS-BFGL-BAC-31586 | Hapmap26711-BTA-149759 | 4 | 1 | Unique |
| 485 | chr15 | 16,655,251 | 16,752,425 | 97,175 | gain | BTA-121003-no-rs | Hapmap44541-BTA-37632 | 3 | 2 | Multiple |
| 486 | chr15 | 21,353,698 | 21,401,836 | 48,139 | gain | Hapmap38222-BTA-38012 | BTB-00582817 | 3 | 1 | Unique |
| 487 | chr15 | 24,588,338 | 24,673,090 | 84,753 | loss | ARS-BFGL-NGS-19320 | ARS-BFGL-BAC-20623 | 4 | 2 | Multiple |
| 488 | chr15 | 46,495,060 | 47,085,423 | 590,364 | gain | BTA-109760-no-rs | ARS-BFGL-NGS-108569 | 10 | 60 | 11.52% |
| 489 | chr15 | 47,819,771 | 48,011,891 | 192,121 | gain | ARS-BFGL-NGS-32444 | BTB-01907848 | 5 | 12 | 2.30% |
| 490 | chr15 | 49,344,597 | 49,538,735 | 194,139 | gain | ARS-BFGL-BAC-19376 | BTB-01917542 | 4 | 14 | 2.69% |
| 491 | chr15 | 49,987,359 | 50,061,056 | 73,698 | gain | BTB-01465034 | BTB-01797941 | 4 | 3 | 0.58% |
| 492 | chr15 | 50,176,540 | 50,270,235 | 93,696 | loss | ARS-BFGL-NGS-102432 | BTB-01928934 | 3 | 2 | Multiple |
| 493 | chr15 | 50,506,552 | 50,629,207 | 122,656 | gain | BTB-01980325 | BTB-01371877 | 3 | 8 | 1.54% |
| 494 | chr15 | 52,205,391 | 52,281,124 | 75,734 | both | UA-IFASA-8473 | BTB-02036460 | 4 | 6 | 1.15% |
| 495 | chr15 | 57,096,106 | 57,179,894 | 83,789 | loss | BTB-00519537 | BTB-00606070 | 3 | 1 | Unique |
| 496 | chr15 | 57,537,540 | 57,715,200 | 177,661 | loss | ARS-BFGL-BAC-19996 | ARS-BFGL-BAC-19991 | 7 | 6 | 1.15% |
| 497 | chr15 | 69,418,001 | 69,552,205 | 134,205 | gain | BTB-00612677 | ARS-BFGL-NGS-74913 | 5 | 3 | 0.58% |
| 498 | chr15 | 70,686,321 | 70,921,852 | 235,532 | loss | BTA-89503-no-rs | BTB-00614284 | 8 | 2 | Multiple |
| 499 | chr15 | 79,720,479 | 79,881,718 | 161,240 | gain | ARS-BFGL-NGS-94527 | BTB-01626557 | 3 | 2 | Multiple |
| 500 | chr15 | 80,003,887 | 80,097,824 | 93,938 | gain | BTB-02096198 | BTB-02012820 | 4 | 2 | Multiple |
| 501 | chr15 | 80,216,620 | 80,956,869 | 740,250 | both | ARS-BFGL-NGS-85785 | BFGL-NGS-113528 | 12 | 157 | 30.13% |
| 502 | chr15 | 83,410,335 | 83,970,308 | 559,974 | gain | BTB-00622199 | BTB-00622270 | 12 | 67 | 12.86% |
| 503 | chr15 | 84,614,250 | 84,730,976 | 116,727 | loss | Hapmap24864-BTA-155172 | ARS-BFGL-NGS-42520 | 5 | 4 | 0.77% |
| 504 | chr15 | 84,865,591 | 85,203,839 | 338,249 | loss | UA-IFASA-5953 | ARS-BFGL-NGS-77257 | 9 | 1 | Unique |
| 505 | chr16 | 259,426 | 518,808 | 259,383 | gain | ARS-BFGL-NGS-79783 | BTB-00623459 | 7 | 12 | 2.30% |
| 506 | chr16 | 5,447,029 | 6,121,332 | 674,304 | both | BTB-01927506 | BTB-01554200 | 14 | 71 | 13.63% |
| 507 | chr16 | 6,523,545 | 6,619,400 | 95,856 | both | BTB-02010595 | Hapmap33722-BTA-155362 | 3 | 4 | 0.77% |
| 508 | chr16 | 6,827,969 | 7,623,582 | 795,614 | both | ARS-BFGL-BAC-20641 | BTB-02077556 | 5 | 14 | 2.69% |
| 509 | chr16 | 8,868,238 | 9,016,133 | 147,896 | loss | BTB-01541076 | BTA-107144-no-rs | 5 | 2 | Multiple |
| 510 | chr16 | 9,148,784 | 9,640,741 | 491,958 | loss | Hapmap43197-BTA-18754 | BTB-01221760 | 17 | 2 | Multiple |
| 511 | chr16 | 9,981,001 | 10,227,386 | 246,386 | loss | ARS-BFGL-BAC-6371 | ARS-BFGL-NGS-101869 | 7 | 11 | 2.11% |
| 512 | chr16 | 10,254,748 | 10,474,014 | 219,267 | loss | BTA-39609-no-rs | Hapmap43811-BTA-22208 | 9 | 5 | 0.96% |
| 513 | chr16 | 10,805,391 | 10,989,690 | 184,300 | loss | Hapmap26204-BTA-160802 | BTB-01691083 | 5 | 1 | Unique |
| 514 | chr16 | 11,096,101 | 11,166,330 | 70,230 | loss | Hapmap33576-BTA-130425 | BTB-01533537 | 3 | 1 | Unique |
| 515 | chr16 | 16,095,676 | 16,244,657 | 148,982 | loss | BTA-91807-no-rs | BTA-91792-no-rs | 5 | 3 | 0.58% |
| 516 | chr16 | 16,340,214 | 16,453,639 | 113,426 | gain | BTA-27064-no-rs | BTA-122806-no-rs | 3 | 1 | Unique |
| 517 | chr16 | 16,711,209 | 17,036,829 | 325,621 | loss | BTA-06888-no-rs | Hapmap23394-BTA-130434 | 3 | 1 | Unique |
| 518 | chr16 | 23,305,801 | 23,471,049 | 165,249 | loss | ARS-BFGL-NGS-76705 | Hapmap42053-BTA-107968 | 3 | 1 | Unique |
| 519 | chr16 | 29,380,948 | 29,515,575 | 134,628 | gain | ARS-BFGL-NGS-61461 | ARS-BFGL-NGS-75970 | 3 | 1 | Unique |
| 520 | chr16 | 44,561,165 | 44,902,559 | 341,395 | gain | ARS-BFGL-NGS-16286 | ARS-BFGL-NGS-12988 | 4 | 4 | 0.77% |
| 521 | chr16 | 49,355,913 | 49,455,109 | 99,197 | gain | BFGL-NGS-118111 | ARS-BFGL-NGS-40119 | 3 | 2 | Multiple |
| 522 | chr16 | 50,256,549 | 50,326,081 | 69,533 | gain | BTB-01842164 | ARS-BFGL-NGS-26113 | 4 | 1 | Unique |
| 523 | chr16 | 50,737,342 | 50,928,943 | 191,602 | loss | BFGL-NGS-116784 | ARS-BFGL-NGS-44177 | 7 | 1 | Unique |
| 524 | chr16 | 52,402,205 | 52,452,122 | 49,918 | loss | ARS-BFGL-NGS-98071 | ARS-BFGL-NGS-32799 | 3 | 1 | Unique |
| 525 | chr16 | 58,650,800 | 58,855,917 | 205,118 | gain | ARS-BFGL-BAC-34694 | BFGL-NGS-116297 | 5 | 4 | 0.77% |
| 526 | chr16 | 61,609,265 | 61,716,582 | 107,318 | loss | BTB-00650039 | BTB-00650136 | 4 | 1 | Unique |
| 527 | chr16 | 61,960,715 | 62,179,726 | 219,012 | gain | BTB-01732320 | BTB-00653808 | 6 | 2 | Multiple |
| 528 | chr16 | 69,542,463 | 69,847,424 | 304,962 | loss | BTA-90680-no-rs | BFGL-NGS-113069 | 9 | 1 | Unique |
| 529 | chr16 | 70,906,202 | 71,125,864 | 219,663 | both | ARS-BFGL-NGS-29266 | ARS-BFGL-NGS-69228 | 5 | 5 | 0.96% |
| 530 | chr16 | 73,573,383 | 73,639,121 | 65,739 | gain | BFGL-NGS-119389 | ARS-BFGL-NGS-11990 | 3 | 1 | Unique |
| 531 | chr16 | 74,481,728 | 74,532,751 | 51,024 | gain | ARS-BFGL-NGS-31253 | ARS-BFGL-BAC-27042 | 3 | 2 | Multiple |
| 532 | chr17 | 1,514,342 | 1,970,122 | 455,781 | loss | Hapmap58958-rs29014979 | Hapmap60791-rs29018318 | 10 | 1 | Unique |
| 533 | chr17 | 6,436,523 | 6,556,513 | 119,991 | loss | Hapmap32996-BTA-131276 | Hapmap36186-SCAFFOLD206949_1268 | 4 | 1 | Unique |
| 534 | chr17 | 8,776,227 | 9,027,765 | 251,539 | both | Hapmap47003-BTA-42050 | Hapmap41863-BTA-42046 | 9 | 90 | 17.27% |
| 535 | chr17 | 11,518,115 | 11,638,348 | 120,234 | loss | ARS-BFGL-NGS-15402 | BTA-21866-no-rs | 3 | 5 | 0.96% |
| 536 | chr17 | 14,141,384 | 14,558,961 | 417,578 | gain | Hapmap33086-BTA-19277 | ARS-BFGL-NGS-82854 | 8 | 5 | 0.96% |
| 537 | chr17 | 14,997,063 | 15,052,590 | 55,528 | loss | Hapmap60968-rs29016935 | BTB-00673952 | 3 | 1 | Unique |
| 538 | chr17 | 18,804,260 | 18,881,179 | 76,920 | loss | Hapmap51224-BTA-40595 | Hapmap40057-BTA-25640 | 3 | 1 | Unique |
| 539 | chr17 | 22,421,753 | 27,855,634 | 5,433,882 | both | Hapmap55890-rs29027310 | ARS-BFGL-NGS-101527 | 83 | 11 | 2.11% |
| 540 | chr17 | 31,516,028 | 31,670,335 | 154,308 | loss | Hapmap28180-BTA-152938 | Hapmap51554-BTA-114371 | 5 | 4 | 0.77% |
| 541 | chr17 | 37,386,292 | 37,542,710 | 156,419 | gain | Hapmap43386-BTA-87090 | UA-IFASA-6417 | 3 | 1 | Unique |
| 542 | chr17 | 43,597,714 | 44,463,115 | 865,402 | gain | BTB-01220886 | BTA-28157-no-rs | 23 | 1 | Unique |
| 543 | chr17 | 50,784,064 | 51,097,333 | 313,270 | loss | ARS-BFGL-NGS-67903 | ARS-BFGL-NGS-75066 | 4 | 1 | Unique |
| 544 | chr17 | 61,171,044 | 61,248,541 | 77,498 | gain | BTA-115762-no-rs | ARS-BFGL-NGS-54448 | 3 | 2 | Multiple |
| 545 | chr17 | 65,257,603 | 65,321,730 | 64,128 | gain | ARS-BFGL-NGS-39345 | Hapmap50355-BTA-41590 | 3 | 1 | Unique |
| 546 | chr17 | 69,030,893 | 69,085,018 | 54,126 | loss | ARS-BFGL-NGS-11355 | BTB-01121732 | 3 | 1 | Unique |
| 547 | chr17 | 70,161,402 | 70,290,307 | 128,906 | gain | Hapmap27966-BTA-149885 | ARS-BFGL-NGS-103734 | 3 | 1 | Unique |
| 548 | chr17 | 71,544,728 | 71,788,904 | 244,177 | gain | ARS-BFGL-NGS-98109 | Hapmap47287-BTA-41904 | 9 | 3 | 0.58% |
| 549 | chr17 | 72,928,125 | 73,118,011 | 189,887 | both | BTB-01972007 | ARS-BFGL-NGS-41599 | 8 | 8 | 1.54% |
| 550 | chr17 | 73,392,682 | 75,144,374 | 1,751,693 | both | ARS-BFGL-NGS-38439 | BTA-25471-no-rs | 52 | 20 | 3.84% |
| 551 | chr18 | 1,486,363 | 1,550,114 | 63,752 | loss | ARS-BFGL-NGS-88592 | BFGL-NGS-114633 | 3 | 1 | Unique |
| 552 | chr18 | 2,793,266 | 2,864,232 | 70,967 | loss | ARS-BFGL-NGS-16731 | Hapmap49988-BTA-42565 | 3 | 2 | Multiple |
| 553 | chr18 | 12,399,824 | 12,491,673 | 91,850 | gain | ARS-BFGL-BAC-33119 | BFGL-NGS-116678 | 3 | 7 | 1.34% |
| 554 | chr18 | 27,863,340 | 28,324,801 | 461,462 | both | ARS-BFGL-NGS-39041 | ARS-BFGL-NGS-108426 | 7 | 5 | 0.96% |
| 555 | chr18 | 30,812,669 | 30,929,829 | 117,161 | gain | ARS-BFGL-NGS-37442 | Hapmap49989-BTA-42921 | 3 | 1 | Unique |
| 556 | chr18 | 35,971,459 | 36,040,190 | 68,732 | loss | BFGL-NGS-113568 | ARS-BFGL-NGS-52078 | 3 | 2 | Multiple |
| 557 | chr18 | 38,394,166 | 38,471,116 | 76,951 | gain | Hapmap48151-BTA-101548 | ARS-BFGL-NGS-12047 | 3 | 1 | Unique |
| 558 | chr18 | 44,675,555 | 44,719,288 | 43,734 | gain | BTA-88168-no-rs | Hapmap40732-BTA-43433 | 3 | 2 | Multiple |
| 559 | chr18 | 50,945,477 | 51,105,877 | 160,401 | gain | BFGL-NGS-118266 | BTA-07285-no-rs | 3 | 20 | 3.84% |
| 560 | chr18 | 53,393,358 | 53,452,847 | 59,490 | gain | ARS-BFGL-BAC-34398 | ARS-BFGL-NGS-71842 | 3 | 4 | 0.77% |
| 561 | chr18 | 55,028,139 | 55,224,848 | 196,710 | gain | ARS-BFGL-NGS-10623 | ARS-BFGL-NGS-70161 | 5 | 11 | 2.11% |
| 562 | chr18 | 57,565,406 | 57,659,303 | 93,898 | gain | BTA-97501-no-rs | UA-IFASA-8862 | 4 | 13 | 2.50% |
| 563 | chr18 | 58,832,747 | 59,178,442 | 345,696 | gain | ARS-BFGL-NGS-15484 | BFGL-NGS-112714 | 5 | 1 | Unique |
| 564 | chr18 | 59,486,268 | 59,586,254 | 99,987 | gain | ARS-BFGL-NGS-102538 | ARS-BFGL-NGS-82090 | 3 | 1 | Unique |
| 565 | chr18 | 60,658,732 | 60,774,670 | 115,939 | gain | BTB-02030509 | BTB-01457720 | 3 | 1 | Unique |
| 566 | chr18 | 60,927,319 | 61,095,214 | 167,896 | loss | ARS-BFGL-NGS-99840 | ARS-BFGL-NGS-11218 | 4 | 1 | Unique |
| 567 | chr18 | 61,267,887 | 62,017,556 | 749,670 | both | ARS-BFGL-NGS-49873 | ARS-BFGL-NGS-21069 | 16 | 61 | 11.71% |
| 568 | chr19 | 2,599,454 | 2,722,336 | 122,883 | gain | BTA-25159-no-rs | ARS-BFGL-NGS-27194 | 3 | 2 | Multiple |
| 569 | chr19 | 5,478,122 | 5,533,206 | 55,085 | gain | Hapmap41273-BTA-46442 | ARS-BFGL-NGS-34992 | 3 | 1 | Unique |
| 570 | chr19 | 5,763,142 | 5,934,293 | 171,152 | loss | ARS-BFGL-NGS-102180 | BFGL-NGS-114778 | 4 | 2 | Multiple |
| 571 | chr19 | 6,337,869 | 6,421,447 | 83,579 | gain | ARS-BFGL-NGS-82387 | ARS-BFGL-NGS-107736 | 3 | 1 | Unique |
| 572 | chr19 | 9,254,757 | 9,354,310 | 99,554 | both | Hapmap51888-BTA-16242 | Hapmap25248-BTA-132715 | 3 | 6 | 1.15% |
| 573 | chr19 | 11,137,122 | 11,238,727 | 101,606 | gain | ARS-BFGL-NGS-37614 | Hapmap30007-BTA-147050 | 3 | 1 | Unique |
| 574 | chr19 | 11,863,651 | 11,998,872 | 135,222 | loss | Hapmap52907-rs29010949 | ARS-BFGL-NGS-40746 | 5 | 2 | Multiple |
| 575 | chr19 | 12,816,221 | 12,892,995 | 76,775 | gain | UA-IFASA-6460 | ARS-BFGL-BAC-36180 | 4 | 8 | 1.54% |
| 576 | chr19 | 16,458,206 | 16,539,982 | 81,777 | gain | Hapmap53903-rs29027471 | ARS-BFGL-NGS-26149 | 4 | 2 | Multiple |
| 577 | chr19 | 19,809,038 | 20,000,986 | 191,949 | both | BFGL-NGS-113381 | UA-IFASA-6968 | 7 | 18 | 3.45% |
| 578 | chr19 | 22,492,349 | 22,574,875 | 82,527 | gain | ARS-BFGL-NGS-100026 | BFGL-NGS-112338 | 3 | 2 | Multiple |
| 579 | chr19 | 32,148,966 | 32,214,521 | 65,556 | loss | Hapmap59534-rs29022537 | BTA-05960-no-rs | 3 | 2 | Multiple |
| 580 | chr19 | 35,191,657 | 35,253,851 | 62,195 | loss | ARS-BFGL-NGS-101953 | ARS-BFGL-NGS-4759 | 3 | 1 | Unique |
| 581 | chr19 | 36,312,509 | 36,395,869 | 83,361 | loss | BTA-45152-no-rs | Hapmap44379-BTA-45149 | 3 | 1 | Unique |
| 582 | chr19 | 42,509,010 | 42,580,813 | 71,804 | loss | BFGL-NGS-119419 | ARS-BFGL-NGS-19337 | 3 | 1 | Unique |
| 583 | chr19 | 50,336,021 | 50,395,622 | 59,602 | both | ARS-BFGL-NGS-104238 | ARS-BFGL-NGS-22742 | 3 | 2 | Multiple |
| 584 | chr19 | 52,799,844 | 52,878,628 | 78,785 | both | Hapmap45689-BTA-91003 | BTB-01416331 | 3 | 2 | Multiple |
| 585 | chr19 | 54,920,324 | 55,024,753 | 104,430 | gain | ARS-BFGL-NGS-25156 | BFGL-NGS-116713 | 4 | 1 | Unique |
| 586 | chr19 | 57,454,104 | 57,558,801 | 104,698 | gain | ARS-BFGL-NGS-28678 | ARS-BFGL-NGS-34845 | 3 | 1 | Unique |
| 587 | chr19 | 62,314,941 | 62,533,475 | 218,535 | loss | UA-IFASA-9580 | ARS-BFGL-NGS-39527 | 4 | 2 | Multiple |
| 588 | chr20 | 18,913,278 | 18,974,803 | 61,526 | loss | BTA-110416-no-rs | BTA-110421-no-rs | 3 | 1 | Unique |
| 589 | chr20 | 28,442,075 | 28,506,228 | 64,154 | loss | BTA-50178-no-rs | ARS-BFGL-BAC-27918 | 3 | 2 | Multiple |
| 590 | chr20 | 29,758,994 | 29,944,792 | 185,799 | loss | Hapmap32390-BTA-162568 | ARS-BFGL-BAC-27933 | 6 | 1 | Unique |
| 591 | chr20 | 34,902,057 | 34,981,347 | 79,291 | loss | BTB-00778405 | ARS-BFGL-BAC-31754 | 4 | 1 | Unique |
| 592 | chr20 | 36,033,202 | 36,097,136 | 63,935 | gain | Hapmap53443-rs29021255 | Hapmap40359-BTA-50418 | 3 | 2 | Multiple |
| 593 | chr20 | 39,404,717 | 39,691,234 | 286,518 | loss | Hapmap31493-BTA-155702 | ARS-BFGL-NGS-14869 | 8 | 1 | Unique |
| 594 | chr20 | 43,004,103 | 43,511,650 | 507,548 | both | BTB-01263230 | ARS-BFGL-BAC-34877 | 15 | 5 | 0.96% |
| 595 | chr20 | 43,878,780 | 44,077,306 | 198,527 | both | BTB-01908187 | BTB-01441573 | 5 | 18 | 3.45% |
| 596 | chr20 | 45,083,603 | 45,144,743 | 61,141 | loss | ARS-BFGL-BAC-27247 | Hapmap41127-BTA-101667 | 3 | 1 | Unique |
| 597 | chr20 | 45,734,507 | 46,272,249 | 537,743 | loss | Hapmap32803-BTA-135212 | ARS-BFGL-BAC-32350 | 16 | 6 | 1.15% |
| 598 | chr20 | 46,787,179 | 47,226,500 | 439,322 | loss | Hapmap39563-BTA-41519 | BTB-01905047 | 12 | 5 | 0.96% |
| 599 | chr20 | 47,750,656 | 47,839,336 | 88,681 | loss | BTA-27302-no-rs | Hapmap54884-rs29017180 | 4 | 2 | Multiple |
| 600 | chr20 | 50,128,719 | 50,443,672 | 314,954 | loss | BTB-01529366 | Hapmap54729-rs29023630 | 7 | 10 | 1.92% |
| 601 | chr20 | 51,422,910 | 52,011,466 | 588,557 | loss | BTB-00787134 | BTB-01748945 | 15 | 3 | 0.58% |
| 602 | chr20 | 53,798,275 | 53,991,925 | 193,651 | loss | BTB-00788635 | BTB-01435010 | 4 | 1 | Unique |
| 603 | chr20 | 54,470,397 | 54,630,287 | 159,891 | both | BTB-01127950 | BTB-01128234 | 5 | 5 | 0.96% |
| 604 | chr20 | 55,554,731 | 55,693,187 | 138,457 | loss | BTB-01705150 | BTB-01304871 | 4 | 5 | 0.96% |
| 605 | chr20 | 64,311,152 | 64,376,028 | 64,877 | gain | BTA-121292-no-rs | ARS-BFGL-NGS-21633 | 3 | 2 | Multiple |
| 606 | chr20 | 67,235,535 | 67,289,400 | 53,866 | gain | BFGL-NGS-110034 | ARS-BFGL-NGS-39177 | 3 | 1 | Unique |
| 607 | chr20 | 70,301,283 | 70,386,902 | 85,620 | gain | ARS-BFGL-NGS-31306 | BTB-01666891 | 4 | 1 | Unique |
| 608 | chr20 | 71,067,704 | 71,433,871 | 366,168 | loss | BTB-00572445 | BFGL-NGS-114380 | 12 | 1 | Unique |
| 609 | chr21 | 9,528,223 | 9,616,959 | 88,737 | both | BTA-76414-no-rs | BTB-00504830 | 4 | 4 | 0.77% |
| 610 | chr21 | 13,052,680 | 13,110,087 | 57,408 | gain | BTA-53438-no-rs | ARS-BFGL-NGS-11443 | 3 | 1 | Unique |
| 611 | chr21 | 15,633,331 | 15,705,393 | 72,063 | gain | ARS-BFGL-NGS-9271 | ARS-BFGL-NGS-100582 | 3 | 1 | Unique |
| 612 | chr21 | 17,398,300 | 17,531,298 | 132,999 | gain | DIAS-242 | Hapmap26740-BTA-155993 | 4 | 1 | Unique |
| 613 | chr21 | 20,194,633 | 20,389,414 | 194,782 | gain | ARS-BFGL-NGS-92246 | BTB-01787714 | 8 | 5 | 0.96% |
| 614 | chr21 | 29,893,539 | 29,966,098 | 72,560 | loss | BTB-01107683 | Hapmap49946-BTA-29235 | 3 | 6 | 1.15% |
| 615 | chr21 | 33,826,042 | 33,893,499 | 67,458 | loss | Hapmap24204-BTA-153183 | Hapmap32329-BTA-147054 | 3 | 2 | Multiple |
| 616 | chr21 | 37,111,472 | 37,233,645 | 122,174 | loss | BTB-01303836 | BTB-01303818 | 4 | 2 | Multiple |
| 617 | chr21 | 38,145,870 | 38,226,290 | 80,421 | gain | Hapmap53995-rs29024931 | BTB-02072074 | 3 | 1 | Unique |
| 618 | chr21 | 40,166,850 | 40,591,366 | 424,517 | both | BTA-52239-no-rs | Hapmap32050-BTA-135951 | 12 | 10 | 1.92% |
| 619 | chr21 | 41,692,311 | 42,051,380 | 359,070 | gain | BFGL-NGS-111692 | ARS-BFGL-NGS-43635 | 7 | 4 | 0.77% |
| 620 | chr21 | 42,244,509 | 42,357,276 | 112,768 | gain | Hapmap39342-BTA-52219 | ARS-BFGL-BAC-29181 | 3 | 1 | Unique |
| 621 | chr21 | 51,138,460 | 51,386,037 | 247,578 | gain | Hapmap32984-BTA-113902 | ARS-BFGL-NGS-18222 | 6 | 2 | Multiple |
| 622 | chr21 | 51,733,686 | 52,070,871 | 337,186 | loss | Hapmap40156-BTA-103844 | Hapmap27728-BTA-103839 | 4 | 3 | 0.58% |
| 623 | chr21 | 52,962,847 | 53,091,967 | 129,121 | loss | ARS-BFGL-NGS-44156 | ARS-BFGL-BAC-35190 | 4 | 1 | Unique |
| 624 | chr21 | 53,500,339 | 53,552,467 | 52,129 | loss | BTB-00649148 | ARS-BFGL-NGS-33035 | 3 | 1 | Unique |
| 625 | chr21 | 53,730,213 | 53,779,256 | 49,044 | loss | DIAS-290 | ARS-BFGL-NGS-19027 | 3 | 1 | Unique |
| 626 | chr21 | 68,965,986 | 69,094,176 | 128,191 | gain | BFGL-NGS-117917 | Hapmap34162-BES8_Contig487_1402 | 3 | 2 | Multiple |
| 627 | chr21 | 69,395,154 | 69,456,812 | 61,659 | gain | BFGL-NGS-115062 | ARS-BFGL-NGS-71969 | 3 | 7 | 1.34% |
| 628 | chr21 | 70,272,221 | 70,608,408 | 336,188 | both | ARS-BFGL-NGS-67767 | ARS-BFGL-NGS-2644 | 11 | 7 | 1.34% |
| 629 | chr21 | 70,931,906 | 71,248,668 | 316,763 | gain | ARS-BFGL-NGS-27992 | ARS-BFGL-NGS-6239 | 9 | 1 | Unique |
| 630 | chr22 | 6,226,915 | 6,450,949 | 224,035 | loss | ARS-BFGL-NGS-66672 | BFGL-NGS-119413 | 6 | 2 | Multiple |
| 631 | chr22 | 11,996,131 | 12,047,112 | 50,982 | gain | ARS-BFGL-NGS-109636 | BFGL-NGS-118910 | 3 | 1 | Unique |
| 632 | chr22 | 19,532,910 | 19,624,572 | 91,663 | gain | Hapmap46118-BTA-108252 | BFGL-NGS-116099 | 3 | 1 | Unique |
| 633 | chr22 | 35,115,756 | 35,250,658 | 134,903 | loss | ARS-BFGL-BAC-35711 | BTA-54170-no-rs | 5 | 11 | 2.11% |
| 634 | chr22 | 54,028,803 | 54,183,730 | 154,928 | loss | ARS-BFGL-NGS-51980 | Hapmap2744-BTA-121362 | 5 | 1 | Unique |
| 635 | chr22 | 59,387,581 | 59,802,619 | 415,039 | both | ARS-BFGL-NGS-30728 | ARS-BFGL-NGS-90643 | 10 | 4 | 0.77% |
| 636 | chr22 | 59,984,067 | 60,105,535 | 121,469 | loss | ARS-BFGL-NGS-16841 | ARS-BFGL-NGS-101683 | 5 | 2 | Multiple |
| 637 | chr22 | 60,243,916 | 60,376,752 | 132,837 | gain | Hapmap39470-BTA-121373 | Hapmap30583-BTA-157589 | 5 | 1 | Unique |
| 638 | chr22 | 61,199,665 | 61,401,870 | 202,206 | both | ARS-BFGL-NGS-105794 | ARS-BFGL-NGS-54240 | 8 | 4 | 0.77% |
| 639 | chr23 | 1,233,395 | 1,326,647 | 93,253 | gain | ARS-BFGL-NGS-11268 | ARS-BFGL-NGS-46133 | 3 | 2 | Multiple |
| 640 | chr23 | 4,557,388 | 4,647,111 | 89,724 | loss | Hapmap44551-BTA-55507 | ARS-BFGL-BAC-31235 | 4 | 2 | Multiple |
| 641 | chr23 | 6,089,999 | 6,176,821 | 86,823 | loss | BTA-57034-no-rs | Hapmap31772-BTA-144780 | 3 | 1 | Unique |
| 642 | chr23 | 12,938,089 | 13,081,503 | 143,415 | gain | ARS-BFGL-NGS-2043 | ARS-BFGL-NGS-42525 | 6 | 1 | Unique |
| 643 | chr23 | 16,321,314 | 16,536,674 | 215,361 | both | ARS-BFGL-NGS-101390 | ARS-BFGL-BAC-28908 | 4 | 4 | 0.77% |
| 644 | chr23 | 21,748,514 | 21,992,298 | 243,785 | both | Hapmap40178-BTA-55802 | Hapmap44684-BTA-55791 | 7 | 7 | 1.34% |
| 645 | chr23 | 25,250,595 | 25,426,985 | 176,391 | gain | ARS-BFGL-NGS-23762 | ARS-BFGL-NGS-54047 | 3 | 3 | 0.58% |
| 646 | chr23 | 27,644,218 | 27,887,914 | 243,697 | gain | ARS-BFGL-NGS-99242 | ARS-BFGL-NGS-16619 | 9 | 21 | 4.03% |
| 647 | chr23 | 28,444,435 | 28,552,178 | 107,744 | gain | BTA-111017-no-rs | ARS-BFGL-NGS-50654 | 3 | 1 | Unique |
| 648 | chr23 | 28,819,118 | 28,902,561 | 83,444 | gain | ARS-BFGL-NGS-105563 | ARS-BFGL-NGS-105966 | 3 | 2 | Multiple |
| 649 | chr23 | 30,343,750 | 30,468,111 | 124,362 | gain | Hapmap39414-BTA-56194 | ARS-BFGL-NGS-31776 | 5 | 1 | Unique |
| 650 | chr23 | 34,673,581 | 34,854,207 | 180,627 | gain | BTB-02011393 | ARS-BFGL-NGS-37691 | 4 | 1 | Unique |
| 651 | chr23 | 43,325,407 | 43,535,918 | 210,512 | both | ARS-BFGL-NGS-64113 | BTB-00871695 | 8 | 10 | 1.92% |
| 652 | chr23 | 44,458,259 | 44,680,105 | 221,847 | loss | BTA-56690-no-rs | BFGL-NGS-115605 | 6 | 3 | 0.58% |
| 653 | chr23 | 51,456,430 | 51,719,827 | 263,398 | loss | ARS-BFGL-NGS-17155 | BFGL-NGS-117465 | 9 | 1 | Unique |
| 654 | chr24 | 320,143 | 580,805 | 260,663 | gain | BTB-02076607 | BTB-00875757 | 7 | 18 | 3.45% |
| 655 | chr24 | 966,055 | 1,137,518 | 171,464 | loss | ARS-BFGL-NGS-10928 | ARS-BFGL-NGS-39061 | 5 | 1 | Unique |
| 656 | chr24 | 5,828,401 | 6,019,806 | 191,406 | loss | BTA-58814-no-rs | Hapmap36731-SCAFFOLD236442_391 | 7 | 4 | 0.77% |
| 657 | chr24 | 7,926,959 | 7,946,406 | 19,448 | gain | BTB-00880612 | Hapmap50408-BTA-58987 | 3 | 1 | Unique |
| 658 | chr24 | 9,251,349 | 9,355,676 | 104,328 | gain | ARS-BFGL-BAC-44134 | UA-IFASA-4296 | 4 | 2 | Multiple |
| 659 | chr24 | 12,351,291 | 12,415,668 | 64,378 | loss | BTA-57412-no-rs | Hapmap48000-BTA-57409 | 3 | 4 | 0.77% |
| 660 | chr24 | 13,250,354 | 13,366,494 | 116,141 | both | BTA-91085-no-rs | ARS-BFGL-NGS-86120 | 5 | 2 | Multiple |
| 661 | chr24 | 21,237,012 | 21,330,516 | 93,505 | gain | ARS-BFGL-NGS-106450 | ARS-BFGL-NGS-78497 | 4 | 1 | Unique |
| 662 | chr24 | 21,618,018 | 21,690,766 | 72,749 | loss | ARS-BFGL-NGS-61055 | ARS-BFGL-NGS-2686 | 3 | 2 | Multiple |
| 663 | chr24 | 28,083,770 | 28,244,323 | 160,554 | loss | BTA-109230-no-rs | Hapmap53299-rs29024075 | 7 | 3 | 0.58% |
| 664 | chr24 | 30,028,775 | 30,137,891 | 109,117 | gain | BTB-00885512 | BTB-00885423 | 3 | 1 | Unique |
| 665 | chr24 | 44,514,696 | 44,626,279 | 111,584 | loss | Hapmap51792-BTA-103575 | ARS-BFGL-NGS-67897 | 4 | 1 | Unique |
| 666 | chr24 | 51,508,537 | 51,643,856 | 135,320 | gain | ARS-BFGL-NGS-93376 | Hapmap42986-BTA-58601 | 3 | 1 | Unique |
| 667 | chr24 | 59,282,440 | 59,383,880 | 101,441 | gain | BFGL-NGS-114199 | ARS-BFGL-NGS-40380 | 4 | 2 | Multiple |
| 668 | chr24 | 62,406,299 | 62,475,205 | 68,907 | gain | BTB-01625019 | BFGL-NGS-119123 | 4 | 2 | Multiple |
| 669 | chr25 | 472,458 | 664,032 | 191,575 | loss | ARS-BFGL-NGS-12001 | ARS-BFGL-NGS-40627 | 6 | 2 | Multiple |
| 670 | chr25 | 736,464 | 813,455 | 76,992 | both | BFGL-NGS-119486 | ARS-BFGL-NGS-62237 | 4 | 6 | 1.15% |
| 671 | chr25 | 930,509 | 983,759 | 53,251 | loss | BFGL-NGS-115164 | ARS-BFGL-NGS-4009 | 3 | 1 | Unique |
| 672 | chr25 | 1,404,930 | 1,456,346 | 51,417 | both | ARS-BFGL-BAC-44214 | Hapmap31901-BTC-016378 | 4 | 3 | 0.58% |
| 673 | chr25 | 11,125,978 | 11,177,426 | 51,449 | loss | ARS-BFGL-NGS-102275 | ARS-BFGL-NGS-35331 | 3 | 2 | Multiple |
| 674 | chr25 | 19,315,456 | 19,762,712 | 447,257 | loss | BTA-59652-no-rs | ARS-BFGL-NGS-101615 | 7 | 1 | Unique |
| 675 | chr25 | 36,448,529 | 36,514,994 | 66,466 | loss | ARS-BFGL-NGS-13197 | ARS-BFGL-NGS-5763 | 4 | 1 | Unique |
| 676 | chr25 | 39,286,957 | 39,785,037 | 498,081 | loss | ARS-BFGL-NGS-105741 | ARS-BFGL-NGS-43022 | 13 | 3 | 0.58% |
| 677 | chr25 | 41,213,862 | 41,370,241 | 156,380 | gain | ARS-BFGL-BAC-47313 | Hapmap27068-BTC-031603 | 6 | 2 | Multiple |
| 678 | chr25 | 42,269,092 | 42,343,345 | 74,254 | loss | ARS-BFGL-NGS-21354 | ARS-BFGL-NGS-26308 | 3 | 1 | Unique |
| 679 | chr26 | 2,001,199 | 2,915,575 | 914,377 | loss | BTB-01734684 | Hapmap51817-BTA-22015 | 15 | 8 | 1.54% |
| 680 | chr26 | 3,010,798 | 3,139,603 | 128,806 | loss | Hapmap60383-rs29019554 | Hapmap44471-BTA-61074 | 4 | 1 | Unique |
| 681 | chr26 | 3,308,703 | 3,475,031 | 166,329 | loss | BTB-01967262 | BTB-01703941 | 5 | 1 | Unique |
| 682 | chr26 | 3,955,269 | 4,160,853 | 205,585 | loss | BTA-61571-no-rs | BTB-00920733 | 6 | 4 | 0.77% |
| 683 | chr26 | 4,558,899 | 4,939,413 | 380,515 | gain | BTB-00921166 | ARS-BFGL-NGS-92477 | 8 | 3 | 0.58% |
| 684 | chr26 | 5,364,728 | 5,504,271 | 139,544 | loss | ARS-BFGL-NGS-80925 | ARS-BFGL-NGS-13248 | 5 | 3 | 0.58% |
| 685 | chr26 | 13,601,655 | 13,697,600 | 95,946 | loss | ARS-BFGL-NGS-16205 | Hapmap38926-BTA-94783 | 4 | 2 | Multiple |
| 686 | chr26 | 16,862,899 | 16,973,274 | 110,376 | loss | BFGL-NGS-118205 | ARS-BFGL-NGS-107286 | 5 | 1 | Unique |
| 687 | chr26 | 18,335,079 | 18,416,740 | 81,662 | gain | Hapmap48023-BTA-62139 | Hapmap39788-BTA-87624 | 3 | 2 | Multiple |
| 688 | chr26 | 19,686,897 | 19,973,761 | 286,865 | loss | BTB-01389311 | BTB-01985838 | 7 | 2 | Multiple |
| 689 | chr26 | 24,401,843 | 24,531,763 | 129,921 | loss | ARS-BFGL-NGS-102845 | ARS-BFGL-NGS-1092 | 5 | 1 | Unique |
| 690 | chr26 | 25,828,973 | 25,982,293 | 153,321 | loss | Hapmap57754-ss46526149 | BFGL-NGS-114371 | 7 | 2 | Multiple |
| 691 | chr26 | 28,723,721 | 28,784,693 | 60,973 | loss | Hapmap38478-BTA-20824 | ARS-BFGL-NGS-55727 | 3 | 5 | 0.96% |
| 692 | chr26 | 29,427,835 | 29,698,221 | 270,387 | gain | BTB-01851763 | ARS-BFGL-NGS-16829 | 9 | 1 | Unique |
| 693 | chr26 | 33,234,819 | 33,306,959 | 72,141 | loss | Hapmap56942-ss46526051 | BTA-61189-no-rs | 3 | 1 | Unique |
| 694 | chr26 | 40,648,090 | 40,903,566 | 255,477 | loss | BTA-100861-no-rs | BFGL-NGS-112635 | 6 | 1 | Unique |
| 695 | chr26 | 48,672,512 | 48,751,188 | 78,677 | loss | BFGL-NGS-118574 | ARS-BFGL-NGS-76550 | 4 | 6 | 1.15% |
| 696 | chr26 | 50,517,416 | 50,680,745 | 163,330 | both | Hapmap46476-BTA-25480 | Hapmap41729-BTA-102219 | 4 | 3 | 0.58% |
| 697 | chr26 | 51,066,859 | 51,267,717 | 200,859 | loss | ARS-BFGL-NGS-97477 | BFGL-NGS-117200 | 6 | 1 | Unique |
| 698 | chr26 | 51,460,945 | 51,680,135 | 219,191 | loss | ARS-BFGL-NGS-101725 | ARS-BFGL-NGS-17431 | 8 | 1 | Unique |
| 699 | chr27 | 6,032,339 | 6,424,694 | 392,356 | both | BTB-02063702 | BTA-112884-no-rs | 7 | 10 | 1.92% |
| 700 | chr27 | 8,326,372 | 9,564,450 | 1,238,079 | both | BTB-01410000 | BTB-00950935 | 30 | 27 | 5.18% |
| 701 | chr27 | 11,185,038 | 11,365,955 | 180,918 | loss | BTB-00951788 | BTB-00951536 | 5 | 1 | Unique |
| 702 | chr27 | 12,330,184 | 12,426,617 | 96,434 | gain | ARS-BFGL-NGS-52875 | BTB-00954075 | 4 | 1 | Unique |
| 703 | chr27 | 15,141,168 | 15,321,772 | 180,605 | gain | Hapmap61080-rs29013547 | BTA-63226-no-rs | 8 | 2 | Multiple |
| 704 | chr27 | 18,164,172 | 18,292,384 | 128,213 | loss | ARS-BFGL-NGS-33046 | Hapmap24667-BTA-139303 | 3 | 2 | Multiple |
| 705 | chr27 | 27,345,322 | 27,402,642 | 57,321 | loss | BTB-00965793 | BTA-00436-rs29013593 | 3 | 1 | Unique |
| 706 | chr27 | 29,586,067 | 29,719,876 | 133,810 | loss | BFGL-NGS-116607 | ARS-BFGL-NGS-21155 | 3 | 7 | 1.34% |
| 707 | chr27 | 37,120,275 | 37,259,754 | 139,480 | gain | ARS-BFGL-NGS-92338 | DIAS-332 | 5 | 1 | Unique |
| 708 | chr27 | 39,061,885 | 39,204,962 | 143,078 | gain | DIAS-335 | DIAS-336 | 3 | 2 | Multiple |
| 709 | chr27 | 44,851,308 | 45,003,136 | 151,829 | gain | Hapmap38844-BTA-63080 | ARS-BFGL-NGS-239 | 4 | 1 | Unique |
| 710 | chr27 | 45,274,514 | 45,368,987 | 94,474 | gain | BTB-00971327 | BTB-01682090 | 4 | 2 | Multiple |
| 711 | chr28 | 2,275,570 | 2,716,997 | 441,428 | both | BTB-01590721 | BTB-02011628 | 8 | 38 | 7.29% |
| 712 | chr28 | 4,162,317 | 4,235,517 | 73,201 | gain | ARS-BFGL-NGS-59257 | ARS-BFGL-NGS-107787 | 3 | 4 | 0.77% |
| 713 | chr28 | 11,082,523 | 11,196,379 | 113,857 | loss | Hapmap39009-BTA-121529 | ARS-BFGL-NGS-4626 | 3 | 2 | Multiple |
| 714 | chr28 | 11,897,521 | 12,030,121 | 132,601 | loss | Hapmap55640-rs29014036 | Hapmap50192-BTA-104693 | 5 | 1 | Unique |
| 715 | chr28 | 21,101,833 | 21,292,836 | 191,004 | loss | BTB-02080610 | BTB-01171367 | 4 | 1 | Unique |
| 716 | chr28 | 21,516,493 | 21,658,311 | 141,819 | loss | BTB-01171634 | BTB-01522449 | 3 | 1 | Unique |
| 717 | chr28 | 21,845,568 | 22,226,165 | 380,598 | both | ARS-BFGL-NGS-36180 | BTB-01650236 | 10 | 5 | 0.96% |
| 718 | chr28 | 23,702,232 | 23,764,335 | 62,104 | loss | Hapmap27166-BTA-139945 | ARS-BFGL-NGS-351 | 4 | 3 | 0.58% |
| 719 | chr28 | 24,292,229 | 24,386,413 | 94,185 | gain | ARS-BFGL-NGS-87034 | ARS-BFGL-NGS-15201 | 3 | 2 | Multiple |
| 720 | chr28 | 25,060,861 | 25,244,340 | 183,480 | loss | BTA-63757-no-rs | Hapmap44654-BTA-63754 | 4 | 3 | 0.58% |
| 721 | chr28 | 25,352,987 | 25,542,150 | 189,164 | gain | Hapmap44554-BTA-63753 | ARS-BFGL-NGS-87782 | 5 | 6 | 1.15% |
| 722 | chr28 | 36,592,156 | 36,776,061 | 183,906 | both | BTA-24338-no-rs | BTB-01469727 | 4 | 7 | 1.34% |
| 723 | chr28 | 38,026,506 | 38,136,070 | 109,565 | loss | BFGL-NGS-115768 | BTB-01713276 | 4 | 6 | 1.15% |
| 724 | chr28 | 45,676,164 | 46,194,755 | 518,592 | loss | ARS-BFGL-NGS-14462 | Hapmap43005-BTA-64606 | 13 | 2 | Multiple |
| 725 | chr29 | 46,338 | 454,112 | 407,775 | loss | BTB-01664545 | ARS-BFGL-NGS-82297 | 5 | 3 | 0.58% |
| 726 | chr29 | 630,087 | 724,198 | 94,112 | loss | Hapmap53898-rs29026969 | Hapmap58534-rs29018685 | 3 | 1 | Unique |
| 727 | chr29 | 2,227,690 | 2,369,574 | 141,885 | gain | Hapmap44268-BTA-66621 | ARS-BFGL-NGS-1591 | 5 | 1 | Unique |
| 728 | chr29 | 5,348,843 | 5,750,781 | 401,939 | gain | ARS-BFGL-NGS-92221 | BTB-02034613 | 7 | 4 | 0.77% |
| 729 | chr29 | 15,213,746 | 15,520,726 | 306,981 | both | BTA-117784-no-rs | Hapmap42430-BTA-112191 | 5 | 3 | 0.58% |
| 730 | chr29 | 15,955,906 | 16,160,945 | 205,040 | loss | BFGL-NGS-115782 | BFGL-NGS-118102 | 4 | 4 | 0.77% |
| 731 | chr29 | 21,930,571 | 21,987,120 | 56,550 | loss | ARS-BFGL-NGS-18922 | Hapmap36817-SCAFFOLD245829_8774 | 3 | 1 | Unique |
| 732 | chr29 | 24,668,066 | 24,774,389 | 106,324 | loss | BFGL-NGS-119138 | Hapmap43319-BTA-65094 | 3 | 1 | Unique |
| 733 | chr29 | 27,614,094 | 28,170,104 | 556,011 | both | BTA-111991-no-rs | ARS-BFGL-NGS-17874 | 17 | 37 | 7.10% |
| 734 | chr29 | 28,849,610 | 28,967,249 | 117,640 | loss | BTB-01017180 | ARS-BFGL-NGS-79220 | 5 | 1 | Unique |
| 735 | chr29 | 37,237,891 | 37,412,121 | 174,231 | gain | BTA-65647-no-rs | BTA-65640-no-rs | 5 | 19 | 3.65% |
| 736 | chr29 | 38,845,455 | 39,033,445 | 187,991 | gain | ARS-BFGL-NGS-97760 | ARS-BFGL-NGS-22832 | 3 | 1 | Unique |
| 737 | chr29 | 39,706,114 | 39,782,539 | 76,426 | gain | ARS-BFGL-NGS-22514 | ARS-BFGL-NGS-23135 | 3 | 1 | Unique |
| 738 | chr29 | 42,921,883 | 42,985,739 | 63,857 | loss | ARS-BFGL-NGS-29002 | ARS-BFGL-NGS-101195 | 3 | 1 | Unique |
| 739 | chr29 | 44,243,444 | 44,292,035 | 48,592 | gain | ARS-BFGL-NGS-82048 | Hapmap36151-SCAFFOLD96691_12353 | 3 | 4 | 0.77% |
| 740 | chr29 | 46,703,510 | 46,796,930 | 93,421 | gain | BTB-01036181 | Hapmap47369-BTA-66337 | 4 | 1 | Unique |
| 741 | chr29 | 46,883,278 | 47,054,342 | 171,065 | loss | ARS-BFGL-NGS-65789 | ARS-BFGL-NGS-108161 | 6 | 1 | Unique |
| 742 | chr29 | 47,914,950 | 48,054,630 | 139,681 | both | ARS-BFGL-NGS-86466 | ARS-BFGL-NGS-42102 | 6 | 7 | 1.34% |
| 743 | chr29 | 49,906,123 | 51,502,868 | 1,596,746 | both | ARS-BFGL-NGS-109317 | ARS-BFGL-NGS-29340 | 39 | 14 | 2.69% |

**Table S5. The comparison of CNVs from 39 trios using three CNV calling algorithms: individual-calling, posterior-calling and joint-calling.**

|  | ***Individual-calling*** | | | ***Posterior-calling*** | | | ***Joint-calling*** | | |
| --- | --- | --- | --- | --- | --- | --- | --- | --- | --- |
| **Trio** | **No CNVs** | **Total Length** | **No SNP** | **No CNVs** | **Total Length** | **No SNP** | **No CNVs** | **Total Length** | **No SNP** |
| 1 | 34 | 5,346,449 | 169 | 59 | 8,910,667 | 279 | 74 | 9,676,855 | 298 |
| 2 | 7 | 781,290 | 25 | 16 | 1,706,829 | 53 | 16 | 1,458,322 | 56 |
| 3 | 9 | 1,050,542 | 34 | 14 | 1,689,452 | 50 | 14 | 1,945,916 | 57 |
| 4 | 15 | 2,379,163 | 73 | 20 | 3,580,083 | 100 | 26 | 3,545,297 | 107 |
| 5 | 16 | 2,340,948 | 76 | 27 | 3,976,736 | 122 | 66 | 7,858,769 | 252 |
| 6 | 31 | 4,004,809 | 131 | 51 | 6,252,106 | 208 | 50 | 5,888,308 | 182 |
| 7 | 25 | 3,640,613 | 119 | 43 | 6,100,384 | 197 | 70 | 9,244,645 | 269 |
| 8 | 13 | 2,138,226 | 72 | 16 | 2,607,038 | 89 | 15 | 2,235,063 | 77 |
| 9 | 13 | 2,203,436 | 75 | 17 | 2,720,835 | 92 | 16 | 2,851,952 | 89 |
| 10 | 12 | 1,160,721 | 43 | 15 | 1,428,223 | 53 | 19 | 1,574,746 | 64 |
| 11 | 23 | 4,170,217 | 128 | 31 | 5,715,628 | 174 | 42 | 6,076,516 | 170 |
| 12 | 19 | 2,265,694 | 76 | 31 | 3,485,146 | 123 | 32 | 3,374,170 | 119 |
| 13 | 35 | 7,255,101 | 207 | 38 | 7,560,623 | 218 | 40 | 7,243,819 | 197 |
| 14 | 23 | 3,445,485 | 108 | 43 | 6,353,042 | 186 | 80 | 9,982,267 | 290 |
| 15 | 21 | 3,271,500 | 117 | 34 | 5,119,331 | 179 | 56 | 6,705,063 | 216 |
| 16 | 23 | 3,642,268 | 116 | 38 | 6,387,591 | 199 | 73 | 8,418,038 | 290 |
| 17 | 17 | 2,391,225 | 77 | 25 | 3,548,746 | 108 | 28 | 3,556,641 | 104 |
| 18 | 20 | 1,929,002 | 72 | 33 | 3,579,703 | 122 | 32 | 3,809,364 | 116 |
| 19 | 17 | 2,903,595 | 79 | 23 | 3,445,084 | 99 | 24 | 3,240,298 | 95 |
| 20 | 14 | 2,194,558 | 70 | 24 | 3,630,373 | 109 | 18 | 2,447,775 | 78 |
| 21 | 12 | 1,948,514 | 57 | 20 | 3,041,978 | 94 | 20 | 3,946,472 | 97 |
| 22 | 11 | 1,446,726 | 47 | 20 | 2,663,728 | 81 | 30 | 3,929,109 | 116 |
| 23 | 8 | 1,096,721 | 30 | 10 | 1,291,295 | 36 | 11 | 1,444,777 | 38 |
| 24 | 7 | 624,035 | 26 | 11 | 1,017,194 | 43 | 20 | 2,534,052 | 78 |
| 25 | 11 | 1,228,912 | 45 | 19 | 1,938,175 | 74 | 21 | 1,986,398 | 79 |
| 26 | 8 | 1,011,833 | 35 | 12 | 1,331,764 | 49 | 7 | 819,912 | 23 |
| 27 | 21 | 5,840,664 | 129 | 33 | 9,210,100 | 209 | 59 | 11,770,580 | 273 |
| 28 | 14 | 2,302,251 | 65 | 21 | 3,272,411 | 90 | 17 | 2,574,603 | 83 |
| 29 | 20 | 2,935,202 | 91 | 32 | 4,956,898 | 142 | 43 | 4,438,103 | 148 |
| 30 | 16 | 3,425,266 | 89 | 19 | 4,150,327 | 105 | 23 | 5,036,717 | 109 |
| 31 | 11 | 2,608,201 | 65 | 15 | 2,964,179 | 77 | 13 | 2,745,681 | 71 |
| 32 | 15 | 2,906,941 | 71 | 20 | 3,412,759 | 91 | 18 | 2,290,818 | 69 |
| 33 | 42 | 9,306,053 | 253 | 48 | 10,958,010 | 289 | 46 | 8,996,435 | 246 |
| 34 | 24 | 3,858,630 | 120 | 28 | 4,524,732 | 144 | 30 | 4,635,034 | 146 |
| 35 | 9 | 1,644,651 | 47 | 13 | 2,070,724 | 63 | 10 | 1,637,374 | 47 |
| 36 | 13 | 3,177,437 | 87 | 16 | 3,704,975 | 102 | 20 | 4,265,922 | 108 |
| 37 | 23 | 3,396,370 | 105 | 33 | 5,476,045 | 164 | 33 | 4,951,802 | 145 |
| 38 | 11 | 1,536,799 | 53 | 18 | 2,574,117 | 87 | 27 | 3,777,004 | 110 |
| 39 | 21 | 5,650,890 | 149 | 33 | 7,413,125 | 201 | 37 | 7,306,054 | 202 |
| Total | 684 | 114,460,938 | 3431 | 1019 | 163,770,156 | 4901 | 1276 | 180,220,671 | 5314 |

**Table S6. Gene contents of cattle CNV regions. See Additional file 2: Table S6.xls.**

**Table S7. Outgroup** CNV regions and their frequencies.

| ***No*** | ***Chr*** | ***Start*** | ***End*** | ***Length*** | ***Type*** | ***StartSNP*** | ***EndSNP*** | ***No. SNP*** | ***Count*** | ***Frequency*** |
| --- | --- | --- | --- | --- | --- | --- | --- | --- | --- | --- |
| 1 | chr1 | 4,070,975 | 5,303,426 | 1,232,452 | loss | BTB-01747944 | BTB-00003715 | 30 | 2 | Multiple |
| 2 | chr1 | 15,134,284 | 18,771,895 | 3,637,612 | loss | BTB-01224604 | Hapmap47551-BTA-25902 | 78 | 2 | Multiple |
| 3 | chr1 | 19,802,294 | 27,732,833 | 7,930,540 | loss | BTA-119061-no-rs | Hapmap29626-BTA-155192 | 168 | 6 | 33.33% |
| 4 | chr1 | 29,885,948 | 31,611,906 | 1,725,959 | loss | ARS-BFGL-NGS-74692 | BTB-01335754 | 38 | 3 | 16.67% |
| 5 | chr1 | 32,423,821 | 34,085,800 | 1,661,980 | loss | BTB-02018681 | BTB-00902144 | 38 | 3 | 16.67% |
| 6 | chr1 | 39,384,792 | 42,813,944 | 3,429,153 | loss | ARS-BFGL-NGS-85768 | BTB-01136210 | 73 | 5 | 27.78% |
| 7 | chr1 | 45,512,563 | 45,647,138 | 134,576 | loss | ARS-BFGL-NGS-57355 | BTB-00021257 | 4 | 2 | Multiple |
| 8 | chr1 | 49,221,200 | 49,403,484 | 182,285 | loss | ARS-BFGL-NGS-50906 | ARS-BFGL-NGS-41264 | 7 | 1 | Unique |
| 9 | chr1 | 49,660,351 | 51,006,140 | 1,345,790 | loss | Hapmap26719-BTA-151471 | Hapmap41716-BTA-100042 | 35 | 5 | 27.78% |
| 10 | chr1 | 53,247,300 | 54,738,190 | 1,490,891 | loss | ARS-BFGL-NGS-78259 | Hapmap48975-BTA-99363 | 42 | 2 | Multiple |
| 11 | chr1 | 60,908,248 | 61,216,644 | 308,397 | loss | BFGL-NGS-115239 | BTA-23353-no-rs | 7 | 4 | 22.22% |
| 12 | chr1 | 83,288,915 | 83,402,940 | 114,026 | loss | BTB-01187540 | BTB-01187501 | 5 | 5 | 27.78% |
| 13 | chr1 | 89,677,286 | 89,771,787 | 94,502 | loss | Hapmap54455-rs29016036 | Hapmap47817-BTA-104461 | 4 | 2 | Multiple |
| 14 | chr1 | 95,898,816 | 96,864,503 | 965,688 | loss | Hapmap42235-BTA-50604 | ARS-BFGL-NGS-25039 | 25 | 1 | Unique |
| 15 | chr1 | 101,253,497 | 101,422,074 | 168,578 | loss | ARS-BFGL-NGS-29680 | BTB-01568299 | 3 | 2 | Multiple |
| 16 | chr1 | 103,004,836 | 106,367,469 | 3,362,634 | loss | BTA-115500-no-rs | BTB-02095892 | 71 | 2 | Multiple |
| 17 | chr1 | 116,951,612 | 117,879,706 | 928,095 | loss | ARS-BFGL-NGS-67613 | Hapmap24434-BTA-48171 | 19 | 1 | Unique |
| 18 | chr1 | 121,845,903 | 121,931,242 | 85,340 | gain | BTA-22577-no-rs | ARS-BFGL-NGS-64650 | 4 | 2 | Multiple |
| 19 | chr1 | 128,361,423 | 128,511,630 | 150,208 | loss | BTA-27135-no-rs | Hapmap51248-BTA-51337 | 6 | 2 | Multiple |
| 20 | chr1 | 134,547,943 | 135,008,984 | 461,042 | loss | BTB-02025691 | BTB-00061551 | 11 | 1 | Unique |
| 21 | chr1 | 141,355,474 | 141,413,935 | 58,462 | loss | ARS-BFGL-NGS-13638 | BTB-00065539 | 3 | 1 | Unique |
| 22 | chr1 | 147,670,375 | 147,795,528 | 125,154 | loss | Hapmap52800-ss46526105 | ARS-BFGL-NGS-53898 | 4 | 2 | Multiple |
| 23 | chr1 | 154,461,110 | 154,573,212 | 112,103 | loss | BTB-00071918 | ARS-BFGL-NGS-16576 | 4 | 2 | Multiple |
| 24 | chr2 | 1,480,031 | 1,550,331 | 70,301 | gain | BTB-01578175 | ARS-BFGL-NGS-31985 | 4 | 2 | Multiple |
| 25 | chr2 | 2,114,586 | 2,241,157 | 126,572 | loss | ARS-BFGL-BAC-35148 | BFGL-NGS-113652 | 3 | 2 | Multiple |
| 26 | chr2 | 8,661,058 | 11,060,395 | 2,399,338 | loss | BTB-01111066 | Hapmap44041-BTA-23382 | 59 | 3 | 16.67% |
| 27 | chr2 | 21,744,159 | 23,352,853 | 1,608,695 | loss | Hapmap59358-rs29024697 | BTB-00084309 | 46 | 4 | 22.22% |
| 28 | chr2 | 24,360,937 | 24,691,213 | 330,277 | loss | BTB-00085877 | Hapmap40436-BTA-46804 | 9 | 1 | Unique |
| 29 | chr2 | 27,107,785 | 27,338,225 | 230,441 | loss | BTB-00091568 | Hapmap52660-ss46526812 | 8 | 1 | Unique |
| 30 | chr2 | 29,540,904 | 30,624,251 | 1,083,348 | loss | ARS-BFGL-BAC-31010 | Hapmap49514-BTA-18186 | 31 | 1 | Unique |
| 31 | chr2 | 31,317,703 | 33,538,423 | 2,220,721 | loss | ARS-BFGL-BAC-30773 | BTB-01549619 | 72 | 4 | 22.22% |
| 32 | chr2 | 38,125,358 | 38,577,906 | 452,549 | loss | Hapmap42250-BTA-55589 | ARS-BFGL-NGS-75 | 9 | 1 | Unique |
| 33 | chr2 | 40,671,382 | 40,755,586 | 84,205 | loss | Hapmap32557-BTA-133920 | Hapmap55542-rs29024346 | 4 | 2 | Multiple |
| 34 | chr2 | 41,910,942 | 42,045,139 | 134,198 | loss | ARS-BFGL-NGS-23722 | ARS-BFGL-NGS-92476 | 5 | 1 | Unique |
| 35 | chr2 | 42,132,665 | 42,191,064 | 58,400 | loss | BTA-08061-no-rs | Hapmap43158-BTA-113479 | 3 | 1 | Unique |
| 36 | chr2 | 43,141,034 | 43,398,479 | 257,446 | loss | Hapmap48777-BTA-47434 | Hapmap44080-BTA-47449 | 10 | 6 | 33.33% |
| 37 | chr2 | 54,145,719 | 58,842,740 | 4,697,022 | loss | Hapmap42227-BTA-47643 | Hapmap28102-BTA-152636 | 130 | 6 | 33.33% |
| 38 | chr2 | 59,718,306 | 59,901,005 | 182,700 | loss | BTA-19224-no-rs | BTB-00099725 | 6 | 3 | 16.67% |
| 39 | chr2 | 64,244,900 | 64,797,702 | 552,803 | loss | BFGL-NGS-109852 | Hapmap52390-rs29021802 | 13 | 1 | Unique |
| 40 | chr2 | 70,475,936 | 71,664,678 | 1,188,743 | loss | BTB-01730399 | ARS-BFGL-NGS-47229 | 33 | 1 | Unique |
| 41 | chr2 | 72,573,878 | 72,754,250 | 180,373 | loss | BTB-00121383 | ARS-BFGL-NGS-62901 | 8 | 3 | 16.67% |
| 42 | chr2 | 79,660,911 | 80,018,457 | 357,547 | loss | BTB-01062977 | Hapmap50277-BTA-20931 | 12 | 1 | Unique |
| 43 | chr2 | 80,059,142 | 80,118,827 | 59,686 | loss | ARS-BFGL-NGS-106237 | BTB-01313103 | 3 | 1 | Unique |
| 44 | chr2 | 82,803,279 | 82,865,615 | 62,337 | loss | BFGL-NGS-110862 | Hapmap33049-BTA-153946 | 3 | 1 | Unique |
| 45 | chr2 | 91,440,102 | 91,815,686 | 375,585 | loss | Hapmap23329-BTA-157704 | ARS-BFGL-NGS-94983 | 7 | 1 | Unique |
| 46 | chr2 | 103,335,636 | 103,573,252 | 237,617 | loss | ARS-BFGL-NGS-101219 | UA-IFASA-4555 | 4 | 2 | Multiple |
| 47 | chr2 | 114,889,415 | 115,024,545 | 135,131 | loss | ARS-BFGL-NGS-76658 | ARS-USMARC-Parent-DQ786757-rs29019900 | 4 | 3 | 16.67% |
| 48 | chr2 | 124,216,287 | 124,293,575 | 77,289 | loss | ARS-BFGL-NGS-89829 | BFGL-NGS-114262 | 4 | 1 | Unique |
| 49 | chr2 | 139,892,519 | 140,331,355 | 438,837 | loss | ARS-BFGL-NGS-38259 | ARS-BFGL-NGS-14555 | 16 | 1 | Unique |
| 50 | chr3 | 743 | 227,160 | 226,418 | loss | BTB-01996597 | BTB-01660449 | 6 | 4 | 22.22% |
| 51 | chr3 | 7,362,256 | 7,649,578 | 287,323 | loss | BFGL-NGS-113472 | BTA-69126-no-rs | 7 | 1 | Unique |
| 52 | chr3 | 24,484,313 | 24,599,313 | 115,001 | loss | BTA-93841-no-rs | ARS-BFGL-NGS-103648 | 4 | 2 | Multiple |
| 53 | chr3 | 25,354,125 | 25,405,073 | 50,949 | loss | BTA-86312-no-rs | BTA-66928-no-rs | 3 | 2 | Multiple |
| 54 | chr3 | 37,010,042 | 37,156,761 | 146,720 | loss | BTA-88149-no-rs | BTA-67536-no-rs | 5 | 1 | Unique |
| 55 | chr3 | 42,561,047 | 42,763,614 | 202,568 | loss | Hapmap51027-BTA-67630 | INRA-359 | 4 | 2 | Multiple |
| 56 | chr3 | 42,953,153 | 43,502,278 | 549,126 | loss | BTA-93165-no-rs | Hapmap55075-rs29013986 | 17 | 2 | Multiple |
| 57 | chr3 | 47,057,515 | 47,166,786 | 109,272 | loss | ARS-BFGL-NGS-57015 | Hapmap42291-BTA-67678 | 3 | 2 | Multiple |
| 58 | chr3 | 56,416,977 | 56,615,223 | 198,247 | loss | Hapmap43965-BTA-89883 | INRA-297 | 8 | 1 | Unique |
| 59 | chr3 | 64,090,602 | 64,834,128 | 743,527 | loss | Hapmap24571-BTA-154685 | Hapmap39465-BTA-116795 | 22 | 1 | Unique |
| 60 | chr3 | 67,315,495 | 69,118,846 | 1,803,352 | loss | BTB-01215937 | BTA-118190-no-rs | 48 | 5 | 27.78% |
| 61 | chr3 | 69,920,342 | 70,170,783 | 250,442 | loss | Hapmap49033-BTA-115567 | Hapmap56885-rs29027552 | 8 | 1 | Unique |
| 62 | chr3 | 73,221,021 | 73,329,640 | 108,620 | loss | BTB-01098205 | BTB-01451979 | 5 | 2 | Multiple |
| 63 | chr3 | 73,697,858 | 74,512,971 | 815,114 | loss | Hapmap51482-BTA-68303 | Hapmap51849-BTA-68314 | 25 | 2 | Multiple |
| 64 | chr3 | 80,516,328 | 81,172,893 | 656,566 | loss | Hapmap45681-BTA-68349 | BTB-01542625 | 18 | 1 | Unique |
| 65 | chr3 | 81,385,248 | 82,395,580 | 1,010,333 | loss | ARS-BFGL-NGS-26551 | Hapmap35132-BES4_Contig245_660 | 29 | 1 | Unique |
| 66 | chr3 | 89,893,858 | 90,335,697 | 441,840 | loss | ARS-BFGL-NGS-1567 | BTA-68545-no-rs | 13 | 1 | Unique |
| 67 | chr3 | 99,683,121 | 99,983,310 | 300,190 | loss | ARS-BFGL-NGS-27702 | INRA-178 | 8 | 3 | 16.67% |
| 68 | chr3 | 101,424,752 | 101,666,585 | 241,834 | loss | BTB-00145926 | BTB-00146049 | 7 | 2 | Multiple |
| 69 | chr3 | 104,612,143 | 105,007,297 | 395,155 | loss | ARS-BFGL-NGS-95604 | BFGL-NGS-110586 | 12 | 4 | 22.22% |
| 70 | chr3 | 115,956,141 | 116,505,274 | 549,134 | loss | ARS-BFGL-NGS-16026 | Hapmap53978-rs29024239 | 16 | 2 | Multiple |
| 71 | chr3 | 119,741,148 | 120,001,314 | 260,167 | loss | Hapmap33076-BTA-163309 | ARS-BFGL-NGS-104342 | 5 | 1 | Unique |
| 72 | chr3 | 121,002,313 | 121,437,514 | 435,202 | loss | BFGL-NGS-116451 | BTB-00157794 | 15 | 3 | 16.67% |
| 73 | chr3 | 124,821,625 | 127,719,187 | 2,897,563 | loss | Hapmap48056-BTA-69793 | ARS-BFGL-NGS-70541 | 84 | 2 | Multiple |
| 74 | chr3 | 127,793,158 | 127,908,629 | 115,472 | loss | ARS-BFGL-NGS-57811 | ARS-BFGL-NGS-19124 | 5 | 1 | Unique |
| 75 | chr4 | 6,263,127 | 7,038,463 | 775,337 | loss | ARS-BFGL-NGS-104086 | ARS-BFGL-NGS-82239 | 16 | 3 | 16.67% |
| 76 | chr4 | 10,704,148 | 10,850,193 | 146,046 | loss | ARS-BFGL-NGS-54920 | Hapmap24260-BTA-155392 | 4 | 3 | 16.67% |
| 77 | chr4 | 17,446,530 | 19,718,653 | 2,272,124 | loss | Hapmap51543-BTA-108077 | BTB-01238492 | 47 | 1 | Unique |
| 78 | chr4 | 20,361,118 | 20,531,145 | 170,028 | loss | ARS-BFGL-NGS-4793 | Hapmap59250-rs29020746 | 3 | 2 | Multiple |
| 79 | chr4 | 21,506,497 | 21,807,441 | 300,945 | loss | Hapmap50078-BTA-71995 | BTA-72259-no-rs | 9 | 1 | Unique |
| 80 | chr4 | 23,032,572 | 23,742,638 | 710,067 | loss | BTB-01054610 | BTA-72579-no-rs | 17 | 1 | Unique |
| 81 | chr4 | 35,249,217 | 41,084,886 | 5,835,670 | loss | BTB-01793118 | Hapmap43454-BTA-108936 | 130 | 4 | 22.22% |
| 82 | chr4 | 43,321,794 | 43,608,739 | 286,946 | loss | BTB-00178816 | BTB-00179295 | 11 | 1 | Unique |
| 83 | chr4 | 49,185,603 | 49,265,709 | 80,107 | loss | BTA-70504-no-rs | Hapmap43660-BTA-70498 | 3 | 2 | Multiple |
| 84 | chr4 | 54,019,973 | 57,121,588 | 3,101,616 | loss | BTB-00185894 | BTB-00186521 | 65 | 2 | Multiple |
| 85 | chr4 | 60,549,560 | 61,011,870 | 462,311 | loss | BTB-01263932 | BTA-70765-no-rs | 13 | 1 | Unique |
| 86 | chr4 | 69,656,393 | 69,828,238 | 171,846 | loss | BFGL-NGS-116611 | BTB-00195350 | 5 | 1 | Unique |
| 87 | chr4 | 76,024,190 | 76,191,527 | 167,338 | loss | BTB-01965166 | BTB-02044451 | 3 | 2 | Multiple |
| 88 | chr4 | 85,644,103 | 85,823,298 | 179,196 | loss | Hapmap30589-BTA-158871 | ARS-BFGL-NGS-25242 | 6 | 1 | Unique |
| 89 | chr4 | 86,062,628 | 87,511,031 | 1,448,404 | loss | BTB-01379114 | Hapmap55226-rs29009595 | 34 | 1 | Unique |
| 90 | chr4 | 93,400,261 | 94,477,476 | 1,077,216 | loss | BTA-109007-no-rs | ARS-BFGL-NGS-12375 | 25 | 3 | 16.67% |
| 91 | chr4 | 111,545,101 | 112,133,333 | 588,233 | loss | BTA-121657-no-rs | ARS-BFGL-NGS-73118 | 14 | 3 | 16.67% |
| 92 | chr4 | 120,699,997 | 120,930,703 | 230,707 | loss | ARS-BFGL-NGS-22183 | BFGL-NGS-115924 | 7 | 1 | Unique |
| 93 | chr4 | 123,973,622 | 124,038,308 | 64,687 | loss | ARS-BFGL-NGS-31650 | Hapmap32572-BTA-142704 | 3 | 2 | Multiple |
| 94 | chr5 | 8,534 | 288,685 | 280,152 | gain | BTB-01498887 | BTB-01711340 | 9 | 2 | Multiple |
| 95 | chr5 | 3,563,151 | 4,649,042 | 1,085,892 | loss | Hapmap44365-BTA-28169 | BTB-00215584 | 29 | 4 | 22.22% |
| 96 | chr5 | 5,665,518 | 5,720,724 | 55,207 | loss | ARS-BFGL-NGS-106198 | Hapmap50082-BTA-73223 | 3 | 2 | Multiple |
| 97 | chr5 | 8,455,044 | 8,565,017 | 109,974 | loss | BTB-01359812 | BTB-01507839 | 5 | 1 | Unique |
| 98 | chr5 | 9,713,680 | 10,052,609 | 338,930 | gain | BTB-00235875 | BTB-00235905 | 4 | 1 | Unique |
| 99 | chr5 | 10,657,977 | 13,197,167 | 2,539,191 | loss | BFGL-NGS-118997 | Hapmap55297-rs29017245 | 60 | 2 | Multiple |
| 100 | chr5 | 16,862,261 | 17,745,377 | 883,117 | loss | Hapmap58532-rs29018522 | Hapmap60079-rs29021032 | 27 | 2 | Multiple |
| 101 | chr5 | 22,708,445 | 23,245,067 | 536,623 | loss | BTA-20092-no-rs | Hapmap26407-BTA-142865 | 7 | 1 | Unique |
| 102 | chr5 | 43,286,974 | 44,232,398 | 945,425 | loss | Hapmap55164-rs29016423 | ARS-BFGL-NGS-19252 | 31 | 5 | 27.78% |
| 103 | chr5 | 45,926,535 | 45,992,976 | 66,442 | loss | BTB-01255930 | BTB-01255969 | 3 | 2 | Multiple |
| 104 | chr5 | 62,264,656 | 62,856,947 | 592,292 | loss | BTB-02026405 | ARS-BFGL-NGS-35956 | 5 | 3 | 16.67% |
| 105 | chr5 | 63,605,941 | 63,933,743 | 327,803 | loss | BTB-02067717 | Hapmap26488-BTA-164056 | 3 | 2 | Multiple |
| 106 | chr5 | 74,978,375 | 75,141,726 | 163,352 | loss | BTA-73842-no-rs | Hapmap28985-BTA-73836 | 6 | 1 | Unique |
| 107 | chr5 | 81,075,178 | 81,312,782 | 237,605 | loss | BTA-74036-no-rs | ARS-BFGL-NGS-73201 | 4 | 2 | Multiple |
| 108 | chr5 | 83,624,593 | 84,177,738 | 553,146 | loss | BTA-74159-no-rs | BTA-11244-rs29017007 | 9 | 2 | Multiple |
| 109 | chr5 | 92,172,835 | 92,258,527 | 85,693 | loss | BFGL-NGS-115024 | BTA-123014-no-rs | 3 | 2 | Multiple |
| 110 | chr5 | 98,260,277 | 98,456,164 | 195,888 | loss | Hapmap38581-BTA-15564 | BTA-15560-no-rs | 4 | 1 | Unique |
| 111 | chr5 | 98,950,819 | 99,378,352 | 427,534 | loss | Hapmap43797-BTA-17694 | Hapmap34380-BES4_Contig293_1272 | 8 | 1 | Unique |
| 112 | chr5 | 113,682,010 | 113,734,416 | 52,407 | loss | ARS-BFGL-NGS-25555 | Hapmap57466-rs29018274 | 3 | 2 | Multiple |
| 113 | chr5 | 116,152,846 | 116,218,904 | 66,059 | loss | Hapmap52801-ss46526219 | ARS-BFGL-NGS-97512 | 4 | 1 | Unique |
| 114 | chr5 | 116,252,572 | 116,362,061 | 109,490 | loss | ARS-BFGL-NGS-627 | ARS-BFGL-NGS-106105 | 4 | 1 | Unique |
| 115 | chr5 | 123,381,180 | 123,598,295 | 217,116 | gain | ARS-BFGL-NGS-89264 | ARS-BFGL-NGS-21635 | 8 | 2 | Multiple |
| 116 | chr5 | 123,626,393 | 123,961,828 | 335,436 | loss | ARS-BFGL-NGS-16699 | ARS-BFGL-NGS-107617 | 12 | 2 | Multiple |
| 117 | chr5 | 124,644,788 | 125,804,605 | 1,159,818 | loss | ARS-BFGL-NGS-107534 | ARS-BFGL-NGS-100728 | 35 | 3 | 16.67% |
| 118 | chr6 | 264,247 | 850,596 | 586,350 | loss | BTB-01573053 | Hapmap46959-BTA-16945 | 17 | 3 | 16.67% |
| 119 | chr6 | 3,350,928 | 3,684,492 | 333,565 | loss | Hapmap39586-BTA-78225 | BTB-01790614 | 10 | 1 | Unique |
| 120 | chr6 | 4,017,889 | 4,243,788 | 225,900 | loss | ARS-BFGL-NGS-80855 | ARS-BFGL-NGS-77840 | 8 | 2 | Multiple |
| 121 | chr6 | 8,850,886 | 12,358,731 | 3,507,846 | loss | BTB-02022899 | BTA-112689-no-rs | 82 | 2 | Multiple |
| 122 | chr6 | 13,269,348 | 13,780,271 | 510,924 | loss | ARS-BFGL-NGS-17079 | ARS-BFGL-NGS-54418 | 17 | 2 | Multiple |
| 123 | chr6 | 17,299,117 | 17,853,627 | 554,511 | loss | BTA-102826-no-rs | BTA-77526-no-rs | 19 | 3 | 16.67% |
| 124 | chr6 | 22,210,179 | 22,556,690 | 346,512 | loss | BTB-01430084 | BTB-01530236 | 10 | 1 | Unique |
| 125 | chr6 | 33,444,496 | 34,135,835 | 691,340 | loss | Hapmap27294-BTC-032117 | Hapmap46243-BTA-75753 | 20 | 1 | Unique |
| 126 | chr6 | 35,778,162 | 35,955,586 | 177,425 | loss | BTA-18965-no-rs | Hapmap59902-rs29013268 | 5 | 3 | 16.67% |
| 127 | chr6 | 38,076,964 | 38,500,210 | 423,247 | loss | BTA-100891-no-rs | Hapmap32207-BTC-034871 | 12 | 3 | 16.67% |
| 128 | chr6 | 39,052,609 | 41,951,591 | 2,898,983 | loss | Hapmap27344-BTC-063259 | BTA-75891-no-rs | 76 | 1 | Unique |
| 129 | chr6 | 43,220,732 | 43,333,442 | 112,711 | loss | ARS-BFGL-NGS-107577 | Hapmap47037-BTA-52688 | 4 | 3 | 16.67% |
| 130 | chr6 | 44,190,262 | 44,530,078 | 339,817 | loss | Hapmap28019-BTC-054809 | BFGL-NGS-117009 | 12 | 1 | Unique |
| 131 | chr6 | 47,058,970 | 47,292,122 | 233,153 | loss | BTA-76119-no-rs | ARS-BFGL-NGS-56572 | 5 | 2 | Multiple |
| 132 | chr6 | 49,972,459 | 51,811,956 | 1,839,498 | loss | Hapmap51152-BTA-115003 | BTB-00477280 | 41 | 2 | Multiple |
| 133 | chr6 | 52,278,780 | 54,354,528 | 2,075,749 | loss | Hapmap51414-BTA-18435 | Hapmap44575-BTA-86867 | 50 | 6 | 33.33% |
| 134 | chr6 | 55,196,036 | 56,074,791 | 878,756 | loss | BTB-00844067 | BTA-00391-no-rs | 27 | 2 | Multiple |
| 135 | chr6 | 57,062,313 | 57,806,017 | 743,705 | loss | BTB-00258382 | Hapmap41788-BTA-18387 | 19 | 1 | Unique |
| 136 | chr6 | 66,481,495 | 68,139,931 | 1,658,437 | loss | BTB-00260704 | BTB-01208895 | 43 | 4 | 22.22% |
| 137 | chr6 | 78,494,906 | 78,780,301 | 285,396 | loss | Hapmap44699-BTA-122320 | BTA-114800-no-rs | 7 | 2 | Multiple |
| 138 | chr6 | 79,885,829 | 79,944,604 | 58,776 | loss | Hapmap48883-BTA-76062 | BTA-76070-no-rs | 3 | 2 | Multiple |
| 139 | chr6 | 80,493,302 | 80,568,730 | 75,429 | loss | ARS-BFGL-NGS-102423 | Hapmap52479-rs29018853 | 3 | 2 | Multiple |
| 140 | chr6 | 82,465,964 | 82,924,932 | 458,969 | loss | BTB-01428337 | ARS-BFGL-NGS-108246 | 15 | 2 | Multiple |
| 141 | chr6 | 83,023,197 | 83,250,025 | 226,829 | loss | BTA-20903-no-rs | BTA-110240-no-rs | 6 | 2 | Multiple |
| 142 | chr6 | 83,957,417 | 84,087,732 | 130,316 | loss | Hapmap31932-BTC-042947 | Hapmap27308-BTC-043338 | 6 | 2 | Multiple |
| 143 | chr6 | 84,782,128 | 85,289,127 | 507,000 | loss | Hapmap47405-BTA-76965 | UA-IFASA-2111 | 12 | 3 | 16.67% |
| 144 | chr6 | 88,160,022 | 88,508,848 | 348,827 | loss | Hapmap54803-rs29015041 | BTA-77057-no-rs | 12 | 1 | Unique |
| 145 | chr6 | 112,484,937 | 112,541,019 | 56,083 | loss | BTA-20913-no-rs | BTB-01429126 | 3 | 2 | Multiple |
| 146 | chr6 | 116,851,144 | 116,998,217 | 147,074 | loss | Hapmap56871-rs29009955 | Hapmap55397-rs29017692 | 4 | 2 | Multiple |
| 147 | chr6 | 118,029,315 | 119,068,909 | 1,039,595 | loss | ARS-BFGL-NGS-91458 | BFGL-NGS-110156 | 27 | 7 | 38.89% |
| 148 | chr6 | 120,402,816 | 120,786,081 | 383,266 | loss | ARS-BFGL-NGS-4767 | ARS-BFGL-NGS-43621 | 11 | 2 | Multiple |
| 149 | chr7 | 1,343,221 | 1,493,916 | 150,696 | loss | ARS-BFGL-NGS-44014 | ARS-BFGL-NGS-62046 | 6 | 1 | Unique |
| 150 | chr7 | 1,782,963 | 1,853,587 | 70,625 | loss | BTB-00080498 | ARS-BFGL-NGS-83344 | 3 | 2 | Multiple |
| 151 | chr7 | 7,192,457 | 7,328,633 | 136,177 | loss | ARS-BFGL-NGS-35623 | ARS-BFGL-NGS-100448 | 6 | 2 | Multiple |
| 152 | chr7 | 23,606,613 | 23,725,387 | 118,775 | gain | Hapmap23838-BTA-163815 | Hapmap51633-BTA-78653 | 4 | 2 | Multiple |
| 153 | chr7 | 30,776,834 | 32,271,914 | 1,495,081 | loss | BTB-01862906 | Hapmap26699-BTA-147881 | 34 | 4 | 22.22% |
| 154 | chr7 | 40,692,412 | 41,312,905 | 620,494 | loss | BTB-00308027 | ARS-BFGL-NGS-11889 | 17 | 6 | 33.33% |
| 155 | chr7 | 41,428,236 | 41,545,602 | 117,367 | loss | BTB-01561790 | BTB-01907592 | 4 | 1 | Unique |
| 156 | chr7 | 42,506,811 | 42,787,608 | 280,798 | loss | ARS-BFGL-NGS-11022 | BFGL-NGS-109712 | 8 | 2 | Multiple |
| 157 | chr7 | 45,216,666 | 46,104,249 | 887,584 | loss | Hapmap50586-BTA-118480 | ARS-BFGL-NGS-18762 | 24 | 1 | Unique |
| 158 | chr7 | 46,516,412 | 46,585,684 | 69,273 | loss | BTB-01473837 | Hapmap52786-rs29024076 | 3 | 1 | Unique |
| 159 | chr7 | 51,295,658 | 51,918,280 | 622,623 | loss | ARS-BFGL-NGS-133 | ARS-BFGL-NGS-52769 | 13 | 1 | Unique |
| 160 | chr7 | 57,315,877 | 57,618,367 | 302,491 | loss | ARS-BFGL-NGS-37263 | BFGL-NGS-113745 | 7 | 2 | Multiple |
| 161 | chr7 | 62,733,503 | 62,800,839 | 67,337 | loss | ARS-BFGL-NGS-11636 | BFGL-NGS-113181 | 3 | 2 | Multiple |
| 162 | chr7 | 63,850,503 | 63,897,016 | 46,514 | loss | BTA-28702-no-rs | BTB-01106344 | 3 | 1 | Unique |
| 163 | chr7 | 63,971,023 | 64,677,828 | 706,806 | loss | BTB-01106504 | BTB-00317657 | 20 | 3 | 16.67% |
| 164 | chr7 | 71,361,555 | 71,483,796 | 122,242 | loss | Hapmap43944-BTA-79809 | BTB-00319289 | 6 | 1 | Unique |
| 165 | chr7 | 76,393,970 | 76,521,447 | 127,478 | loss | ARS-BFGL-NGS-35321 | BTA-98900-no-rs | 5 | 2 | Multiple |
| 166 | chr7 | 77,443,327 | 78,251,906 | 808,580 | loss | ARS-BFGL-NGS-41030 | BTB-00321513 | 23 | 2 | Multiple |
| 167 | chr7 | 90,549,127 | 90,661,453 | 112,327 | loss | Hapmap39636-BTA-21139 | ARS-BFGL-NGS-42061 | 3 | 2 | Multiple |
| 168 | chr7 | 93,268,631 | 93,534,061 | 265,431 | loss | ARS-BFGL-NGS-55808 | BTA-88250-no-rs | 7 | 5 | 27.78% |
| 169 | chr7 | 100,894,204 | 101,083,258 | 189,055 | loss | ARS-BFGL-NGS-14878 | Hapmap41090-BTA-80433 | 3 | 2 | Multiple |
| 170 | chr7 | 103,302,145 | 103,904,288 | 602,144 | loss | Hapmap22951-BTA-163311 | BTB-01805931 | 17 | 1 | Unique |
| 171 | chr7 | 104,641,305 | 105,769,863 | 1,128,559 | loss | BTB-00328499 | BTB-00956013 | 33 | 2 | Multiple |
| 172 | chr7 | 107,255,548 | 107,500,146 | 244,599 | loss | Hapmap51837-BTA-49470 | BTB-01878273 | 8 | 4 | 22.22% |
| 173 | chr7 | 107,808,267 | 111,761,300 | 3,953,034 | loss | BTA-118266-no-rs | BTB-01550935 | 106 | 3 | 16.67% |
| 174 | chr8 | 7,956,687 | 8,682,578 | 725,892 | loss | BFGL-NGS-117583 | ARS-BFGL-NGS-44248 | 16 | 1 | Unique |
| 175 | chr8 | 16,262,391 | 17,094,003 | 831,613 | loss | BTA-83017-no-rs | ARS-BFGL-NGS-36155 | 17 | 1 | Unique |
| 176 | chr8 | 18,100,799 | 18,222,132 | 121,334 | loss | BTA-80796-no-rs | ARS-BFGL-NGS-10089 | 4 | 1 | Unique |
| 177 | chr8 | 18,644,876 | 18,775,988 | 131,113 | loss | Hapmap42330-BTA-80832 | Hapmap49547-BTA-25463 | 3 | 2 | Multiple |
| 178 | chr8 | 20,893,495 | 21,016,274 | 122,780 | loss | ARS-BFGL-NGS-107527 | BFGL-NGS-110622 | 3 | 2 | Multiple |
| 179 | chr8 | 27,363,523 | 27,419,426 | 55,904 | loss | BTB-01051794 | BTA-27527-no-rs | 3 | 1 | Unique |
| 180 | chr8 | 27,495,240 | 27,851,465 | 356,226 | loss | ARS-BFGL-NGS-52642 | Hapmap51430-BTA-29826 | 10 | 2 | Multiple |
| 181 | chr8 | 28,721,492 | 29,308,131 | 586,640 | loss | BTA-80886-no-rs | BTB-01271294 | 13 | 1 | Unique |
| 182 | chr8 | 33,923,290 | 34,578,842 | 655,553 | loss | ARS-BFGL-NGS-96184 | BTA-76382-no-rs | 15 | 2 | Multiple |
| 183 | chr8 | 36,171,902 | 37,702,631 | 1,530,730 | loss | BTB-01184997 | Hapmap51410-BTA-122762 | 37 | 1 | Unique |
| 184 | chr8 | 52,950,178 | 53,499,374 | 549,197 | loss | BTB-00347575 | BTB-00349308 | 15 | 1 | Unique |
| 185 | chr8 | 53,942,222 | 53,991,683 | 49,462 | gain | BTB-00348844 | Hapmap57994-rs29022887 | 3 | 2 | Multiple |
| 186 | chr8 | 86,851,859 | 88,129,259 | 1,277,401 | loss | Hapmap26709-BTA-149656 | Hapmap41653-BTA-82121 | 30 | 2 | Multiple |
| 187 | chr8 | 91,326,289 | 91,407,193 | 80,905 | loss | Hapmap49329-BTA-82142 | BTB-00364735 | 3 | 2 | Multiple |
| 188 | chr8 | 95,574,886 | 95,773,222 | 198,337 | loss | Hapmap48568-BTA-103950 | BTB-01415906 | 5 | 1 | Unique |
| 189 | chr8 | 96,677,788 | 97,972,512 | 1,294,725 | loss | Hapmap41495-BTA-28967 | BTB-01734135 | 33 | 7 | 38.89% |
| 190 | chr8 | 103,656,644 | 103,797,179 | 140,536 | loss | ARS-BFGL-NGS-108343 | BTA-108157-no-rs | 4 | 2 | Multiple |
| 191 | chr8 | 104,929,794 | 105,035,117 | 105,324 | loss | BTB-00371041 | BTA-107369-no-rs | 4 | 1 | Unique |
| 192 | chr8 | 108,035,533 | 108,304,424 | 268,892 | loss | BFGL-NGS-118553 | ARS-BFGL-NGS-27157 | 9 | 1 | Unique |
| 193 | chr8 | 110,444,686 | 110,581,519 | 136,834 | gain | Hapmap57719-rs29018611 | BFGL-NGS-114840 | 4 | 2 | Multiple |
| 194 | chr8 | 114,134,677 | 114,215,199 | 80,523 | loss | ARS-BFGL-NGS-21307 | ARS-BFGL-NGS-2204 | 3 | 2 | Multiple |
| 195 | chr8 | 115,680,785 | 115,773,590 | 92,806 | loss | BFGL-NGS-113981 | BTA-82851-no-rs | 4 | 1 | Unique |
| 196 | chr9 | 1,053,431 | 2,289,237 | 1,235,807 | loss | Hapmap40061-BTA-28737 | Hapmap36664-SCAFFOLD50340_7682 | 29 | 2 | Multiple |
| 197 | chr9 | 2,733,562 | 2,918,894 | 185,333 | loss | ARS-BFGL-NGS-26056 | Hapmap33371-BTA-147485 | 4 | 1 | Unique |
| 198 | chr9 | 3,139,316 | 3,932,650 | 793,335 | loss | BTA-86675-no-rs | ARS-BFGL-NGS-13958 | 19 | 3 | 16.67% |
| 199 | chr9 | 4,366,424 | 4,554,052 | 187,629 | loss | BTA-83564-no-rs | Hapmap26163-BTA-151864 | 7 | 2 | Multiple |
| 200 | chr9 | 7,704,447 | 8,027,657 | 323,211 | loss | BTA-83825-no-rs | BFGL-NGS-113730 | 9 | 1 | Unique |
| 201 | chr9 | 13,958,461 | 15,414,089 | 1,455,629 | loss | ARS-BFGL-NGS-14740 | BTB-00380334 | 45 | 3 | 16.67% |
| 202 | chr9 | 25,735,289 | 25,863,098 | 127,810 | loss | Hapmap59909-rs29013870 | BTB-02020793 | 5 | 2 | Multiple |
| 203 | chr9 | 28,670,905 | 29,277,761 | 606,857 | loss | BTB-01932422 | Hapmap39984-BTA-21408 | 18 | 1 | Unique |
| 204 | chr9 | 32,217,742 | 32,778,459 | 560,718 | loss | BTB-01444539 | BTB-01440667 | 13 | 1 | Unique |
| 205 | chr9 | 34,328,320 | 34,602,197 | 273,878 | loss | Hapmap23593-BTA-146417 | BTA-112863-no-rs | 6 | 2 | Multiple |
| 206 | chr9 | 35,528,244 | 35,648,458 | 120,215 | loss | BTB-00388348 | BTB-00388186 | 5 | 2 | Multiple |
| 207 | chr9 | 36,578,500 | 36,967,501 | 389,002 | loss | BTB-01843749 | ARS-BFGL-NGS-23960 | 12 | 1 | Unique |
| 208 | chr9 | 36,989,725 | 37,072,013 | 82,289 | loss | ARS-BFGL-NGS-4290 | Hapmap33388-BTA-156375 | 3 | 3 | 16.67% |
| 209 | chr9 | 37,134,721 | 37,442,895 | 308,175 | loss | BTB-02058916 | BTB-00518033 | 7 | 2 | Multiple |
| 210 | chr9 | 38,711,556 | 39,369,495 | 657,940 | loss | ARS-BFGL-NGS-32807 | Hapmap60126-rs29022845 | 20 | 3 | 16.67% |
| 211 | chr9 | 48,117,824 | 48,252,872 | 135,049 | loss | Hapmap33674-BTA-156367 | BTA-83597-no-rs | 3 | 2 | Multiple |
| 212 | chr9 | 49,902,303 | 50,183,018 | 280,716 | gain | Hapmap49702-BTA-67493 | ARS-BFGL-NGS-88160 | 3 | 2 | Multiple |
| 213 | chr9 | 57,185,609 | 58,255,092 | 1,069,484 | loss | Hapmap26414-BTA-146523 | BTA-83741-no-rs | 29 | 1 | Unique |
| 214 | chr9 | 58,626,197 | 60,331,150 | 1,704,954 | loss | BTB-01151441 | BTB-00394165 | 45 | 1 | Unique |
| 215 | chr9 | 84,763,065 | 84,949,725 | 186,661 | loss | BTB-00400161 | ARS-BFGL-NGS-56140 | 7 | 1 | Unique |
| 216 | chr9 | 89,994,803 | 90,138,029 | 143,227 | loss | Hapmap56597-rs29018761 | BTB-01693152 | 6 | 3 | 16.67% |
| 217 | chr9 | 92,461,402 | 93,252,900 | 791,499 | loss | BTB-02074519 | BTB-01839335 | 20 | 2 | Multiple |
| 218 | chr9 | 100,608,493 | 100,948,909 | 340,417 | loss | Hapmap44659-BTA-84990 | UA-IFASA-2515 | 8 | 1 | Unique |
| 219 | chr9 | 103,899,331 | 104,581,465 | 682,135 | loss | ARS-BFGL-NGS-4320 | ARS-BFGL-NGS-106604 | 15 | 1 | Unique |
| 220 | chr10 | 13,034,549 | 13,247,673 | 213,125 | loss | ARS-BFGL-NGS-9514 | BTB-00410742 | 8 | 2 | Multiple |
| 221 | chr10 | 14,893,832 | 15,513,723 | 619,892 | loss | ARS-BFGL-NGS-92657 | ARS-BFGL-NGS-73149 | 19 | 1 | Unique |
| 222 | chr10 | 23,813,257 | 24,019,888 | 206,632 | gain | Hapmap32589-BTA-147560 | BTB-01799640 | 4 | 1 | Unique |
| 223 | chr10 | 39,996,113 | 40,540,809 | 544,697 | loss | Hapmap60128-rs29023056 | Hapmap27858-BTA-125431 | 18 | 2 | Multiple |
| 224 | chr10 | 42,755,545 | 42,892,585 | 137,041 | loss | ARS-BFGL-NGS-15026 | BFGL-NGS-112899 | 5 | 1 | Unique |
| 225 | chr10 | 45,524,238 | 46,244,873 | 720,636 | loss | BTB-00419986 | ARS-BFGL-NGS-61625 | 22 | 2 | Multiple |
| 226 | chr10 | 50,896,763 | 51,073,231 | 176,469 | loss | BTA-92902-no-rs | Hapmap51989-BTA-92903 | 5 | 2 | Multiple |
| 227 | chr10 | 51,875,050 | 52,298,327 | 423,278 | loss | BTB-00992193 | BTB-00424008 | 14 | 1 | Unique |
| 228 | chr10 | 53,512,385 | 53,747,496 | 235,112 | loss | BFGL-NGS-110070 | Hapmap57046-rs29017141 | 8 | 1 | Unique |
| 229 | chr10 | 63,979,689 | 64,128,838 | 149,150 | loss | ARS-BFGL-NGS-93201 | BTA-73310-no-rs | 5 | 2 | Multiple |
| 230 | chr10 | 72,483,724 | 73,237,869 | 754,146 | loss | Hapmap47719-BTA-74313 | BTB-01791763 | 23 | 4 | 22.22% |
| 231 | chr10 | 73,801,364 | 73,935,992 | 134,629 | loss | Hapmap51558-BTA-117380 | Hapmap27480-BTA-75244 | 4 | 1 | Unique |
| 232 | chr10 | 74,380,448 | 74,489,703 | 109,256 | loss | BTB-00435823 | Hapmap27627-BTA-155600 | 4 | 2 | Multiple |
| 233 | chr10 | 80,215,901 | 80,295,284 | 79,384 | loss | BFGL-NGS-111205 | BTA-105475-no-rs | 4 | 4 | 22.22% |
| 234 | chr10 | 89,655,670 | 89,835,156 | 179,487 | loss | ARS-BFGL-NGS-54445 | ARS-BFGL-NGS-3752 | 6 | 2 | Multiple |
| 235 | chr10 | 91,901,742 | 92,006,601 | 104,860 | loss | ARS-BFGL-NGS-9246 | BTA-80363-no-rs | 4 | 2 | Multiple |
| 236 | chr10 | 96,332,757 | 97,641,437 | 1,308,681 | loss | BTB-01663299 | BTB-01147244 | 31 | 3 | 16.67% |
| 237 | chr10 | 102,536,224 | 102,664,785 | 128,562 | loss | BTA-104505-no-rs | ARS-BFGL-NGS-26243 | 5 | 1 | Unique |
| 238 | chr10 | 104,033,163 | 104,123,602 | 90,440 | loss | ARS-BFGL-NGS-40686 | ARS-BFGL-NGS-63793 | 3 | 1 | Unique |
| 239 | chr11 | 1,146,342 | 1,231,011 | 84,670 | loss | BFGL-NGS-119513 | BTB-00449522 | 4 | 1 | Unique |
| 240 | chr11 | 3,612,596 | 4,110,774 | 498,179 | loss | Hapmap38641-BTA-93090 | ARS-BFGL-BAC-14277 | 16 | 3 | 16.67% |
| 241 | chr11 | 8,375,197 | 8,683,333 | 308,137 | loss | ARS-BFGL-BAC-11154 | BTB-00455867 | 8 | 1 | Unique |
| 242 | chr11 | 16,452,789 | 16,520,807 | 68,019 | loss | BFGL-NGS-119512 | ARS-BFGL-BAC-14856 | 3 | 1 | Unique |
| 243 | chr11 | 19,098,592 | 19,866,657 | 768,066 | loss | BTA-28869-no-rs | ARS-BFGL-NGS-66637 | 24 | 1 | Unique |
| 244 | chr11 | 23,286,508 | 25,011,591 | 1,725,084 | loss | BFGL-NGS-114345 | Hapmap51172-BTA-122698 | 48 | 3 | 16.67% |
| 245 | chr11 | 35,359,692 | 36,113,456 | 753,765 | loss | Hapmap44196-BTA-113212 | BTB-01427174 | 19 | 2 | Multiple |
| 246 | chr11 | 37,165,493 | 37,300,075 | 134,583 | loss | BTA-08480-no-rs | BTA-118786-no-rs | 5 | 2 | Multiple |
| 247 | chr11 | 52,250,223 | 52,355,098 | 104,876 | loss | BTB-01660245 | BTB-00474616 | 4 | 2 | Multiple |
| 248 | chr11 | 52,793,421 | 53,000,095 | 206,675 | loss | ARS-BFGL-NGS-66467 | ARS-BFGL-NGS-26558 | 3 | 2 | Multiple |
| 249 | chr11 | 59,907,601 | 60,264,787 | 357,187 | loss | BTB-01652806 | ARS-BFGL-BAC-11748 | 10 | 1 | Unique |
| 250 | chr11 | 63,556,147 | 64,224,343 | 668,197 | loss | ARS-BFGL-BAC-13039 | Hapmap42394-BTA-100120 | 16 | 1 | Unique |
| 251 | chr11 | 78,484,412 | 78,986,559 | 502,148 | loss | BFGL-NGS-112276 | Hapmap42047-BTA-106297 | 10 | 3 | 16.67% |
| 252 | chr11 | 106,573,154 | 110,171,705 | 3,598,552 | loss | Hapmap51810-BTA-119667 | ARS-BFGL-NGS-34624 | 93 | 6 | 33.33% |
| 253 | chr12 | 1,627,266 | 2,734,960 | 1,107,695 | loss | ARS-BFGL-NGS-75777 | BFGL-NGS-111874 | 34 | 4 | 22.22% |
| 254 | chr12 | 15,318,457 | 15,413,522 | 95,066 | loss | ARS-BFGL-NGS-105749 | Hapmap47914-BTA-31718 | 3 | 3 | 16.67% |
| 255 | chr12 | 20,675,711 | 20,748,454 | 72,744 | loss | ARS-BFGL-NGS-62496 | BFGL-NGS-118769 | 3 | 2 | Multiple |
| 256 | chr12 | 25,552,723 | 26,132,031 | 579,309 | loss | Hapmap42159-BTA-26305 | Hapmap24457-BTA-153392 | 11 | 2 | Multiple |
| 257 | chr12 | 30,509,649 | 30,562,181 | 52,533 | loss | ARS-BFGL-NGS-75842 | ARS-BFGL-NGS-4221 | 3 | 1 | Unique |
| 258 | chr12 | 36,124,143 | 36,240,165 | 116,023 | loss | ARS-BFGL-NGS-23138 | BTA-20724-no-rs | 5 | 1 | Unique |
| 259 | chr12 | 38,564,180 | 38,822,417 | 258,238 | loss | BTA-90709-no-rs | Hapmap49412-BTA-102739 | 6 | 2 | Multiple |
| 260 | chr12 | 44,796,637 | 45,202,694 | 406,058 | loss | BTA-21671-no-rs | BTB-01438513 | 13 | 1 | Unique |
| 261 | chr12 | 47,486,002 | 47,941,930 | 455,929 | loss | Hapmap30508-BTA-127628 | BTB-00776345 | 14 | 1 | Unique |
| 262 | chr12 | 48,065,066 | 48,277,917 | 212,852 | loss | ARS-BFGL-NGS-107671 | BTA-109387-no-rs | 7 | 2 | Multiple |
| 263 | chr12 | 48,696,636 | 48,890,474 | 193,839 | loss | ARS-BFGL-NGS-35729 | BTB-01194674 | 7 | 1 | Unique |
| 264 | chr12 | 52,639,544 | 52,756,058 | 116,515 | loss | Hapmap43521-BTA-23812 | BTB-00493019 | 6 | 1 | Unique |
| 265 | chr12 | 56,278,598 | 57,357,710 | 1,079,113 | loss | BTA-65462-no-rs | Hapmap43220-BTA-26980 | 30 | 2 | Multiple |
| 266 | chr12 | 57,806,289 | 58,499,788 | 693,500 | loss | BTB-01839492 | BTB-00266340 | 17 | 3 | 16.67% |
| 267 | chr12 | 59,319,943 | 59,659,165 | 339,223 | loss | ARS-BFGL-NGS-31482 | BTB-01202158 | 9 | 5 | 27.78% |
| 268 | chr12 | 61,765,131 | 63,206,726 | 1,441,596 | loss | BTB-01791112 | ARS-BFGL-NGS-102285 | 40 | 7 | 38.89% |
| 269 | chr12 | 64,985,768 | 65,604,221 | 618,454 | loss | BTA-99443-no-rs | BTB-00502546 | 17 | 3 | 16.67% |
| 270 | chr12 | 68,680,802 | 68,940,040 | 259,239 | loss | BTB-00503371 | ARS-BFGL-NGS-59515 | 10 | 2 | Multiple |
| 271 | chr12 | 69,785,534 | 70,169,216 | 383,683 | loss | BTB-01470808 | BTB-01938429 | 13 | 3 | 16.67% |
| 272 | chr12 | 70,623,009 | 70,696,014 | 73,006 | loss | ARS-BFGL-NGS-21566 | ARS-BFGL-NGS-15142 | 4 | 1 | Unique |
| 273 | chr12 | 78,604,343 | 79,233,396 | 629,054 | loss | BTB-00506579 | ARS-BFGL-NGS-22481 | 19 | 1 | Unique |
| 274 | chr12 | 84,204,016 | 85,202,836 | 998,821 | loss | BFGL-NGS-114545 | ARS-BFGL-NGS-49526 | 33 | 3 | 16.67% |
| 275 | chr13 | 8,868,545 | 9,115,049 | 246,505 | loss | BTA-33135-no-rs | BTA-33268-no-rs | 7 | 2 | Multiple |
| 276 | chr13 | 10,041,235 | 10,309,655 | 268,421 | loss | ARS-BFGL-NGS-80694 | BTA-24190-no-rs | 6 | 2 | Multiple |
| 277 | chr13 | 16,498,575 | 16,896,008 | 397,434 | gain | ARS-BFGL-BAC-11234 | BTB-00515690 | 10 | 2 | Multiple |
| 278 | chr13 | 19,270,400 | 20,921,843 | 1,651,444 | loss | ARS-BFGL-NGS-59742 | ARS-BFGL-NGS-103700 | 33 | 2 | Multiple |
| 279 | chr13 | 40,184,854 | 40,269,760 | 84,907 | loss | ARS-BFGL-NGS-1812 | ARS-BFGL-NGS-14594 | 4 | 1 | Unique |
| 280 | chr13 | 71,912,790 | 72,273,468 | 360,679 | loss | Hapmap53091-rs29015689 | Hapmap51825-BTA-33675 | 13 | 1 | Unique |
| 281 | chr13 | 72,994,622 | 73,064,284 | 69,663 | loss | ARS-BFGL-NGS-103887 | ARS-BFGL-NGS-107401 | 3 | 1 | Unique |
| 282 | chr13 | 73,104,253 | 73,469,467 | 365,215 | loss | ARS-BFGL-NGS-71538 | ARS-BFGL-NGS-73115 | 7 | 1 | Unique |
| 283 | chr13 | 78,433,765 | 78,561,543 | 127,779 | loss | ARS-BFGL-NGS-66863 | ARS-BFGL-NGS-2010 | 5 | 1 | Unique |
| 284 | chr13 | 79,108,300 | 79,337,899 | 229,600 | loss | BFGL-NGS-110851 | ARS-BFGL-NGS-36865 | 7 | 3 | 16.67% |
| 285 | chr13 | 84,198,973 | 84,250,459 | 51,487 | loss | ARS-BFGL-NGS-54483 | Hapmap40030-BTA-87432 | 3 | 2 | Multiple |
| 286 | chr14 | 931,163 | 1,215,180 | 284,018 | loss | ARS-BFGL-NGS-101653 | ARS-BFGL-NGS-107222 | 12 | 1 | Unique |
| 287 | chr14 | 4,665,139 | 4,983,527 | 318,389 | loss | ARS-BFGL-NGS-95830 | ARS-BFGL-BAC-10375 | 10 | 1 | Unique |
| 288 | chr14 | 6,457,258 | 6,694,008 | 236,751 | loss | Hapmap24518-BTC-062393 | ARS-BFGL-NGS-36549 | 9 | 4 | 22.22% |
| 289 | chr14 | 11,377,912 | 12,022,056 | 644,145 | loss | ARS-BFGL-NGS-104836 | Hapmap29971-BTA-128951 | 22 | 1 | Unique |
| 290 | chr14 | 12,958,060 | 13,355,843 | 397,784 | both | BFGL-NGS-119174 | Hapmap31182-BTA-159357 | 13 | 7 | 38.89% |
| 291 | chr14 | 22,768,980 | 22,967,674 | 198,695 | loss | BTB-01530836 | Hapmap40120-BTA-34288 | 5 | 2 | Multiple |
| 292 | chr14 | 24,730,409 | 26,117,015 | 1,386,607 | loss | ARS-BFGL-NGS-83583 | Hapmap27563-BTC-073928 | 45 | 2 | Multiple |
| 293 | chr14 | 31,687,339 | 32,004,992 | 317,654 | loss | BTB-01728481 | Hapmap33516-BTA-160247 | 6 | 2 | Multiple |
| 294 | chr14 | 32,251,101 | 32,854,553 | 603,453 | loss | Hapmap51590-BTA-40163 | UA-IFASA-7535 | 11 | 2 | Multiple |
| 295 | chr14 | 35,522,424 | 36,523,786 | 1,001,363 | loss | BTB-01362889 | UA-IFASA-8629 | 21 | 2 | Multiple |
| 296 | chr14 | 46,049,615 | 47,177,953 | 1,128,339 | loss | Hapmap26094-BTA-113379 | Hapmap43974-BTA-94241 | 27 | 3 | 16.67% |
| 297 | chr14 | 50,713,046 | 54,249,825 | 3,536,780 | loss | BTB-00333025 | BTB-01290167 | 97 | 3 | 16.67% |
| 298 | chr14 | 57,716,443 | 57,904,573 | 188,131 | loss | BTB-01075956 | UA-IFASA-9447 | 6 | 2 | Multiple |
| 299 | chr14 | 58,530,250 | 58,934,124 | 403,875 | loss | ARS-BFGL-BAC-25609 | Hapmap54769-rs29012948 | 11 | 2 | Multiple |
| 300 | chr14 | 65,632,690 | 66,302,949 | 670,260 | loss | ARS-BFGL-NGS-29155 | ARS-BFGL-BAC-23075 | 16 | 4 | 22.22% |
| 301 | chr14 | 72,674,131 | 72,848,265 | 174,135 | both | ARS-BFGL-NGS-13904 | ARS-BFGL-NGS-57639 | 7 | 3 | 16.67% |
| 302 | chr14 | 76,364,123 | 76,520,018 | 155,896 | loss | ARS-BFGL-NGS-106221 | ARS-USMARC-Parent-DQ846692-rs29010281 | 6 | 2 | Multiple |
| 303 | chr14 | 79,618,038 | 80,584,935 | 966,898 | both | Hapmap49062-BTA-122353 | ARS-BFGL-NGS-53263 | 22 | 4 | 22.22% |
| 304 | chr15 | 1,808,449 | 1,910,294 | 101,846 | loss | ARS-BFGL-NGS-101623 | ARS-BFGL-NGS-72590 | 3 | 4 | 22.22% |
| 305 | chr15 | 3,501,101 | 4,707,547 | 1,206,447 | loss | Hapmap25580-BTA-149003 | Hapmap57381-rs29020495 | 30 | 7 | 38.89% |
| 306 | chr15 | 6,300,046 | 6,585,863 | 285,818 | loss | Hapmap50589-BTA-119599 | BTB-02089263 | 6 | 3 | 16.67% |
| 307 | chr15 | 9,508,158 | 9,647,484 | 139,327 | loss | Hapmap51870-BTA-100797 | ARS-BFGL-NGS-14904 | 4 | 1 | Unique |
| 308 | chr15 | 9,872,142 | 11,576,701 | 1,704,560 | loss | Hapmap31453-BTA-148475 | BTB-00581622 | 41 | 3 | 16.67% |
| 309 | chr15 | 15,505,957 | 15,588,437 | 82,481 | loss | BTB-01296218 | ARS-BFGL-NGS-10895 | 3 | 2 | Multiple |
| 310 | chr15 | 30,726,249 | 31,040,130 | 313,882 | loss | ARS-BFGL-NGS-104624 | BTA-36518-no-rs | 7 | 3 | 16.67% |
| 311 | chr15 | 31,874,190 | 31,999,587 | 125,398 | loss | ARS-BFGL-NGS-38840 | Hapmap39409-BTA-46690 | 4 | 2 | Multiple |
| 312 | chr15 | 33,186,639 | 33,299,428 | 112,790 | loss | BFGL-NGS-118826 | ARS-BFGL-NGS-43617 | 4 | 2 | Multiple |
| 313 | chr15 | 37,208,949 | 37,313,146 | 104,198 | loss | ARS-BFGL-BAC-27763 | ARS-BFGL-NGS-11846 | 3 | 1 | Unique |
| 314 | chr15 | 46,815,885 | 46,953,962 | 138,078 | loss | BTB-02094536 | ARS-BFGL-NGS-40561 | 4 | 2 | Multiple |
| 315 | chr15 | 50,929,910 | 50,984,522 | 54,613 | gain | BTB-02024135 | BTB-02036460 | 3 | 1 | Unique |
| 316 | chr15 | 53,414,620 | 53,656,410 | 241,791 | loss | ARS-BFGL-NGS-40224 | Hapmap26652-BTA-130021 | 5 | 2 | Multiple |
| 317 | chr15 | 59,062,885 | 59,776,516 | 713,632 | loss | BTA-96695-no-rs | Hapmap42006-BTA-91398 | 17 | 1 | Unique |
| 318 | chr15 | 64,603,536 | 64,743,671 | 140,136 | loss | BFGL-NGS-111210 | ARS-BFGL-NGS-13628 | 6 | 2 | Multiple |
| 319 | chr15 | 67,594,684 | 71,377,310 | 3,782,627 | loss | BTA-37407-no-rs | Hapmap42924-BTA-37587 | 85 | 3 | 16.67% |
| 320 | chr15 | 72,978,572 | 73,145,760 | 167,189 | loss | ARS-BFGL-NGS-40765 | DIAS-232 | 5 | 2 | Multiple |
| 321 | chr15 | 75,929,805 | 76,061,121 | 131,317 | loss | ARS-BFGL-NGS-1045 | ARS-BFGL-NGS-3375 | 4 | 2 | Multiple |
| 322 | chr15 | 78,608,606 | 78,970,733 | 362,128 | both | BTA-26651-no-rs | ARS-BFGL-NGS-94527 | 10 | 2 | Multiple |
| 323 | chr15 | 79,340,261 | 79,617,457 | 277,197 | loss | BTB-02012820 | ARS-BFGL-NGS-69075 | 7 | 1 | Unique |
| 324 | chr15 | 79,968,923 | 80,175,945 | 207,023 | loss | BTB-01766588 | BFGL-NGS-113528 | 7 | 2 | Multiple |
| 325 | chr15 | 81,785,928 | 82,001,980 | 216,053 | loss | ARS-BFGL-NGS-14541 | ARS-BFGL-NGS-16343 | 7 | 2 | Multiple |
| 326 | chr15 | 83,328,724 | 83,434,168 | 105,445 | gain | BTB-01821444 | BTB-02014893 | 4 | 1 | Unique |
| 327 | chr15 | 84,225,112 | 84,271,828 | 46,717 | loss | Hapmap58511-rs29024296 | ARS-BFGL-NGS-42520 | 3 | 1 | Unique |
| 328 | chr16 | 2,609,244 | 2,826,101 | 216,858 | loss | BTB-00623849 | ARS-BFGL-NGS-58366 | 7 | 2 | Multiple |
| 329 | chr16 | 6,073,782 | 6,295,400 | 221,619 | loss | BTB-01397819 | BTA-88251-no-rs | 4 | 1 | Unique |
| 330 | chr16 | 7,937,101 | 9,120,260 | 1,183,160 | loss | BTB-01569285 | ARS-BFGL-NGS-26891 | 33 | 3 | 16.67% |
| 331 | chr16 | 9,283,438 | 9,472,339 | 188,902 | loss | Hapmap51359-BTA-96781 | BTB-02066851 | 8 | 3 | 16.67% |
| 332 | chr16 | 13,866,093 | 14,442,044 | 575,952 | loss | BTA-40259-no-rs | ARS-BFGL-BAC-20631 | 12 | 2 | Multiple |
| 333 | chr16 | 26,958,519 | 27,811,734 | 853,216 | loss | ARS-BFGL-NGS-59860 | ARS-BFGL-NGS-37475 | 23 | 1 | Unique |
| 334 | chr16 | 43,561,366 | 43,662,348 | 100,983 | loss | ARS-BFGL-NGS-33431 | ARS-BFGL-NGS-40078 | 4 | 1 | Unique |
| 335 | chr16 | 44,295,556 | 44,553,485 | 257,930 | loss | ARS-BFGL-NGS-18661 | ARS-BFGL-NGS-3487 | 7 | 1 | Unique |
| 336 | chr16 | 46,299,669 | 46,497,903 | 198,235 | loss | BTB-01842164 | ARS-BFGL-NGS-96028 | 8 | 1 | Unique |
| 337 | chr16 | 48,424,663 | 48,482,490 | 57,828 | loss | ARS-BFGL-NGS-98071 | ARS-BFGL-NGS-56551 | 3 | 2 | Multiple |
| 338 | chr16 | 48,525,713 | 48,699,302 | 173,590 | loss | ARS-BFGL-NGS-32799 | ARS-BFGL-NGS-1346 | 6 | 2 | Multiple |
| 339 | chr16 | 60,405,376 | 60,514,703 | 109,328 | loss | ARS-BFGL-NGS-91618 | ARS-BFGL-NGS-24788 | 4 | 2 | Multiple |
| 340 | chr16 | 74,489,443 | 74,900,510 | 411,068 | loss | ARS-BFGL-NGS-106184 | BTB-01299674 | 11 | 3 | 16.67% |
| 341 | chr16 | 76,649,872 | 76,771,583 | 121,712 | loss | ARS-BFGL-NGS-1297 | ARS-BFGL-NGS-4646 | 3 | 1 | Unique |
| 342 | chr17 | 771,834 | 875,223 | 103,390 | loss | Hapmap41213-BTA-26249 | ARS-BFGL-NGS-25835 | 3 | 3 | 16.67% |
| 343 | chr17 | 995,320 | 1,761,080 | 765,761 | loss | BTB-02009238 | Hapmap43464-BTA-111790 | 22 | 3 | 16.67% |
| 344 | chr17 | 3,278,026 | 3,504,881 | 226,856 | loss | BTB-00667009 | Hapmap48326-BTA-41656 | 8 | 1 | Unique |
| 345 | chr17 | 6,664,897 | 6,895,346 | 230,450 | loss | ARS-BFGL-NGS-79791 | Hapmap51728-BTA-41894 | 9 | 1 | Unique |
| 346 | chr17 | 8,319,739 | 9,271,971 | 952,233 | loss | BTB-01311007 | BFGL-NGS-114713 | 22 | 3 | 16.67% |
| 347 | chr17 | 12,222,068 | 12,720,106 | 498,039 | loss | BTB-01435781 | ARS-BFGL-NGS-67872 | 10 | 2 | Multiple |
| 348 | chr17 | 15,028,357 | 15,825,282 | 796,926 | loss | ARS-BFGL-NGS-93036 | BTB-00673952 | 19 | 4 | 22.22% |
| 349 | chr17 | 21,004,410 | 21,054,363 | 49,954 | loss | Hapmap33065-BTA-16026 | ARS-BFGL-NGS-23824 | 3 | 2 | Multiple |
| 350 | chr17 | 24,561,648 | 24,727,631 | 165,984 | loss | BTB-00675063 | BTA-88834-no-rs | 6 | 1 | Unique |
| 351 | chr17 | 26,136,776 | 26,370,919 | 234,144 | loss | ARS-BFGL-NGS-23409 | ARS-BFGL-NGS-88447 | 7 | 1 | Unique |
| 352 | chr17 | 28,276,191 | 28,816,786 | 540,596 | loss | Hapmap34820-BES2_Contig369_543 | ARS-BFGL-NGS-68171 | 10 | 1 | Unique |
| 353 | chr17 | 31,697,778 | 31,863,347 | 165,570 | loss | Hapmap55183-rs29024809 | ARS-USMARC-547 | 4 | 2 | Multiple |
| 354 | chr17 | 34,706,313 | 35,660,216 | 953,904 | loss | BTB-01421373 | Hapmap45113-BTA-54994 | 24 | 1 | Unique |
| 355 | chr17 | 37,312,780 | 39,505,881 | 2,193,102 | loss | BTA-40865-no-rs | ARS-BFGL-BAC-34666 | 46 | 5 | 27.78% |
| 356 | chr17 | 40,822,702 | 41,013,181 | 190,480 | loss | Hapmap27870-BTA-141223 | Hapmap51617-BTA-67409 | 5 | 1 | Unique |
| 357 | chr17 | 45,656,849 | 45,980,011 | 323,163 | loss | BTB-00677011 | Hapmap54457-rs29016184 | 9 | 1 | Unique |
| 358 | chr17 | 52,798,617 | 52,909,227 | 110,611 | loss | ARS-BFGL-NGS-9438 | ARS-BFGL-NGS-17507 | 3 | 1 | Unique |
| 359 | chr17 | 66,227,783 | 66,994,796 | 767,014 | loss | BFGL-NGS-118407 | BTB-00683557 | 23 | 3 | 16.67% |
| 360 | chr17 | 71,688,553 | 71,747,554 | 59,002 | loss | ARS-BFGL-NGS-70426 | ARS-BFGL-NGS-78487 | 3 | 1 | Unique |
| 361 | chr17 | 72,890,151 | 73,006,732 | 116,582 | loss | ARS-BFGL-NGS-98109 | BFGL-NGS-111165 | 5 | 2 | Multiple |
| 362 | chr17 | 74,292,266 | 76,487,768 | 2,195,503 | loss | ARS-BFGL-NGS-102993 | ARS-BFGL-NGS-35060 | 61 | 2 | Multiple |
| 363 | chr18 | 2,200,339 | 2,270,542 | 70,204 | loss | Hapmap47948-BTA-42577 | BTA-42548-no-rs | 3 | 2 | Multiple |
| 364 | chr18 | 7,200,062 | 7,459,381 | 259,320 | loss | ARS-BFGL-NGS-56265 | Hapmap58328-rs29013933 | 10 | 1 | Unique |
| 365 | chr18 | 10,348,281 | 10,990,136 | 641,856 | loss | ARS-BFGL-NGS-95752 | Hapmap47011-BTA-44425 | 18 | 2 | Multiple |
| 366 | chr18 | 16,199,104 | 16,753,603 | 554,500 | loss | ARS-BFGL-NGS-84061 | ARS-BFGL-NGS-2638 | 14 | 2 | Multiple |
| 367 | chr18 | 20,098,812 | 20,339,949 | 241,138 | loss | BFGL-NGS-119693 | ARS-BFGL-NGS-67638 | 5 | 1 | Unique |
| 368 | chr18 | 25,824,394 | 26,569,630 | 745,237 | loss | ARS-BFGL-NGS-44657 | Hapmap39026-BTA-42843 | 23 | 4 | 22.22% |
| 369 | chr18 | 31,343,934 | 31,529,408 | 185,475 | loss | BTB-00707941 | Hapmap40535-BTA-42974 | 4 | 2 | Multiple |
| 370 | chr18 | 33,612,930 | 33,690,057 | 77,128 | loss | ARS-BFGL-NGS-21451 | BFGL-NGS-112014 | 3 | 1 | Unique |
| 371 | chr18 | 47,597,796 | 47,737,412 | 139,617 | loss | ARS-BFGL-NGS-59215 | ARS-BFGL-NGS-18019 | 3 | 1 | Unique |
| 372 | chr18 | 47,861,333 | 48,070,223 | 208,891 | loss | Hapmap35421-SCAFFOLD98325_1119 | ARS-BFGL-NGS-64415 | 7 | 1 | Unique |
| 373 | chr18 | 52,712,568 | 52,852,779 | 140,212 | loss | BTA-43458-no-rs | ARS-BFGL-BAC-36969 | 5 | 2 | Multiple |
| 374 | chr18 | 57,095,120 | 57,306,049 | 210,930 | loss | BTA-97501-no-rs | ARS-BFGL-NGS-15079 | 8 | 2 | Multiple |
| 375 | chr18 | 58,497,360 | 59,139,981 | 642,622 | gain | BTB-01750971 | ARS-BFGL-NGS-82090 | 10 | 2 | Multiple |
| 376 | chr18 | 59,220,972 | 59,413,220 | 192,249 | loss | BTB-01425816 | BTB-01439818 | 4 | 1 | Unique |
| 377 | chr18 | 61,247,595 | 61,607,122 | 359,528 | gain | BTB-00149908 | BFGL-NGS-110828 | 8 | 2 | Multiple |
| 378 | chr18 | 61,914,052 | 61,970,850 | 56,799 | loss | BTB-00732576 | ARS-BFGL-NGS-28328 | 3 | 3 | 16.67% |
| 379 | chr18 | 62,669,295 | 63,217,107 | 547,813 | loss | ARS-BFGL-NGS-25963 | BFGL-NGS-119439 | 13 | 1 | Unique |
| 380 | chr19 | 3,208,837 | 3,365,189 | 156,353 | loss | ARS-BFGL-NGS-75390 | ARS-BFGL-NGS-16174 | 4 | 2 | Multiple |
| 381 | chr19 | 14,202,179 | 14,396,738 | 194,560 | loss | UA-IFASA-6550 | BFGL-NGS-110713 | 6 | 2 | Multiple |
| 382 | chr19 | 56,974,655 | 57,220,724 | 246,070 | loss | BFGL-NGS-110875 | BTA-08388-no-rs | 5 | 1 | Unique |
| 383 | chr19 | 61,024,043 | 61,153,124 | 129,082 | loss | UA-IFASA-6929 | UA-IFASA-8216 | 5 | 1 | Unique |
| 384 | chr19 | 61,190,707 | 61,501,060 | 310,354 | loss | ARS-BFGL-NGS-10846 | ARS-BFGL-NGS-105092 | 10 | 1 | Unique |
| 385 | chr19 | 63,437,193 | 63,788,680 | 351,488 | loss | BTB-00766881 | Hapmap29820-BTA-133508 | 10 | 1 | Unique |
| 386 | chr19 | 63,846,754 | 63,898,534 | 51,781 | loss | ARS-BFGL-NGS-99812 | ARS-BFGL-NGS-19972 | 3 | 2 | Multiple |
| 387 | chr20 | 14,778,766 | 15,183,218 | 404,453 | loss | Hapmap39930-BTA-51548 | BTA-51558-no-rs | 10 | 1 | Unique |
| 388 | chr20 | 19,771,943 | 22,089,393 | 2,317,451 | loss | ARS-BFGL-BAC-33645 | BTA-106532-no-rs | 45 | 3 | 16.67% |
| 389 | chr20 | 28,953,137 | 29,240,613 | 287,477 | loss | BTA-50152-no-rs | Hapmap50988-BTA-50138 | 6 | 1 | Unique |
| 390 | chr20 | 35,344,973 | 35,552,478 | 207,506 | loss | ARS-BFGL-BAC-27946 | ARS-BFGL-BAC-2469 | 8 | 1 | Unique |
| 391 | chr20 | 41,189,146 | 41,430,183 | 241,038 | loss | Hapmap42161-BTA-26363 | ARS-BFGL-NGS-13426 | 7 | 1 | Unique |
| 392 | chr20 | 44,712,037 | 44,824,549 | 112,513 | loss | BTA-50619-no-rs | BTB-00784252 | 4 | 1 | Unique |
| 393 | chr20 | 45,638,444 | 46,727,897 | 1,089,454 | loss | BTB-01263250 | Hapmap28978-BTA-149870 | 26 | 1 | Unique |
| 394 | chr20 | 47,747,151 | 52,789,237 | 5,042,087 | loss | ARS-BFGL-BAC-27247 | BTB-01529431 | 112 | 6 | 33.33% |
| 395 | chr20 | 53,290,555 | 55,143,519 | 1,852,965 | loss | BTA-111516-no-rs | Hapmap56784-rs29012419 | 41 | 2 | Multiple |
| 396 | chr20 | 56,066,862 | 56,965,211 | 898,350 | loss | BTB-01216319 | BTA-50817-no-rs | 21 | 1 | Unique |
| 397 | chr20 | 57,890,535 | 58,728,652 | 838,118 | loss | BTB-01128159 | ARS-BFGL-NGS-71359 | 16 | 1 | Unique |
| 398 | chr20 | 62,052,058 | 62,228,627 | 176,570 | loss | ARS-BFGL-NGS-4112 | BFGL-NGS-113022 | 6 | 1 | Unique |
| 399 | chr20 | 73,207,956 | 73,302,544 | 94,589 | loss | ARS-BFGL-NGS-100393 | BTB-00798633 | 4 | 1 | Unique |
| 400 | chr20 | 73,331,593 | 73,410,227 | 78,635 | loss | Hapmap51842-BTA-51266 | BTA-112312-no-rs | 4 | 3 | 16.67% |
| 401 | chr20 | 73,432,073 | 73,520,025 | 87,953 | loss | ARS-BFGL-NGS-38032 | ARS-BFGL-NGS-80240 | 4 | 1 | Unique |
| 402 | chr20 | 74,494,941 | 74,555,665 | 60,725 | loss | BTB-01518094 | ARS-BFGL-NGS-37036 | 3 | 1 | Unique |
| 403 | chr20 | 74,877,574 | 75,476,188 | 598,615 | loss | Hapmap44700-BTA-34998 | BFGL-NGS-112221 | 16 | 5 | 27.78% |
| 404 | chr21 | 7,321,340 | 7,784,478 | 463,139 | loss | Hapmap41815-BTA-28879 | ARS-BFGL-NGS-44686 | 14 | 2 | Multiple |
| 405 | chr21 | 10,922,512 | 12,009,434 | 1,086,923 | loss | BTA-12959-no-rs | BTA-53429-no-rs | 29 | 3 | 16.67% |
| 406 | chr21 | 49,338,616 | 54,172,391 | 4,833,776 | loss | Hapmap29405-BTA-159679 | ARS-BFGL-NGS-46793 | 103 | 3 | 16.67% |
| 407 | chr21 | 58,150,578 | 58,413,381 | 262,804 | gain | ARS-BFGL-BAC-34509 | ARS-BFGL-NGS-16304 | 7 | 2 | Multiple |
| 408 | chr21 | 62,475,739 | 62,596,140 | 120,402 | loss | ARS-BFGL-NGS-30590 | BTA-52914-no-rs | 4 | 1 | Unique |
| 409 | chr21 | 64,542,774 | 65,108,810 | 566,037 | loss | BTA-52957-no-rs | UA-IFASA-5796 | 15 | 2 | Multiple |
| 410 | chr21 | 67,355,169 | 67,982,081 | 626,913 | loss | ARS-BFGL-NGS-10808 | ARS-BFGL-NGS-71969 | 13 | 1 | Unique |
| 411 | chr21 | 68,918,117 | 68,992,562 | 74,446 | loss | ARS-BFGL-NGS-67767 | ARS-BFGL-NGS-104354 | 3 | 2 | Multiple |
| 412 | chr21 | 69,029,934 | 69,106,615 | 76,682 | gain | ARS-BFGL-NGS-32701 | ARS-BFGL-NGS-18250 | 4 | 2 | Multiple |
| 413 | chr22 | 7,566,101 | 7,746,077 | 179,977 | loss | ARS-BFGL-NGS-16037 | Hapmap23270-BTA-148536 | 5 | 2 | Multiple |
| 414 | chr22 | 12,126,595 | 12,191,537 | 64,943 | loss | BFGL-NGS-118910 | ARS-BFGL-NGS-43002 | 3 | 2 | Multiple |
| 415 | chr22 | 24,997,615 | 25,108,521 | 110,907 | loss | BTA-53883-no-rs | BTA-53892-no-rs | 4 | 2 | Multiple |
| 416 | chr22 | 26,866,219 | 27,219,118 | 352,900 | loss | ARS-BFGL-NGS-54727 | Hapmap51493-BTA-77185 | 7 | 4 | 22.22% |
| 417 | chr22 | 29,288,347 | 29,404,887 | 116,541 | loss | Hapmap40360-BTA-53989 | Hapmap27395-BTA-136468 | 4 | 1 | Unique |
| 418 | chr22 | 34,869,020 | 34,953,637 | 84,618 | loss | ARS-BFGL-NGS-91144 | BTA-60902-no-rs | 4 | 1 | Unique |
| 419 | chr22 | 38,262,056 | 38,471,067 | 209,012 | loss | BFGL-NGS-116802 | BTA-109833-no-rs | 7 | 2 | Multiple |
| 420 | chr22 | 42,442,618 | 42,584,907 | 142,290 | loss | Hapmap57565-rs29010880 | ARS-BFGL-NGS-20777 | 6 | 1 | Unique |
| 421 | chr22 | 49,891,542 | 50,266,202 | 374,661 | loss | ARS-BFGL-NGS-104532 | ARS-BFGL-NGS-44801 | 7 | 1 | Unique |
| 422 | chr22 | 60,179,969 | 60,619,340 | 439,372 | loss | ARS-BFGL-NGS-41278 | ARS-BFGL-NGS-13887 | 14 | 3 | 16.67% |
| 423 | chr23 | 1,829,364 | 1,952,279 | 122,916 | loss | ARS-BFGL-BAC-31235 | ARS-BFGL-BAC-30440 | 5 | 1 | Unique |
| 424 | chr23 | 22,575,045 | 22,680,716 | 105,672 | loss | Hapmap35058-BES7_Contig424_1473 | UA-IFASA-5859 | 3 | 3 | 16.67% |
| 425 | chr23 | 33,081,407 | 33,155,762 | 74,356 | loss | UA-IFASA-4233 | ARS-BFGL-BAC-28947 | 3 | 1 | Unique |
| 426 | chr23 | 35,475,106 | 35,573,710 | 98,605 | gain | Hapmap41775-BTA-121406 | ARS-BFGL-NGS-23431 | 3 | 2 | Multiple |
| 427 | chr23 | 50,241,214 | 50,282,271 | 41,058 | loss | ARS-BFGL-NGS-103489 | ARS-BFGL-NGS-28289 | 3 | 2 | Multiple |
| 428 | chr23 | 52,747,002 | 52,869,716 | 122,715 | gain | ARS-BFGL-NGS-92459 | BFGL-NGS-117465 | 5 | 1 | Unique |
| 429 | chr24 | 52,778 | 929,582 | 876,805 | loss | BTB-00875757 | BTB-00875313 | 24 | 1 | Unique |
| 430 | chr24 | 15,682,721 | 15,950,221 | 267,501 | loss | ARS-BFGL-NGS-101091 | BTA-24495-no-rs | 6 | 1 | Unique |
| 431 | chr24 | 18,360,447 | 19,327,908 | 967,462 | loss | ARS-BFGL-NGS-44468 | ARS-BFGL-NGS-28993 | 15 | 3 | 16.67% |
| 432 | chr24 | 21,616,747 | 21,678,887 | 62,141 | loss | ARS-BFGL-NGS-81820 | Hapmap59517-rs29027550 | 3 | 2 | Multiple |
| 433 | chr24 | 38,331,137 | 38,771,600 | 440,464 | loss | BTB-01189227 | ARS-BFGL-BAC-28286 | 14 | 2 | Multiple |
| 434 | chr24 | 59,981,903 | 60,413,160 | 431,258 | loss | ARS-BFGL-NGS-104375 | ARS-BFGL-NGS-45332 | 9 | 2 | Multiple |
| 435 | chr25 | 490,351 | 1,138,957 | 648,607 | loss | BTA-91900-no-rs | ARS-BFGL-NGS-108460 | 20 | 3 | 16.67% |
| 436 | chr25 | 35,133,908 | 35,352,563 | 218,656 | loss | ARS-BFGL-NGS-58465 | ARS-BFGL-NGS-104568 | 7 | 2 | Multiple |
| 437 | chr25 | 36,695,594 | 37,122,367 | 426,774 | loss | ARS-BFGL-NGS-13985 | ARS-BFGL-BAC-42642 | 14 | 1 | Unique |
| 438 | chr25 | 39,359,069 | 39,588,051 | 228,983 | loss | ARS-BFGL-NGS-9068 | ARS-BFGL-NGS-28673 | 7 | 2 | Multiple |
| 439 | chr25 | 40,716,235 | 43,938,950 | 3,222,716 | loss | ARS-BFGL-NGS-66266 | ARS-BFGL-NGS-35357 | 87 | 5 | 27.78% |
| 440 | chr26 | 3,360,805 | 4,588,174 | 1,227,370 | loss | ARS-BFGL-NGS-102718 | ARS-BFGL-NGS-76430 | 21 | 2 | Multiple |
| 441 | chr26 | 6,850,276 | 7,137,485 | 287,210 | loss | ARS-BFGL-NGS-98526 | ARS-BFGL-NGS-86536 | 8 | 2 | Multiple |
| 442 | chr26 | 8,309,050 | 8,434,010 | 124,961 | loss | Hapmap40338-BTA-113591 | Hapmap53362-rs29013727 | 5 | 2 | Multiple |
| 443 | chr26 | 11,699,089 | 11,901,519 | 202,431 | loss | BTB-00925273 | ARS-BFGL-NGS-53115 | 6 | 1 | Unique |
| 444 | chr26 | 14,031,690 | 14,241,512 | 209,823 | loss | ARS-BFGL-NGS-16205 | BTB-01134531 | 7 | 2 | Multiple |
| 445 | chr26 | 20,388,135 | 20,476,860 | 88,726 | loss | ARS-BFGL-NGS-90129 | BFGL-NGS-111739 | 3 | 2 | Multiple |
| 446 | chr26 | 25,542,842 | 25,829,547 | 286,706 | loss | BTA-02609-no-rs | Hapmap25945-BTA-61048 | 8 | 1 | Unique |
| 447 | chr26 | 43,849,121 | 44,399,075 | 549,955 | loss | Hapmap52062-rs29027270 | BFGL-NGS-117809 | 15 | 2 | Multiple |
| 448 | chr26 | 49,627,247 | 50,262,534 | 635,288 | loss | Hapmap28514-BTA-163525 | ARS-BFGL-NGS-19710 | 20 | 2 | Multiple |
| 449 | chr26 | 50,951,076 | 51,276,688 | 325,613 | loss | ARS-BFGL-NGS-28014 | ARS-BFGL-NGS-28608 | 10 | 1 | Unique |
| 450 | chr27 | 1,864,510 | 2,058,224 | 193,715 | loss | Hapmap48565-BTA-103629 | ARS-BFGL-NGS-41182 | 6 | 1 | Unique |
| 451 | chr27 | 4,833,630 | 4,973,003 | 139,374 | loss | Hapmap23103-BTA-62512 | Hapmap33202-BTA-139213 | 5 | 2 | Multiple |
| 452 | chr27 | 9,681,101 | 11,258,272 | 1,577,172 | loss | ARS-BFGL-NGS-78836 | BTB-00950742 | 40 | 4 | 22.22% |
| 453 | chr27 | 12,637,210 | 13,641,251 | 1,004,042 | loss | Hapmap58782-rs29016179 | Hapmap60236-rs29015203 | 19 | 1 | Unique |
| 454 | chr27 | 17,397,248 | 18,833,832 | 1,436,585 | loss | Hapmap61080-rs29013547 | BTB-00957943 | 41 | 1 | Unique |
| 455 | chr27 | 19,569,812 | 20,228,818 | 659,007 | loss | BTB-00000910 | BTB-00959009 | 16 | 2 | Multiple |
| 456 | chr27 | 21,649,690 | 21,942,707 | 293,018 | loss | ARS-BFGL-NGS-29169 | BTB-00959704 | 7 | 1 | Unique |
| 457 | chr27 | 22,319,466 | 22,773,879 | 454,414 | loss | Hapmap45196-BTA-62416 | DIAS-298 | 11 | 3 | 16.67% |
| 458 | chr27 | 25,421,631 | 25,736,878 | 315,248 | loss | Hapmap52620-rs29010181 | Hapmap44556-BTA-66734 | 11 | 4 | 22.22% |
| 459 | chr27 | 32,048,619 | 32,184,909 | 136,291 | loss | BFGL-NGS-116607 | ARS-BFGL-NGS-21155 | 3 | 2 | Multiple |
| 460 | chr27 | 39,071,705 | 39,440,046 | 368,342 | loss | ARS-BFGL-NGS-88722 | Hapmap47065-BTA-62853 | 11 | 1 | Unique |
| 461 | chr27 | 40,362,805 | 41,342,421 | 979,617 | loss | BFGL-NGS-117168 | Hapmap58237-rs29017248 | 24 | 3 | 16.67% |
| 462 | chr27 | 41,556,848 | 41,715,718 | 158,871 | gain | Hapmap59286-rs29021722 | ARS-BFGL-NGS-96775 | 5 | 2 | Multiple |
| 463 | chr27 | 46,820,986 | 46,901,680 | 80,695 | loss | BTB-01795364 | Hapmap42612-BTA-63039 | 3 | 1 | Unique |
| 464 | chr28 | 9,580,455 | 9,935,659 | 355,205 | loss | Hapmap39009-BTA-121529 | BTA-64714-no-rs | 5 | 2 | Multiple |
| 465 | chr28 | 10,430,536 | 10,802,567 | 372,032 | loss | Hapmap55640-rs29014036 | Hapmap53063-rs29021085 | 9 | 1 | Unique |
| 466 | chr28 | 11,127,515 | 11,460,946 | 333,432 | loss | Hapmap55826-rs29023262 | Hapmap45049-BTA-64763 | 7 | 2 | Multiple |
| 467 | chr28 | 14,672,525 | 15,044,993 | 372,469 | loss | Hapmap32897-BTA-63586 | BFGL-NGS-119777 | 10 | 1 | Unique |
| 468 | chr28 | 19,525,842 | 21,046,555 | 1,520,714 | loss | BTB-01128535 | ARS-BFGL-NGS-71405 | 29 | 1 | Unique |
| 469 | chr28 | 21,258,397 | 21,355,007 | 96,611 | loss | BTB-01650236 | ARS-BFGL-NGS-49095 | 3 | 1 | Unique |
| 470 | chr28 | 24,023,598 | 24,436,312 | 412,715 | loss | BTA-63758-no-rs | Hapmap44554-BTA-63753 | 11 | 1 | Unique |
| 471 | chr28 | 26,163,899 | 26,459,549 | 295,651 | loss | ARS-BFGL-NGS-76173 | ARS-BFGL-NGS-8380 | 9 | 2 | Multiple |
| 472 | chr28 | 36,623,843 | 37,144,503 | 520,661 | loss | Hapmap49693-BTA-64161 | BFGL-NGS-110226 | 17 | 2 | Multiple |
| 473 | chr28 | 41,724,262 | 41,867,131 | 142,870 | loss | ARS-BFGL-NGS-40955 | BFGL-NGS-119555 | 5 | 2 | Multiple |
| 474 | chr29 | 9,543,767 | 10,243,575 | 699,809 | loss | Hapmap39855-BTA-66440 | Hapmap49260-BTA-66294 | 19 | 1 | Unique |
| 475 | chr29 | 16,949,905 | 17,476,493 | 526,589 | loss | BTA-64901-no-rs | ARS-BFGL-NGS-2529 | 12 | 1 | Unique |
| 476 | chr29 | 20,754,360 | 20,935,927 | 181,568 | loss | ARS-BFGL-NGS-1678 | Hapmap54563-rs29009937 | 4 | 2 | Multiple |
| 477 | chr29 | 23,249,812 | 23,844,887 | 595,076 | loss | ARS-BFGL-NGS-70494 | BTB-00511910 | 15 | 2 | Multiple |
| 478 | chr29 | 29,211,966 | 29,270,057 | 58,092 | gain | BFGL-NGS-114791 | ARS-BFGL-NGS-17874 | 3 | 2 | Multiple |
| 479 | chr29 | 31,219,550 | 31,837,246 | 617,697 | loss | BFGL-NGS-118114 | ARS-BFGL-NGS-68193 | 17 | 3 | 16.67% |
| 480 | chr29 | 32,470,203 | 33,014,347 | 544,145 | loss | BTB-01022586 | BTB-01020342 | 13 | 2 | Multiple |
| 481 | chr29 | 44,193,317 | 44,277,693 | 84,377 | loss | ARS-BFGL-NGS-101195 | ARS-BFGL-NGS-7054 | 3 | 2 | Multiple |
| 482 | chr29 | 50,118,683 | 50,346,962 | 228,280 | loss | Hapmap40456-BTA-66218 | ARS-BFGL-NGS-26500 | 8 | 1 | Unique |
| 483 | chr29 | 50,879,371 | 51,788,033 | 908,663 | loss | ARS-BFGL-NGS-3129 | ARS-BFGL-NGS-14481 | 25 | 5 | 27.78% |

**Table S8. The effects of CNVR length and frequency on calling consistences between CNV callings based on SNP and aCGH.**

| ***Filtering Criteria*** | ***Total Overlaps*** | ***Array CGH CNVR*** | | | ***SNP CNVR*** | | |
| --- | --- | --- | --- | --- | --- | --- | --- |
|  |  | ***Unique overlap*** | ***Total*** | ***Percent*** | ***Unique overlap*** | ***Total*** | ***Percent*** |
| SNP Count |  |  |  |  |  |  |  |
| 3 | 59 | 59 | 163 | 36.20% | 57 | 682 | 8.36% |
| 5 | 35 | 35 | 163 | 21.47% | 33 | 279 | 11.83% |
| 8 | 21 | 21 | 163 | 12.88% | 20 | 104 | 19.23% |
| 10 | 16 | 16 | 163 | 9.82% | 15 | 69 | 21.74% |
| Animal Count |  |  |  |  |  |  |  |
| 2 | 52 | 52 | 163 | 31.90% | 50 | 404 | 12.38% |
| 3 | 45 | 45 | 163 | 27.61% | 43 | 234 | 18.38% |
| 5 | 37 | 37 | 163 | 22.70% | 35 | 136 | 25.74% |
| 10 | 29 | 29 | 163 | 17.79% | 28 | 58 | 48.28% |
| 20 | 21 | 21 | 163 | 12.88% | 20 | 25 | 80.00% |
| 25 | 18 | 18 | 163 | 11.04% | 17 | 19 | 89.47% |
| 50 | 10 | 10 | 163 | 6.13% | 9 | 9 | 100.00% |

| ***No*** | ***CNVR*** | ***Primer name*** | ***Chr*** | ***Forward start*** | ***Forward end*** | ***Reverse end*** | ***Reverse start*** | ***SNP name*** | ***SNP position*** | ***PennCNV*** | ***qPCR*** | ***Confirmed*** |
| --- | --- | --- | --- | --- | --- | --- | --- | --- | --- | --- | --- | --- |
| 1 | 58 | c1_LMS023_1 | 1 | 156,320,553 | 156,320,574 | 156,320,691 | 156,320,712 | Hapmap28036-BTA-125012 | 156,320,504 | 1 | 1.41 | yes |
| 2 | 184 | c5_HFD002_2 | 5 | 96,481,108 | 96,481,129 | 96,481,274 | 96,481,295 | Hapmap42271-BTA-61859 | 96,448,995 | 3 | 1.45 | no |
| 3 | 257 | c7_HOL013_2 | 7 | 96,985,984 | 96,986,005 | 96,986,116 | 96,986,137 | ARS-BFGL-NGS-68719 | 96,985,983 | 1 | 1.42 | yes |
|  |  | c7_HOL013_3 | 7 | 97,011,271 | 97,011,292 | 97,011,444 | 97,011,465 | Hapmap24200-BTA-147598 | 97,011,276 | 1 | 1.31 | yes |
| 4 | 278 | c8_HOL010_1 | 8 | 68,525,485 | 68,525,506 | 68,525,685 | 68,525,706 | BTB-01952185 | 68,525,502 | 3 | 1.65 | no |
|  |  | c8_HOL010_2 | 8 | 68,552,736 | 68,552,757 | 68,552,914 | 68,552,935 | BTB-01657524 | 68,552,526 | 3 | 0.91 | no |
| 5 | 345 | c11_HFD016_1 | 11 | 41,312,433 | 41,312,454 | 41,312,641 | 41,312,662 | Hapmap32339-BTA-149195 | 41,312,831 | 3 | 1.95 | no |
|  |  | c11_HFD016_2 | 11 | 41,340,503 | 41,340,524 | 41,340,688 | 41,340,709 | ARS-BFGL-BAC-13663 | 41,340,542 | 3 | 2.08 | no |
| 6 | 368 | 1_c12_HOL009_1 | 12 | 39,952,779 | 39,952,800 | 39,952,953 | 39,952,974 | ARS-BFGL-BAC-16276 | 39,952,770 | 1 | 2.12 | no |
|  |  | 1_c12_HOL009_2 | 12 | 40,029,594 | 40,029,616 | 40,029,782 | 40,029,803 | BTA-102546-no-rs | 40,029,647 | 1 | 1.47 | yes |
| 7 | 371 | 2_c12_HOL009_2 | 12 | 45,590,534 | 45,590,556 | 45,590,698 | 45,590,719 | BTA-22706-no-rs | 45,590,684 | 1 | 1.31 | yes |
| 8 | 434 | c15_HOL037_3 | 15 | 8,243,688 | 8,243,709 | 8,243,816 | 8,243,837 | BTB-00580758 | 8,243,906 | 1 | 1.13 | yes |
| 9 | 464 | c16_HOL037_2 | 16 | 8,804,619 | 8,804,640 | 8,804,749 | 8,804,770 | BTB-01691098 | 8,804,867 | 3 | 1.72 | no |
|  |  | c16_HOL037_3 | 16 | 8,835,954 | 8,835,975 | 8,836,115 | 8,836,136 | BTB-01691083 | 8,835,941 | 3 | 3.22 | yes |
| 10 | 508 | c18_HOL010_1 | 18 | 54,489,321 | 54,489,342 | 54,489,504 | 54,489,524 | ARS-BFGL-NGS-101147 | 54,489,550 | 3 | 1.81 | no |
|  |  | c18_HOL010_2 | 18 | 54,516,034 | 54,516,055 | 54,516,223 | 54,516,244 | ARS-BFGL-NGS-13667 | 54,516,088 | 3 | 0.78 | no |
| 11 | 609 | c25_HFD002_1 | 25 | 8,229,972 | 8,229,993 | 8,230,176 | 8,230,197 | ARS-BFGL-NGS-3547 | 8,230,074 | 3 | 1.67 | no |
| 12 | 626 | c26_HFD002_1 | 26 | 29,087,166 | 29,087,187 | 29,087,376 | 29,087,397 | BTB-01547063 | 29,087,354 | 3 | 1.79 | no |
| 13 | 624 | c26_HFD016_1 | 26 | 24,656,632 | 24,656,653 | 24,656,847 | 24,656,868 | ARS-BFGL-NGS-102845 | 24,656,664 | 1 | 1.90 | no |
|  |  | c26_HFD016_2 | 26 | 24,700,558 | 24,700,579 | 24,700,735 | 24,700,756 | ARS-BFGL-NGS-33708 | 24,700,724 | 1 | 0.90 | yes |
| 14 | 645 | c27_HFD002_1 | 27 | 42,284,971 | 42,284,992 | 42,285,117 | 42,285,138 | BTB-01682077 | 42,285,165 | 3 | 2.96 | yes |
|  |  | c27_HFD002_2 | 27 | 42,310,124 | 42,310,145 | 42,310,289 | 42,310,310 | BTB-00971327 | 42,310,105 | 3 | 3.12 | yes |
| 15 | 668 | c29_HOL013_1 | 29 | 27,814,525 | 27,814,546 | 27,814,698 | 27,814,719 | ARS-BFGL-NGS-32195 | 27,814,492 | 3 | 2.88 | yes |
|  |  | c29_HOL013_2 | 29 | 27,871,041 | 27,871,062 | 27,871,175 | 27,871,196 | BTB-01015218 | 27,870,992 | 3 | 1.49 | no |

**Table S9. qPCR Summary.**

**Table S10. Over/Underrepresentation of PANTHER molecular function, biological process and pathway terms.**

|  | ***Genome (23415)*** | ***CNVR (1537)*** | ***Expected*** | ***Over/Under***  ***representation*** | | ***P-value*** |
| --- | --- | --- | --- | --- | --- | --- |
| **Molecular Function** |  |  |  |  |  | |
| Other receptor | 366 | 70 | 24.02 | + | 1.64E-12 | |
| Receptor | 2862 | 284 | 187.87 | + | 3.51E-11 | |
| Molecular function unclassified | 5912 | 493 | 388.07 | + | 3.31E-08 | |
| Defense/immunity protein | 706 | 86 | 46.34 | + | 2.09E-06 | |
| Nucleic acid binding | 2942 | 128 | 193.12 | - | 2.29E-06 | |
| Other defense and immunity protein | 137 | 27 | 8.99 | + | 1.38E-04 | |
| Hydrolase | 840 | 28 | 55.14 | - | 9.45E-04 | |
| G-protein coupled receptor | 1538 | 145 | 100.96 | + | 1.78E-03 | |
| Large G-protein | 74 | 15 | 4.86 | + | 3.24E-02 | |
| Miscellaneous function | 931 | 39 | 61.11 | - | 4.06E-02 | |
| **Biological Process** |  |  |  |  |  | |
| Macrophage-mediated immunity | 207 | 60 | 13.59 | + | 1.36E-18 | |
| Immunity and defense | 1912 | 210 | 125.51 | + | 8.77E-12 | |
| Chemosensory perception | 1073 | 123 | 70.43 | + | 5.11E-07 | |
| Protein metabolism and modification | 3406 | 151 | 223.58 | - | 6.64E-07 | |
| Olfaction | 1063 | 122 | 69.78 | + | 7.58E-07 | |
| Biological process unclassified | 6347 | 504 | 416.63 | + | 1.55E-05 | |
| Sensory perception | 1457 | 142 | 95.64 | + | 8.16E-05 | |
| Nucleoside, nucleotide and nucleic acid metabolism | 3590 | 180 | 235.65 | - | 8.43E-04 | |
| Interferon-mediated immunity | 80 | 18 | 5.25 | + | 1.43E-03 | |
| Protein modification | 1318 | 54 | 86.52 | - | 1.17E-02 | |
| T-cell mediated immunity | 349 | 43 | 22.91 | + | 1.45E-02 | |
| Developmental processes | 2390 | 122 | 156.88 | - | 4.34E-02 | |
| **Pathway** |  |  |  |  |  | |
| Unclassified | 19893 | 1369 | 1305.81 | + | 2.69E-04 | |

**Table S11. Cattle CNV regions overlap with genomic regions under positive selection, human orthologous OMIM genes and cattle QTLs. See Additional file 3: Table S11.xls.**

**Table S12. CNVR frequency differences among breeds.**

| **Breed** | **HOL** | **ANG** | | **LMS** | | **HFD** | | **JER** | | **CHL** | | **BSW** | | **PMT** | | | **RMG** | | **GNS** | | **NRC** | **RGU** | **GBV** | | **SIM** | | **BRM** | | **GIR** | | **NEL** | | | **BMA** | | **SGT** | | **NDA** | | **SHK** |
| --- | --- | --- | --- | --- | --- | --- | --- | --- | --- | --- | --- | --- | --- | --- | --- | --- | --- | --- | --- | --- | --- | --- | --- | --- | --- | --- | --- | --- | --- | --- | --- | --- | --- | --- | --- | --- | --- | --- | --- | --- |
| **(No)** | **57** | **59** | | **40** | | **24** | | **27** | | **25** | | **24** | | **19** | | | **23** | | **20** | | **21** | **12** | **3** | | **3** | | **17** | | **23** | | **19** | | | **23** | | **23** | | **18** | | **14** |
| cnvr5 | 0.00 | | 0.00 | | 0.00 | | 0.04 | | 0.00 | | 0.20 | | 0.00 | | 0.05 | 0.00 | | 0.00 | | 0.00 | | 0.08 | | 0.00 | | 0.00 | | 0.00 | | 0.00 | | 0.00 | 0.00 | | 0.00 | | 0.00 | | 0.00 | |
| cnvr9 | 0.00 | | 0.00 | | 0.00 | | 0.00 | | 0.00 | | 0.00 | | 0.00 | | 0.00 | 0.00 | | 0.00 | | 0.00 | | 0.00 | | 0.00 | | 0.00 | | 0.18 | | 0.00 | | 0.00 | 0.00 | | 0.00 | | 0.00 | | 0.00 | |
| cnvr19 | 0.00 | | 0.00 | | 0.00 | | 0.00 | | 0.00 | | 0.00 | | 0.00 | | 0.00 | 0.00 | | 0.15 | | 0.00 | | 0.00 | | 0.00 | | 0.00 | | 0.00 | | 0.00 | | 0.00 | 0.00 | | 0.00 | | 0.00 | | 0.00 | |
| cnvr24 | 0.02 | | 0.00 | | 0.00 | | 0.00 | | 0.00 | | 0.16 | | 0.00 | | 0.05 | 0.00 | | 0.00 | | 0.00 | | 0.00 | | 0.33 | | 0.00 | | 0.00 | | 0.00 | | 0.00 | 0.00 | | 0.00 | | 0.00 | | 0.00 | |
| cnvr36 | 0.00 | | 0.00 | | 0.00 | | 0.00 | | 0.00 | | 0.00 | | 0.00 | | 0.00 | 0.00 | | 0.00 | | 0.14 | | 0.00 | | 0.00 | | 0.00 | | 0.00 | | 0.00 | | 0.00 | 0.00 | | 0.00 | | 0.06 | | 0.14 | |
| cnvr41 | 0.00 | | 0.12 | | 0.03 | | 0.00 | | 0.07 | | 0.04 | | 0.00 | | 0.00 | 0.00 | | 0.00 | | 0.00 | | 0.00 | | 0.00 | | 0.00 | | 0.00 | | 0.13 | | 0.00 | 0.00 | | 0.04 | | 0.06 | | 0.07 | |
| cnvr50 | 0.00 | | 0.00 | | 0.00 | | 0.00 | | 0.00 | | 0.04 | | 0.00 | | 0.00 | 0.00 | | 0.00 | | 0.05 | | 0.00 | | 0.00 | | 0.00 | | 0.00 | | 0.13 | | 0.00 | 0.00 | | 0.09 | | 0.00 | | 0.00 | |
| cnvr51 | 0.00 | | 0.00 | | 0.05 | | 0.00 | | 0.00 | | 0.00 | | 0.00 | | 0.00 | 0.13 | | 0.00 | | 0.05 | | 0.00 | | 0.00 | | 0.00 | | 0.00 | | 0.00 | | 0.00 | 0.00 | | 0.00 | | 0.00 | | 0.29 | |
| cnvr56 | 0.00 | | 0.00 | | 0.00 | | 0.00 | | 0.00 | | 0.12 | | 0.00 | | 0.00 | 0.00 | | 0.00 | | 0.00 | | 0.00 | | 0.00 | | 0.00 | | 0.00 | | 0.00 | | 0.00 | 0.00 | | 0.00 | | 0.00 | | 0.00 | |
| cnvr57 | 0.00 | | 0.00 | | 0.00 | | 0.00 | | 0.00 | | 0.00 | | 0.00 | | 0.00 | 0.13 | | 0.00 | | 0.00 | | 0.00 | | 0.00 | | 0.00 | | 0.00 | | 0.00 | | 0.00 | 0.00 | | 0.00 | | 0.00 | | 0.00 | |
| cnvr72 | 0.00 | | 0.00 | | 0.00 | | 0.00 | | 0.00 | | 0.00 | | 0.25 | | 0.00 | 0.00 | | 0.00 | | 0.00 | | 0.00 | | 0.00 | | 0.00 | | 0.00 | | 0.00 | | 0.00 | 0.00 | | 0.00 | | 0.00 | | 0.00 | |
| cnvr79 | 0.00 | | 0.00 | | 0.00 | | 0.00 | | 0.00 | | 0.00 | | 0.00 | | 0.00 | 0.09 | | 0.00 | | 0.00 | | 0.00 | | 0.00 | | 0.00 | | 0.18 | | 0.30 | | 0.21 | 0.04 | | 0.00 | | 0.00 | | 0.14 | |
| cnvr92 | 0.00 | | 0.00 | | 0.00 | | 0.00 | | 0.00 | | 0.00 | | 0.00 | | 0.00 | 0.00 | | 0.00 | | 0.00 | | 0.00 | | 0.00 | | 0.00 | | 0.06 | | 0.04 | | 0.05 | 0.04 | | 0.13 | | 0.00 | | 0.07 | |
| cnvr95 | 0.00 | | 0.00 | | 0.00 | | 0.00 | | 0.00 | | 0.00 | | 0.00 | | 0.00 | 0.00 | | 0.00 | | 0.00 | | 0.00 | | 0.00 | | 0.00 | | 0.00 | | 0.04 | | 0.16 | 0.09 | | 0.04 | | 0.00 | | 0.00 | |
| cnvr98 | 0.00 | | 0.00 | | 0.00 | | 0.00 | | 0.00 | | 0.00 | | 0.00 | | 0.00 | 0.00 | | 0.00 | | 0.00 | | 0.00 | | 0.00 | | 0.00 | | 0.35 | | 0.17 | | 0.32 | 0.13 | | 0.00 | | 0.00 | | 0.00 | |
| cnvr99 | 0.00 | | 0.00 | | 0.08 | | 0.13 | | 0.00 | | 0.00 | | 0.04 | | 0.00 | 0.00 | | 0.15 | | 0.00 | | 0.00 | | 0.00 | | 0.00 | | 0.00 | | 0.00 | | 0.00 | 0.00 | | 0.00 | | 0.00 | | 0.07 | |
| cnvr107 | 0.02 | | 0.02 | | 0.18 | | 0.00 | | 0.00 | | 0.04 | | 0.13 | | 0.11 | 0.00 | | 0.00 | | 0.00 | | 0.00 | | 0.00 | | 0.00 | | 0.00 | | 0.00 | | 0.00 | 0.04 | | 0.00 | | 0.50 | | 0.14 | |
| cnvr115 | 0.11 | | 0.08 | | 0.15 | | 0.29 | | 0.00 | | 0.40 | | 0.13 | | 0.32 | 0.22 | | 0.25 | | 0.29 | | 0.17 | | 0.00 | | 0.00 | | 0.00 | | 0.35 | | 0.16 | 0.39 | | 0.22 | | 0.00 | | 0.00 | |
| cnvr122 | 0.00 | | 0.05 | | 0.03 | | 0.21 | | 0.00 | | 0.04 | | 0.00 | | 0.00 | 0.00 | | 0.00 | | 0.00 | | 0.00 | | 0.00 | | 0.00 | | 0.00 | | 0.00 | | 0.00 | 0.00 | | 0.00 | | 0.00 | | 0.00 | |
| cnvr132 | 0.00 | | 0.00 | | 0.00 | | 0.00 | | 0.00 | | 0.00 | | 0.00 | | 0.00 | 0.00 | | 0.00 | | 0.00 | | 0.00 | | 0.00 | | 0.00 | | 0.29 | | 0.00 | | 0.11 | 0.00 | | 0.00 | | 0.00 | | 0.00 | |
| cnvr146 | 0.00 | | 0.00 | | 0.00 | | 0.00 | | 0.00 | | 0.00 | | 0.25 | | 0.00 | 0.00 | | 0.00 | | 0.00 | | 0.00 | | 0.00 | | 0.00 | | 0.00 | | 0.00 | | 0.00 | 0.00 | | 0.00 | | 0.00 | | 0.00 | |
| cnvr148 | 0.04 | | 0.00 | | 0.10 | | 0.00 | | 0.00 | | 0.16 | | 0.00 | | 0.05 | 0.04 | | 0.00 | | 0.00 | | 0.00 | | 0.00 | | 0.00 | | 0.00 | | 0.04 | | 0.00 | 0.00 | | 0.00 | | 0.06 | | 0.00 | |
| cnvr151 | 0.00 | | 0.00 | | 0.00 | | 0.00 | | 0.00 | | 0.32 | | 0.00 | | 0.00 | 0.00 | | 0.00 | | 0.00 | | 0.00 | | 0.00 | | 0.00 | | 0.00 | | 0.00 | | 0.00 | 0.00 | | 0.00 | | 0.00 | | 0.00 | |
| cnvr158 | 0.00 | | 0.00 | | 0.00 | | 0.00 | | 0.00 | | 0.00 | | 0.00 | | 0.00 | 0.00 | | 0.00 | | 0.00 | | 0.00 | | 0.00 | | 0.00 | | 0.00 | | 0.22 | | 0.00 | 0.04 | | 0.13 | | 0.00 | | 0.00 | |
| cnvr159 | 0.00 | | 0.00 | | 0.00 | | 0.00 | | 0.00 | | 0.00 | | 0.00 | | 0.00 | 0.00 | | 0.00 | | 0.00 | | 0.00 | | 0.00 | | 0.00 | | 0.12 | | 0.17 | | 0.00 | 0.00 | | 0.00 | | 0.00 | | 0.00 | |
| cnvr162 | 0.25 | | 0.19 | | 0.23 | | 0.08 | | 0.04 | | 0.16 | | 0.00 | | 0.05 | 0.26 | | 0.10 | | 0.33 | | 0.17 | | 0.33 | | 0.00 | | 0.29 | | 0.22 | | 0.21 | 0.13 | | 0.17 | | 0.06 | | 0.29 | |
| cnvr175 | 0.00 | | 0.00 | | 0.00 | | 0.00 | | 0.00 | | 0.00 | | 0.00 | | 0.00 | 0.00 | | 0.00 | | 0.00 | | 0.00 | | 0.00 | | 0.00 | | 0.00 | | 0.17 | | 0.00 | 0.00 | | 0.00 | | 0.00 | | 0.00 | |
| cnvr178 | 0.26 | | 0.32 | | 0.20 | | 0.29 | | 0.19 | | 0.16 | | 0.13 | | 0.16 | 0.09 | | 0.20 | | 0.24 | | 0.00 | | 0.00 | | 0.00 | | 0.41 | | 0.17 | | 0.26 | 0.35 | | 0.30 | | 0.22 | | 0.29 | |
| cnvr186 | 0.18 | | 0.15 | | 0.43 | | 0.21 | | 0.33 | | 0.32 | | 0.46 | | 0.16 | 0.04 | | 0.25 | | 0.05 | | 0.08 | | 0.00 | | 1.00 | | 0.47 | | 0.17 | | 0.05 | 0.26 | | 0.09 | | 0.39 | | 0.36 | |
| cnvr194 | 0.04 | | 0.05 | | 0.05 | | 0.04 | | 0.00 | | 0.08 | | 0.33 | | 0.11 | 0.04 | | 0.00 | | 0.05 | | 0.00 | | 0.33 | | 0.00 | | 0.00 | | 0.00 | | 0.00 | 0.00 | | 0.00 | | 0.00 | | 0.00 | |
| cnvr211 | 0.00 | | 0.00 | | 0.00 | | 0.00 | | 0.00 | | 0.00 | | 0.00 | | 0.00 | 0.00 | | 0.00 | | 0.00 | | 0.00 | | 0.00 | | 0.00 | | 0.29 | | 0.09 | | 0.11 | 0.04 | | 0.00 | | 0.00 | | 0.00 | |
| cnvr214 | 0.00 | | 0.00 | | 0.00 | | 0.00 | | 0.00 | | 0.00 | | 0.00 | | 0.00 | 0.13 | | 0.00 | | 0.00 | | 0.00 | | 0.00 | | 0.00 | | 0.00 | | 0.00 | | 0.00 | 0.00 | | 0.00 | | 0.00 | | 0.07 | |
| cnvr220 | 0.00 | | 0.00 | | 0.00 | | 0.13 | | 0.00 | | 0.00 | | 0.00 | | 0.00 | 0.00 | | 0.00 | | 0.00 | | 0.00 | | 0.00 | | 0.00 | | 0.00 | | 0.00 | | 0.00 | 0.00 | | 0.00 | | 0.00 | | 0.00 | |
| cnvr231 | 0.00 | | 0.00 | | 0.00 | | 0.00 | | 0.00 | | 0.12 | | 0.00 | | 0.00 | 0.00 | | 0.00 | | 0.00 | | 0.00 | | 0.00 | | 0.00 | | 0.00 | | 0.00 | | 0.00 | 0.00 | | 0.00 | | 0.00 | | 0.00 | |
| cnvr241 | 0.05 | | 0.00 | | 0.00 | | 0.04 | | 0.15 | | 0.04 | | 0.00 | | 0.00 | 0.04 | | 0.00 | | 0.00 | | 0.08 | | 0.00 | | 0.00 | | 0.18 | | 0.00 | | 0.00 | 0.22 | | 0.09 | | 0.06 | | 0.07 | |
| cnvr243 | 0.00 | | 0.00 | | 0.00 | | 0.00 | | 0.00 | | 0.00 | | 0.00 | | 0.21 | 0.00 | | 0.00 | | 0.00 | | 0.00 | | 0.00 | | 0.00 | | 0.00 | | 0.00 | | 0.00 | 0.00 | | 0.00 | | 0.00 | | 0.00 | |
| cnvr245 | 0.09 | | 0.00 | | 0.00 | | 0.00 | | 0.00 | | 0.08 | | 0.13 | | 0.00 | 0.00 | | 0.00 | | 0.00 | | 0.00 | | 0.00 | | 0.00 | | 0.29 | | 0.30 | | 0.21 | 0.26 | | 0.00 | | 0.00 | | 0.21 | |
| cnvr253 | 0.02 | | 0.05 | | 0.05 | | 0.08 | | 0.11 | | 0.28 | | 0.00 | | 0.00 | 0.04 | | 0.35 | | 0.05 | | 0.00 | | 0.00 | | 0.00 | | 0.18 | | 0.17 | | 0.11 | 0.00 | | 0.09 | | 0.22 | | 0.14 | |
| cnvr266 | 0.02 | | 0.00 | | 0.00 | | 0.04 | | 0.00 | | 0.00 | | 0.17 | | 0.05 | 0.00 | | 0.05 | | 0.00 | | 0.00 | | 0.00 | | 0.00 | | 0.00 | | 0.00 | | 0.05 | 0.00 | | 0.00 | | 0.00 | | 0.00 | |
| cnvr270 | 0.00 | | 0.00 | | 0.03 | | 0.00 | | 0.00 | | 0.00 | | 0.00 | | 0.05 | 0.00 | | 0.00 | | 0.05 | | 0.00 | | 0.00 | | 0.33 | | 0.18 | | 0.00 | | 0.05 | 0.00 | | 0.00 | | 0.00 | | 0.00 | |
| cnvr280 | 0.00 | | 0.00 | | 0.00 | | 0.04 | | 0.00 | | 0.00 | | 0.00 | | 0.00 | 0.00 | | 0.00 | | 0.00 | | 0.00 | | 0.33 | | 0.00 | | 0.06 | | 0.09 | | 0.00 | 0.00 | | 0.17 | | 0.00 | | 0.00 | |
| cnvr295 | 0.00 | | 0.00 | | 0.00 | | 0.00 | | 0.00 | | 0.00 | | 0.00 | | 0.00 | 0.00 | | 0.00 | | 0.00 | | 0.00 | | 0.00 | | 0.00 | | 0.12 | | 0.22 | | 0.05 | 0.00 | | 0.00 | | 0.06 | | 0.00 | |
| cnvr318 | 0.00 | | 0.00 | | 0.00 | | 0.04 | | 0.00 | | 0.00 | | 0.00 | | 0.00 | 0.13 | | 0.00 | | 0.00 | | 0.00 | | 0.00 | | 0.00 | | 0.00 | | 0.17 | | 0.16 | 0.00 | | 0.00 | | 0.00 | | 0.00 | |
| cnvr319 | 0.19 | | 0.14 | | 0.40 | | 0.33 | | 0.04 | | 0.04 | | 0.08 | | 0.16 | 0.26 | | 0.30 | | 0.14 | | 0.17 | | 0.33 | | 0.33 | | 0.41 | | 0.39 | | 0.26 | 0.39 | | 0.35 | | 0.17 | | 0.36 | |
| cnvr337 | 0.00 | | 0.00 | | 0.00 | | 0.00 | | 0.00 | | 0.00 | | 0.04 | | 0.00 | 0.00 | | 0.00 | | 0.00 | | 0.00 | | 0.00 | | 0.00 | | 0.00 | | 0.17 | | 0.00 | 0.00 | | 0.00 | | 0.11 | | 0.00 | |
| cnvr353 | 0.00 | | 0.00 | | 0.00 | | 0.25 | | 0.00 | | 0.00 | | 0.00 | | 0.00 | 0.00 | | 0.00 | | 0.00 | | 0.00 | | 0.00 | | 0.00 | | 0.00 | | 0.00 | | 0.00 | 0.09 | | 0.00 | | 0.00 | | 0.00 | |
| cnvr360 | 0.00 | | 0.00 | | 0.00 | | 0.00 | | 0.00 | | 0.00 | | 0.00 | | 0.00 | 0.00 | | 0.00 | | 0.00 | | 0.00 | | 0.00 | | 0.00 | | 0.24 | | 0.00 | | 0.05 | 0.04 | | 0.00 | | 0.00 | | 0.00 | |
| cnvr364 | 0.00 | | 0.00 | | 0.00 | | 0.00 | | 0.00 | | 0.00 | | 0.00 | | 0.00 | 0.00 | | 0.00 | | 0.19 | | 0.00 | | 0.00 | | 0.00 | | 0.00 | | 0.00 | | 0.00 | 0.00 | | 0.00 | | 0.00 | | 0.00 | |
| cnvr366 | 0.00 | | 0.00 | | 0.00 | | 0.00 | | 0.00 | | 0.00 | | 0.00 | | 0.00 | 0.00 | | 0.00 | | 0.00 | | 0.00 | | 0.00 | | 0.00 | | 0.24 | | 0.30 | | 0.32 | 0.00 | | 0.00 | | 0.00 | | 0.14 | |
| cnvr371 | 0.02 | | 0.00 | | 0.00 | | 0.00 | | 0.00 | | 0.04 | | 0.00 | | 0.00 | 0.00 | | 0.00 | | 0.00 | | 0.00 | | 0.00 | | 0.00 | | 0.00 | | 0.13 | | 0.11 | 0.00 | | 0.09 | | 0.00 | | 0.00 | |
| cnvr376 | 0.02 | | 0.03 | | 0.03 | | 0.00 | | 0.04 | | 0.00 | | 0.04 | | 0.00 | 0.00 | | 0.00 | | 0.24 | | 0.00 | | 0.00 | | 0.00 | | 0.06 | | 0.00 | | 0.00 | 0.00 | | 0.09 | | 0.00 | | 0.07 | |
| cnvr380 | 0.11 | | 0.08 | | 0.10 | | 0.08 | | 0.07 | | 0.00 | | 0.04 | | 0.11 | 0.00 | | 0.15 | | 0.05 | | 0.00 | | 0.00 | | 0.00 | | 0.00 | | 0.09 | | 0.00 | 0.26 | | 0.17 | | 0.00 | | 0.00 | |
| cnvr381 | 0.49 | | 0.44 | | 0.65 | | 0.46 | | 0.44 | | 0.40 | | 0.38 | | 0.53 | 0.70 | | 0.60 | | 0.24 | | 0.25 | | 0.33 | | 0.33 | | 0.71 | | 0.61 | | 0.26 | 0.39 | | 0.57 | | 0.61 | | 0.43 | |
| cnvr386 | 0.00 | | 0.00 | | 0.00 | | 0.00 | | 0.00 | | 0.00 | | 0.04 | | 0.00 | 0.00 | | 0.00 | | 0.00 | | 0.00 | | 0.00 | | 0.00 | | 0.18 | | 0.09 | | 0.00 | 0.09 | | 0.00 | | 0.00 | | 0.00 | |
| cnvr404 | 0.00 | | 0.00 | | 0.00 | | 0.00 | | 0.00 | | 0.00 | | 0.00 | | 0.00 | 0.00 | | 0.00 | | 0.00 | | 0.33 | | 0.00 | | 0.00 | | 0.00 | | 0.00 | | 0.00 | 0.00 | | 0.00 | | 0.00 | | 0.00 | |
| cnvr407 | 0.44 | | 0.46 | | 0.35 | | 0.46 | | 0.22 | | 0.16 | | 0.42 | | 0.26 | 0.39 | | 0.45 | | 0.19 | | 0.33 | | 0.67 | | 0.33 | | 0.82 | | 0.35 | | 0.32 | 0.48 | | 0.26 | | 0.28 | | 0.64 | |
| cnvr408 | 0.00 | | 0.00 | | 0.00 | | 0.00 | | 0.00 | | 0.00 | | 0.00 | | 0.00 | 0.00 | | 0.00 | | 0.00 | | 0.00 | | 0.00 | | 0.00 | | 0.00 | | 0.17 | | 0.00 | 0.00 | | 0.00 | | 0.00 | | 0.00 | |
| cnvr424 | 0.00 | | 0.00 | | 0.00 | | 0.04 | | 0.00 | | 0.00 | | 0.00 | | 0.16 | 0.00 | | 0.05 | | 0.00 | | 0.00 | | 0.00 | | 0.00 | | 0.00 | | 0.00 | | 0.00 | 0.00 | | 0.00 | | 0.00 | | 0.00 | |
| cnvr434 | 0.02 | | 0.02 | | 0.00 | | 0.04 | | 0.00 | | 0.08 | | 0.00 | | 0.00 | 0.00 | | 0.00 | | 0.19 | | 0.00 | | 0.00 | | 0.00 | | 0.00 | | 0.00 | | 0.00 | 0.00 | | 0.00 | | 0.00 | | 0.00 | |
| cnvr449 | 0.00 | | 0.02 | | 0.00 | | 0.00 | | 0.00 | | 0.00 | | 0.00 | | 0.16 | 0.00 | | 0.00 | | 0.00 | | 0.00 | | 0.00 | | 0.00 | | 0.06 | | 0.00 | | 0.00 | 0.04 | | 0.00 | | 0.00 | | 0.00 | |
| cnvr454 | 0.40 | | 0.32 | | 0.40 | | 0.38 | | 0.00 | | 0.28 | | 0.38 | | 0.37 | 0.30 | | 0.45 | | 0.19 | | 0.33 | | 0.00 | | 0.67 | | 0.29 | | 0.22 | | 0.11 | 0.26 | | 0.35 | | 1.17 | | 0.64 | |
| cnvr455 | 0.18 | | 0.15 | | 0.13 | | 0.17 | | 0.15 | | 0.08 | | 0.04 | | 0.11 | 0.00 | | 0.05 | | 0.14 | | 0.25 | | 0.00 | | 0.33 | | 0.00 | | 0.00 | | 0.00 | 0.00 | | 0.00 | | 0.00 | | 0.00 | |
| cnvr456 | 0.05 | | 0.03 | | 0.13 | | 0.13 | | 0.07 | | 0.04 | | 0.08 | | 0.05 | 0.00 | | 0.00 | | 0.10 | | 0.08 | | 0.00 | | 0.00 | | 0.06 | | 0.04 | | 0.11 | 0.26 | | 0.09 | | 0.00 | | 0.07 | |
| cnvr457 | 0.00 | | 0.00 | | 0.00 | | 0.00 | | 0.00 | | 0.00 | | 0.00 | | 0.00 | 0.00 | | 0.00 | | 0.00 | | 0.00 | | 0.00 | | 0.00 | | 0.18 | | 0.00 | | 0.05 | 0.00 | | 0.00 | | 0.00 | | 0.00 | |
| cnvr459 | 0.11 | | 0.03 | | 0.03 | | 0.04 | | 0.04 | | 0.04 | | 0.00 | | 0.00 | 0.00 | | 0.20 | | 0.14 | | 0.25 | | 0.00 | | 0.00 | | 0.00 | | 0.04 | | 0.00 | 0.00 | | 0.04 | | 0.06 | | 0.21 | |
| cnvr470 | 0.02 | | 0.00 | | 0.00 | | 0.00 | | 0.00 | | 0.00 | | 0.00 | | 0.00 | 0.00 | | 0.00 | | 0.00 | | 0.00 | | 0.00 | | 0.00 | | 0.00 | | 0.09 | | 0.16 | 0.09 | | 0.09 | | 0.00 | | 0.00 | |
| cnvr485 | 0.05 | | 0.03 | | 0.00 | | 0.00 | | 0.00 | | 0.00 | | 0.00 | | 0.11 | 0.00 | | 0.00 | | 0.24 | | 0.00 | | 0.00 | | 0.00 | | 0.12 | | 0.13 | | 0.00 | 0.04 | | 0.09 | | 0.06 | | 0.00 | |
| cnvr494 | 0.04 | | 0.05 | | 0.00 | | 0.00 | | 0.00 | | 0.12 | | 0.00 | | 0.00 | 0.00 | | 0.00 | | 0.10 | | 0.00 | | 0.00 | | 0.00 | | 0.12 | | 0.13 | | 0.00 | 0.00 | | 0.09 | | 0.00 | | 0.14 | |
| cnvr498 | 0.12 | | 0.00 | | 0.00 | | 0.00 | | 0.00 | | 0.00 | | 0.00 | | 0.00 | 0.00 | | 0.00 | | 0.00 | | 0.00 | | 0.00 | | 0.00 | | 0.00 | | 0.00 | | 0.00 | 0.00 | | 0.00 | | 0.00 | | 0.00 | |
| cnvr506 | 0.04 | | 0.02 | | 0.03 | | 0.21 | | 0.00 | | 0.08 | | 0.00 | | 0.11 | 0.09 | | 0.00 | | 0.14 | | 0.00 | | 0.00 | | 0.00 | | 0.00 | | 0.00 | | 0.00 | 0.04 | | 0.00 | | 0.00 | | 0.00 | |
| cnvr508 | 0.04 | | 0.00 | | 0.00 | | 0.04 | | 0.00 | | 0.00 | | 0.00 | | 0.00 | 0.04 | | 0.00 | | 0.00 | | 0.00 | | 0.00 | | 0.00 | | 0.12 | | 0.22 | | 0.00 | 0.00 | | 0.00 | | 0.06 | | 0.00 | |
| cnvr509 | 0.02 | | 0.03 | | 0.08 | | 0.17 | | 0.07 | | 0.00 | | 0.00 | | 0.21 | 0.22 | | 0.00 | | 0.00 | | 0.00 | | 0.00 | | 0.33 | | 0.76 | | 0.70 | | 0.74 | 0.13 | | 0.22 | | 0.00 | | 0.50 | |
| cnvr514 | 0.18 | | 0.00 | | 0.10 | | 0.21 | | 0.11 | | 0.04 | | 0.13 | | 0.21 | 0.13 | | 0.00 | | 0.00 | | 0.00 | | 0.00 | | 0.33 | | 0.00 | | 0.00 | | 0.05 | 0.04 | | 0.00 | | 0.00 | | 0.07 | |
| cnvr521 | 0.00 | | 0.00 | | 0.03 | | 0.13 | | 0.00 | | 0.00 | | 0.00 | | 0.00 | 0.00 | | 0.00 | | 0.00 | | 0.00 | | 0.00 | | 0.00 | | 0.00 | | 0.00 | | 0.00 | 0.00 | | 0.00 | | 0.00 | | 0.00 | |
| cnvr529 | 0.00 | | 0.00 | | 0.00 | | 0.00 | | 0.00 | | 0.00 | | 0.00 | | 0.00 | 0.04 | | 0.00 | | 0.00 | | 0.00 | | 0.00 | | 0.00 | | 0.24 | | 0.09 | | 0.16 | 0.09 | | 0.00 | | 0.00 | | 0.00 | |
| cnvr543 | 0.02 | | 0.00 | | 0.00 | | 0.00 | | 0.07 | | 0.00 | | 0.00 | | 0.00 | 0.00 | | 0.00 | | 0.00 | | 0.00 | | 0.00 | | 0.00 | | 0.18 | | 0.09 | | 0.00 | 0.00 | | 0.00 | | 0.00 | | 0.00 | |
| cnvr558 | 0.00 | | 0.00 | | 0.00 | | 0.00 | | 0.00 | | 0.00 | | 0.00 | | 0.00 | 0.00 | | 0.00 | | 0.00 | | 0.00 | | 0.00 | | 0.00 | | 0.00 | | 0.26 | | 0.00 | 0.00 | | 0.00 | | 0.00 | | 0.00 | |
| cnvr562 | 0.00 | | 0.00 | | 0.00 | | 0.00 | | 0.00 | | 0.00 | | 0.08 | | 0.05 | 0.00 | | 0.00 | | 0.05 | | 0.00 | | 0.00 | | 0.00 | | 0.00 | | 0.17 | | 0.05 | 0.00 | | 0.00 | | 0.00 | | 0.07 | |
| cnvr568 | 0.00 | | 0.00 | | 0.00 | | 0.00 | | 0.00 | | 0.00 | | 0.00 | | 0.00 | 0.00 | | 0.00 | | 0.00 | | 0.00 | | 0.00 | | 0.00 | | 0.00 | | 0.22 | | 0.00 | 0.00 | | 0.00 | | 0.00 | | 0.00 | |
| cnvr574 | 0.00 | | 0.00 | | 0.00 | | 0.00 | | 0.00 | | 0.00 | | 0.00 | | 0.00 | 0.00 | | 0.00 | | 0.00 | | 0.00 | | 0.00 | | 0.00 | | 0.18 | | 0.22 | | 0.11 | 0.00 | | 0.00 | | 0.00 | | 0.07 | |
| cnvr584 | 0.02 | | 0.00 | | 0.00 | | 0.00 | | 0.00 | | 0.00 | | 0.00 | | 0.00 | 0.00 | | 0.00 | | 0.00 | | 0.00 | | 0.00 | | 0.00 | | 0.12 | | 0.13 | | 0.00 | 0.04 | | 0.00 | | 0.00 | | 0.00 | |
| cnvr586 | 0.04 | | 0.00 | | 0.13 | | 0.21 | | 0.00 | | 0.04 | | 0.00 | | 0.05 | 0.22 | | 0.05 | | 0.00 | | 0.17 | | 0.33 | | 0.00 | | 0.06 | | 0.00 | | 0.00 | 0.00 | | 0.00 | | 0.00 | | 0.00 | |
| cnvr606 | 0.02 | | 0.00 | | 0.00 | | 0.00 | | 0.00 | | 0.00 | | 0.00 | | 0.00 | 0.00 | | 0.00 | | 0.00 | | 0.00 | | 0.00 | | 0.00 | | 0.00 | | 0.00 | | 0.00 | 0.00 | | 0.17 | | 0.00 | | 0.00 | |
| cnvr631 | 0.00 | | 0.00 | | 0.00 | | 0.00 | | 0.00 | | 0.00 | | 0.00 | | 0.00 | 0.00 | | 0.00 | | 0.00 | | 0.00 | | 0.00 | | 0.00 | | 0.00 | | 0.00 | | 0.16 | 0.00 | | 0.04 | | 0.00 | | 0.00 | |
| cnvr637 | 0.02 | | 0.05 | | 0.08 | | 0.00 | | 0.00 | | 0.20 | | 0.00 | | 0.05 | 0.00 | | 0.00 | | 0.19 | | 0.25 | | 0.00 | | 0.00 | | 0.12 | | 0.00 | | 0.00 | 0.00 | | 0.00 | | 0.06 | | 0.07 | |
| cnvr642 | 0.00 | | 0.00 | | 0.00 | | 0.00 | | 0.00 | | 0.00 | | 0.00 | | 0.00 | 0.00 | | 0.00 | | 0.00 | | 0.00 | | 0.00 | | 0.00 | | 0.06 | | 0.13 | | 0.00 | 0.09 | | 0.00 | | 0.00 | | 0.07 | |
| cnvr647 | 0.00 | | 0.00 | | 0.00 | | 0.00 | | 0.00 | | 0.00 | | 0.00 | | 0.00 | 0.00 | | 0.00 | | 0.00 | | 0.00 | | 0.00 | | 0.00 | | 0.24 | | 0.00 | | 0.00 | 0.00 | | 0.00 | | 0.00 | | 0.00 | |
| cnvr656 | 0.00 | | 0.00 | | 0.00 | | 0.00 | | 0.00 | | 0.04 | | 0.00 | | 0.00 | 0.00 | | 0.00 | | 0.00 | | 0.00 | | 0.00 | | 0.00 | | 0.00 | | 0.00 | | 0.00 | 0.30 | | 0.00 | | 0.00 | | 0.07 | |
| cnvr670 | 0.00 | | 0.02 | | 0.03 | | 0.00 | | 0.00 | | 0.00 | | 0.00 | | 0.00 | 0.00 | | 0.05 | | 0.05 | | 0.00 | | 0.00 | | 0.00 | | 0.29 | | 0.30 | | 0.32 | 0.00 | | 0.09 | | 0.00 | | 0.14 | |
| cnvr673 | 0.00 | | 0.00 | | 0.10 | | 0.00 | | 0.00 | | 0.04 | | 0.00 | | 0.05 | 0.09 | | 0.00 | | 0.00 | | 0.00 | | 0.00 | | 0.00 | | 0.00 | | 0.00 | | 0.00 | 0.00 | | 0.00 | | 0.00 | | 0.00 | |
| cnvr682 | 0.04 | | 0.00 | | 0.05 | | 0.04 | | 0.00 | | 0.00 | | 0.00 | | 0.00 | 0.00 | | 0.00 | | 0.00 | | 0.00 | | 0.00 | | 0.00 | | 0.00 | | 0.17 | | 0.00 | 0.00 | | 0.00 | | 0.00 | | 0.00 | |


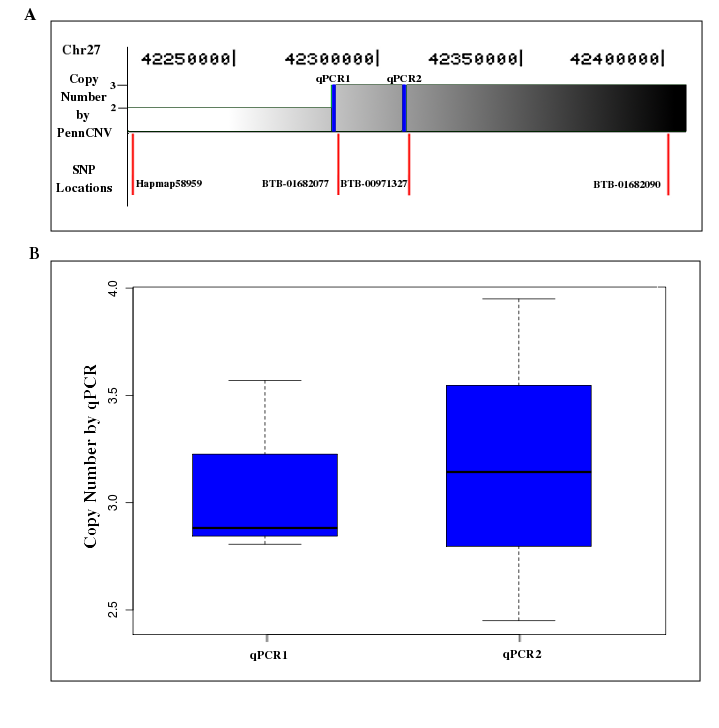


**Figure S1. Illustration of a typical CNV call with qPCR validation.** (A) Chromosome and coordinate positions of SNP markers and PennCNV predictions for CNVR 645 (Additional file 1: Table S2). Nucleotide positions are labeled on the top. SNP marker positions are depicted as long red lines at the bottom. The shaded histogram in the middle represents the copy number prediction by the PennCNV program. Blue bars on the histogram represent the positions chosen for qPCR validation of the CNVR. (B) Whisker plot of qPCR validation experiments for CNVR 645. The Y axis represents the copy number value predicted by a ddCT comparsion of qPCR CT values with CT values from the same locus in Dominette.
